# Diretriz sobre Diagnóstico e Tratamento da Cardiomiopatia Hipertrófica – 2024

**DOI:** 10.36660/abc.20240415

**Published:** 2024-06-20

**Authors:** Fabio Fernandes, Marcus V. Simões, Edileide de Barros Correia, Fabiana Goulart Marcondes-Braga, Otavio Rizzi Coelho, Cláudio Tinoco Mesquita, Wilson Mathias, Murillo de Oliveira Antunes, Edmundo Arteaga-Fernández, Carlos Eduardo Rochitte, Felix José Alvarez Ramires, Silvia Marinho Martins Alves, Marcelo Westerlund Montera, Renato Delascio Lopes, Mucio Tavares de Oliveira, Fernando Luis Scolari, Walkiria Samuel Avila, Manoel Fernandes Canesin, Edimar Alcides Bocchi, Fernando Bacal, Lidia Zytynski Moura, Eduardo Benchimol Saad, Mauricio Ibrahim Scanavacca, Bruno Pereira Valdigem, Manuel Nicolas Cano, Alexandre Antonio Cunha Abizaid, Henrique Barbosa Ribeiro, Pedro Alves Lemos, Gustavo Calado de Aguiar Ribeiro, Fabio Biscegli Jatene, Ricardo Ribeiro Dias, Luis Beck-da-Silva, Luis Eduardo Paim Rohde, Marcelo Imbroinise Bittencourt, Alexandre da Costa Pereira, José Eduardo Krieger, Humberto Villacorta, Wolney de Andrade Martins, José Albuquerque de Figueiredo, Juliano Novaes Cardoso, Carlos Alberto Pastore, Ieda Biscegli Jatene, Ana Cristina Sayuri Tanaka, Viviane Tiemi Hotta, Minna Moreira Dias Romano, Denilson Campos de Albuquerque, Ricardo Mourilhe-Rocha, Ludhmila Abrahão Hajjar, Fabio Sandoli de Brito, Bruno Caramelli, Daniela Calderaro, Pedro Silvio Farsky, Alexandre Siciliano Colafranceschi, Ibraim Masciarelli Francisco Pinto, Marcelo Luiz Campos Vieira, Luiz Claudio Danzmann, Silvio Henrique Barberato, Charles Mady, Martino Martinelli, Ana Flavia Malheiros Torbey, Pedro Vellosa Schwartzmann, Ariane Vieira Scarlatelli Macedo, Silvia Moreira Ayub Ferreira, Andre Schmidt, Marcelo Dantas Tavares de Melo, Moysés Oliveira Lima, Andrei C. Sposito, Flávio de Souza Brito, Andreia Biolo, Vagner Madrini, Stephanie Itala Rizk, Evandro Tinoco Mesquita

**Affiliations:** 1 Universidade de São Paulo Hospital das Clínicas da Faculdade de Medicina Instituto do Coração São Paulo SP Brasil Instituto do Coração (InCor) do Hospital das Clínicas da Faculdade de Medicina da Universidade de São Paulo (HCFMUSP), São Paulo, SP – Brasil; 2 Universidade de São Paulo Faculdade de Medicina de Ribeirão Preto Ribeirão Preto SP Brasil Faculdade de Medicina de Ribeirão Preto da Universidade de São Paulo, Ribeirão Preto, SP – Brasil; 3 Governo do Estado de São Paulo São Paulo SP Brasil Governo do Estado de São Paulo, São Paulo, SP – Brasil; 4 Universidade Estadual de Campinas Campinas SP Brasil Universidade Estadual de Campinas (UNICAMP), Campinas, SP – Brasil; 5 Universidade Federal Fluminense Rio de Janeiro RJ Brasil Universidade Federal Fluminense (UFF), Rio de Janeiro, RJ – Brasil; 6 Universidade São Francisco São Paulo SP Brasil Universidade São Francisco (USF), São Paulo, SP – Brasil; Pronto Socorro Cardiológico de Pernambuco (PROCAPE), Recife, PE – Brasil; Pronto Socorro Cardiológico de Pernambuco Recife PE Brasil; 7 Universidade de Pernambuco Recife PE Brasil Universidade de Pernambuco (UPE), Recife, PE – Brasil; 8 Hospital Pró-Cardíaco Rio de Janeiro RJ Brasil Hospital Pró-Cardíaco, Rio de Janeiro, RJ – Brasil; 9 Duke University Durham EUA Duke University, Durham – EUA; 10 Hospital de Clínicas de Porto Alegre Porto Alegre RS Brasil Hospital de Clínicas de Porto Alegre, Porto Alegre, RS – Brasil; 11 Universidade Estadual de Londrina Londrina PR Brasil Universidade Estadual de Londrina, Londrina, PR – Brasil; 12 Universidade de São Paulo São Paulo SP Brasil Universidade de São Paulo (USP), São Paulo, SP – Brasil; 13 Pontifícia Universidade Católica de Curitiba Curitiba PR Brasil Pontifícia Universidade Católica de Curitiba, Curitiba, PR – Brasil; 14 Hospital Samaritano Rio de Janeiro RJ Brasil Hospital Samaritano, Rio de Janeiro, RJ – Brasil; 15 Beth Israel Deaconess Medical Center Harvard Medical School Boston USA Beth Israel Deaconess Medical Center / Harvard Medical School, Boston – USA; 16 Instituto Dante Pazzanese de Cardiologia São Paulo SP Brasil Instituto Dante Pazzanese de Cardiologia, São Paulo, SP – Brasil; 17 Hospital do Coração São Paulo SP Brasil Hospital do Coração (Hcor), São Paulo, SP – Brasil; 18 Hospital Israelita Albert Einstein São Paulo SP Brasil Hospital Israelita Albert Einstein, São Paulo, SP – Brasil; 19 Pontifícia Universidade Católica de Campinas Campinas SP Brasil Pontifícia Universidade Católica de Campinas (PUCC), Campinas, SP – Brasil; 20 Fundação Zerbini São Paulo SP Brasil Fundação Zerbini, São Paulo, SP – Brasil; 21 Universidade Federal do Rio Grande do Sul Porto Alegre RS Brasil Universidade Federal do Rio Grande do Sul (UFRGS), Porto Alegre, RS – Brasil; 22 Universidade do Estado do Rio de Janeiro Rio de Janeiro RJ Brasil Universidade do Estado do Rio de Janeiro (UERJ), Rio de Janeiro, RJ – Brasil; 23 Universidade Federal do Maranhão Hospital Universitário Presidente Dutra São Luís MA Brasil Hospital Universitário Presidente Dutra, Universidade Federal do Maranhão (UFMA), São Luís, MA – Brasil; 24 Faculdade Santa Marcelina São Paulo SP Brasil Faculdade Santa Marcelina, São Paulo, SP – Brasil; 25 Fleury Medicina e Saúde São Paulo SP Brasil Fleury Medicina e Saúde, São Paulo, SP – Brasil; 26 Instituto D’Or de Pesquisa e Ensino Rio de Janeiro RJ Brasil Instituto D’Or de Pesquisa e Ensino (IDOR), Rio de Janeiro, RJ – Brasil; 27 Instituto Nacional de Cardiologia Rio de Janeiro RJ Brasil Instituto Nacional de Cardiologia, Rio de Janeiro, RJ – Brasil; 28 Universidade Luterana do Brasil Canoas RS Brasil Universidade Luterana do Brasil, Canoas, RS – Brasil; 29 CardioEco Centro de Diagnóstico Cardiovascular e Ecocardiografia Curitiba PR Brasil CardioEco Centro de Diagnóstico Cardiovascular e Ecocardiografia, Curitiba, PR – Brasil; 30 Quanta Diagnósticos Curitiba PR Brasil Quanta Diagnósticos, Curitiba, PR – Brasil; 31 Hospital Unimed Ribeirão Preto Ribeirão Preto SP Brasil Hospital Unimed Ribeirão Preto, Ribeirão Preto, SP – Brasil; 32 Centro Avançado de Pesquisa, Ensino e Diagnóstico Ribeirão Preto SP Brasil Centro Avançado de Pesquisa, Ensino e Diagnóstico (CAPED), Ribeirão Preto, SP – Brasil; 33 Santa Casa de Misericórdia de São Paulo São Paulo SP Brasil Santa Casa de Misericórdia de São Paulo, São Paulo, SP – Brasil; 34 Universidade Federal da Paraíba João Pessoa PB Brasil Universidade Federal da Paraíba, João Pessoa, PB – Brasil; 35 Universidade de São Paulo Hospital das Clínicas de Ribeirão Preto Ribeirão Preto SP Brasil Hospital das Clínicas de Ribeirão Preto da Universidade de São Paulo, Ribeirão Preto, SP – Brasil; 36 Hospital Vera Cruz Campinas SP Brasil Hospital Vera Cruz, Campinas, SP – Brasil; 37 Universidade Estadual Paulista "Júlio de Mesquita Filho" Hospital das Clínicas da Faculdade de Medicina de Botucatu São Paulo SP Brasil Hospital das Clínicas da Faculdade de Medicina de Botucatu, Universidade Estadual Paulista "Júlio de Mesquita Filho" (UNESP), São Paulo, SP – Brasil; 38 Centro de Pesquisa Clínica – Indacor São Paulo SP Brasil Centro de Pesquisa Clínica – Indacor, São Paulo, SP – Brasil; 39 Hospital Moinhos de Vento Porto Alegre RS Brasil Hospital Moinhos de Vento, Porto Alegre, RS – Brasil; 40 Hospital de Clínicas de Porto Alegre Porto Alegre RS Brasil Hospital de Clínicas de Porto Alegre (HCPA), Porto Alegre, RS – Brasil

**Table:** 

Diretriz sobre Diagnóstico e Tratamento da Cardiomiopatia Hipertrófica – 2024	
O relatório abaixo lista as declarações de interesse conforme relatadas à SBC pelos especialistas durante o período de desenvolvimento deste posicionamento, 2022-2024.	
**Especialista**	**Tipo de relacionamento com a indústria**
Alexandre Antonio Cunha Abizaid	Nada a ser declarado
Alexandre da Costa Pereira	Nada a ser declarado
Alexandre Siciliano Colafranceschi	Nada a ser declarado
Ana Cristina Sayuri Tanaka	Nada a ser declarado
Ana Flávia Malheiros Torbey	Nada a ser declarado
Andre Schmidt	Nada a ser declarado
Andrei C. Sposito	Nada a ser declarado
Andreia Biolo	Declaração financeira B - Financiamento de pesquisas sob sua responsabilidade direta/pessoal (direcionado ao departamento ou instituição) provenientes da indústria farmacêutica, de órteses, próteses, equipamentos e implantes, brasileiras ou estrangeiras: - Anylam: amiloidose.
Ariane Vieira Scarlatelli Macedo	Declaração financeira A - Pagamento de qualquer espécie e desde que economicamente apreciáveis, feitos a (i) você, (ii) ao seu cônjuge/companheiro ou a qualquer outro membro que resida com você, (iii) a qualquer pessoa jurídica em que qualquer destes seja controlador, sócio, acionista ou participante, de forma direta ou indireta, recebimento por palestras, aulas, atuação como proctor de treinamentos, remunerações, honorários pagos por participações em conselhos consultivos, de investigadores, ou outros comitês, etc. Provenientes da indústria farmacêutica, de órteses, próteses, equipamentos e implantes, brasileiras ou estrangeiras: - Bayer: Anticoagulação e insuficiência cardíaca; Pfizer: Anticoagulação e amiloidose; Jannsen: Leucemia. Outros relacionamentos Financiamento de atividades de educação médica continuada, incluindo viagens, hospedagens e inscrições para congressos e cursos, provenientes da indústria farmacêutica, de órteses, próteses, equipamentos e implantes, brasileiras ou estrangeiras: - Bayer: Insuficiência cardíaca.
Bruno Caramelli	Nada a ser declarado
Bruno Pereira Valdigem	Outros relacionamentos Financiamento de atividades de educação médica continuada, incluindo viagens, hospedagens e inscrições para congressos e cursos, provenientes da indústria farmacêutica, de órteses, próteses, equipamentos e implantes, brasileiras ou estrangeiras: - BMS: mavacamten; ABBOTT, Boston e Medtronic: materiais para Ablação por cateter.
Carlos Alberto Pastore	Nada a ser declarado
Carlos Eduardo Rochitte	Nada a ser declarado
Charles Mady	Nada a ser declarado
Claudio Tinoco Mesquita	Declaração financeira B - Financiamento de pesquisas sob sua responsabilidade direta/pessoal (direcionado ao departamento ou instituição) provenientes da indústria farmacêutica, de órteses, próteses, equipamentos e implantes, brasileiras ou estrangeiras: - Patisiran/Alnylam: amiloidose. Outros relacionamentos Financiamento de atividades de educação médica continuada, incluindo viagens, hospedagens e inscrições para congressos e cursos, provenientes da indústria farmacêutica, de órteses, próteses, equipamentos e implantes, brasileiras ou estrangeiras: - Tafamidis/Pfizer: amiloidose.
Daniela Calderaro	Declaração financeira A - Pagamento de qualquer espécie e desde que economicamente apreciáveis, feitos a (i) você, (ii) ao seu cônjuge/ companheiro ou a qualquer outro membro que resida com você, (iii) a qualquer pessoa jurídica em que qualquer destes seja controlador, sócio, acionista ou participante, de forma direta ou indireta, recebimento por palestras, aulas, atuação como proctor de treinamentos, remunerações, honorários pagos por participações em conselhos consultivos, de investigadores, ou outros comitês, etc. Provenientes da indústria farmacêutica, de órteses, próteses, equipamentos e implantes, brasileiras ou estrangeiras: - Bayer: fibrilação atrial, TEV, nefropatia diabética. Outros relacionamentos Financiamento de atividades de educação médica continuada, incluindo viagens, hospedagens e inscrições para congressos e cursos, provenientes da indústria farmacêutica, de órteses, próteses, equipamentos e implantes, brasileiras ou estrangeiras: - Bayer: nefropatia diabética.
Denilson Campos Albuquerque	Outros relacionamentos Financiamento de atividades de educação médica continuada, incluindo viagens, hospedagens e inscrições para congressos e cursos, provenientes da indústria farmacêutica, de órteses, próteses, equipamentos e implantes, brasileiras ou estrangeiras: - NovoNordisk: Semaglutida.
Edileide de Barros Correia	Declaração financeira A - Pagamento de qualquer espécie e desde que economicamente apreciáveis, feitos a (i) você, (ii) ao seu cônjuge/ companheiro ou a qualquer outro membro que resida com você, (iii) a qualquer pessoa jurídica em que qualquer destes seja controlador, sócio, acionista ou participante, de forma direta ou indireta, recebimento por palestras, aulas, atuação como proctor de treinamentos, remunerações, honorários pagos por participações em conselhos consultivos, de investigadores, ou outros comitês, etc. Provenientes da indústria farmacêutica, de órteses, próteses, equipamentos e implantes, brasileiras ou estrangeiras: - Pfizer: amiloidose; Bristol: mavacanteno; Sanofi: fabrazyme; Takeda: replagal. Outros relacionamentos Financiamento de atividades de educação médica continuada, incluindo viagens, hospedagens e inscrições para congressos e cursos, provenientes da indústria farmacêutica, de órteses, próteses, equipamentos e implantes, brasileiras ou estrangeiras: - Pfizer, AstraZeneca: amiloidose.
Edimar Alcides Bocchi	Declaração financeira A - Pagamento de qualquer espécie e desde que economicamente apreciáveis, feitos a (i) você, (ii) ao seu cônjuge/ companheiro ou a qualquer outro membro que resida com você, (iii) a qualquer pessoa jurídica em que qualquer destes seja controlador, sócio, acionista ou participante, de forma direta ou indireta, recebimento por palestras, aulas, atuação como proctor de treinamentos, remunerações, honorários pagos por participações em conselhos consultivos, de investigadores, ou outros comitês, etc. Provenientes da indústria farmacêutica, de órteses, próteses, equipamentos e implantes, brasileiras ou estrangeiras: - Servier, AstraZeneca, Merck: insuficiência cardíaca. B - Financiamento de pesquisas sob sua responsabilidade direta/pessoal (direcionado ao departamento ou instituição) provenientes da indústria farmacêutica, de órteses, próteses, equipamentos e implantes, brasileiras ou estrangeiras: - Merck: insuficiência cardíaca. Outros relacionamentos Financiamento de atividades de educação médica continuada, incluindo viagens, hospedagens e inscrições para congressos e cursos, provenientes da indústria farmacêutica, de órteses, próteses, equipamentos e implantes, brasileiras ou estrangeiras: - Servier: insuficiência cardíaca.
Edmundo Arteaga-Fernández	Nada a ser declarado
Eduardo Benchimol Saad	Declaração financeira A - Pagamento de qualquer espécie e desde que economicamente apreciáveis, feitos a (i) você, (ii) ao seu cônjuge/ companheiro ou a qualquer outro membro que resida com você, (iii) a qualquer pessoa jurídica em que qualquer destes seja controlador, sócio, acionista ou participante, de forma direta ou indireta, recebimento por palestras, aulas, atuação como proctor de treinamentos, remunerações, honorários pagos por participações em conselhos consultivos, de investigadores, ou outros comitês, etc. Provenientes da indústria farmacêutica, de órteses, próteses, equipamentos e implantes, brasileiras ou estrangeiras: - J&J, Abbott: eletrofisiologia.
Evandro Tinoco Mesquita	Declaração financeira A - Pagamento de qualquer espécie e desde que economicamente apreciáveis, feitos a (i) você, (ii) ao seu cônjuge/ companheiro ou a qualquer outro membro que resida com você, (iii) a qualquer pessoa jurídica em que qualquer destes seja controlador, sócio, acionista ou participante, de forma direta ou indireta, recebimento por palestras, aulas, atuação como proctor de treinamentos, remunerações, honorários pagos por participações em conselhos consultivos, de investigadores, ou outros comitês, etc. Provenientes da indústria farmacêutica, de órteses, próteses, equipamentos e implantes, brasileiras ou estrangeiras: - Ache: material educacional e aulas Astra. Outros relacionamentos Financiamento de atividades de educação médica continuada, incluindo viagens, hospedagens e inscrições para congressos e cursos, provenientes da indústria farmacêutica, de órteses, próteses, equipamentos e implantes, brasileiras ou estrangeiras: - Pfizer: amiloidose.
Fabiana Goulart Marcondes-Braga	Declaração financeira A - Pagamento de qualquer espécie e desde que economicamente apreciáveis, feitos a (i) você, (ii) ao seu cônjuge/ companheiro ou a qualquer outro membro que resida com você, (iii) a qualquer pessoa jurídica em que qualquer destes seja controlador, sócio, acionista ou participante, de forma direta ou indireta, recebimento por palestras, aulas, atuação como proctor de treinamentos, remunerações, honorários pagos por participações em conselhos consultivos, de investigadores, ou outros comitês, etc. Provenientes da indústria farmacêutica, de órteses, próteses, equipamentos e implantes, brasileiras ou estrangeiras: - Palestras e Comitê Consultivo: AstraZeneca; Boehringher-Lilly; Novartis; Bayer. B - Financiamento de pesquisas sob sua responsabilidade direta/pessoal (direcionado ao departamento ou instituição) provenientes da indústria farmacêutica, de órteses, próteses, equipamentos e implantes, brasileiras ou estrangeiras: - MSD: Victor Trial.
Fabio Biscegli Jatene	Declaração financeira A - Pagamento de qualquer espécie e desde que economicamente apreciáveis, feitos a (i) você, (ii) ao seu cônjuge/ companheiro ou a qualquer outro membro que resida com você, (iii) a qualquer pessoa jurídica em que qualquer destes seja controlador, sócio, acionista ou participante, de forma direta ou indireta, recebimento por palestras, aulas, atuação como proctor de treinamentos, remunerações, honorários pagos por participações em conselhos consultivos, de investigadores, ou outros comitês, etc. Provenientes da indústria farmacêutica, de órteses, próteses, equipamentos e implantes, brasileiras ou estrangeiras: - Edwards: próteses; remuneração por palestras em eventos científicos. B - Financiamento de pesquisas sob sua responsabilidade direta/pessoal (direcionado ao departamento ou instituição) provenientes da indústria farmacêutica, de órteses, próteses, equipamentos e implantes, brasileiras ou estrangeiras: - Edwards: próteses valvares; Samsung: wearables.
Fabio Fernandes	Declaração financeira A - Pagamento de qualquer espécie e desde que economicamente apreciáveis, feitos a (i) você, (ii) ao seu cônjuge/ companheiro ou a qualquer outro membro que resida com você, (iii) a qualquer pessoa jurídica em que qualquer destes seja controlador, sócio, acionista ou participante, de forma direta ou indireta, recebimento por palestras, aulas, atuação como proctor de treinamentos, remunerações, honorários pagos por participações em conselhos consultivos, de investigadores, ou outros comitês, etc. Provenientes da indústria farmacêutica, de órteses, próteses, equipamentos e implantes, brasileiras ou estrangeiras: - Pfizer: Tafamidis, Alnylam, Patisiran; Bristol: Mavacanteno. B - Financiamento de pesquisas sob sua responsabilidade direta/pessoal (direcionado ao departamento ou instituição) provenientes da indústria farmacêutica, de órteses, próteses, equipamentos e implantes, brasileiras ou estrangeiras: - Registro Estado de São Paulo em amiloidose, Pfizer, sem relação com medicação. Outros relacionamentos Financiamento de atividades de educação médica continuada, incluindo viagens, hospedagens e inscrições para congressos e cursos, provenientes da indústria farmacêutica, de órteses, próteses, equipamentos e implantes, brasileiras ou estrangeiras: - Pfizer: congresso do ISA 2024.
Fabio Sandoli de Brito Junior	Declaração financeira A - Pagamento de qualquer espécie e desde que economicamente apreciáveis, feitos a (i) você, (ii) ao seu cônjuge/ companheiro ou a qualquer outro membro que resida com você, (iii) a qualquer pessoa jurídica em que qualquer destes seja controlador, sócio, acionista ou participante, de forma direta ou indireta, recebimento por palestras, aulas, atuação como proctor de treinamentos, remunerações, honorários pagos por participações em conselhos consultivos, de investigadores, ou outros comitês, etc. Provenientes da indústria farmacêutica, de órteses, próteses, equipamentos e implantes, brasileiras ou estrangeiras: - Proctor Edwards, Medtronic, Boston: TAVI.
Felix José Alvarez Ramires	Declaração financeira A - Pagamento de qualquer espécie e desde que economicamente apreciáveis, feitos a (i) você, (ii) ao seu cônjuge/companheiro ou a qualquer outro membro que resida com você, (iii) a qualquer pessoa jurídica em que qualquer destes seja controlador, sócio, acionista ou participante, de forma direta ou indireta, recebimento por palestras, aulas, atuação como proctor de treinamentos, remunerações, honorários pagos por participações em conselhos consultivos, de investigadores, ou outros comitês, etc. Provenientes da indústria farmacêutica, de órteses, próteses, equipamentos e implantes, brasileiras ou estrangeiras: - Novartis: Sacubitril/Valsartana; Pfizer: Patisiran; Merck: Vericiquat; Amgen.
Fernando Bacal	Outros relacionamentos Financiamento de atividades de educação médica continuada, incluindo viagens, hospedagens e inscrições para congressos e cursos, provenientes da indústria farmacêutica, de órteses, próteses, equipamentos e implantes, brasileiras ou estrangeiras: - Novartis: palestra em congresso.
Fernando Luis Scolari	Declaração financeira A - Pagamento de qualquer espécie e desde que economicamente apreciáveis, feitos a (i) você, (ii) ao seu cônjuge/ companheiro ou a qualquer outro membro que resida com você, (iii) a qualquer pessoa jurídica em que qualquer destes seja controlador, sócio, acionista ou participante, de forma direta ou indireta, recebimento por palestras, aulas, atuação como proctor de treinamentos, remunerações, honorários pagos por participações em conselhos consultivos, de investigadores, ou outros comitês, etc. Provenientes da indústria farmacêutica, de órteses, próteses, equipamentos e implantes, brasileiras ou estrangeiras: - Bristol Myers Squibb. Outros relacionamentos Financiamento de atividades de educação médica continuada, incluindo viagens, hospedagens e inscrições para congressos e cursos, provenientes da indústria farmacêutica, de órteses, próteses, equipamentos e implantes, brasileiras ou estrangeiras: - Bristol Myers Squibb.
Flávio de Souza Brito	Declaração financeira A - Pagamento de qualquer espécie e desde que economicamente apreciáveis, feitos a (i) você, (ii) ao seu cônjuge/ companheiro ou a qualquer outro membro que resida com você, (iii) a qualquer pessoa jurídica em que qualquer destes seja controlador, sócio, acionista ou participante, de forma direta ou indireta, recebimento por palestras, aulas, atuação como proctor de treinamentos, remunerações, honorários pagos por participações em conselhos consultivos, de investigadores, ou outros comitês, etc. Provenientes da indústria farmacêutica, de órteses, próteses, equipamentos e implantes, brasileiras ou estrangeiras: - AstraZeneca: Forxiga; Novartis: Entresto; Servier: Procoralan; Boehringer Ingelheim: Jardiance; Bayer: Xarelto; Merck: Concor; Daichii Sankyo: Lixiana; Viatris: Inspra; EMS: Bramicar; Biolab: Bivolet; Novonordisk: Ozempic. Outros relacionamentos Financiamento de atividades de educação médica continuada, incluindo viagens, hospedagens e inscrições para congressos e cursos, provenientes da indústria farmacêutica, de órteses, próteses, equipamentos e implantes, brasileiras ou estrangeiras: - Novonordisk: Ozempic.
Gustavo Calado de Aguiar Ribeiro	Nada a ser declarado
Henrique Barbosa Ribeiro	Declaração financeira A - Pagamento de qualquer espécie e desde que economicamente apreciáveis, feitos a (i) você, (ii) ao seu cônjuge/ companheiro ou a qualquer outro membro que resida com você, (iii) a qualquer pessoa jurídica em que qualquer destes seja controlador, sócio, acionista ou participante, de forma direta ou indireta, recebimento por palestras, aulas, atuação como proctor de treinamentos, remunerações, honorários pagos por participações em conselhos consultivos, de investigadores, ou outros comitês, etc. Provenientes da indústria farmacêutica, de órteses, próteses, equipamentos e implantes, brasileiras ou estrangeiras: - Medtronic, Boston Scientific, Edwards Lifesciences: TAVI aulas e proctorship. B - Financiamento de pesquisas sob sua responsabilidade direta/pessoal (direcionado ao departamento ou instituição) provenientes da indústria farmacêutica, de órteses, próteses, equipamentos e implantes, brasileiras ou estrangeiras: - Medtronic: estenose aórtica.
Humberto Villacorta Junior	Declaração financeira A - Pagamento de qualquer espécie e desde que economicamente apreciáveis, feitos a (i) você, (ii) ao seu cônjuge/ companheiro ou a qualquer outro membro que resida com você, (iii) a qualquer pessoa jurídica em que qualquer destes seja controlador, sócio, acionista ou participante, de forma direta ou indireta, recebimento por palestras, aulas, atuação como proctor de treinamentos, remunerações, honorários pagos por participações em conselhos consultivos, de investigadores, ou outros comitês, etc. Provenientes da indústria farmacêutica, de órteses, próteses, equipamentos e implantes, brasileiras ou estrangeiras: - AstraZeneca: Forxiga e Lokelma; Novartis: Entresto; Pfizer: amiloidose; Roche Diagnostics: NT-proBNP. B - Financiamento de pesquisas sob sua responsabilidade direta/pessoal (direcionado ao departamento ou instituição) provenientes da indústria farmacêutica, de órteses, próteses, equipamentos e implantes, brasileiras ou estrangeiras: - Roche: kits de GDF-15.
Ibraim Masciarelli Francisco Pinto	Nada a ser declarado
Ieda Biscegli Jatene	Declaração financeira A - Pagamento de qualquer espécie e desde que economicamente apreciáveis, feitos a (i) você, (ii) ao seu cônjuge/ companheiro ou a qualquer outro membro que resida com você, (iii) a qualquer pessoa jurídica em que qualquer destes seja controlador, sócio, acionista ou participante, de forma direta ou indireta, recebimento por palestras, aulas, atuação como proctor de treinamentos, remunerações, honorários pagos por participações em conselhos consultivos, de investigadores, ou outros comitês, etc. Provenientes da indústria farmacêutica, de órteses, próteses, equipamentos e implantes, brasileiras ou estrangeiras: - AstraZeneca: Palivizumabe. Outros relacionamentos Participação em órgãos governamentais de regulação, ou de defesa de direitos na área de cardiologia: - Conselho Regional de Medicina do Estado de São Paulo.
Josá albuquerque de Figueiredo Neto	Declaração financeira A - Pagamento de qualquer espécie e desde que economicamente apreciáveis, feitos a (i) você, (ii) ao seu cônjuge/ companheiro ou a qualquer outro membro que resida com você, (iii) a qualquer pessoa jurídica em que qualquer destes seja controlador, sócio, acionista ou participante, de forma direta ou indireta, recebimento por palestras, aulas, atuação como proctor de treinamentos, remunerações, honorários pagos por participações em conselhos consultivos, de investigadores, ou outros comitês, etc. Provenientes da indústria farmacêutica, de órteses, próteses, equipamentos e implantes, brasileiras ou estrangeiras: - Novartis; AstraZeneca; Boehringer; Servier. B - Financiamento de pesquisas sob sua responsabilidade direta/pessoal (direcionado ao departamento ou instituição) provenientes da indústria farmacêutica, de órteses, próteses, equipamentos e implantes, brasileiras ou estrangeiras: - Novartis. Outros relacionamentos Financiamento de atividades de educação médica continuada, incluindo viagens, hospedagens e inscrições para congressos e cursos, provenientes da indústria farmacêutica, de órteses, próteses, equipamentos e implantes, brasileiras ou estrangeiras: - Novartis; AstraZeneca.
José Eduardo Krieger	Nada a ser declarado
Juliano Novaes Cardoso	Nada a ser declarado
Lidia Zytynski Moura	Declaração financeira A - Pagamento de qualquer espécie e desde que economicamente apreciáveis, feitos a (i) você, (ii) ao seu cônjuge/ companheiro ou a qualquer outro membro que resida com você, (iii) a qualquer pessoa jurídica em que qualquer destes seja controlador, sócio, acionista ou participante, de forma direta ou indireta, recebimento por palestras, aulas, atuação como proctor de treinamentos, remunerações, honorários pagos por participações em conselhos consultivos, de investigadores, ou outros comitês, etc. Provenientes da indústria farmacêutica, de órteses, próteses, equipamentos e implantes, brasileiras ou estrangeiras: - Novartis: Entresto; AstraZeneca: Forxiga; Boehringer: Jardiance; Bayer: Vericiguat; Vifor: Ferrinject. B - Financiamento de pesquisas sob sua responsabilidade direta/pessoal (direcionado ao departamento ou instituição) provenientes da indústria farmacêutica, de órteses, próteses, equipamentos e implantes, brasileiras ou estrangeiras. - Bayer: Vericiguat.
Ludhmila Abrahão Hajjar	Nada a ser declarado
Luis Beck-da-Silva	Declaração financeira A - Pagamento de qualquer espécie e desde que economicamente apreciáveis, feitos a (i) você, (ii) ao seu cônjuge/ companheiro ou a qualquer outro membro que resida com você, (iii) a qualquer pessoa jurídica em que qualquer destes seja controlador, sócio, acionista ou participante, de forma direta ou indireta, recebimento por palestras, aulas, atuação como proctor de treinamentos, remunerações, honorários pagos por participações em conselhos consultivos, de investigadores, ou outros comitês, etc. Provenientes da indústria farmacêutica, de órteses, próteses, equipamentos e implantes, brasileiras ou estrangeiras: - Viatris; Pfizer; AstraZeneca; NovoNordisk. B - Financiamento de pesquisas sob sua responsabilidade direta/pessoal (direcionado ao departamento ou instituição) provenientes da indústria farmacêutica, de órteses, próteses, equipamentos e implantes, brasileiras ou estrangeiras: - CSL Vifor. Outros relacionamentos Financiamento de atividades de educação médica continuada, incluindo viagens, hospedagens e inscrições para congressos e cursos, provenientes da indústria farmacêutica, de órteses, próteses, equipamentos e implantes, brasileiras ou estrangeiras: - NovoNordisk; Viatris.
Luis Eduardo Paim Rohde	Declaração financeira A - Pagamento de qualquer espécie e desde que economicamente apreciáveis, feitos a (i) você, (ii) ao seu cônjuge/ companheiro ou a qualquer outro membro que resida com você, (iii) a qualquer pessoa jurídica em que qualquer destes seja controlador, sócio, acionista ou participante, de forma direta ou indireta, recebimento por palestras, aulas, atuação como proctor de treinamentos, remunerações, honorários pagos por participações em conselhos consultivos, de investigadores, ou outros comitês, etc. Provenientes da indústria farmacêutica, de órteses, próteses, equipamentos e implantes, brasileiras ou estrangeiras: - AstraZeneca: Dapagliflozina e Ciclossilicato de Zircônio Sódico; Novartis: Sacubitril-Valsartana; Novonordisk: Semaglutida; Aché: Dapagliflozina; Merck Serono: Bisoprolol. Outros relacionamentos Financiamento de atividades de educação médica continuada, incluindo viagens, hospedagens e inscrições para congressos e cursos, provenientes da indústria farmacêutica, de órteses, próteses, equipamentos e implantes, brasileiras ou estrangeiras: - Novonordisk: Semaglutida; Viatris: Eplerenone.
Luiz Claudio Danzmann	Declaração financeira A - Pagamento de qualquer espécie e desde que economicamente apreciáveis, feitos a (i) você, (ii) ao seu cônjuge/ companheiro ou a qualquer outro membro que resida com você, (iii) a qualquer pessoa jurídica em que qualquer destes seja controlador, sócio, acionista ou participante, de forma direta ou indireta, recebimento por palestras, aulas, atuação como proctor de treinamentos, remunerações, honorários pagos por participações em conselhos consultivos, de investigadores, ou outros comitês, etc. Provenientes da indústria farmacêutica, de órteses, próteses, equipamentos e implantes, brasileiras ou estrangeiras: - Novartis: Entresto; AstraZeneca: Forxiga; Servier: Procoralan.
Manoel Fernandes Canesin	Nada a ser declarado
Manuel Nicolas Cano	Nada a ser declarado
Marcelo Dantas Tavares de Melo	Nada a ser declarado
Marcelo Imbroinise Bittencourt	Declaração financeira A - Pagamento de qualquer espécie e desde que economicamente apreciáveis, feitos a (i) você, (ii) ao seu cônjuge/ companheiro ou a qualquer outro membro que resida com você, (iii) a qualquer pessoa jurídica em que qualquer destes seja controlador, sócio, acionista ou participante, de forma direta ou indireta, recebimento por palestras, aulas, atuação como proctor de treinamentos, remunerações, honorários pagos por participações em conselhos consultivos, de investigadores, ou outros comitês, etc. Provenientes da indústria farmacêutica, de órteses, próteses, equipamentos e implantes, brasileiras ou estrangeiras: - Pfizer: amiloidose; Bristol: cardiomiopatia hipertrófica; Sanofi: Doença de Fabry.
Marcelo Luiz Campos Vieira	Nada a ser declarado
Marcelo Westerlund Montera	Nada a ser declarado
Marcus V. Simões	Nada a ser declarado
Martino Martinelli Filho	Nada a ser declarado
Mauricio Ibrahim Scanavacca	Declaração financeira A - Pagamento de qualquer espécie e desde que economicamente apreciáveis, feitos a (i) você, (ii) ao seu cônjuge/ companheiro ou a qualquer outro membro que resida com você, (iii) a qualquer pessoa jurídica em que qualquer destes seja controlador, sócio, acionista ou participante, de forma direta ou indireta, recebimento por palestras, aulas, atuação como proctor de treinamentos, remunerações, honorários pagos por participações em conselhos consultivos, de investigadores, ou outros comitês, etc. Provenientes da indústria farmacêutica, de órteses, próteses, equipamentos e implantes, brasileiras ou estrangeiras: - Daichii Sankyo: anticoagulação. B - Financiamento de pesquisas sob sua responsabilidade direta/pessoal (direcionado ao departamento ou instituição) provenientes da indústria farmacêutica, de órteses, próteses, equipamentos e implantes, brasileiras ou estrangeiras: - J&J: ablação de taquicardia ventricular. C - Financiamento de pesquisa (pessoal), cujas receitas tenham sido provenientes da indústria farmacêutica, de órteses, próteses, equipamentos e implantes, brasileiras ou estrangeiras: - ABBOT: ablação por cateter da cindam vaso vagal. Outros relacionamentos Financiamento de atividades de educação médica continuada, incluindo viagens, hospedagens e inscrições para congressos e cursos, provenientes da indústria farmacêutica, de órteses, próteses, equipamentos e implantes, brasileiras ou estrangeiras: - J&J: simpósio patrocinado pela indústria.
Minna Moreira Dias Romano	Nada a ser declarado
Moysés de Oliveira Lima Filho	Outros relacionamentos Vínculo empregatício com a indústria farmacêutica, de órteses, próteses, equipamentos e implantes, brasileiras ou estrangeiras, assim como se tem relação vínculo empregatício com operadoras de planos de saúde ou em auditorias médicas (incluindo meio período) durante o ano para o qual você está declarando: - Membro da Diretoria Executiva da Unimed de Ribeirão Preto.
Mucio Tavares de Oliveira Junior	Declaração financeira A - Pagamento de qualquer espécie e desde que economicamente apreciáveis, feitos a (i) você, (ii) ao seu cônjuge/ companheiro ou a qualquer outro membro que resida com você, (iii) a qualquer pessoa jurídica em que qualquer destes seja controlador, sócio, acionista ou participante, de forma direta ou indireta, recebimento por palestras, aulas, atuação como proctor de treinamentos, remunerações, honorários pagos por participações em conselhos consultivos, de investigadores, ou outros comitês, etc. Provenientes da indústria farmacêutica, de órteses, próteses, equipamentos e implantes, brasileiras ou estrangeiras: - Sanofi Pasteur: vacinas; Viatris: Inspra; AstraZeneca: Forxiga. B - Financiamento de pesquisas sob sua responsabilidade direta/pessoal (direcionado ao departamento ou instituição) provenientes da indústria farmacêutica, de órteses, próteses, equipamentos e implantes, brasileiras ou estrangeiras: - Sanofi Pasteur: influenza.
Murillo de Oliveira Antunes	Declaração financeira A - Pagamento de qualquer espécie e desde que economicamente apreciáveis, feitos a (i) você, (ii) ao seu cônjuge/ companheiro ou a qualquer outro membro que resida com você, (iii) a qualquer pessoa jurídica em que qualquer destes seja controlador, sócio, acionista ou participante, de forma direta ou indireta, recebimento por palestras, aulas, atuação como proctor de treinamentos, remunerações, honorários pagos por participações em conselhos consultivos, de investigadores, ou outros comitês, etc. Provenientes da indústria farmacêutica, de órteses, próteses, equipamentos e implantes, brasileiras ou estrangeiras: - Takeda; Bayer; Pfizer; Sanofi. B - Financiamento de pesquisas sob sua responsabilidade direta/pessoal (direcionado ao departamento ou instituição) provenientes da indústria farmacêutica, de órteses, próteses, equipamentos e implantes, brasileiras ou estrangeiras: - Bristol: Mavacamten. Outros relacionamentos Financiamento de atividades de educação médica continuada, incluindo viagens, hospedagens e inscrições para congressos e cursos, provenientes da indústria farmacêutica, de órteses, próteses, equipamentos e implantes, brasileiras ou estrangeiras: - Biolab; Takeda; Sanofi; Alnaylam.
Otavio Rizzi Coelho-Filho	Declaração financeira A - Pagamento de qualquer espécie e desde que economicamente apreciáveis, feitos a (i) você, (ii) ao seu cônjuge/ companheiro ou a qualquer outro membro que resida com você, (iii) a qualquer pessoa jurídica em que qualquer destes seja controlador, sócio, acionista ou participante, de forma direta ou indireta, recebimento por palestras, aulas, atuação como proctor de treinamentos, remunerações, honorários pagos por participações em conselhos consultivos, de investigadores, ou outros comitês, etc. Provenientes da indústria farmacêutica, de órteses, próteses, equipamentos e implantes, brasileiras ou estrangeiras: - Pfizer: amiloidose/Tafamides; AstraZeneca: insuficiência cardíaca/Forxiga; Bayer: Firialta; Norvartis: insuficiência cardíaca/dislipidemia; EMS: insuficiência cardíaca. Outros relacionamentos Financiamento de atividades de educação médica continuada, incluindo viagens, hospedagens e inscrições para congressos e cursos, provenientes da indústria farmacêutica, de órteses, próteses, equipamentos e implantes, brasileiras ou estrangeiras: - Bayer; Pfizer; AstraZeneca.
Pedro Alves Lemos Neto	Nada a ser declarado
Pedro Silvio Farsky	Declaração financeira A - Pagamento de qualquer espécie e desde que economicamente apreciáveis, feitos a (i) você, (ii) ao seu cônjuge/ companheiro ou a qualquer outro membro que resida com você, (iii) a qualquer pessoa jurídica em que qualquer destes seja controlador, sócio, acionista ou participante, de forma direta ou indireta, recebimento por palestras, aulas, atuação como proctor de treinamentos, remunerações, honorários pagos por participações em conselhos consultivos, de investigadores, ou outros comitês, etc. Provenientes da indústria farmacêutica, de órteses, próteses, equipamentos e implantes, brasileiras ou estrangeiras: - Lilly: empagliflozina. Outros relacionamentos Financiamento de atividades de educação médica continuada, incluindo viagens, hospedagens e inscrições para congressos e cursos, provenientes da indústria farmacêutica, de órteses, próteses, equipamentos e implantes, brasileiras ou estrangeiras: - Novo Nordisk: semaglutida.
Pedro Vellosa Schwartzmann	Declaração financeira B - Financiamento de pesquisas sob sua responsabilidade direta/pessoal (direcionado ao departamento ou instituição) provenientes da indústria farmacêutica, de órteses, próteses, equipamentos e implantes, brasileiras ou estrangeiras: - MSD; Novartis; AstraZeneca; Alnylam; BridgeBio; Ionis; Lilly. Outros relacionamentos Financiamento de atividades de educação médica continuada, incluindo viagens, hospedagens e inscrições para congressos e cursos, provenientes da indústria farmacêutica, de órteses, próteses, equipamentos e implantes, brasileiras ou estrangeiras: - Pfizer; AstraZeneca; Novartis; Alnylam; NovoNordisk.
Renato Delascio Lopes	Nada a ser declarado
Ricardo Mourilhe-Rocha	Declaração financeira A - Pagamento de qualquer espécie e desde que economicamente apreciáveis, feitos a (i) você, (ii) ao seu cônjuge/ companheiro ou a qualquer outro membro que resida com você, (iii) a qualquer pessoa jurídica em que qualquer destes seja controlador, sócio, acionista ou participante, de forma direta ou indireta, recebimento por palestras, aulas, atuação como proctor de treinamentos, remunerações, honorários pagos por participações em conselhos consultivos, de investigadores, ou outros comitês, etc. Provenientes da indústria farmacêutica, de órteses, próteses, equipamentos e implantes, brasileiras ou estrangeiras: - AstraZeneca: insuficiência cardíaca e discalemias;/ Bayer: insuficiência renal; Boehringer-Lilly: insuficiência cardíaca; Novartis: insuficiência cardíaca; NovoNordisk: insuficiência cardíaca; Servier: insuficiência cardíaca; Daiichi-Sankyo: fibrilação atrial. B - Financiamento de pesquisas sob sua responsabilidade direta/pessoal (direcionado ao departamento ou instituição) provenientes da indústria farmacêutica, de órteses, próteses, equipamentos e implantes, brasileiras ou estrangeiras: - BMS: mavacanteno); Boehringer: empagliflozina; Novartis: sacubitril/valsartan.
Ricardo Ribeiro Dias	Nada a ser declarado
Silvia Marinho Martins Alves	Outros relacionamentos Financiamento de atividades de educação médica continuada, incluindo viagens, hospedagens e inscrições para congressos e cursos, provenientes da indústria farmacêutica, de órteses, próteses, equipamentos e implantes, brasileiras ou estrangeiras: - Novartis: Entresto.
Silvia Moreira Ayub Ferreira	Declaração financeira A - Pagamento de qualquer espécie e desde que economicamente apreciáveis, feitos a (i) você, (ii) ao seu cônjuge/ companheiro ou a qualquer outro membro que resida com você, (iii) a qualquer pessoa jurídica em que qualquer destes seja controlador, sócio, acionista ou participante, de forma direta ou indireta, recebimento por palestras, aulas, atuação como proctor de treinamentos, remunerações, honorários pagos por participações em conselhos consultivos, de investigadores, ou outros comitês, etc. Provenientes da indústria farmacêutica, de órteses, próteses, equipamentos e implantes, brasileiras ou estrangeiras: - Abbott, CSL Vifor.
Sivio Henrique Barberato	Outros relacionamentos Financiamento de atividades de educação médica continuada, incluindo viagens, hospedagens e inscrições para congressos e cursos, provenientes da indústria farmacêutica, de órteses, próteses, equipamentos e implantes, brasileiras ou estrangeiras: - Bristol: Camzyos.
Stephanie Itala Rizk	Nada a ser declarado
Vagner Madrini Junior	Nada a ser declarado
Viviane Tiemi Hotta	Nada a ser declarado
Walkiria Samuel Avila	Nada a ser declarado
Wilson Mathias Junior	Nada a ser declarado
Wolney de Andrade Martins	Nada a ser declarado

## 1. Introdução

O conhecimento médico científico sobre a cardiomiopatia hipertrófica (CMH) recebeu grande avanço nas últimas décadas. O melhor entendimento da sua patogênese, os avanços expressivos no emprego dos métodos de imagem e a aplicação mais corriqueira da análise genética, além da melhor caracterização da história natural dessa doença miocárdica, trouxeram uma profunda reformulação do seu significado clínico e prognóstico. Por outro lado, esses progressos foram acompanhados do desenvolvimento de novas drogas endereçando mecanismos moleculares intrinsecamente ligados à fisiopatogênese da doença e suas manifestações, representando uma enorme conquista da ciência e um marco na história da cardiologia e das doenças miocárdicas.

Assim, frente a tantos novos conhecimentos, cuja incorporação à prática clínica se faz necessária e premente, a presente diretriz tem por objetivo apresentar as recomendações mais atuais para o diagnóstico, estadiamento prognóstico e tratamento da CMH, com base na revisão crítica das evidências científicas atuais.

Neste posicionamento, as tabelas de classes de recomendação e níveis de evidência foram construídas conforme a padronização a seguir ( Tabela 1 ).

**Tabela 1:** Padronização das classes de recomendação e níveis de evidência empregados nesta diretriz

Classes (graus) de recomendação:
**Classe I** – condições para as quais há evidências conclusivas ou, em sua ausência, consenso geral de que o procedimento é seguro e útil/eficaz.
**Classe IIa** – condições para as quais há evidências conflitantes e/ou divergência de opinião sobre segurança e utilidade/eficácia do procedimento; peso ou evidência/opinião a favor do procedimento. A maioria dos estudos/especialistas aprova.
**Classe IIb** – condições para as quais há evidências conflitantes e/ou divergência de opinião sobre segurança e utilidade/eficácia do procedimento. Segurança e utilidade/eficácia menos bem estabelecida, não havendo predomínio de opiniões a favor.
**Classe III** – condições para as quais há evidências e/ou consenso de que o procedimento não é útil/eficaz e, em alguns casos, pode ser prejudicial.
**Níveis de evidência:**
**Nível A** – dados obtidos a partir de múltiplos estudos randomizados de bom porte, concordantes e/ou de metanálise robusta de estudos clínicos randomizados.
**Nível B** – dados obtidos a partir de metanálise menos robusta, a partir de um único estudo randomizado ou de estudos não randomizados (observacionais).
**Nível C** – dados obtidos de opiniões consensuais de especialistas.

### 1.1. Definição e História Natural da Cardiomiopatia Hipertrófica

A CMH é definida pela presença de hipertrofia do miocárdio, que determina o aumento das espessuras das paredes, sem dilatação ventricular nas fases iniciais, que ocorre na ausência de outras doenças cardíacas, sistêmicas, metabólicas ou sindrômicas que justifiquem o aparecimento dessa alteração fenotípica.^1^

O diagnóstico da CMH é estabelecido por exames de imagem, ecocardiografia 2D ou ressonância magnética cardiovascular (RMC), e é definida em adultos a presença de uma espessura diastólica final em quaisquer segmentos dos ventrículos de ≥ 15 mm ou ≥ 13 mm em familiares de um paciente com CMH ou em conjunto com um teste genético positivo.^2 , 3^

Na pediatria, é essencial que os valores obtidos nos exames de imagem sejam indexados para a superfície corporal. As associações médicas americanas American College of Cardiology (ACC)/American Heart Association (AHA) consideram valores superiores a *z-score* > 2,5 como hipertrofia patológica em crianças assintomáticas e com ausência de história familiar positiva, por outro lado, valores superiores a dois desvios padrão são suficientes para que seja feito um diagnóstico precoce.^2 , 4^ Em adultos, o *z-score* que corresponde à medida septal de 15 mm é de 6 a 7, enquanto o septo de 13 mm equivale a um *z-score* de 4 a 6.

A CMH é a mais prevalente das cardiopatias de origem genética,^2^ sendo transmitida de forma autossômica dominante e causada predominantemente por mutações em genes que codificam proteínas sarcoméricas.^5^ Ela é reconhecida por uma ampla diversidade de apresentações fenotípicas quanto aos sintomas, evolução clínica, prognóstico e penetrância da doença.^6^ A maioria dos indivíduos com CMH é assintomática e tem uma expectativa de vida normal, enquanto uma minoria sofre sintomas debilitantes e apresentam um risco aumentado de morte súbita cardíaca (MSC) prematura. A CMH é considerada uma das principais causas de MSC em jovens e atletas.^7 , 8^ Como a obstrução da via de saída do ventrículo esquerdo (VSVE) está presente ou se desenvolve ao longo do tempo na maioria dos pacientes com CMH, mas 1/3 permanece não obstrutivo, o comitê de redação recomenda o termo CMH (com ou sem obstrução da via de saída). Os sintomas podem estar relacionados a uma série de mecanismos fisiopatológicos, incluindo disfunção diastólica,^9^ insuficiência cardíaca (IC) com fração de ejeção (FE) preservada ou reduzida,^10 , 11^ obstrução da VSVE^12 , 13^ com ou sem insuficiência mitral significativa,^14^ disfunção autonômica,^15^ isquemia^16 , 17^ e arritmias cardíacas.^18 - 21^

**Figure:**
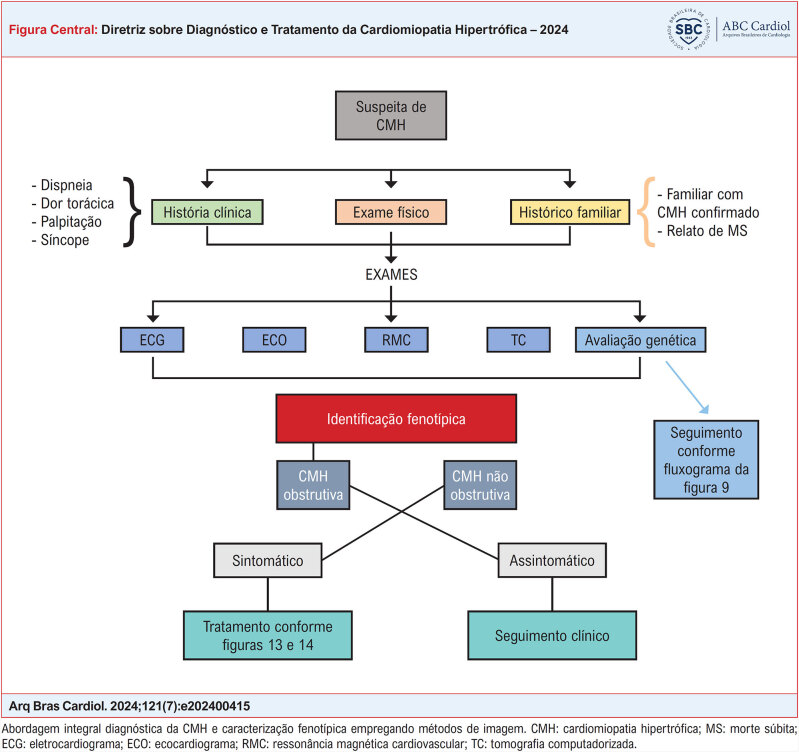


### 1.2. Epidemiologia

Atualmente, a CMH já foi reportada em 122 países diferentes, o que representa 90% da população mundial com prevalência de 1 caso por 500 pessoas na população geral, conforme os primeiros registros epidemiológicos baseados em ecocardiografia.^22 , 23^ No entanto, estudos mais recentes incluindo indivíduos portadores de genes considerados patogênicos (genótipo positivo e fenótipo negativo) encontraram uma prevalência de 1 caso por 200 pessoas,^24^ o que representa uma condição muito mais comum do que se pensava anteriormente, estimando-se que, no Brasil, cerca de 400 mil pessoas possam ser afetadas.^25^

### 1.3. História Natural

**Figura 1:**
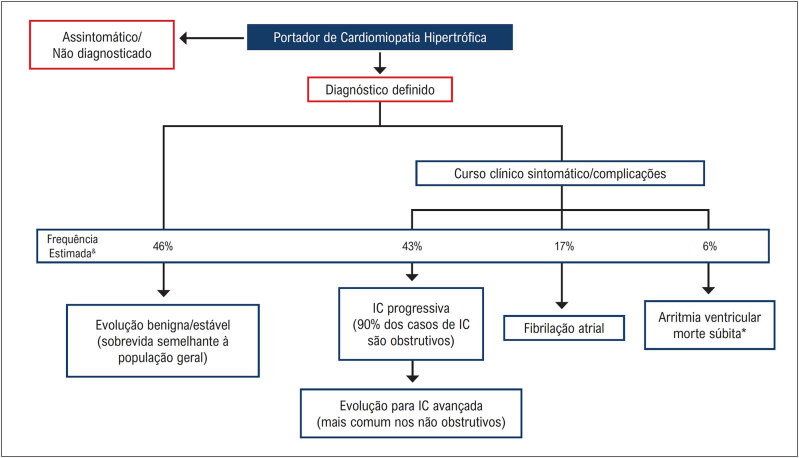
História natural da CMH, com a frequência estimada da evolução clínica benigna e das diferentes complicações. *Com adequada estratificação de risco de morte súbita e implante de CDI, a incidência de morte súbita pode ser reduzida para cerca de 1%. IC: insuficiência cardíaca. Adaptada a partir de Maron et al.^33^

A CMH ainda é uma doença heterogênea de curso imprevisível, com muitas particularidades e desafios. Ela pode se manifestar desde a infância até a oitava década de vida. Coortes mais recentes mostram que cerca de 46% dos pacientes podem apresentar uma evolução benigna, com uma expectativa de vida normal e sem limitações, e o restante dos pacientes pode evoluir com sintomas progressivos ou apresentar complicações clínicas e eventos adversos ao longo da vida ( Figura 1 ). Os principais eventos adversos que encontramos ao longo da evolução do paciente com CMH são: morte súbita, isquemia miocárdica, limitação funcional progressiva devido à obstrução da VSVE ou por disfunção diastólica, evolução para disfunção sistólica do ventrículo esquerdo (VE), fibrilação atrial (FA) com risco aumentado de eventos trombóticos e arritmias ventriculares^26 - 29^ ( Figura 1 ). Estudos sugerem que o risco desses eventos adversos ao longo da evolução pode ser maior entre pacientes diagnosticados no início da vida ou naqueles com variantes patogênicas do gene sarcomérico.^29^

A avaliação periódica do risco individual de MSC do paciente é muito importante e ainda é um desafio no manejo da CMH. Entre os principais fatores relacionados com a morte súbita encontramos: indivíduos mais jovens (< 30 anos); a história pessoal de MSC abortada; arritmias ventriculares sustentadas; espessura máxima da parede ventricular esquerda; síncope relacionada com arritmia; história familiar de MSC; taquicardia ventricular não sustentada (TVNS); FE do VE diminuída (< 50%); e fibrose miocárdica extensa identificada na RMC (> 15–20%).^30^ Nos pacientes jovens de alto risco, deve ser considerado o implante de um cardiodesfibrilador implantável (CDI).^31^

A IC na CMH é causada por diversos mecanismos fisiopatológicos, incluindo obstrução da VSVE, além da disfunção ventricular diastólica ou sistólica global. Um estudo revelou que, em um acompanhamento médio de 6 anos, 17% dos pacientes evoluíram para IC de classe funcional (NYHA) III/IV, enquanto 55% permaneceram em classe funcional I. A FA provou ser a variável de doença mais comum associada à IC progressiva nesse acompanhamento. Entre os pacientes com CMH, 30 a 40% apresentarão eventos adversos, incluindo: 1) eventos de morte súbita; 2) sintomas limitantes progressivos por causa da obstrução da VSVE ou disfunção diastólica; 3) sintomas de IC associados à disfunção sistólica; e 4) FA com risco de acidente vascular cerebral (AVC) tromboembólico. No entanto, estudos que fazem o acompanhamento desses pacientes em longo prazo demonstraram que, para pacientes em risco ou que desenvolvem uma dessas complicações relacionadas à doença, a aplicação de terapias e intervenções cardiovasculares contemporâneas reduziram as taxas de mortalidade para < 1,0%/ano.^24 , 32^

## 2. Quadro Clínico

### 2.1. Arritmias e Síncope

O paciente portador de CMH pode evoluir com arritmias cardíacas, que podem ser assintomáticas — detectadas em exame de rotina ou através de monitorização eletrocardiográfica de 24 horas por sistema *Holter* — ou sintomáticas. As arritmias mais encontradas são extrassístoles (supraventriculares ou ventriculares), fibrilação ou *flutter* atrial e taquicardia ventricular não sustentada (TVNS) ou sustentada (TVS). Pode ocorrer, ainda, taquicardia paroxística supraventricular, em casos de associação de CMH com síndromes de pré-excitação. Além disso, o risco de FA e de síncope parece estar aumentado quando essa associação ocorre.^1^ Em alguns casos, a morte súbita pode ser a primeira manifestação de um quadro de CMH, principalmente durante o exercício físico, sendo essa a principal causa de morte súbita em atletas.^34^

Os principais sintomas indicativos de possíveis arritmias cardíacas são palpitações, pré-síncope e síncope.

### 2.2. Dor Torácica

Os pacientes com CMH podem apresentar dor torácica desencadeada pelo exercício físico ou que também pode ocorrer no repouso. A isquemia miocárdica é mais comumente causada por alterações próprias da CMH, que cursa com um desbalanço entre a oferta e o consumo de oxigênio pelo miocardio.^35^ Alterações microvasculares, hipertrofia ventricular, aumento do estresse na parede e obstrução na VSVE são alterações que ocorrem na CMH e podem também ser responsáveis pela dor torácica. As anormalidades do fluxo coronariano decorrem também devido a efeitos resultantes da compressão e deformação dos vasos sanguíneos intramurais e do relaxamento do ventrículo alterado.^17 , 36^ Entretanto, nos casos de angina típica, é importante excluir a doença coronariana epicárdica, principalmente em populações de faixas etárias mais avançadas e com fatores de risco cardiovascular. Para essa finalidade, preconiza-se o uso da angiotomografia de coronárias ou cineangiocoronariografia, que podem revelar uma doença obstrutiva aterosclerótica associada ou também alterações das coronárias como ponte intramiocárdica, que também podem cursar com angina.^17 , 36 , 37^

### 2.3. Insuficiência Cardíaca

Portadores de CMH podem evoluir com grau muito variado de comprometimento estrutural e funcional cardíaco. A vasta maioria dos pacientes exibindo síndrome de IC, estimados em 90%, exibe obstrução dinâmica do trato de saída do VE em repouso ou esforço, indicando um papel central desse distúrbio no desenvolvimento da IC na CMH. A dispneia de esforço e a fadiga constituem as principais manifestações, enquanto a ortopneia e a dispneia paroxística são menos encontradas. A população mais jovem, com menor prevalência de comorbidades, amplo predomínio com FE preservada e menor mortalidade caracterizam o quadro. A IC avançada com manifestações de grave congestão sistêmica e/ou pulmonar é rara, tornando o quadro de manejo bem mais ambulatorial.^38^

Os portadores de IC com obstrução de VSVE, seja em repouso ou em exercício, apresentam elevada pressão ventricular esquerda e regurgitação mitral (RM) secundária, evoluindo, habitualmente, com refratariedade ao tratamento medicamentoso.^7^ O quadro, em geral, vem acompanhado de hipertensão pulmonar, disfunção diastólica e resposta inadequada ao aumento do volume sistólico com o exercício e com outros fatores extrínsecos.^39^

As alterações dinâmicas no gradiente provavelmente explicam a variabilidade e as flutuações dos sintomas relatados entre dias ou horas. A obstrução pode ser desencadeada por fatores como posição, hidratação, alimentação, consumo de álcool ou qualquer variável que aumente a contratilidade do VE e o débito cardíaco, como taquicardia ou redução do volume ventricular.^15^ Apesar de o gradiente em repouso guardar relação com o desenvolvimento de IC, paradoxalmente, alguns pacientes, mesmo com gradiente alto, evoluem satisfatoriamente com idade mais avançada acima de 65 anos.^40^

Entre os portadores de CMH com padrão não obstrutivo, a evolução é habitualmente favorável, oligo ou assintomática, com minoria progredindo para quadros avançados. As hospitalizações são infrequentes, o transplante cardíaco fica ao redor de 2 a 3% e são raros os óbitos causados pela própria IC.^41^

### 2.4. Fisiopatologia da Cardiomiopatia Hipertrófica

**Figura 2:**
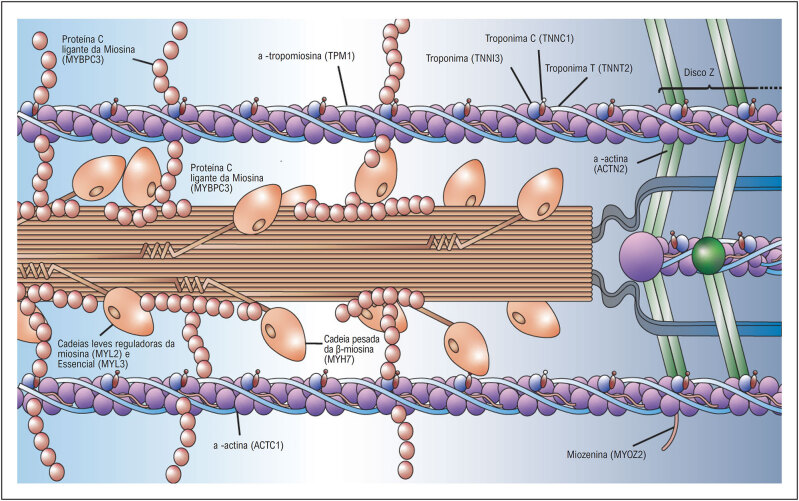
Representação esquemática dos miofilamentos, ativação da miosina e principais mutações sarcoméricas. Adaptada a partir de Baron e Maron.^42^

Vários estudos pré-clínicos indicam que a hipertrofia miocárdica observada na CMH está intimamente ligada ao aumento da contratilidade miocárdica, com aumento da ativação da miosina, e que várias mutações em diferentes componentes do sarcômero têm sido associadas a esse processo ( Figura 2 ).

As manifestações clínicas da CMH são resultantes da interação de vários elementos fisiopatológicos: (1) a obstrução dinâmica da VSVE; (2) a isquemia miocárdica; (3) as arritmias cardíacas; (4) a insuficiência mitral; (5) a disfunção diastólica ventricular e (6) a disfunção autonômica.^2^

### 2.5. Obstrução Dinâmica da Via de Saída do Ventrículo Esquerdo

**Figura 3:**
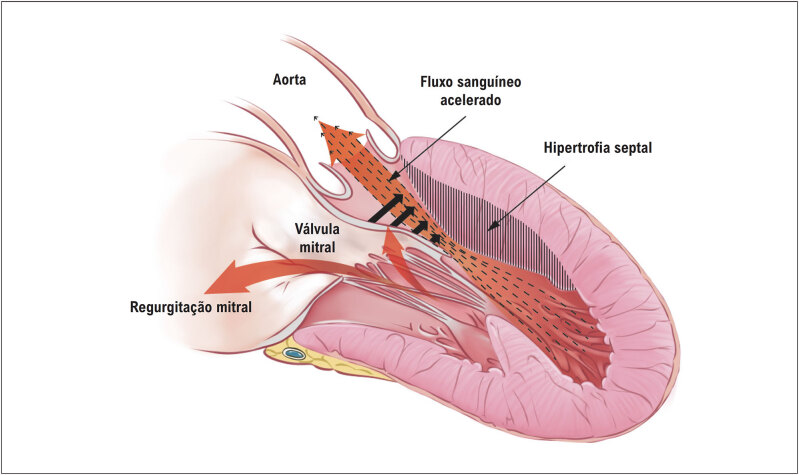
Representação esquemática dos principais fatores que levam à obstrução do trato de saída do ventrículo esquerdo e insuficiência mitral nos pacientes com CMH, com destaque para o movimento sistólico anterior do folheto anterior da válvula mitral (setas negras grossas). Adaptada a partir de Young et al.^50^

A obstrução do trato de saída do ventrículo esquerdo (OTSVE), em repouso ou dinâmica (induzida por manobras provocativas), está presente em aproximadamente 75% dos pacientes com CMH.^14^ Os dois mecanismos principais são: a hipertrofia septal com estreitamento do TSVE, levando a fluxo sanguíneo anormal que desloca anteriormente os folhetos da válvula mitral; e alterações anatômicas na valva mitral e ou deslocamento dos músculos papilares e aparelho valvar mitral ( Figura 3 ). Por causar aumento da pressão sistólica do VE, a OTSVE também pode agravar a hipertrofia ventricular esquerda (HVE), insuficiência miocárdica, isquemia e prolongar o relaxamento ventricular.^43 - 45^ A detecção de um gradiente de pico no TSVE (trato de saída do ventrículo esquerdo) ≥ 30 mmHg classifica a CMH como obstrutiva e a detecção de gradientes em repouso ou dinâmicos (durante manobras provocativas) ≥ 50 mmHg definem obstrução significativa do TSVE, podendo ser considerada a intervenção com terapias de redução septal, a depender dos sintomas e da refratariedade ao tratamento clínico.^43 - 44^

Modificações no gradiente do TSVE podem ocorrer por modificações de pré-carga, da pós-carga ou da contratilidade miocárdica, secundárias a estímulos, tais como: com atividades diárias, respiração, alimentos e ingestão de álcool.^46^ Manobras provocativas, com realização simultânea de ecocardiografia, podem ser necessárias em pacientes com gradiente em repouso ausente ou com valores pico < 30 mmHg, para induzir a obstrução TSVE (OTSVE), tais como: manobra de Valsalva, posição ortostática, inalação de nitrito de amilo e exercício físico.^47 - 49^ Conforme a localização e as suas características anatômicas, a obstrução pode se caracterizar como valvular, dinâmica subvalvular no TSVE, músculos papilares hipertrofiados, inserção anômala do músculo papilar ou obstrução muscular causada por hipercinesia compensatória após infarto.^46 - 49^

### 2.6. Isquemia Miocárdica

Classicamente, a isquemia miocárdica nos pacientes com CMH é secundária ao desequilíbrio entre oferta e consumo de oxigênio de modo análogo ao que ocorre na doença coronária aterosclerótica, mas sem a presença de placas de ateroma nas artérias coronárias epicárdicas. Quando há doença aterosclerótica sobreposta, acrescenta gravidade ao prognóstico, com menor sobrevida e maior risco de morte súbita.^36^ Entretanto, os mecanismos de isquemia na CMH são muito mais complexos. Apesar de elevado fluxo e baixa resistência coronariana basal,^17^ os pacientes com CMH têm reduzida reserva coronariana ao serem submetidos à taquicardia, com menor limiar isquêmico e significativa taxa de *angina pectoris.* ^17 , 35^ O referido desequilíbrio é resultante de diversos fatores. Os elementos que diminuem a oferta de oxigênio são: (a) a disfunção microvascular; (b) a diminuição da densidade das arteríolas em relação ao miocárdio hipertrofiado; (c) a hipertrofia da camada média das arteríolas; (d) o aumento das pressões intracavitárias; e (e) a presença de ponte miocárdica com obstrução significativa ao fluxo.^18 , 51^ O aumento do consumo miocárdico de O_2_ também concorre para tal desequilíbrio, e os elementos determinantes são: (a) a hipertrofia miocárdica; (b) o estado hiperdinâmico do paciente.^2 , 18 , 51^

A importância clínica da isquemia nos pacientes com CMH reside em suas consequências deletérias, tais como a instabilidade elétrica do cardiomiócito e consequentes arritmias malignas, a indução de fibrose difusa miocárdica ou áreas de infarto miocárdico e a formação de aneurismas e disfunção sistólica ventricular. A isquemia miocárdica contribui para o mau prognóstico e eventos agudos graves.^2^

### 2.7. Arritmias

Sintomas atribuídos a arritmias são relatados com frequência nos pacientes com CMH. Uma dessas manifestações é a síncope, que, apesar de inexplicada em 91% dos casos, é relatada em 16% dos pacientes com CMH. Eventos arrítmicos são mais frequentes nos pacientes com CMH e síncope do que aqueles sem relato sincopal (7,7% vs. 3,6%). Outros mecanismos de síncope são devido à obstrução da via de saída, usualmente observada no esforço ou logo após o pico do esforço físico; e a síncope vasovagal, devido a quadros de disautonomia, geralmente precedida de pródromos.

O entendimento do mecanismo subjacente da síncope é fundamental para a abordagem terapêutica. O temor da arritmia ventricular maligna como causa é sempre lembrado. Entretanto, o paciente com CMH pode ter como gatilho da síncope o baixo débito secundário à hipovolemia, ao uso de diuréticos ou vasodilatadores ou à anemia grave não compensada.^52 , 53^

A fibrose miocárdica é encontrada na evolução da CMH, e muitos trabalhos mostraram que a fibrose é marcadora de mau prognóstico nessa cardiopatia. Há tentativas de se correlacionar a fibrose com as arritmias na CMH. Entretanto, os resultados dos diferentes estudos não são consensuais. Recentemente, constatou-se que não é a mera presença, mas a extensão da fibrose miocárdica avaliada pelo realce tardio à RMC que se associa ao maior risco de ocorrência de TVNS em pacientes com CMH.^54^

### 2.8. Insuficiência Mitral

A RM pode ocorrer em associação direta com a OTSVE e com o movimento sistólico anterior (MSA) do folheto da válvula mitral ou dever-se a anormalidades estruturais primárias dos folhetos da válvula mitral. O MSA da válvula mitral leva à perda de coaptação do folheto, levando a um jato predominantemente na sístole média a tardia e com orientação posterior ou lateral. Entretanto, jatos centrais e anteriores também podem resultar de MSA da válvula mitral.^15^ Fatores que afetam a gravidade da OTSVE também podem afetar o grau de RM. Anomalias primárias da válvula mitral e de seu aparelho subvalvar também são comuns, incluindo alongamento do folheto, inserção anômala do músculo papilar e músculos papilares deslocados anteriormente.^44 , 45^ A presença de insuficiência mitral pode determinar o agravamento da congestão pulmonar, sendo elemento importante na gênese da dispneia e redução da capacidade funcional nos pacientes com CMH.

### 2.9. Disfunção Ventricular Diastólica

A disfunção diastólica na CMH ocorre por vários mecanismos associados, como não uniformidade na contração e relaxamento ventricular, altas pressões intracavitárias e inativação atrasada pela recaptação anormal do cálcio intracelular, podendo ocorrer intolerância ao exercício ou sintomas de IC na ausência de OSTVE.^9 , 10 , 55^ A rigidez da câmara pode surgir secundária à hipertrofia miocárdica, isquemia e substituição ou fibrose intersticial. Em alguns pacientes, a gravidade da hipertrofia também compromete significativamente o tamanho da cavidade ventricular e o volume sistólico, sendo causas de diminuição da capacidade de exercício, o que traz impacto prognóstico independente da OTSVE.^56^ Com comprometimento do relaxamento miocárdico ventricular, o enchimento ventricular pode depender mais da sístole atrial, levando a uma baixa tolerância à FA, e existe uma associação entre fibrose atrial esquerda, CMH e FA.^57^

### 2.10. Disfunção Autonômica

A prevalência de disfunção autonômica nos pacientes com CMH é incerta. Está descrita a resposta anormal da pressão arterial ao exercício físico, assim como a dificuldade de recuperação da frequência cardíaca e o comprometimento da vasodilatação.^2 , 16^

## 3. Exames Complementares

A abordagem diagnóstica da CMH implica no emprego de métodos de imagem para a identificação fenotípica, tendo como ponto principal a identificação da CMH forma obstrutiva. Como mostrado na Figura Central , após a suspeita baseada na história clínica, exame físico e história familiar, os diversos exames de imagem podem ser empregados, além da avaliação genética.

### 3.1. Eletrocardiografia

Há várias alterações do eletrocardiograma (ECG) relacionadas à CMH, como listadas na Tabela 2 .^58^ Entretanto, essas alterações eletrocardiográficas não possuem um padrão característico, possivelmente devido à expressão genética variável, o que torna inviável a associação clínica e a caracterização de gravidade baseada no ECG. Anatomicamente, os achados eletrocardiográficos podem indicar áreas de fibrose do miocárdio nas paredes anterior e lateral do VE.^58^ O traçado de ECG pode ser anormal em 90% dos indivíduos com CMH e em 75% dos familiares assintomáticos, mas as alterações do ECG não são específicas da doença.^59^ Na população pediátrica, o ECG apresenta-se majoritariamente alterado, nos casos de etiologia sarcomérica antes mesmo do aparecimento de HVE ao ecocardiograma.^60^ Em pacientes com CMH, o ECG pode demonstrar padrões de pseudoinfarto com ondas Q proeminentes inferolaterais e ondas R proeminentes em derivações precordiais, relacionadas ao aumento das forças de despolarização do septo hipertrófico.^59^ A associação de critérios de voltagem para HVE pode estar presente entre as alterações eletrocardiográficas, mas apenas 2% delas são isoladas.^61^ Bloqueios completos de ramo também são incomuns na CMH, cuja presença sugere intervenções invasivas anteriores para aliviar a obstrução da VSVE.^62^

**Tabela 2:** Principais alterações do ECG encontradas nos pacientes com CMH

Desvio do eixo para esquerda;
Anormalidades da onda P, refletindo aumento atrial esquerdo ou biatrial e prolongamento da onda P (um preditor de desenvolvimento futuro de fibrilação atrial).^62^
Ondas Q rápidas e profundas nas derivações inferiores (DII, DIII, avF) ou precordiais, mais comuns nas derivações laterais (D1, aVL, V4-V6) associadas a sinais clássicos de hipertrofia ventricular esquerda.
Alterações da repolarização com infradesnivelamento do segmento ST (> -0,2 mV) e ondas T negativas profundas (ondas T negativas gigantes); nas derivações laterais é um marcador de CMH apical.^62 - 65^
O intervalo QTc > 480 ms é mais comum em pacientes com CMH e reflete hipertrofia cardíaca, fibrose e obstrução da via de saída.

**Figura 4:**
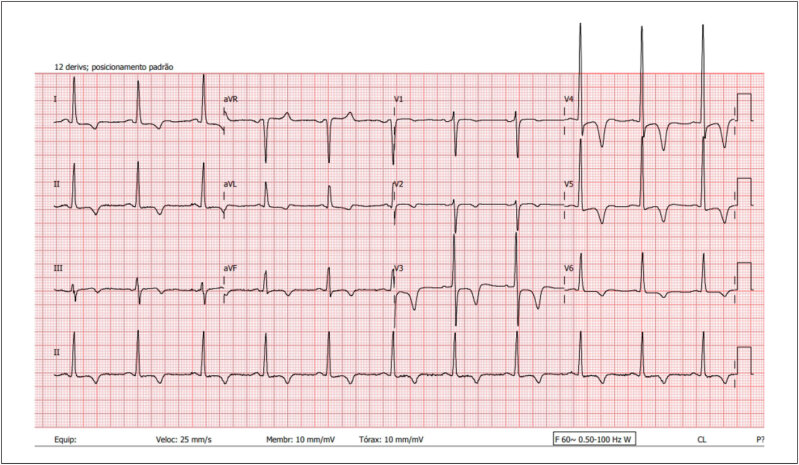
Traçado eletrocardiográfico de 12 derivações em repouso de paciente portador de CMH forma apical, mostrando hipertrofia ventricular esquerda e ondas T negativas e profundas na parede ântero-septal e lateral. Fonte: Arquivo pessoal dos autores.

Cumpre salientar que o ECG pode mudar significativamente ao longo da progressão da doença. Numa fase inicial, podemos encontrar aumento das voltagens do complexo QRS e alterações ST-T. Ao longo das décadas, as alterações mais comuns são representadas pelo aumento progressivo do átrio esquerdo e vários graus de prolongamento do QRS, refletindo fibrose septal e envolvimento do tecido de condução. Nos estágios posteriores, quando a carga de fibrose miocárdica se torna significativa, o bloqueio do ramo esquerdo pode se desenvolver.^62 , 66^ Podemos encontrar achados característicos da CMH forma apical ( Figura 4 ).

### 3.2. Biomarcadores

Peptídeos natriuréticos (PN) são biomarcadores estabelecidos no diagnóstico da IC. Eles podem estar alterados em pacientes com manifestação fenotípica de CMH, mas não são específicos para o diagnóstico dessa enfermidade. O peptídeo natriurético tipo B (BNP) e o N-terminal do pró-hormônio do peptídeo natriurético do tipo B (NT-proBNP) correlacionam-se com pior classe funcional e gravidade das alterações ecocardiográficas, tais como: gradiente na TSVE, espessura septal e grau de insuficiência mitral.^67 , 68^ Da mesma forma, troponina cardíaca de alta sensibilidade (TcAS) pode estar alterada. Em um estudo, a TcAS acima do corte de 0,014 ng/mL foi encontrada em 54% dos portadores de CMH e correlacionou-se com parâmetros de gravidade ao ecocardiograma, tais como: espessura septal e diâmetro do átrio esquerdo.^69^ Embora esses biomarcadores não sejam específicos para o diagnóstico da CMH, eles são importantes marcadores prognósticos.^3 , 67 - 69^ PN e TcAS são preditores de eventos cardiovasculares, como morte por todas as causas, morte cardiovascular, transplante cardíaco e hospitalizações por IC. No entanto, não são preditores de morte súbita, nem de implante de CDI.^3 , 67 - 69^ Uma metanálise recente identificou NT-proBNP e proteína C reativa ultrassensível como preditores de morte cardiovascular e TcAS como preditor do evento combinado de IC, arritmias ventriculares malignas e AVC.^70^

### 3.3. Ecocardiograma

**Figura 5:**
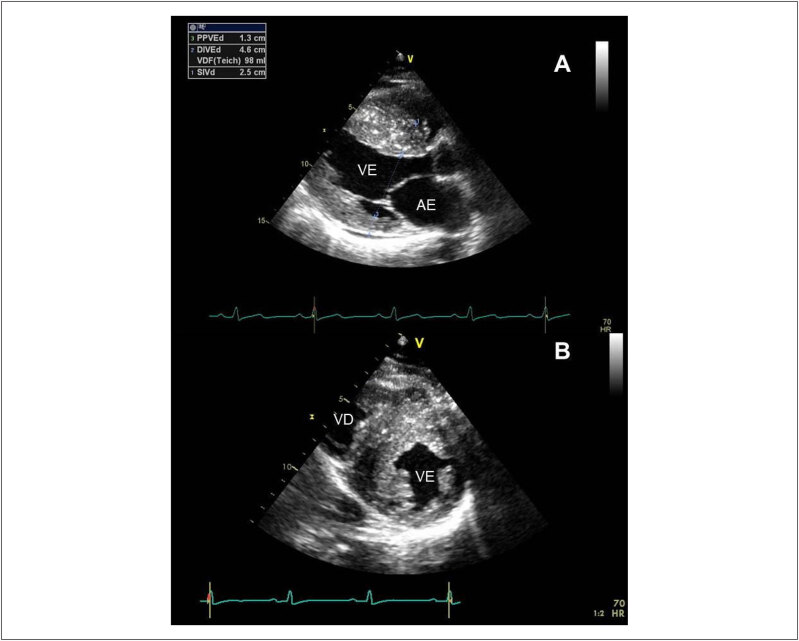
Imagens ao ecocardiograma transtorácico de um paciente com CMH. Observa-se aumento importante da espessura miocárdica (25 mm) da parede septal ao corte paraesternal longitudinal (A) e transversal ao nível dos músculos papilares (B). AE: átrio esquerdo; VE: ventrículo esquerdo; VD: ventrículo direito. Fonte: Arquivo pessoal dos autores.

O ecocardiograma transtorácico (ETT) é o método diagnóstico fundamental e inicial para o diagnóstico de CMH, que é realizado na presença de espessura miocárdica diastólica ≥ 15 mm na ausência de quaisquer condições que justifiquem a HVE em um ventrículo não dilatado ( Figura 5 ).^2^ Em pacientes com história familiar de CMH ou doença conhecida causando mutação genética, uma espessura miocárdica diastólica ≥ 13 mm pode ser diagnóstica. Em crianças, não é possível a aplicação de um simples valor de corte para pacientes de diferentes idades e áreas de superfície corpórea, uma vez que o crescimento deve ser considerado para o diagnóstico. Assim, as medidas cardíacas são expressas em *z-score* , representando desvios padrão a partir de valores médios específicos para o tamanho de cada paciente. Na população pediátrica, o diagnóstico de CMH é realizado na presença de valores com *z-score* > 2.^71 , 72^ As medidas devem ser realizadas na diástole guiadas pelo traçado eletrocardiográfico e no momento do maior diâmetro ventricular esquerdo, evitando encurtamentos e a inclusão de cordoalhas e outras estruturas como trabeculações nessa medida. A identificação da hipertrofia ventricular à avaliação ecocardiográfica faz necessária a avaliação durante o exame de causas que possam justificar a presença de HVE, como a presença de estenose aórtica, membrana subaórtica e coarctação aórtica.^73^ O uso do contraste tem ainda mais valor, particularmente nos pacientes com genética positiva identificada no rastreamento de familiares de casos confirmados de CMH, alterações sugestivas de CMH ao ECG e também nas formas apicais. A avaliação da massa miocárdica pela ecocardiografia tridimensional (Eco 3D) em pacientes com CMH tem melhor correlação com as medidas obtidas pela RMC quando comparada às medidas pela ecocardiografia bidimensional (Eco 2D) e deve ser o método de escolha se disponível para esta finalidade.^74 , 75^ A espessura do ventrículo direito (VD) da parede livre deve ser medida no corte subcostal ao final da diástole e não deve incluir a gordura epicárdica.^76^ A Tabela 3 contempla as informações importantes no laudo do ecocardiograma dos pacientes com CMH.

**Tabela 3:** Informações importantes no laudo do ecocardiograma dos pacientes com CMH

Volume indexado do AE
Espessura do SIV, PP
Localização dos segmentos com aumento da espessura miocárdica
Fração de ejeção ventricular esquerda
Descrição de aneurisma apical quando presente
Avaliação de *strain* longitudinal global se disponível
Análise da função diastólica
Descrição da presença de gradiente intraventricular (em VSVE, médio ventricular ou apical) e mensuração do gradiente em repouso e após manobras provocativas
Descrever presença de MSA da VM
Descrever anatomia e presença de anormalidades da VM, bem como dos músculos papilares e aparato subvalvar
Descrição da insuficiência mitral e mecanismos

AE: átrio esquerdo; SIV: septo interventricular; PP: parede posterior; VSVE: via de saída ventricular esquerda; MSA: movimento sistólico anterior; VM: valva mitral.

#### 3.3.1. Avaliação da Função Sistólica

**Figura 6:**
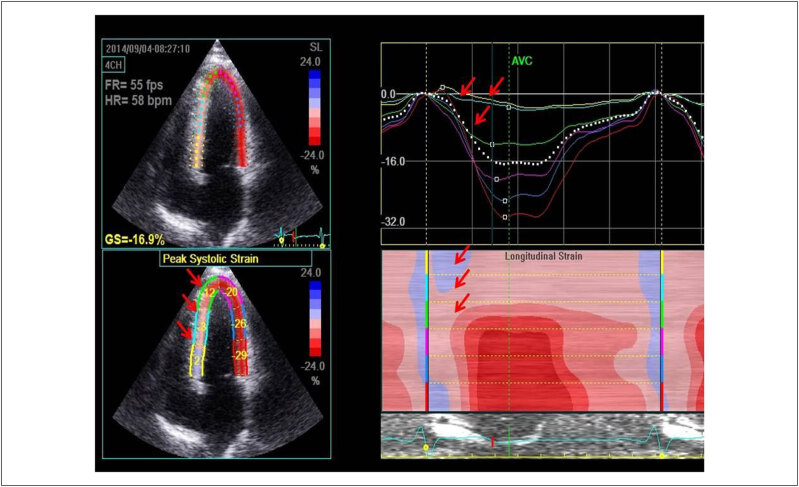
Representação da avaliação do strain longitudinal em paciente com CMH. Observa-se redução dos valores do strain longitudinal nos segmentos (basal, médio e apical) hipertróficos da parede septal (setas) no corte apical 4 câmaras. Fonte: Arquivo pessoal dos autores.

**Figura 7:**
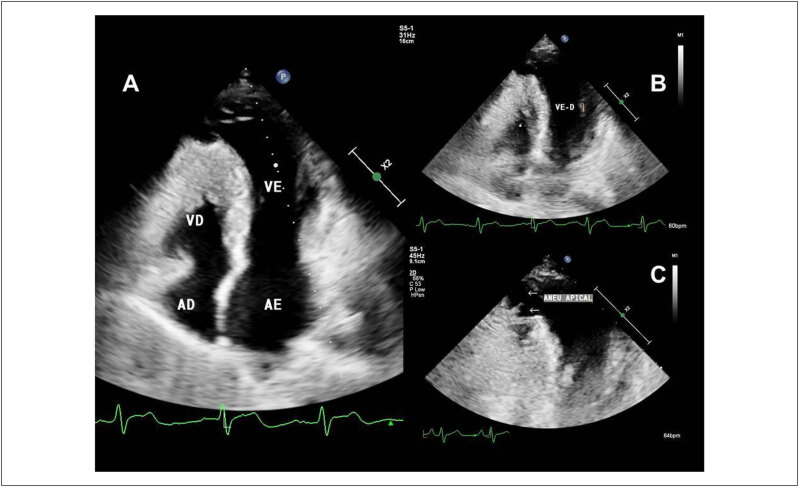
Imagens ao ecocardiograma transtorácico de paciente com MCH evidenciando aneurisma apical ao corte apical 4 câmaras (A e B) e apical 2-câmaras (C). AD: átrio direito; AE: átrio esquerdo; VD: ventrículo direito; VE: ventrículo esquerdo. Fonte: Arquivo pessoal dos autores.

A CMH apresenta-se, comumente, com fração de ejeção ventricular esquerda (FEVE) preservada ou até hiperdinâmica. Menos de 10% dos pacientes com CMH apresentam redução da FEVE, e valores de FEVE < 0,50 indicam pior prognóstico. Para suprir as limitações relacionadas à análise da FEVE biplanar pelo método de Simpson, a análise da mecânica cardíaca através do *strain* longitudinal global (GLS) surge como uma ferramenta promissora no diagnóstico diferencial das diversas fenocópias de hipertrofia ventricular ( Figura 6 )^2 , 75^ e melhor estratificação prognóstica nos casos com FEVE preservada. Apesar de ser uma ferramenta conhecida e pesquisada há mais de uma década, tem maior relevância a sua análise visual paramétrica, principalmente na diferenciação com a Doença de Fabry e com a amiloidose cardíaca.^74 , 76^ É bem esclarecida a relação inversa entre espessura miocárdica, assim como a quantificação de fibrose do VE e o GLS. Este método de imagem permite também a identificação de aneurisma apical, que está associado com maior risco de morte súbita ( Figura 7 ).

#### 3.3.2. Avaliação da Função Diastólica

A disfunção diastólica de VE é consequência da doença, secundária ao enrijecimento miocárdico, impactando a fase precoce de enchimento ventricular, e também à redução de complacência, a qual se manifesta na sua fase tardia e está associada às alterações de geometria e função atrial esquerda.^2^ Os parâmetros de maior acurácia diagnóstica para indicar a presença de disfunção diastólica são: volume do átrio esquerdo (> 34 mL/m^2^),^71 , 72^ Doppler tecidual em anel mitral (lateral e septal, se inferiores a 8 cm/s), elevação de pressão sistólica em artéria pulmonar estimada pela velocidade da regurgitação tricúspide (> 2,8 m/s) e valores de velocidade de enchimento precoce pela valva mitral (E), esta última melhor se comparada à relação das velocidade de Doppler tecidual como E/e´ média (se > 14).^73^ Em decisões mais difíceis, a mensuração da duração de ondas A (pela valva mitral) ou de Ar (pelo refluxo em veias pulmonares) pode ser de valia, lembrando-se que tais parâmetros estarão ausentes na FA, tão comum na evolução da doença. Na presença de FA, parâmetros como tempo de desaceleração mitral (TD < 150 mseg) e a relação E/e´ elevada são os de melhor acurácia. É importante lembrar que, quando houver insuficiência mitral de grau moderado ou maior, pode haver superestimação das velocidades de Doppler tecidual e ainda maiores aumentos de átrio esquerdo, que devem ser considerados de maneira individualizada. Há evidências de que a presença de padrão restritivo de disfunção diastólica de VE (grau III ou IV) associa-se à pior prognóstico nesse grupo de pacientes, independentemente da presença de obstrução ao fluxo em VSVE. A análise de função atrial esquerda pelo *speckle tracking* pode mostrar alterações precoces com potencial valor prognóstico para desfechos secundários, como o desenvolvimento de FA. Ainda, o uso de testes sob estresse físico pode trazer informações importantes acerca da função diastólica nesses pacientes, estando indicado não só na pesquisa da obstrução dinâmica, como também da presença de disfunção diastólica incipiente^74^ e, portanto, deve incluir a análise dos parâmetros elencados acima após esforço físico.

### 3.4. Avaliação de Obstrução do TSVE e Insuficiência Mitral

A obstrução dinâmica na VSVE é de extrema importância clínica pelo seu impacto na morbimortalidade, estando presente em aproximadamente 2/3 dos indivíduos, quando considerarmos a quantificação em repouso e com manobras provocativas.^72 , 73 , 76^ Além do grau e localização da hipertrofia septal, as alterações do aparato valvar e subvalvar têm um importante papel na gênese da obstrução dinâmica do TSVE. O resultado da interação dinâmica entre o septo e a valva mitral nos fenótipos obstrutivos é a obstrução progressiva do fluxo na VSVE pela cúspide anterior redundante, fenômeno conhecido como movimento sistólico anterior (MSA). Por definição, o paciente é considerado como portador da forma obstrutiva na presença de gradiente máximo ≥ 30 mmHg em repouso ou com manobras provocativas, como manobra de Valsalva ou esforço físico.^74^ Uma série de alterações morfológicas valvares e subvalvares ajuda a entender o mecanismo dinâmico da obstrução do fluxo, assim como possibilita direcionar para o diagnóstico etiológico da MCH frente às outras fenocópias que cursam com hipertrofia assimétrica e obstrução dinâmica em VSVE, como doença de Fabry e amiloidose cardíaca, por exemplo.^76 , 77^ A presença de anomalias do músculo papilar favorece a redução da distância entre os papilares e o septo. Assim, além de facilitar a obstrução em VSVE, essas alterações podem resultar em obstrução dinâmica na porção média do ventrículo.^26^ O deslocamento anterior dos músculos papilares tem papel fundamental na gênese do MSA, assim como a hipertrofia, e também contribui para a aproximação do septo, bem como para uma diminuição da tensão na cúspide anterior, tornando-a mais redundante.^78^ A inserção anômala dos papilares ocorre em diferentes apresentações, desde seu deslocamento apical, até alterações de sua inserção, podendo ocorrer inserção direta da musculatura na cúspide anterior, lembrando a valva mitral em arcada, até inserção direta na VSVE. Tais alterações na disposição dos papilares somam alterações fisiopatológicas, favorecendo direta e indiretamente a obstrução dinâmica na VSVE.^79^ Achados adicionais também são descritos, como presença de falsa corda, cordas secundárias, bandas musculares, alongamento da cúspide anterior, hipertrabeculação apical que favorece disfunção dos papilares. Os pacientes com fenótipos obstrutivos se apresentam mais sintomáticos, assim como existe associação com morte súbita, devendo-se reforçar a importância em pesquisar a obstrução dinâmica por meio de manobras provocativas com manobra de Valsalva, nitrito de amilo, quando disponível, ou com estresse físico para desmascarar o que corresponde a quase 1/3 dos pacientes. A avaliação da gravidade da insuficiência mitral nos pacientes com obstrução em VSVE, visto que o mecanismo de ambos está relacionado ao MSA, é muito desafiadora. De qualquer forma, uma avaliação integrada entre o volume do átrio esquerdo, o grau de obstrução em VSVE e a extensão do jato regurgitante podem fornecer dados para ao menos uma quantificação qualitativa do grau de insuficiência. Uma outra limitação inclui a dificuldade em se diferenciar o jato de insuficiência mitral e o de obstrução em VSVE. Como regra geral, jatos em saída com velocidade acima de 5,5 m/s devem ser suspeitos de contaminação com a insuficiência mitral. Além disso, a curva ao estudo Doppler da insuficiência mitral possui formato arredondado e está presente durante a contração isovolumétrica, enquanto o jato de obstrução dinâmica da VSVE possui formato em adaga típica, com pico tardio, indicando o atraso protossistólico na ejeção.

#### 3.4.1. *Screening* de Familiares

O ETT é o método de escolha no rastreamento de pacientes com suspeita de CMH integrada à avaliação clínica e eletrocardiográfica. A periodicidade dos exames em pacientes assintomáticos e familiares de pacientes com diagnóstico de CMH varia com a idade do paciente, com a patogenicidade do gene envolvido na doença, além da idade de início dos sintomas nos familiares acometidos. Nos pacientes assintomáticos, crianças e adolescentes com variantes patogênicas e/ou início precoce dos sinais e sintomas, o ETT deve ser realizado a cada 1 ou 2 anos e, na ausência dessas duas condições, a cada 2 ou 3 anos. Em adultos, o rastreamento é recomentado a cada 3 ou 5 anos. Porém, independentemente da idade, a avaliação deve ser realizada em qualquer momento na presença de sintomas como dispneia, palpitações, tonturas ou síncope ( Tabela 4 ).

**Tabela 4:** Periodicidade sugerida para o rastreio de pacientes assintomáticos familiares de casos confirmados de CMH

Situação clínica	Periodicidade de realização do ETT
Crianças e adolescentes com variantes patogênicas E/OU com familiares com início precoce dos sinais e sintomas de CMH	1 ou 2 anos
Crianças e adolescentes sem as condições descritas acima	2 a 3 anos
Adultos	3 a 5 anos
Avaliação clínica, ETT e ECG em qualquer momento na presença de sintomas como dispneia, palpitações, tonturas ou síncope.	

ETT: ecocardiograma transtorácico; CMH: cardiomiopatia hipertrófica; ECG: eletrocardiograma.

A RMC deve ser reservada para pacientes com janela acústica limitada para a realização do ETT ou em pacientes com alterações no ECG, mas com ETT aparentemente normal.

A Tabela 5 contempla as recomendações para o emprego da ecocardiografia em pacientes com CMH.

**Tabela 5:** Recomendações para emprego da ecocardiografia em pacientes com CMH

Recomendação	Classe de recomendação	Nível de evidência
O ETT é recomendado na avaliação inicial de pacientes suspeitos de CMH.	I	B
Repetir o ETT é recomendado em pacientes com CMH exibindo mudança do estado clínico ou que tenham apresentado evento clínico ou complicação.	I	B
Repetir o ETT é recomendado em pacientes com CMH a cada 1 ou 2 anos, mesmo na ausência de progressão de sintomas ou complicações, para avaliar grau de HVE, obstrução do TSVE, regurgitação mitral e função sistólica do VE.	I	C
Realizar o ETT com manobras provocativas (manobra de Valsalva e/ou ortostase) é recomendado nos pacientes com CMH com gradiente no TSVE < 50 mmHg.	I	B
Recomenda-se realizar ETT com esforço em pacientes com CMH sintomáticos que não apresentem gradiente do TSVE ≥ 50 mmHg em repouso ou manobra provocativa.	I	B
O ETT com esforço pode ser realizado em pacientes com CMH assintomáticos que não apresentem gradiente do TSVE ≥ 50 mmHg em repouso ou manobra provocativa.	IIa	C
O ETT com uso de contraste ecocardiográfico pode ser usado em situações em que o ETT sem contraste é inconclusivo quanto à caracterização da HVE.	IIa	C
O ETE pode ser usado em pacientes com CMH em que o ETT é inconclusivo ou para o planejamento de TRS ou melhor avaliação do aparato valvar mitral.	IIa	C
Recomenda-se a realização de ETE durante a realização de miectomia cirúrgica.	I	B
Recomenda-se a realização de ETE ou ETT, com uso de contraste ecocardiográfico, durante procedimento de alcoolização septal, para avaliar o território de irrigação da artéria septal alvo do procedimento.	I	B
Recomenda-se realizar o ETT 3 a 6 meses após o TRS para avaliar o resultado do procedimento.	I	B
Recomenda-se realizar o ETT em parentes de primeiro grau de pacientes com CMH, como estratégia inicial de *screening* .	I	B

ETT: ecocardiografia transtorácica; ETE: ecocardiografia transesofágica; CMH: cardiomiopatia hipertrófica; HVE: hipertrofia ventricular esquerda; TSVE: trato de saída do ventrículo esquerdo; VE: ventrículo esquerdo; TRS: terapia de redução septal.

### 3.5. Ressonância Magnética Cardíaca

**Figura 8:**
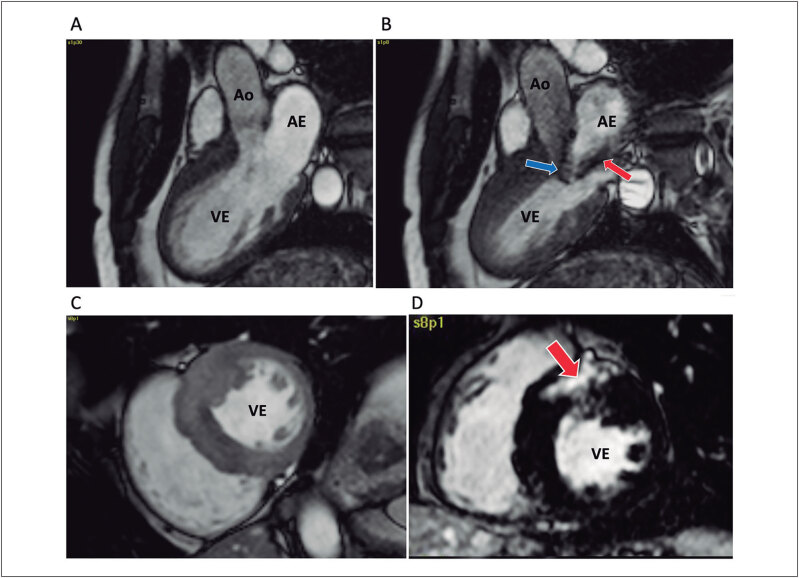
RMC de pacientes de 49 anos com CMH com obstrução significativa da VSVE em repouso com movimento anterior sistólico do folheto anterior da valva mitral com consequente regurgitação mitral moderada. A: Imagem em cine SSFP diastólica em corte de 3 câmaras. B: Imagem em cine SSFP sistólica em corte de 3 câmaras. A seta azul mostra o movimento anterior sistólico do folheto anterior da valva mitral. A seta vermelha mostra a regurgitação mitral. C: Imagem diastólica em SSFP do eixo curto medial, mostrando maior espessura de 28 mm, no segmento anterosseptal medial. D: Realce tardio em eixo curto mostrando realce tardio com padrão mesocárdico (seta vermelha) acometendo 8% da massa do VE. VE: ventrículo esquerdo, Ao: aorta, AE: átrio esquerdo. Fonte: Arquivo pessoal dos autores.

A RMC tem se transformado em importante exame complementar para a confirmação diagnóstica, para estabelecer o prognóstico e auxiliar no planejamento terapêutico — incluindo a prevenção de morte súbita — em pacientes com suspeita clínica ou com diagnóstico estabelecido de CMH. Os principais atrativos desse exame incluem a resolução temporal satisfatória, a boa resolução espacial e, especialmente, a elevada resolução de contraste, que permite identificar áreas de defeitos de perfusão, edema e fibrose miocárdica. A RMC possibilita identificar formas não convencionais de CMH, como nos casos de formas apicais, de hipertrofias mais localizadas e a presença de defeitos adicionais, tais como aneurisma de ponta de VE. Adicionalmente, a grande reprodutibilidade do método o torna a opção ideal para confirmar os volumes, as massas, a espessura e a função sistólica de ambos os ventrículos e, com o emprego de técnicas mais modernas de obtenção dinâmica de imagens, também faculta estimar o gradiente presente na VSVE. Em decorrência disso, o papel da RMC na avaliação da CMH vem aumentando de modo significativo^26^^,^^76 - 80^ ( Figura 8 ).

A RMC pode ser utilizada para identificar ou confirmar a presença de áreas de aumento patológico da espessura miocárdica em pacientes que apresentem quadro clínico e de outros exames que tenham levantado a suspeita de CMH, em especial quando há ecocardiogramas inconclusivos ou conflitantes. Além disso, o exame permite a avaliação precisa de outras características anatômicas, tais como o implante anormal dos músculos papilares, a extensão dos folhetos valvares, a presença de implante direto dos músculos papilares na valva mitral, feixes musculares acessórios, feixes e bandas musculares anormais, com o objetivo de confirmar ou de completar a avaliação desses elementos feita pela ecocardiografia. Tais elementos são particularmente relevantes quando se trata de candidatos a tratamento cirúrgico ou intervencionista da hipertrofia miocárdica.^26 , 76 , 78 , 81 , 82^ A redução da função contrátil do VE, em especial se acompanhada de dilatação da cavidade, marca pior prognóstico desses casos, e a RMC pode ter papel fundamental nesses casos, principalmente se houver resultados conflitantes ou inconclusivos pelo ecocardiograma. De modo semelhante, a detecção de defeitos localizados de contratilidade ventricular, tal como discinesias e aneurismas de ápice ventricular esquerdo, é avaliada de forma mais precisa pela RMC. Particular interesse tem o encontro de regiões afiladas em segmentos do VE, pois estas estão, muitas vezes, associadas à presença de fibrose e podem estar associadas a pior prognóstico, e tais achados são vistos de modo preciso pela RMC.^26 , 78 - 81 , 83^

A principal contribuição da RMC, porém, dá-se na caracterização tecidual, destacadamente por meio das técnicas de realce tardio. Essa modalidade de imagem reproduz de modo adequado a presença e quantidade de fibrose que compromete o músculo cardíaco de pacientes com diferentes tipos de cardiomiopatia. Nos casos em que há realce tardio, destacadamente se este comprometer mais de um segmento, há pior prognóstico, com maior risco de morte súbita por arritmia. Quando há 15% ou mais da massa miocárdica comprometida por realce tardio, esse risco aumenta ao menos duas vezes. Além disso, áreas extensas de realce tardio também podem indicar maior possibilidade de evolução para formas dilatadas, com desenvolvimento de disfunção ventricular. Se, por um lado, ainda há certa controvérsia no sentido de definir qual a melhor forma de quantificar a massa total de realce tardio entre os diferentes investigadores, por outro, concorda-se que, se o realce comprometer poucas regiões ou se ele não existir, há baixo risco de acontecer morte súbita por arritmia.^26 , 78 , 79 , 83 - 85^ Estudos recentes demonstraram que a quantidade de realce se relaciona com diferentes mutações que levam à hipertrofia e que esses elementos têm poder prognóstico aditivo.^86^

As técnicas de realce tardio são particularmente úteis para o diagnóstico diferencial das distintas causas de fenocópias de hipertrofia miocárdica. Padrões de realce são diferentes em casos de amiloidose, sarcoidose, doença de Fabry e outras mutações na maioria das vezes, e o uso dessa modalidade de avaliação miocárdica pode ser fundamental para estabelecer o diagnóstico e guiar o tratamento. Particular relevância tem a diferenciação de portadores de CMH e atletas, pelo risco potencial que a prática de atividades físicas competitivas traz em portadores de CMH.^78 , 84 , 86^

A despeito de suas contribuições relevantes, o realce tardio mostra algumas limitações. Há superposição da morfologia de algumas miocardiopatias, e as alterações podem não estar presentes em períodos mais precoces da doença. Em virtude disso, foi desenvolvida outra forma de caracterização tecidual por RMC, conhecida como mapa T1. Esse é um modo de analisar alterações no miocárdio que possam não ser identificáveis visualmente, mas que já se encontrem em andamento e cuja percepção possa permitir o diagnóstico e o manejo adequado. As alterações no mapa T1 e na quantificação do espaço extracelular (mensuração do mapa T1 antes e depois da injeção do meio de contraste paramagnético) são úteis para distinguir a CMH da doença de Fabry, bem como para realizar a diferenciação em fases iniciais de pacientes com aumento da espessura secundária à hipertensão daqueles com CMH.^86 - 88^ Dados preliminares sugerem que alterações do mapa T1 possam estar associadas à maior ocorrência de arritmias e que possam vir a ser implementadas nas calculadoras de risco de morte súbita nesse grupo de pacientes.^89^

A Tabela 6 contempla as recomendações para o emprego da RMC em pacientes com CMH.

**Tabela 6:** Recomendações para o emprego da RMC em pacientes com CMH

Recomendações	Classe de recomendação	Nível de evidência
Recomenda-se o emprego da RMC para confirmar o diagnóstico em casos de ecocardiografia inconclusiva.	I	B
Recomenda-se o emprego da RMC para realizar diagnóstico diferencial de fenocópias de hipertrofia miocárdica, incluindo coração de atleta, amiloidose, doença de Fabry e hipertrofia secundária à hipertensão.	I	B
Recomenda-se o emprego da RMC para o aprimoramento da estratificação de risco, incluindo melhor análise de alterações anatômicas e da presença de realce tardio.	I	B
Recomenda-se o emprego da RMC para a complementação diagnóstica para a medida da espessura miocárdica, variações da anatomia dos músculos papilares, diagnóstico de isquemia miocárdica e definição da presença de obstrução na via de saída do VE.	I	B
O emprego da RMC deve ser considerado para a identificação e o diagnóstico de realce tardio para estimar o risco de morte súbita e, como critério adicional, para decidir pelo implante de desfibrilador, em especial nos casos em que os outros critérios não foram conclusivos.	IIa	B
O emprego da RMC deve ser considerado para aprimorar a análise da anatomia e dos mecanismos de obstrução da via de saída do VE em pacientes candidatos a tratamento cirúrgico ou intervencionista.	IIa	B

RMC: ressonância magnética cardiovascular; VE: ventrículo esquerdo.

### 3.6. Tomografia Computadorizada

A experiência com o uso da tomografia computadorizada no estudo das cardiomiopatias ainda é limitada a poucos centros, mas o exame mostrou-se útil também para o estudo da função sistólica do VE e até mesmo para detectar fibrose miocárdica, podendo servir de opção quando a ecocardiografia não apresentar resultados conclusivos e quando a RMC não se encontrar disponível. Em virtude da elevada resolução espacial, a tomografia pode revelar alterações anatômicas envolvendo a valva mitral, os músculos papilares e eventuais feixes miocárdicos atípicos.^90 - 92^

A tomografia também pode contribuir no sentido de avaliar de modo mais preciso as artérias coronárias, identificando obstruções (que podem estar presentes em 7 a 19% dos casos), diagnosticando a presença de trajetos intramiocárdicos, apontando casos em que possa haver compressão significativa por parte do músculo cardíaco e também possibilitando a avaliação dos primeiros ramos septais, dado fundamental nos casos em que se planeja realizar tratamentos intervencionistas, em especial em candidatos ao tratamento percutâneo.^92 - 95^

A Tabela 7 contempla as recomendações para o emprego da RMC em pacientes com CMH

**Tabela 7:** Recomendações para o emprego da TC em pacientes com CMH

Recomendações	Classe de recomendação	Nível de evidência
A TC é recomendada para a avaliação não invasiva de doença coronária e do trajeto das artérias coronárias em pacientes com diagnóstico de cardiomiopatia hipertrófica.	I	B
A TC é recomendada para a análise das artérias coronárias e dos ramos septais para o planejamento de tratamento invasivo da cardiomiopatia hipertrófica.	I	B
A TC deve ser considerada para a avaliação de dor torácica em pacientes com cardiomiopatia hipertrófica com risco intermediário para doença arterial coronária.	IIa	C
A TC pode ser considerada para a avaliação da anatomia e função ventricular em pacientes com suspeita de cardiomiopatia hipertrófica com ecocardiogramas conflitantes ou inconclusivos com contraindicação à ressonância ou sem ressonância disponível.	IIb	C
A TC pode ser considerada para a identificação e quantificação de realce tardio em pacientes com diagnóstico de cardiomiopatia hipertrófica com ecocardiogramas conflitantes ou inconclusivos com contraindicação à ressonância ou sem ressonância disponível.	IIb	C

TC: tomografia computadorizada.

### 3.7. Estudo Genético

#### 3.7.1. Importância do Diagnóstico Genético

Nas situações em que o diagnóstico clínico é uma certeza, o estabelecimento do defeito molecular através de análises do DNA constitui-se apenas em confirmação diagnóstica. Ainda assim, o estabelecimento do diagnóstico molecular pode contribuir para aumentar a certeza diagnóstica em casos incertos no qual o paciente possui hipertrofia limítrofe ou moderada, como em hipertrofia miocárdica identificada em atletas ou pacientes hipertensos suspeitos de também apresentarem CMH ( **ver Fenocópias na CMH** ).^96 , 97^

O diagnóstico molecular de mutações também permite a identificação de crianças e adultos com manifestações subclínicas da doença. Esses indivíduos, especialmente quando no contexto de uma família com CMH, seriam candidatos a um controle mais rígido de fatores de risco ao desenvolvimento de CMH, assim como de uma monitorização médica mais rigorosa.

Finalmente, deve-se entender que o diagnóstico molecular, especialmente em indivíduos assintomáticos, não significa doença e, sim, um risco aumentado ao desenvolvimento da mesma (vide genótipo positivo fenótipo negativo).^98^

**Figura 9:**
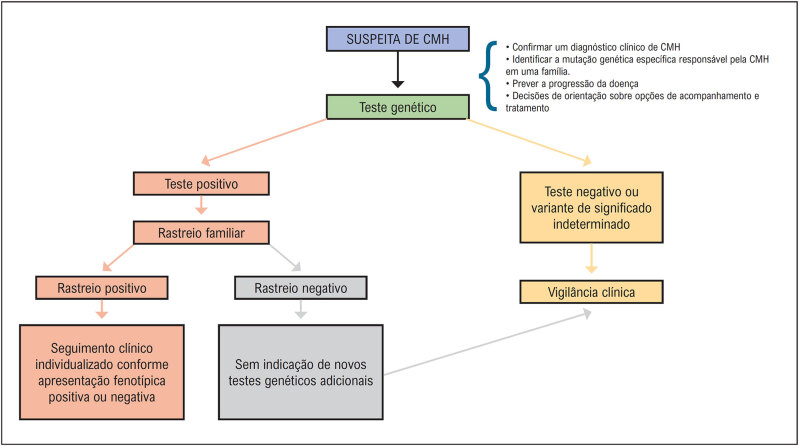
Aplicação do teste genético no cenário clínico da suspeita de CMH. CMH: cardiomiopatia hipertrófica.

De acordo com trabalhos previamente publicados, o rastreamento genético de pacientes com CMH e seus familiares é a estratégia mais custo-efetiva quando comparada ao rastreamento clínico isolado.^98^ Uma vez identificada a mutação, o rastreamento de familiares se torna ainda mais importante, porque permite o diagnóstico precoce e propicia um acompanhamento adequado dos pacientes portadores, além de assegurar ao familiar que não tem a mutação de que não existe risco do desenvolvimento da doença. A Figura 9 ilustra a aplicação do teste genético no cenário da suspeita de CMH.

A Tabela 8 sumariza as vantagens da aplicação do teste genético nos pacientes com CMH.

**Tabela 8:** As vantagens do teste genético na CMH

Maior precisão do diagnóstico
Melhor compreensão da progressão da doença e avaliação de risco
Decisões de tratamento e gerenciamento mais informadas
Capacidade de identificar portadores de CMH nas famílias e fornecer aconselhamento genético
Potencial para medicina personalizada baseada em mutações genéticas específicas

CMH: cardiomiopatia hipertrófica.

O sequenciamento de nova geração permite a realização do diagnóstico de forma mais rápida e barata, além de abrir a oportunidade de se incluir mais genes na análise sem aumento substancial do custo e é considerado o método de escolha para testes genéticos na CMH.

O número de genes e sua associação com diferentes diagnósticos diferenciais varia de acordo com os diferentes provedores de serviço e deve ser avaliado individualmente em cada caso (ver as mutações mais frequentes em Mutações sarcoméricas).

#### 3.7.2. Mutações Sarcoméricas

As mutações sarcoméricas referem-se a alterações genéticas que ocorrem nos genes responsáveis pela formação do sarcômero, que é a unidade funcional das fibras musculares. Essas mutações podem levar a vários distúrbios musculares, como CMH, cardiomiopatia dilatada e distrofia muscular das cinturas. A gravidade e o tipo de doença muscular causada por mutações sarcoméricas podem variar amplamente, variando de fraqueza leve a comprometimento cardíaco ou muscular esquelético grave.

Os testes genéticos permitiram confirmar que a CMH é, predominantemente, uma doença do sarcômero, com capacidade para estabelecer diagnóstico molecular em 45 a 60% dos casos. A variantes genéticas patogênicas descritas nessa doença ocorrem em várias proteínas do sarcômero. As mais prevalentes (em torno de 70%) são mutações nos genes que codificam a cadeia pesada da beta-miosina (MYH7) e a proteína C de ligação à miosina (MYBPC3). Todos os outros genes envolvidos, incluindo troponina cardíaca I e T (TNNI3, TNNT2), tropomiosina alfa-1 cadeia (TPM1) e miosina cadeia leve 3 (MYL3), apresentam uma frequência menor de variantes patogênicas, e algumas são mutações presentes em apenas uma família. Apesar dos grandes avanços nas técnicas de sequenciamento, ainda não é possível identificar a mutação causal em cerca de 30 a 40% dos doentes com CMH.^96^ A partir desse dado, um crescente corpo de evidências vem surgindo baseado em dados de registros, que evidenciam dois grupos distintos de CMH: aqueles que são mutações sarcoméricas positivas (SARC+) e os que são mutações negativas (SARC-). Pacientes com CMH-SARC+ tendem a apresentar-se mais jovens ao diagnóstico, com história familiar mais frequente de CMH ou morte súbita, com maior grau de hipertrofia (predominantemente assimétrica), associados com mais fibrose miocárdica, menos obstrução da VSVE em repouso e maior associação com morte súbita quando comparados com os pacientes com CMH-SARC-.^86 , 99^ Eles também tendem a ter mais disfunção microvascular, o que pode estar relacionado ao maior desenvolvimento de fibrose. Em contrapartida, pacientes com CMH-SARC- apresentam, frequentemente, outras comorbidades (hipertensão arterial e obesidade), maior prevalência de septo sigmoide, menos fibrose e mais obstrução na VSVE.

Um desafio importante continua sendo identificar associações relevantes entre genes ou mutações específicas e desfechos previsíveis, especialmente os de maior risco. Recentemente, o registro internacional ShaRe expandiu esse conceito.^100^ Ele envolveu 4.591 pacientes com CMH com seguimento médio de 5,4 anos e identificou a presença de mutação sarcomérica como um dos preditores de desfechos adversos, incluindo arritmias ventriculares. Observou-se, também, que múltiplas mutações sarcoméricas tendem a se apresentar mais precocemente com um fenótipo mais grave (IC necessitando de transplante e dispositivo de assistência ventricular – DAV).

Com base nessas evidências sobre as mutações sarcoméricas, explora-se a possibilidade de a CMH não ser uma doença exclusivamente monogênica e depender de outros fatores genéticos moduladores e não genéticos relacionados à exposição e outras comorbidades. Isso é particularmente evidente em função da dificuldade de se estabelecerem correlações genótipo-fenótipo e/ou da ocorrência de expressividade variável da doença em pacientes de uma mesma família e portadores da mesma variante genética candidata. É possível que mesmo variantes genéticas caracterizadas como patogênicas ou provavelmente patogênicas possam ser influenciadas por fatores epigenéticos, ambientais e de outras variantes genéticas até então não consideradas.^101 , 102^

Ainda assim, algumas associações genótipo-fenótipo têm sido valorizadas e são apresentadas na Tabela 9 .^103^

**Tabela 9:** Associações entre genótipo e fenótipo na CMH

Gene	Fenótipo
Mutações no filamento fino (TNNT2, TNNI3, TPM1 e ACTC)	Hipertrofia leve e atípica (padrões concêntricos e apicais)
	Aumento da fibrose do VE
	Maior taxa de progressão para IC
	Maior probabilidade de remodelamento levando a disfunção sistólica ou diastólica grave
Mutações na TNNI3	Fenótipo restritivo
Mutações no ACTC	Hipertrofia apical
Mutações no MYH7	Maior taxa de FA (independente de fatores clínicos e ecocardiográficos)
	Idade mais precoce no diagnóstico
	Evolução mais frequente para transplante cardíaco
	Mais arritmias ventriculares
	Mais doença do sistema de condução
	Fenótipo restritivo
Mutações no MYBPC3	Início em idosos e penetrância mais baixa
Mutações no PLN	Taquicardia ventricular não sustentada

VE: ventrículo esquerdo; IC: insuficiência cardíaca; FA: fibrilação atrial. Adaptada de Maron et al.^104^

A Tabela 10 contempla as recomendações para o teste genético na CMH.

**Tabela 10:** Recomendações para o teste genético na CMH

Recomendações	Classe de recomendação	Nível de evidência
Em pacientes com uma apresentação fenotípica de CMH, quando há suspeita de outra condição genética, recomenda-se a investigação genética para CMH e outras causas genéticas de espessamento miocárdico inexplicável ("fenocópias").	I	B
Em pacientes com CMH, deve ser considerada a realização do teste genético para facilitar a identificação de familiares em risco de desenvolver CMH.	IIa	B
A triagem de parentes de primeiro grau de pacientes com CMH com teste genético deve ser considerada.	IIa	B
Em pacientes com CMH, o teste genético na avaliação do risco de MS pode ser considerado.	IIb	B
Para pacientes com CMH que realizaram testes genéticos e não apresentaram variantes patogênicas (ou seja, abrigam apenas variantes benignas/provavelmente benignas), o teste genético familiar não é recomendado	III	B

CMH: cardiomiopatia hipertrófica; MS: morte súbita.

#### 3.7.3. Pacientes Genótipo Positivo/ Fenótipo Negativo

Com o aumento da acessibilidade do mapeamento genético para parentes de pacientes portadores de CMH, temos observado, com maior frequência, um subgrupo de pacientes portadores da mutação genética do sarcômero cardíaco (genótipo positivos, G+), porém sem manifestação fenotípica de hipertrofia ventricular, evidenciada ao ecocardiograma ou RMC, fenótipo negativo (F-).^105 , 106^

O valor prognóstico e a história natural desses pacientes não estão bem estabelecidos, uma vez que não encontramos, na literatura, estudos com número de pacientes adequado, bem como seguimento de longo prazo que permitam estabelecer estratégias de seguimento clínico ou de terapêuticas preventivas. O momento e o grau de conversão para o fenótipo positivo dependem de vários fatores, tais como: diferentes tipos de mutação, raça e genero.^107 , 108^

Jensen e colaboradores^109^ demonstraram em um seguimento de 12 anos de 36 pacientes < 18 anos com G+/F-, 6% desenvolveram fenótipo de CMH entre 26 e 28 anos, sem apresentar eventos cardiovasculares ou morte durante esse período. Resultados semelhantes foram observados por Vermeer et al.,^110^ em 119 crianças com idade média de 12 anos, das quais, durante o seguimento de 6,9 a 3,8 anos, somente 5% desenvolveram CMH, e nenhuma apresentou evento cardiovascular nesse período. Em indivíduos mais jovens G+/F-, com idade < 12 anos, num seguimento de 6 anos, 15,3% desenvolveram CMH, sendo 52% antes dos 10 anos de idade.^111^

Em estudo multicêntrico internacional conduzido por Maurizi et al.,^112^ 203 indivíduos G+/F- com idade média de 32±11 anos, sendo 20% com idade > 50 anos, foram seguidos por 6±2 anos, com intervalos de 1 a 5 anos, nas avaliações clínicas, ECG e ecocardiograma. Cerca de 10% dos pacientes apresentaram conversão para F+, com uma taxa de 0,3%/ano e com uma frequência similar, independentemente da faixa etária, com evolução benigna e sem apresentar eventos cardiovasculares relacionados a CMH.

Esses estudos suportam o racional para a recomendação do seguimento clínico e por imagem a cada 1 a 2 anos até a idade de adulto jovem em crianças e adolescentes em decorrência do maior grau de penetração da mutação nessas faixas etárias. Em pacientes adultos, o seguimento é sugerido a cada 3 a 5 anos, pela menor taxa de conversão em decorrência a menor grau de penetração da mutação dos indivíduos que chegam a essa idade com F-. O prognóstico dos indivíduos G+/F- tem se mostrado benigno, independentemente da faixa etária, com baixa morbimortalidade e raramente eventos de morte súbita. Portanto não há restrições quanto à participação em esportes competitivos ou recomendações para a monitorização com ECG de 24 horas ou teste ergométrico na avaliação de risco de morte súbita. A utilização de terapêutica farmacológica preventiva e indicação de CDI como prevenção primária também não apresentam recomendações. Os pacientes com história familiar positiva para morte súbita podem ter uma avaliação particularizada, embora não haja estudos comprovando o maior risco nesses pacientes com G+/F.^2^

A Tabela 11 contempla as recomendações para o acompanhamento dos indivíduos genótipo-positivo e fenótipo-negativo na CMH.

**Tabela 11:** Recomendações para acompanhamento dos indivíduos genótipo-positivo e fenótipo-negativo na CMH

Recomendação	Classe de recomendação	Nível de evidência
Recomenda-se seguimento clínico, ECG, e imagem cardíaca (ETT) a cada 1 a 2 anos em crianças e adolescentes até os 30 anos de idade; e RMC em casos duvidosos.	I	C
Recomenda-se seguimento clínico, ECG, e imagem cardíaca (ETT) a cada 3 a 5 anos a partir da idade adulta; e RMC em casos duvidosos.	I	C
A liberação para participação em esportes competitivos de qualquer intensidade deve ser considerada em indivíduos sem história familiar de morte súbita.	IIa	C
A liberação para participação em esportes competitivos de qualquer intensidade pode ser considerada, com avaliação prévia por teste ergométrico e ECG de 24 horas, a cada 1 a 2 anos, em indivíduos com história familiar de morte súbita.	IIb	C
O CDI ou a terapêutica farmacológica para prevenção primária de morte súbita não são recomendados.	III	C

ETT: ecocardiograma transtorácico; RMC: ressonância magnética cardiovascular; ECG: eletrocardiograma; CDI: cardiodesfibrilador implantável.

#### 3.7.4. Fenocópias da CMH Sarcomérica

Fenocópias se caracterizam por apresentarem fenótipos indistinguíveis da CMH sarcomérica, com achados de exames de imagem semelhantes, portanto, com diagnóstico diferencial desafiador. Apesar de o fenótipo ser indistinguível da CMH, a magnitude e a distribuição do aumento de espessura da parede podem ser similares à CMH causada por variantes sarcoméricas, e essas doenças têm diferentes mecanismos fisiopatológicos, história natural e estratégias terapêuticas, algumas, inclusive, com modificação da evolução natural da doença quando o tratamento é instituído precocemente.^113 , 114^ O contexto clínico é de fundamental importância para o diagnóstico dessas fenocópias, levando-se em consideração dados demográficos, exames laboratoriais, evolução clínica e ECG. Os métodos de imagem podem, isoladamente, levantar a suspeita, como também podem dar suporte à suspeita diagnóstica e guiar o rastreio subsequente.^73 , 115 , 116^ Fatores que podem ser úteis no diagnóstico diferencial incluem: idade do início da HVE, modo de herança, manifestações extracardíacas e alterações dos exames complementares.

#### 3.7.5. Idade

**Figura 10:**
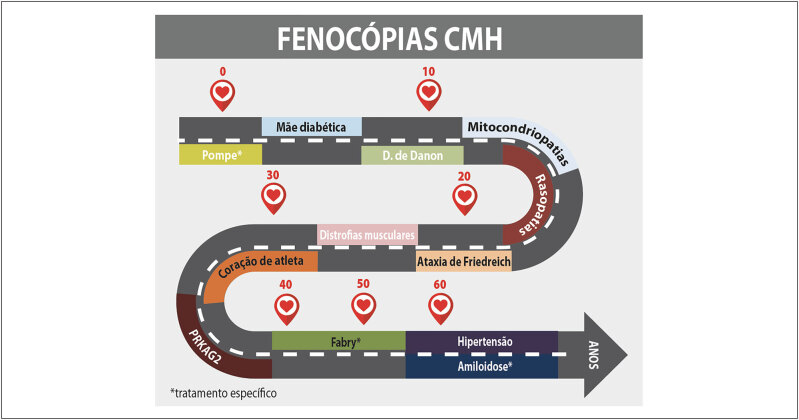
Etiologias da HVE relacionadas à faixa etária de início de apresentação.

A idade no diagnóstico ou nos primeiros sintomas é um importante sinalizador da etiologia e o primeiro fator a ser levado em consideração para o diagnóstico diferencial entre a CMH e doenças específicas. No período neonatal, as etiologias mais prevalentes além da CMH são a doença de Pompe, recém-nascido de mãe diabética e as mitocondriopatias. Em crianças dos 0 aos 20 anos: doença de Danon, RASopatias como a síndrome de Noonan e a síndrome de Leopard. Na adolescência: ataxia de Friedreich e distrofias musculares. Entre 20 e 30 anos: PRKAG2, coração de atleta. Aos 40 anos: doença de Fabry. Após os 60 anos, as etiologias mais comuns são amiloidose e hipertensão arterial^73^ ( Figura 10 ).

#### 3.7.6. História Familiar

Quando a etiologia é genética, a realização do heredograma de três gerações pode ser útil no diagnóstico diferencial, já que algumas doenças que cursam com HVE têm a transmissão ligada ao cromossomo X, como a doença de Fabry e a doença de Danon, portanto, podem ser excluídas se um familiar masculino acometido tiver seu pai também acometido.^73 , 115 , 116^ Esse fato pode levar a se considerar outra hipótese diagnóstica, como a síndrome de PRKAG2, que tem quadro clínico semelhante à doença de Fabry, mas tem transmissão genética autossômica dominante. O modo de transmissão também tem relevância no que diz respeito à magnitude da expressão clínica, maior nos pacientes masculinos quando é ligada ao cromossomo X, com implicações terapêuticas.^73 , 115 , 116^

#### 3.7.7. Manifestações Extracardíacas

**Figura 11:**
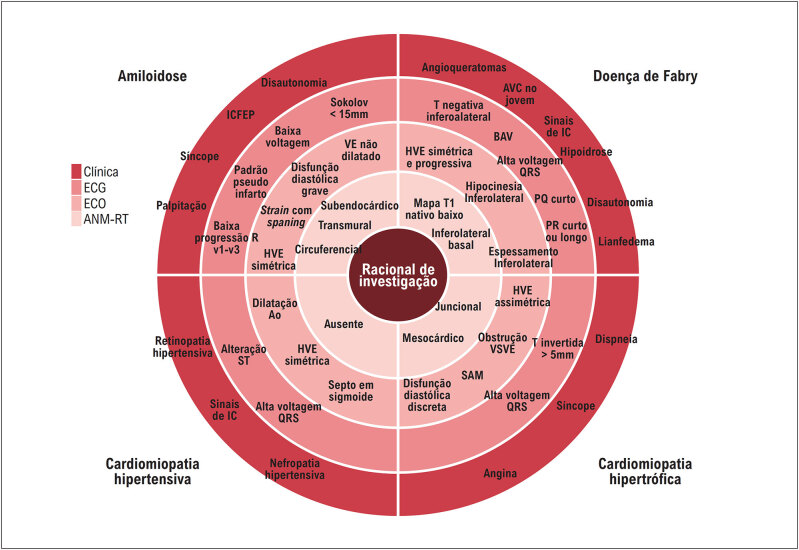
Quadro comparativo entre as principais fenocópias de CMH. Bruscky, LVR diagnóstico diferencial das cardiomiopatias que cursam com hipertrofia ventricular. ECG: eletrocardiograma; ECO: ecocardiograma; RNM-RT: ressonância magnética com realce tardio. ICFEP: insuficiência cardíaca com fração de ejeção preservada; AVC: acidente vascular cerebral; IC: insuficiência cardíaca; BAV: bloqueio atrioventricular; VE: ventrículo esquerdo; HVE: hipertrofia ventricular esquerda; VSVE: via de saída do ventrículo esquerdo; MSA: movimento sistólico anterior. Reproduzida a partir de Bruscky et al.^116^

Alterações de exames laboratoriais podem ser reveladoras de certas patologias, como a elevação dos níveis séricos de CPK nas distrofias musculares, na doença de Pompe e doença de Danon, elevações de TGO/TGP na doença de Danon. Outras manifestações clínicas também podem indicar etiologias específicas, como alteração da marcha e desequilíbrio na ataxia de Friedereich, déficit de cognição na doença de Danon, angioqueratomas na doença de Fabry, lentigos, surdez e baixa estatura na síndrome de Leopard, macroglossia, púrpura periorbitária, polineuropatia, alterações ortopédicas como síndrome do túnel do carpo bilateral, rotura espontânea do tendão do bíceps e estenose lombar espinhal na amiloidose; fraqueza muscular nas distrofias, retinopatia hipertensiva na hipertensão arterial e córnea verticilata na doença de Fabry^73 , 116^ ( Figura 11 ).

### 3.8. Eletrocardiograma

O ECG pode ser decisivo no diagnóstico diferencial das cardiopatias que cursam com hipertrofia ventricular. Podem preceder as alterações estruturais do coração, incluindo HVE, e podem ser a única manifestação da doença miocárdica. Quando o intervalo PR é curto (inferior a 120 ms), deve-se suspeitar de doença de Fabry. Outras alterações do ECG que sugerem doença de Fabry incluem infradesnivelamento do ST em parede inferior, alargamento do QRS, bloqueio completo ou incompleto do ramo direito e onda r em AVL maior que 11 mm.^117^ A presença de pré-excitação é indicativa de doença de Danon e PRKAG2, mas também pode estar presente na doença de Pompe.^73^ Alterações sugestivas de amiloidose incluem: desproporção entre a voltagem do QRS no ECG e o aumento de espessura de parede, no ecocardiograma, padrão de pseudoinfarto, diminuição de progressão de onda r de V1 para V3, baixa voltagem do QRS no plano frontal, presença de FA, bloqueios de ramo, bloqueios AV, índice de Sokolov < 15 mm.^118^

### 3.9. Ecocardiograma

Desproporção da voltagem do QRS e grau de aumento simétrico de espessura no ecocardiograma, derrame pericárdico discreto, espessamento do septo interatrial e interventricular, aspecto refringente do septo e *strain* reduzido com padrão de "cereja no bolo" são achados indicativos de amiloidose.^119^

### 3.10. Ressonância Magnética Cardíaca

**Figura 12:**
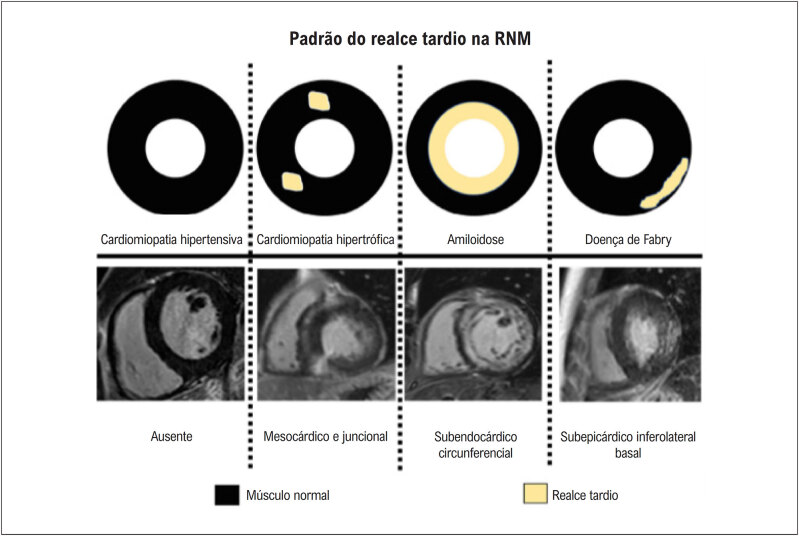
Tipos de realce tardio e sua localização nos diferentes tipos de CMH.^116^ RNM: ressonância magnética.

A RMC pode complementar os achados estruturais do ecocardiograma e contribui para o diagnóstico diferencial pela localização e intensidade do realce tardio, bem como os mapas T1 e T2. O realce tardio tem padrões típicos em algumas doenças que cursam com HVE, mas não em todas, nem sempre está presente e não há uma especificidade comprovada. Na cardiopatia hipertensiva, ou está ausente ou é muito discreto e sem padrão típico. Na CMH, costuma ser mesocárdico em pontos isolados, geralmente nos locais de maior hipertrofia, particularmente nas junções do septo interventricular e da parede livre do ventrículo direito. Na amiloidose, o realce tardio característico é subendocárdico circunferencial, mas, nas fases iniciais, pode não estar presente e, na fase tardia, pode ser ou dificuldade de se obter o sinal. Na Doença de Fabry, o realce tardio é mesocárdico na parede inferolateral basal e média. Nessa doença, é muito característico o mapa T1 baixo, diferente de todas as outras doenças que cursam com HVE ( Figura 12 ).^73^

### 3.11. Principais Fenocópias da CMH

#### 3.11.1. Amiloidose Cardíaca

A amiloidose cardíaca é causada por depósitos extracelulares de amiloide no miocárdio e pode ser decorrente de depósitos de imunoglobulinas (amiloidose AL) ou por depósito de transtirretina (ATTR). O envolvimento cardíaco piora significativamente o prognóstico na amiloidose, e os sintomas incluem angina, IC, arritmias e morte súbita. A condição também é caracterizada por envolvimento multissistêmico, como síndrome do túnel do carpo, facilidade de hematomas, macroglossia, neuropatia e hepatomegalia. O ECG mostra tipicamente complexos QRS de baixa tensão. A ecocardiografia mostra hipertrofia biventricular com espessamento da valva, dilatação biatrial e disfunção diastólica. Técnicas especiais de ecocardiografia, como a imagem por deformação e a taxa de deformação, derivadas do rastreamento de *speckle* , podem ajudar a distingui-la da CMH. Enquanto o padrão de HVE costuma ser concêntrico e não obstrutivo na amiloidose cardíaca, formas obstrutivas assimétricas também foram descritas, imitando, assim, a CMH.^120^ A RMC mostrando realce tardio subendocárdio é patognomônica da amiloidose cardíaca e também prediz o prognóstico da condição. O tratamento da amiloidose cardíaca é geralmente de suporte, devido ao pobre prognóstico da condição.^121^

#### 3.11.2. Citopatias Mitocondriais

Esse é um grupo heterogêneo de distúrbios causados por mutações do genoma mitocondrial materno ou em genes nucleares responsáveis por codificar proteínas mitocondriais e que leva à produção disfuncional de energia e ao envolvimento multissistêmico (particularmente sistema nervoso central, coração e sistema esquelético). Pessoas afetadas podem apresentar sintomas a qualquer momento da infância à idade adulta.

Existem diversos tipos de citopatias mitocondriais. Alguns dos sintomas comuns desses distúrbios que podem aumentar a suspeita diagnóstica incluem: miopatia, mialgia, fadiga não explicada por disfunção miocárdica, oftalmoplegia, perda auditiva e déficits de desenvolvimento psicomotor.

A hipertrofia miocárdica é a forma mais comum de acometimento cardíaco; no entanto, as cardiomiopatias mitocondriais também podem se apresentar como cardiomiopatias dilatadas, restritivas e não compactadas do VE.^122^ Tipicamente, encontra-se uma miocardiopatia não obstrutiva com hipertrofia concêntrica discreta em cerca de 1/4 dos pacientes com doença mitocondrial, o que representa 50% das manifestações cardíacas em pessoas com cardiomiopatias mitocondriais. Ela pode ser detectada ainda no período antenatal e pode ser a única manifestação de uma doença mitocondrial ou parte de uma doença multissistêmica. Uma hipertrofia obstrutiva ocorre raramente, mas, quando ocorre a presença de hipertrofia cardíaca, esta frequentemente evolui para disfunção sistólica, seguida de dilatação do VE. A cardiomiopatia dilatada, que pode ser primária ou secundária após a hipertrofia ventricular, ocorre menos frequentemente, enquanto a cardiomiopatia restritiva é uma manifestação rara de doenças mitocondriais.^123^ Embora a cardiomiopatia não compactada do VE também seja um achado raro em doenças mitocondriais, entre indivíduos com não compactação, as doenças mitocondriais são altamente prevalentes. A cardiomiopatia não compactada do VE é mais frequente em homens e tende a se desenvolver durante a gravidez em mulheres. Ocasionalmente, pode desaparecer durante o curso da doença em alguns indivíduos com doenças mitocondriais.

Em caso de suspeita diagnóstica, a avaliação inicial para a doença mitocondrial deve incluir a análise do sangue para contagem completa de glóbulos vermelhos, CPK, transaminases, albumina, lactato e piruvato, aminoácidos e acilcarnitinas, junto com ácidos orgânicos quantitativos ou qualitativos na urina.

As doenças mitocondriais são causadas por mutações no mtDNA materno ou em um dos muitos genes que codificam proteínas mitocondriais, mas que se localizam no genoma nuclear. A sequência do genoma mtDNA e a análise de heteroplasmia podem, agora, ser realizadas no sangue. No geral, o advento de tecnologias de sequenciamento de nova geração (NGS) tornou-se a metodologia padrão-ouro para a sequenciamento do genoma mtDNA. Dessa maneira, o teste genético inicial para avaliar doença mitocondrial no sangue, na urina ou em tecidos específicos, dependendo da apresentação dos sintomas e da disponibilidade da amostra, deve ser o sequenciamento completo do genoma mitocondrial. No caso de alta suspeita clínica, mas teste genético negativo, o sequenciamento do genoma codificante nuclear através de análise de exoma é indicado. A descrição de mutação no genoma nuclear causando doença mitocondrial deve ser especialmente avaliada para casos de diagnóstico na idade adulta.^124^

A identificação de uma mutação causadora de doença mitocondrial permite que as famílias encerrem sua jornada diagnóstica e recebam orientação genética apropriada, teste de portador e diagnóstico pré-natal seletivo.

#### 3.11.3. Síndrome de Barth

A síndrome de Barth é uma doença ligada ao cromossomo X, caracterizada por cardiomiopatia, miopatia esquelética, retardo do crescimento, neutropenia e níveis aumentados de ácido 3-metilglutacônico na urina. Ela é causada por mutações no gene TAZ que codifica para a tafazina, uma transacilase de fosfolipídios localizada na membrana interna mitocondrial e que desempenha um papel importante na remodelação da cardiolipina. As cardiomiopatias são comumente cardiomiopatias não compactadas do VE e dilatadas, enquanto a CMH parece ser menos comum. Outras manifestações cardíacas da síndrome de Barth são arritmias (incluindo taquicardia supraventricular e ventricular) e morte súbita.^125^

Outras doenças nesse grupo que podem estar associadas à cardiomiopatia são causadas por mutações em DNAJC19, TMEM70 e AGK. A acidúria de 3-metilglutaconico associada às mutações em DNAJC19 (síndrome de ataxia e cardiomiopatia dilatada) resulta da importação deficiente de proteínas mitocondriais e é caracterizada por cardiomiopatia dilatada ou não compactada do VE, ataxia cerebelar não progressiva, disgenesia testicular e falha de crescimento. As mutações em TMEM70 (deficiência do complexo V mitocondrial), que codificam uma proteína envolvida na inserção da ATP sintase (complexo V) na membrana mitocondrial, resultam em doença mitocondrial multissistêmica com CMH. A síndrome de Sengers, causada por mutações em AGK, também pode ser acompanhada por acidúria de 3-metilglutacônico e é caracterizada por CMH, catarata, miopatia, intolerância ao exercício e acidose lática. O produto do gene AGK é uma quinase de acilglicerol e está envolvido na montagem do ANT1, um transportador de nucleotídeos adenínicos mitocondrial.

#### 3.11.4. Ataxia de Friedreich

A ataxia de Friedreich é uma doença neurodegenerativa autossômica recessiva causada por mutações no gene FXN, que codifica a frataxina, uma proteína mitocondrial de ligação de ferro envolvida na síntese dos complexos Fe-S necessários para a cadeia de transporte de elétrons. A apresentação clínica inclui ataxia progressiva após os anos da adolescência, disartria, perda de reflexos nas pernas, neuropatia sensorial periférica e diabetes mellitus. A manifestação cardíaca mais comum é a hipertrofia de VE.^126^

A identificação da ataxia de Friedreich como a causa de hipertrofia ventricular depende da identificação de manifestações extracardíacas, que incluem ataxia e fraqueza progressivas nos membros inferiores, disartria, nistagmo e perda da propriocepção e também podem incluir escoliose, diabetes mellitus, visão e audição prejudicadas. A maioria dos pacientes perderá progressivamente a capacidade de andar. O envolvimento cardíaco é raramente encontrado em outras ataxias hereditárias. No entanto, o fenótipo é altamente variável e, em alguns pacientes, a primeira manifestação é a cardiomiopatia.

Do ponto de vista cardiovascular, existe uma série de manifestações fenotípicas, incluindo hipertrofia concêntrica/assimétrica ou cardiomiopatia dilatada. A hipertrofia concêntrica/assimétrica é menos comum, mas a cardiomiopatia dilatada com arritmia é mais frequentemente associada à mortalidade. A função sistólica dos pacientes com ataxia de Friedreich pode mostrar queda no final da vida. No entanto, há poucos dados de estudos prospectivos a longo prazo da progressão cardíaca nesses pacientes, e a causa da morte é frequentemente atribuída a IC e arritmia.^127^

Achados ecocardiográficos incluem uma aparência granular semelhante à vista na amiloidose, embora sem efusão pericárdica ou aumento biatrial. Estudos de RMC detectam fibrose do VE e remodelamento concêntrico mesmo antes da hipertrofia, juntamente com uma reserva diminuída de perfusão miocárdica.

#### 3.11.5. Doenças de Depósito de Glicogênio

Algumas características importantes podem ajudar a distinguir essas condições da HVE com defeitos genéticos sarcoméricos. Esses incluem evidências de características clínicas multissistêmicas em idade precoce, HVE extrema (estimulada por vacúolos preenchidos com glicogênio e não pelo desarranjo sarcomérico ou pela fibrose celular característica da HVE sarcomérica), progressão precoce para miocardiopatia dilatada e anormalidades eletrocardiográficas, como pré-excitação ventricular e defeitos do sistema de condução.^128^ A pré-excitação ventricular nessas condições tem sido considerada como consequência de mecanismo único de disrupção do anel fibroso por células musculares preenchidas com glicogênio, e não pela presença de trajetos distintos histologicamente. As características típicas de cada uma das condições são detalhadas a seguir.

Atualmente, reconhece-se que quatro cardiomiopatias metabólicas de armazenamento, PRKAG2, LAMP2 (doença de Danon), doença de Pompe e doença de Forbes, apresentam um fenótipo clínico de HVE que imita a expressão da doença da HVE "típica" sarcomérica e funcionam como fenocópias da CMH. Embora essas miocardiopatias metabólicas componham apenas uma pequena fração dos pacientes adultos com HVE não explicada (< 1%), a distinção diagnóstica da CMH é crucial, pois a história natural, o prognóstico e, em alguns casos, as estratégias de tratamento são marcadamente diferentes para essas fenocópias.

O padrão de herança da cardiomiopatia causada por mutações em PRKAG2 é caracteristicamente de doença autossômica dominante. A expressão morfológica dos quadros é geralmente um padrão simétrico de HVE, em que a espessura da parede é amplamente similar em todos os segmentos da câmara, mas geralmente é massiva (> 30 mm), na ausência de obstrução do trato de saída. Ainda, a alteração está associada a um espectro de anormalidades de condução eletrocardiográfica (ECG), mais comumente pré-excitação ventricular (frequentemente diagnosticada como Wolff-Parkinson-White – WPF) e bloqueio de ramo. Desfechos relacionados à doença ocorrem tipicamente na terceira e quarta década, mais comumente fibrilação/ *flutter* atrial ou doença de condução que pode necessitar de implante de marca-passo. O risco de morte súbita e de progressão para disfunção sistólica importante parece ser semelhante ao da CMH, o que dificulta o diagnóstico diferencial.^129^

A deficiência de proteína de membrana associada a lisossomos 2 (LAMP2) (GSD IIb) é conhecida como doença de Danon, afeta principalmente o músculo cardíaco e esquelético e também inclui manifestações neurológicas. A doença é caracterizada por miocardiopatia grave, miopatia esquelética leve, anormalidades oftalmológicas e diferentes graus de incapacidade intelectual e sintomas psiquiátricos. A idade de início varia desde a infância até a idade adulta. As portadoras femininas geralmente têm um início mais tardio da doença e apresentam uma menor incidência de incapacidade intelectual. A miocardiopatia pode ser a manifestação inicial, geralmente é de forma hipertrófica, mas também pode ser dilatada, e as arritmias cardíacas, devido em parte à fibrose progressiva, são uma causa comum de morte súbita.^130^

Mesmo na ausência de fraqueza muscular, a miopatia sutil é quase sempre presente em homens, manifestando-se com CPK persistentemente elevada. Pacientes do sexo masculino, muitas vezes, passam por investigações repetidas em clínicas de gastroenterologia devido à elevação de transaminases, sem outra indicação de doença hepática. As manifestações neuropsiquiátricas variam de retardo mental ou psicose a até anormalidades comportamentais menores, transtorno de déficit de atenção, transtorno de ansiedade ou podem ser totalmente ausentes. A retinopatia pigmentar é comum, mas raramente leva a distúrbios visuais clinicamente manifestos.

A suspeita clínica da condição deve motivar o teste de níveis de CPK e enzimas hepáticas no soro, ambos os quais geralmente estão elevados nessa condição. O ECG pode mostrar alterações do tipo WPW (intervalo PR curto e pré-excitação) em até 2/3 dos homens e menos de 1/3 das mulheres, e essa descoberta, juntamente com complexos de voltagem muito grandes, especialmente entre adolescentes, também deve suscitar suspeita da condição. A biópsia muscular esquelética mostra vacúolos positivos para ácido periódico-Schiff intrassarcolema. Enquanto a ecocardiografia geralmente mostra HVE grave e concêntrica, a hipertrofia septal assimétrica também pode ter sido observada em alguns pacientes, semelhante ao padrão visto na HVE sarcomérica, e, nos estágios finais, as alterações de miocardiopatia dilatada predominam. O rastreamento genético molecular revelando mutação do gene LAMP2 confirma o diagnóstico. Não há terapia médica específica disponível para a condição. Deve ser considerado o implante de CDI, embora a hipertrofia grave esteja associada a limiares de desfibrilação altos e falha em terminar a fibrilação ventricular.

#### 3.11.6. Cardiomiopatia por Depósito de Oxalato

A cardiomiopatia por depósito de oxalato é uma causa extremamente rara de HVE, consistindo em depósitos extracelulares de oxalato, devido a uma hiperprodução causada por uma deficiência da enzima alanina-glicoxilato aminotransferase no fígado. Faz parte das chamadas hiperoxalúrias primárias, distúrbios autossômicos recessivos do metabolismo do oxalato. A IC como resultado de disfunção sistólica e cardiomiopatia restritiva, com HVE em 1/3 dos pacientes, juntamente com distúrbios de ritmo, são as características cardíacas encontradas nessa entidade. Litíase urinária, nefrocalcinose e insuficiência renal progressiva completam o quadro dessa síndrome.^131^

#### 3.11.7. Doença de Fabry

A doença de Fabry [também conhecida como doença de Anderson-Fabry (AFD) ou *angioceratoma corporis diffusum* ] é uma doença rara do metabolismo da α-galactosidase A, uma enzima lisossômica que degrada glicoesfingolipídeos neutros, principalmente a globotriaosilceramida (Gb3). Alterações na enzima resultam em progressivo acúmulo intracelular de lípides.

O acometimento cardíaco está presente em todas as formas da doença de Fabry, com a HVE concêntrica sendo a apresentação mais comum.^132^ A proporção de pacientes com doença de Fabry entre aqueles previamente diagnosticados com CMH varia de 0,5% a 12%, dependendo das séries relatadas.^133^

O ECG na doença de Fabry é uma ferramenta muito útil para sugerir o diagnóstico (critérios de HVE e intervalo PR curto) e também se mostra útil na estratificação de risco, como com a presença de FA e doença do sistema de condução, principalmente bloqueio atrioventricular (BAV) completo.^117^ O intervalo PR curto, que pode estar presente em crianças menores de 10 anos de idade, é visto em cerca de 40% dos pacientes. Interessantemente, não é devido a uma via acessória (ou como o sugerido na cardiomiopatia por PRKAG2), mas à condução acelerada do nó atrioventricular.^134^ Alguns estudos relatam incidência de fibrilação atrial (FA) em monitoramento ambulatorial de 17%.^135^ Também podem estar presentes arritmias ventriculares em 8% dos casos.^136^ Alguns pacientes com doença de Fabry relatam angina. Isso é mais comumente visto em combinação com HVE e pode ser devido a um aumento na demanda de oxigênio por motivos semelhantes aos que ocorrem na CMH, mas também em resposta à arteriopatia difusa associada ao dano celular das paredes arteriais. Por exemplo, a prevalência da dilatação da aorta aumenta com a idade, é mais prevalente em homens e geralmente afeta o sínus de Valsalva e a aorta ascendente.^137^

A importância na identificação da doença de Fabry reside na existência de terapia específica para a doença.^138^

#### 3.11.8. Síndrome de Noonan e outras RASopatias

As RASopatias são um grupo de doenças multissistêmicas causadas por mutações germinativas em genes que codificam transdutores de sinal e proteínas reguladoras ligadas funcionalmente à via RAS/proteinase ativada por mitógenos (MAPK). Coletivamente, esses distúrbios têm uma prevalência estimada de 1 em 1.000 a 1 em 2.500 entre nascimentos vivos. De acordo com um grande registro clínico, as RASopatias podem representar o diagnóstico subjacente em cerca de 18% da CMH na infância, especialmente entre bebês com menos de 1 ano, em que elas representam cerca de 42% dos casos.^126^

Doenças que fazem parte das RASopatias incluem a síndrome de Noonan (SN), a SN com múltiplos lentigos (NSML, anteriormente conhecida como síndrome LEOPARD), a síndrome cardiocutâneofacial (CFCS), a síndrome de Mazzanti (também conhecida como transtorno semelhante à SN com cabelo anágeno solto), a síndrome de Costello (CS), a neurofibromatose tipo 1 e a síndrome de Legius, que são bem reconhecidas e caracterizadas clinicamente; no entanto, outras condições relacionadas clinicamente estão surgindo.

Aparentemente, a extensão da variabilidade clínica caracterizando cada RASopatia está relacionada à extensão da variabilidade molecular e à heterogeneidade dessas doenças. Por exemplo, a SN, que é a doença mais comum entre as RASopatias e é causada por mutações em mais de 10 genes (como PTPN11, SOS1, SOS2, NRAS, KRAS, MRAS, RRAS2, RIT1, LZTR1, RAF1, MAP2K1), que são preferencialmente associados a determinadas características, incluindo o crescimento adequado e a cognição (SOS1, SOS2), alta prevalência de estenose pulmonar (PS) (PTPN11) ou CMH (por exemplo, RAF1, MRAS e RIT1). Por outro lado, outras RASopatias são relativamente homogêneas, sendo causadas por um espectro estreito de mutações em um único gene, como no caso da CS e da síndrome de Mazzanti, que são causadas por um conjunto de mutações em HRAS e SHOC2, respectivamente.^139^

A hipertrofia miocárdica nas RASopatias é mais prevalente durante a infância, quando comparada à CMH. Em 5 a 10% dos casos, a hipertrofia nas RASopatias está associada a uma apresentação clínica grave, especialmente para bebês com sinais de IC, com uma taxa de mortalidade de 70% em um ano.^140^ Com exceção desses casos, o estado clínico tende a melhorar com o tempo, e a progressão da HVE, descrita nas RASopatias, parece incomum. Pelo contrário, foi relatado um processo de remodelamento inverso do VE com regressão dos valores-z das medidas da espessura da parede miocárdica ao longo do tempo em exames ecocardiográficos seriados em muitos estudos clínicos.^141^

## 4. Tratamento

**Figura 13:**
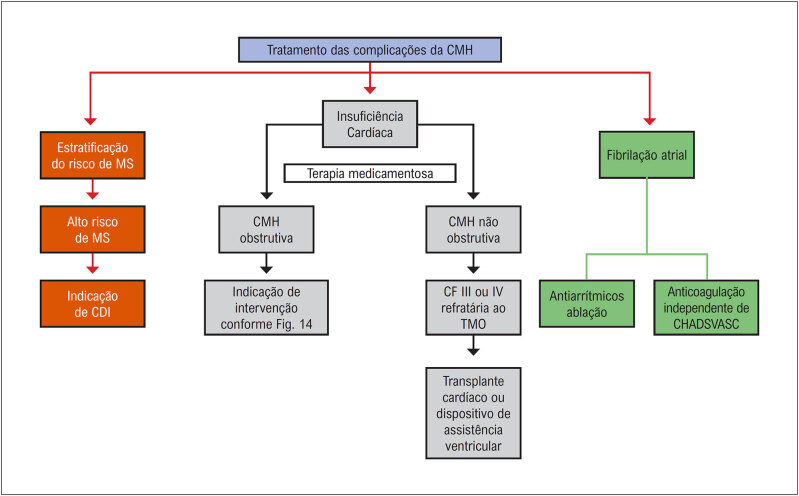
Delineamento geral das opções de tratamento das complicações da CMH. CMH: cardiomiopatia hipertrófica; MS: morte súbita; CDI: cardiodesfibrilador implantável; TMO: tratamento médico otimizado.

Dentro da história natural da CMH, as opções de tratamento e seguimento clínico podem variar de acordo com a forma de apresentação individual de cada paciente. Uma visão geral das opções está ilustrada na Figura 13 .

Os pacientes portadores de mutações, familiares de pacientes com CMH considerados genótipo positivo e fenótipo negativo devem ser acompanhados periodicamente, e essa condição é abordada em um tópico específico desta diretriz.

Os indivíduos que manifestam o fenótipo de CMH podem ser diferenciados naqueles com evolução assintomática e benigna e com baixo risco de morte súbita, que correspondem, grosso modo, a 50% desses pacientes, em que tratamentos medicamentosos ou intervenções não são necessários. Eles devem submeter-se à avaliação periódica de estratificação de risco e à avaliação de progresso da doença.

**Figura 14:**
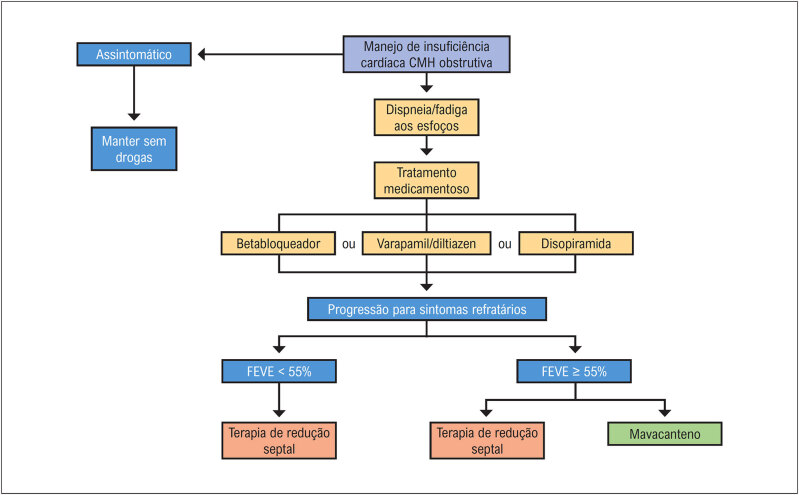
Delineamento das opções de tratamento da insuficiência cardíaca na CMH obstrutiva. CMH: cardiomiopatia hipertrófica; FEVE: fração de ejeção ventricular esquerda.

O curso clínico com complicações ocorre em cerca de 40 a 50% dos pacientes com CMH clinicamente manifesta, podendo ocorrer risco aumentado de morte súbita, sintomas de IC e FA. As opções de terapia para essas condições, resumidas nas Figuras 13 , 14 e 15 , serão abordadas nos tópicos que se seguem.

**Figura 15:**
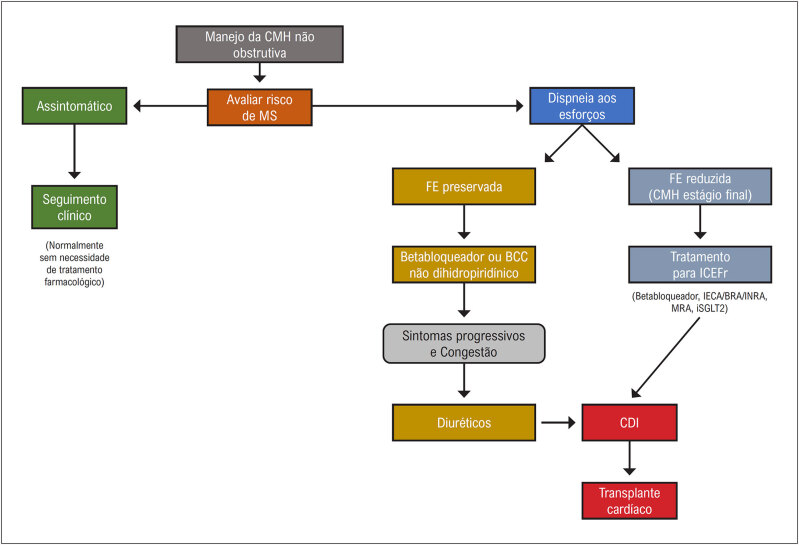
Delineamento das opções de tratamento da insuficiência cardíaca na CMH não obstrutiva. CMH: cardiomiopatia hipertrófica; MS: morte súbita; FE: fração de ejeção; BCC: bloqueador dos canais de cálcio; ICFEr: insuficiência cardíaca com fração de ejeção reduzida; IECA: inibidores da enzima de conversão da angiotensina II; BRA: bloqueador do receptor da angiotensina; INRA: inibidor da neprilisina e do receptor da angiotensina; MRA: antagonista do receptor mineralocorticoide; iSGLT2: inibidores do cotransportador sódio-glicose 2; CDI: cardiodesfibrilador implantável.

### 4.1. Tratamento Medicamentoso

O tratamento farmacológico é a primeira opção entre pacientes com CMH sintomática, seja na forma obstrutiva ou não obstrutiva. Não existe evidência clara do seu papel na redução da mortalidade nessa população. Entretanto, a terapia farmacológica otimizada é capaz de melhorar significativamente os sintomas e reduzir a necessidade de procedimentos de terapia de redução septal na forma obstrutiva. Discutimos as principais classes de fármacos indicados nesse tratamento.

#### 4.1.1. Betabloqueadores

Betabloqueadores são indicados para alívio sintomático (por exemplo: angina, dispneia) em indivíduos com CMH. Essa indicação se faz presente em ambas as formas, obstrutivas ou não obstrutivas.

A indicação de betabloqueadores nesse contexto é baseada nos efeitos benéficos esperados associados ao bloqueio beta-adrenérgico, como aumento do tempo diastólico, melhora da perfusão coronariana, redução do consumo miocárdico de oxigênio, redução de risco de taquiarritmias e, consequentemente, redução do risco de morte súbita. Há um entendimento de que os efeitos inotrópicos da estimulação catecolaminérgica produzem um aumento nos gradientes de obstrução da VSVE, e esse conhecimento desencadeou uma tentativa de tratar a obstrução do TSVE com bloqueio dos receptores beta-adrenérgicos.^142 , 143^

Apenas muito recentemente tivemos um ensaio clínico randomizado, cruzado, com 29 pacientes, comparando metoprolol com placebo que evidenciou que o metoprolol reduziu a obstrução da VSVE em repouso e durante o exercício, proporcionou alívio dos sintomas e melhorou a qualidade de vida em pacientes com CMH obstrutiva.^144^ A despeito de um nível de evidências ainda incipiente, é consensual entre especialistas que betabloqueadores devem ser usados como base do tratamento clínico da CMH.

##### 4.1.1.1. Bloqueadores dos Canais do Cálcio Não Dihidropiridínicos

Os bloqueadores dos canais do cálcio não dihidropiridínicos (BCCND), verapamil e diltiazem, podem proporcionar alívio dos sintomas em pacientes com CMH obstrutiva. Ambos os agentes possuem propriedades vasodilatadoras, além de efeito inotrópico e cronotrópico negativos, sendo listados como terapia sintomática para CMH em diversas diretrizes.^2 , 3^ O verapamil (dose inicial de 40 mg, três vezes ao dia, até um máximo de 480 mg por dia) é o mais estudado e pode ser indicado quando os ß-bloqueadores são contraindicados ou ineficazes, mas um monitoramento rigoroso é necessário em pacientes com obstrução grave (≥ 100 mmHg) ou elevação sistólica da pressão da artéria pulmonar, pois pode provocar edema pulmonar.^3^

Os efeitos hemodinâmicos da administração intravenosa de verapamil foram examinados por Rosing et al.^145^ em 27 pacientes com CMH. O estudo observou que doses crescentes de verapamil produziram pequenos aumentos na frequência cardíaca (de 72 ± 3 para 81 ± 6 batimentos/min, p < 0,01) e uma redução considerável da pressão arterial sistólica (de 118 ± 8 para 99 ± 5 mmHg, p < 0,005). Uma dose elevada (0,021 mg/kg/min) diminuiu o gradiente basal da VSVE (de 94 ± 14 a 49 ± 14 mmHg, p < 0,05) e o gradiente médio da VSVE durante a manobra de Valsalva (de 76 ± 5 a 63 ± 13 mmHg, p < 0,01).

Os efeitos clínicos do verapamil a longo prazo foram demonstrados em estudo observacional com 78 participantes. Do total, houve melhora dos sintomas em 54% dos pacientes. A capacidade funcional avaliada por teste ergométrico demonstrou um aumento do tempo de exercício depois de 5 dias do início da terapia em 3,1 ± 0,6 minutos. Um aumento adicional de 2,3 ± 0,6 minutos (25 ± 7%, p < 0,0025) sobre o valor inicial com verapamil foi registrado na última visita dos pacientes (mediana 12 meses após o início da terapia).^146^

Dos efeitos adversos dos BBCND na CMH, foram reportados episódios de colapso hemodinâmico em pacientes com gradientes elevados no TSVE em repouso (acima de 80 a 100 mmHg) e sintomas de IC submetidos ao verapamil, com relatos de bradicardia importante e baixo débito cardíaco.^147^

Dessa forma, apesar do nível reduzido de evidências, os BCCND (verapamil ou diltiazen) são recomendados para controle de sintomas em pacientes com CMH, obstrutivos ou não obstrutivos, em substituição ao betabloqueador, quando não tolerado ou ineficaz (Classe I, nível de evidência C).

#### 4.1.2. Drogas Inibidoras da Miosina Cardíaca

##### 4.1.2.1. Mavacanteno

O mavacanteno é o primeiro inibidor alostérico seletivo de pequena molécula da ATPase da miosina cardíaca que foi desenvolvido para diminuir as interações de ponte cruzada de actina-miosina, reduzindo, assim, a contratilidade cardíaca. Seu principal atributo clínico é a redução do gradiente de pressão de VSVE, com base nas suas propriedades inotrópicas negativas, semelhantes, nesse aspecto, à disopiramida.^148^ O ensaio clínico EXPLORER-HCM ( *Mavacamten for treatment of symptomatic obstructive hypertrophic cardiomyopathy* ) foi o estudo que avaliou o benefício clínico desse fármaco (titulado de 2,5 até 15 mg ao dia) de forma mais consistente. Trata-se de ensaio clínico randomizado controlado por placebo com duração de 30 semanas envolvendo 251 pacientes com CMH obstrutiva (gradiente em VSVE > 50 mmHg) e sintomáticos (classe funcional da NYHA de II-III), em uso de terapia de base com betabloqueadores e bloqueadores de canal de cálcio (> 90% dos indivíduos). O desfecho primário foi a melhora no consumo de pico de oxigênio durante exercício (VO_2_ pico) de 3,0 mL/kg por minuto sem piora na classe funcional da NYHA ou melhora do VO_2_ pico de 1,5 mL/kg por minuto e redução de pelo menos uma classe funcional da NYHA. O desfecho primário foi observado em 37% dos pacientes em uso de mavacanteno *versus* 17% dos pacientes do grupo placebo (p < 0,0001). O uso de mavacanteno também reduziu de forma significativa os gradientes intraventriculares após o exercício e melhorou a classe funcional dos pacientes. O mavacanteno demonstrou ser seguro e bem tolerado, com um perfil geral de eventos adversos comparável ao placebo.^149^ Um estudo subsequente avaliou o benefício do fármaco em escores de qualidade de vida [ *Kansas City Cardiomyopathy Questionnaire* (KCCQ)]. A proporção de pacientes com uma mudança acentuada (KCCQ- *Overall Summary* ≥ 20 pontos) foi de 36% (33 de 92) no grupo mavacanteno *versus* 15% (13 de 88) no grupo placebo, com uma diferença absoluta estimada de 21% [intervalo de confiança (IC) de 95% 8,8-33,4] e número necessário para tratar de cinco (IC95% 3-11). Esses ganhos retornaram à linha de base após a interrupção do tratamento.^150^ Um subestudo ecocardiográfico demonstrou que mais pacientes em uso de mavacanteno (80,9%), comparativamente ao uso de placebo (34,0%), apresentaram resolução do movimento anterior sistólico da válvula mitral após 30 semanas (p < 0,0001). O mavacanteno também melhorou parâmetros da função diastólica de VE, incluindo índice do volume atrial, a razão E/e’ lateral e níveis de pró-peptídeo tipo B N-terminal, achados que sugerem melhora em marcadores da fisiopatologia da MCH obstrutiva.^151^ Em outro subestudo, envolvendo apenas 35 pacientes (mavacanteno, n = 17; placebo, n = 18), dados de RMC demonstraram que os pacientes que receberam mavacamten apresentaram maior redução no índice de massa de VE [diferença média entre os grupos, -15,8 g/m^2^ (IC95% -22,6 a -9,0; p < 0,0001). Houve também redução mais acentuada da FE do VE com mavacanteno quando comparado com placebo [-6,6% (6,39%) *versus* –3,9% (7,7%), p = 0,002], mas não foram identificadas alterações significativas entre os grupos no realce tardio por gadolínio e no volume extracelular.^152^

O mavacanteno também foi estudado em 112 pacientes com CMH obstrutiva com indicação de terapia redutora septal baseada em recomendações de diretrizes vigentes no estudo VALOR-HCM. O desfecho primário foi composto pela proporção de pacientes que procedeu terapia redutora septal ou se manteve elegível para essa terapia após 16 semanas de tratamento. A maior parte dos pacientes arrolados estavam em classe funcional NYHA III-IV com gradiente médio em VSVE após exercício de 84 mmHg. Após 16 semanas, 43 de 56 pacientes no grupo placebo (77%) e 10 de 56 pacientes que receberam mavacanteno (18%) atingiram critérios de diretrizes ou foram submetidos a terapias redutoras (p < 0.0001).^153^ Em 02/01/2023, a ANVISA aprovou o uso de mavacanteno no Brasil, para pacientes com CMH obstrutiva com sintomas, classe funcional II e III da NYHA.

Essas evidências indicam que o mavacanteno deve ser considerado para a melhora dos sintomas em pacientes adultos com CMH obstrutiva, com FEVE ≥ 55%, exibindo gradiente intraventricular 50 mmHg em repouso ou sob provocação, sintomáticos em CF II ou III da NYHA, apesar do uso de betabloqueadores ou bloqueadores dos canais de cálcio não dihidropiridínicos em máximas doses toleradas, com grau de recomendação IIa nível de evidência B ( Tabela 12 ).

**Tabela 12:** Recomendações para o tratamento medicamentoso da CMH

Recomendação	Classe de recomendação	Nível de evidência
Recomenda-se o uso de betabloqueadores, em pacientes sintomáticos para a redução de sintomas nas formas obstrutivas ou não.	I	B
Recomenda-se o uso de bloqueadores dos canais do cálcio não dihidropiridínicos em pacientes intolerantes aos betabloqueadores ou quando os betabloqueadores forem ineficazes, nas formas obstrutivas ou não.	I	B
Deve ser considerado o uso de mavacanteno em pacientes adultos com forma obstrutiva, com gradiente intraventricular > 50 mmHg em repouso ou sob provocação, com FEVE > 55%, que persistam sintomáticos apesar do uso de betabloqueadores ou bloqueadores dos canais de cálcio não dihidropiridínicos em máximas doses toleradas, em CF II-III da NYHA, para redução de sintomas e do gradiente intraventricular.	IIa	B
Deve ser considerado o uso de bloqueadores dos canais do cálcio não dihidropiridínicos em associação aos betabloqueadores para controle de frequência cardíaca e melhora de sintomas.	IIa	C
Pode ser considerado o uso de bloqueadores do receptor AT1 da angiotensina-II para a melhora da capacidade física e controle da hipertensão arterial sistêmica.	IIb	B
Deve ser considerado evitar-se o uso de vasodilatadores, especialmente em pacientes com a forma obstrutiva.	IIa	C

FEVE: fração de ejeção ventricular esquerda.

Encontra-se em andamento o estudo clínico randomizado ODYSSEY-HCM, duplo-cego, controlado por placebo, para avaliar a segurança, tolerância e eficácia do mavacanteno em adultos com CMH não obstrutiva sintomática ( *Clinical trials* – Protocolo CV027031).

##### 4.1.2.2. Aficanteno

O aficanteno é o segundo inibidor da miosina cardíaca em avaliação em estudos clínicos. Ele tem uma vida mais curta em comparação com o mavacamten, atinge o estado de equilíbrio dentro de 2 semanas e parece ter uma janela terapêutica mais ampla. No estudo fase II randomizado e controlado por placebo REDWOOD-HCM (n = 41), doses elevadas de aficanteno (10-30 mg diariamente) tiveram um perfil de segurança favorável e reduziram os gradientes em repouso (diferença média: -40 ± 27 mmHg e -43 ± 37 mmHg nas Coortes 1 e 2, p = 0,0003 e p = 0,0004 vs. placebo, respectivamente) e com Valsalva (-36 ± 27 mmHg e -53 ± 44 mmHg, p = 0,001 e < 0,0001 vs. placebo, respectivamente).^154^ Estudos clínicos fase III são aguardados para que os benefícios e segurança dessa droga sejam documentados e permitam alguma recomendação para o seu uso.

##### 4.1.2.3. Disopiramida

A disopiramida é um fármaco antiarrítmico tipo I com efeito inotrópico negativo e pode ser considerada uma alternativa potencial no esquema medicamentoso para CMH com obstrução da VSVE.^155^

Em várias séries de casos, incluindo populações com CMH, a disopiramida se mostrou eficaz em reduzir o gradiente na VSVE.^155 - 157^ A droga foi testada em um estudo retrospectivo multicêntrico, comparando 118 participantes em uso de disopiramida com 373 pacientes sem o uso da medicação num período de seguimento de 3,1±2,6 anos. O estudo demonstrou que 66% dos pacientes não necessitaram de intervenção adicional invasiva e as taxas de mortalidade cardíaca anual por todas as causas não foram estatisticamente diferentes entre os grupos com disopiramida e os controles (1,4% vs. 2,6%/ano, p = 0,07). As taxas de morte súbita também não foram diferentes entre os grupos de tratamento e controle (1,0%/ano vs. 1,8%/ano).

A segurança para o início do tratamento em nível ambulatorial foi investigada numa série grande de casos por Adler et al.^158^ No estudo, de um total de 2.015 pacientes atendidos com CMH, foram observados 168 que iniciaram com disopiramida. Durante o acompanhamento de longo prazo (média de 447 dias), 38 pacientes (23%) desenvolveram efeitos colaterais à disopiramida e 18 (11%) interromperam o medicamento por causa desses efeitos colaterais. A disopiramida na dose de 300 mg prolongou o intervalo QTc médio em 19±23ms. Efeitos anticolinérgicos, como xerostomia, xeroftalmia, retenção urinária e constipação podem ocorrer, sendo que esses sintomas podem ser diminuídos com o uso concomitante de piridostigmina. A aceleração da condução no nodo atrioventricular também pode provocar rápida condução por essa estrutura no caso do surgimento de uma FA.^159^

As diretrizes contemporâneas recomendam o uso da disopiramida para pacientes com não resposta aos betabloqueadores ou bloqueadores dos canais do cálcio, de preferência em associação com alguns desses fármacos, para reduzir sintomas em pacientes com CMH com obstrução dinâmica da VSVE em repouso ou provocada por manobras ou exercício.^2^ Contudo, a medicação não está disponível no Brasil.

##### 4.1.2.4. Inibidores do Sistema Renina-Angiotensina-Aldosterona

Os inibidores do sistema renina-angiotensina-aldosterona são reservados para portadores de CMH em fase de IC com FE reduzida sem padrão obstrutivo. Nos pacientes sem disfunção sistólica do VE e não obstrutivos, essa estratégia terapêutica deve ser individualizada pela falta de resultados claramente benéficos nessa população.

Entre os fármacos moduladores desse sistema, os bloqueadores dos receptores AT1 da angiotensina II (BRA) foram os mais estudados. A partir do racional de sua aplicabilidade na redução da hipertrofia na hipertensão arterial, estudo randomizado comparando a losartana vs. placebo com o objetivo de avaliar a redução da massa ventricular em pacientes com CMH foi realizado. Apesar de não ter demonstrado diferença significativa entre os grupos estudados, o ensaio demonstrou boa tolerância dos pacientes ao fármaco, sem aumento dos episódios de síncope, bem como evidenciou a capacidade de reduzir a pressão arterial, sendo uma opção no tratamento da hipertensão arterial concomitante ao quadro de CMH.^147^

Um *pool* de estudos com a mesma classe de fármacos foi incluído em recente metanálise, sendo observado que o uso dos BRA não mostrou benefício na redução da hipertrofia ventricular nem na melhora da FE.^160^

O *The Valsartan for Attenuating Disease Evolution in Early Sarcomeric Hypertrophic Cardiomyopathy* – VANISH *trial* , foi um estudo fase 2 randomizado com 178 pacientes com CMH sarcomérica randomizados para valsartana 320 mg/dia em adultos e 80 a 160 mg em crianças ou placebo. A média de idade dos participantes foi de 20 a 30 anos, avaliando a valsartana na população de pacientes com CMH e desenhado com desfechos combinados múltiplos incluindo alterações estruturais, funcionais e de biomarcadores. Tal estudo sugeriu melhora no desfecho primário múltiplo, sugerindo potencial de retardar a progressão da doença. No entanto, tal resposta necessita de melhor avaliação em estudos de maior tempo de seguimento.^161^

### 4.2. Terapias de Redução Septal

Uma das características marcantes da CMH é a forte associação entre sintomas e a obstrução do TSVE, sendo a resolução desta um alvo terapêutico reconhecido. Apesar de os fármacos inotrópicos negativos poderem aliviar a obstrução do TSVE e os sintomas em muitos pacientes, cerca de 5 a 10% permanecem refratários à terapêutica farmacológica.^143^

Dessa forma, as terapias de redução septal são alternativas invasivas para os pacientes com CMH obstrutiva que apresentem gradiente no TSVE acima de 50 mmHg, em repouso ou sob provocação, e sintomáticos em classe funcional III e IV a despeito do tratamento medicamentoso otimizado. Atualmente, as terapias de redução septal incluem a alcoolização septal, a miectomia cirúrgica e a ablação septal por cateter de radiofrequência (RF).

#### 4.2.1. Alcoolização Septal

A injeção seletiva de álcool através de ramo perfurante septal foi proposta pela primeira vez em 1995,^162^ com o intuito de provocar uma cicatriz localizada em região septal e reduzir o gradiente intraventricular em pacientes com CMH obstrutiva. A ablação septal por alcoolização (ASA) ocasiona também a redução da insuficiência mitral, da pressão diastólica final do VE, com consequente melhora dos sintomas de dispneia, aumento da capacidade funcional e da qualidade de vida, além de efeitos secundários na redução da incidência de FA e do grau de hipertensão pulmonar.^163 - 169^

Ao longo dessas quase 3 décadas de evolução, houve significativo aperfeiçoamento da técnica, tornando a ASA importante alternativa à miectomia cirúrgica em pacientes sintomáticos, por apresentar menores taxas de complicações periprocedimento, incluindo dor torácica (por evitar a esternotomia), menor tempo de internação e mortalidade < 1% em centros com experiência no procedimento.^165 , 170^ Não há estudo randomizado comparando a redução do gradiente intraventricular entre essas terapias (ASA vs. miectomia cirúrgica). Sendo assim, a literatura é baseada em registros, eventualmente com escores de propensão e seguimento de longo prazo. A ASA em relação à miectomia cirúrgica está relacionada a menores reduções do gradiente intraventricular, especialmente em pacientes com gradientes basais muito altos (> 100 mmHg) e com hipertrofia septal exuberante (> 30 mm), apesar de melhora de classe funcional similar.^171 , 172^ Por outro lado, a presença de doença intrínseca do aparato mitral ou do músculo papilar favorece a miectomia. Nessa situação, a obstrução é ocasionada pelo alongamento dos folhetos mitrais, em especial o anterior, e pelo mau posicionamento dos músculos papilares (geralmente o lateral), que se encontram inseridos anteriormente, mudando a zona de coaptação da valva mitral que é deslocada anteriormente.^169^

O reparo ponta-a-ponta transcateter da válvula mitral (MitraClip) pode ser útil na redução do gradiente do TSVE e no alívio dos sintomas de IC em pacientes com CMH e sintomatologia refratária ligada à obstrução do TSVE que não são apropriados para uma terapia de redução septal. Em uma revisão de quatro estudos realizados entre 2010 e 2016 (15 pacientes, idade média de 79,5 ± 8,1 anos), houve uma redução imediata de 75,8 ± 39,7 para 11,0 ± 5,6 mmHg, mas apenas acompanhamento parcial, até um máximo de 6 meses.^173^

Além disso, em seguimento de longo prazo, a mortalidade global e a ocorrência de morte súbita após ASA foram similares às observadas com a miectomia cirúrgica, bem como em relação a pacientes-controle com CMH não obstrutiva.^164 , 174 - 176^

A principal complicação periprocedimento da ASA é o bloqueio de ramo direito e, eventualmente, o BAV avançado, sendo a necessidade de marca-passo definitivo bastante variável entre 7 e 20%.^166 - 168 , 175 , 177^ Em recente metanálise com 4.213 pacientes submetidos a ASA, a média de implante de marca-passo definitivo foi de 10% vs. 5% após miectomia cirúrgica.^164^ Estudos prévios determinaram que a idade avançada, presença de bloqueio de ramo esquerdo, BAV periprocedimento e duração do intervalo QRS > 120 ms foram preditores da necessidade de marca-passo definitivo.^178 - 180^ Habitualmente, a telemetria por 48 horas é recomendada, com manutenção de marca-passo provisório, especialmente nessas circunstâncias de maior risco para BAV avançado.^179 - 181^ A realização de Holter de 24 horas antes da alta hospitalar também é recomendada.

Em função da grande variabilidade da perfusão da porção basal do septo, a monitorização do procedimento pelo ecocardiograma transtorácico ou transesofágico com contraste é essencial durante o procedimento de ASA.^182 - 185^ Em pacientes nos quais o agente de contraste não pode ser localizado exclusivamente na região basal do septo e adjacente ao ponto de contato mitro-septal, o procedimento deve ser abandonado pelo risco de complicações associadas.^182 - 184^ A injeção de grandes quantidades de álcool (> 2-3 mL), de maneira rápida e envolvendo múltiplos ramos septais, com o intuito de obter reduções adicionais do gradiente, não está indicada pelo alto risco de complicações e eventos arrítmicos.^186^ A ocorrência de nova comunicação interventricular (CIV) como complicação da ASA ou da miectomia cirúrgica tem sido bastante rara nas séries atuais,^164^ porém pode ser mais frequente em pacientes com hipertrofia septal discreta (≤ 16 mm).^3^ Nessa situação, pode-se recomendar terapia alternativa para o tratamento de pacientes selecionados refratários ao tratamento clínico.

A Tabela 13 contempla as recomendações para angiografia e estudo hemodinâmico invasivo.

**Tabela 13:** Recomendações para angiografia e estudo hemodinâmico invasivo

Recomendação	Classe de recomendação	Nível de evidência
Recomenda-se o emprego de estudo hemodinâmico invasivo em pacientes com CMH candidatos para terapia de redução septal nos quais há dúvida quanto à presença e gravidade da obstrução da via de saída do VE nos exames não invasivos; pode-se recomendar o cateterismo cardíaco esquerdo invasivo com manometria.	I	B
Recomenda-se o emprego de estudo hemodinâmico invasivo em paciente com CMH sintomáticos com evidência de isquemia; a angiografia coronária (TCMD ou invasiva) é recomendada.	I	B
Recomenda-se o emprego de estudo hemodinâmico invasivo em pacientes com CMH que têm fatores de risco para aterosclerose coronária; a angiografia coronária (TCMD ou invasiva) é recomendada antes da miectomia cirúrgica.	I	B

CMH: cardiomiopatia hipertrófica obstrutiva; VE: ventrículo esquerdo; TCMD: tomografia computadorizada de múltiplos detectores.

A Tabela 14 contempla as recomendações das terapias de redução septal na CMH obstrutiva.

**Tabela 14:** Recomendações das terapias de redução septal na CMH obstrutiva

Recomendações	Classe de recomendação	Nível de evidência
A terapia de redução septal é recomendada em pacientes com CMH obstrutiva com gradiente em repouso ou provocado > 50 mmHg na via de saída do VE e em classe funcional III-IV da NYHA, apesar de medicação otimizada.	I	B
A terapia de redução septal é recomendada em pacientes com síncope de esforço causada por gradiente de repouso ou provocado na via de saída do VE > 50 mmHg, apesar de terapia médica otimizada.	I	B
É recomendada a realização da terapia de redução septal por operadores experientes, trabalhando dentro de um *Heart Team* com experiência no manejo de pacientes com CMH.	I	C
É recomendada a realização da terapia de redução septal em pacientes com CMH obstrutiva com sintomas importantes (NYHA III-IV), apesar de tratamento otimizado, nos quais a cirurgia é contraindicada ou de alto risco pela presença de comorbidades importantes ou idade avançada; recomenda-se a ASA na presença de anatomia favorável e realizada em centros com experiência.	I	B
A miectomia cirúrgica deve ser preferida em relação à ASA em pacientes com indicação de terapia de redução septal e que requeiram outra intervenção cirúrgica (ou seja, reparo ou substituição mitral, intervenção no músculo papilar, doença coronária multiarterial, estenose aórtica).	I	B
Em todos os pacientes submetidos a ASA, recomenda-se a realização de ecocardiograma transtorácico ou transesofágico com auxílio de contraste de ecocardiografia durante o procedimento, para guiar o procedimento e determinar a correta localização do ramo que se direciona à porção septal.	I	B
Para pacientes sintomáticos com CMH obstrutiva, a terapia de redução septal em pacientes elegíveis, realizada em centros com experiência, pode ser considerada como alternativa ao aumento de medicação após decisão compartilhada incluindo os riscos e benefícios	IIb	B
A terapia de redução septal pode ser considerada para pacientes com sintomas com CF II da NYHA, com presença de: progressiva hipertensão pulmonar causada pela obstrução do TSVE ou insuficiência mitral, aumento atrial esquerdo com mais de 1 episódio de FA, baixa capacidade funcional atribuída a obstrução em teste de esforço gradientes > 100 mmHg em adulto jovem e criança.	IIb	B
Em pacientes com CMH obstrutiva assintomáticos com capacidade normal ao exercício, a terapia de redução septal não está indicada.	III	C
Em pacientes com CMH obstrutiva sintomáticos, nos quais a terapia de redução septal é uma opção, a substituição valvar mitral não deve ser recomendada como opção para aliviar a via de saída do VE.	III	C

ASA: alcoolização septal; VE: ventrículo esquerdo; CMH: cardiomiopatia hipertrófica obstrutiva; TSVE: trato de saída do ventrículo esquerdo; FA: fibrilação atrial.

#### 4.2.2. Miectomia Septal

A miectomia septal para a desobstrução do TSVE foi descrita por Morrow há mais de 60 anos. Essa operação consiste na ressecção retangular da área protusa do septo interventricular, imediatamente abaixo do anel valvar aórtico, na região que se opõe ao folheto anterior da mitral. Esse procedimento permite a redução significativa ou mesmo a abolição do gradiente da via de saída em cerca de 90% dos casos, com redução do MSA, melhora da capacidade aos esforços e alívio dos sintomas.^187 , 188^

Além do impacto na qualidade de vida, a miectomia cirúrgica permite uma expectativa de vida semelhante à população geral sem doença e bastante superior aos pacientes com CMH e obstrução na VSVE que não foram operados.^189^

Nas últimas 2 décadas, concomitante à utilização da ecocardiografia transesofágica, durante a maioria das cirurgias cardiovasculares, houve um melhor entendimento da necessidade de se ampliar a ressecção cirúrgica desse excedente de músculo que existe não somente no TSVE, como em toda porção intraventricular, para o tratamento da CMH obstrutiva. A partir da técnica original, estendeu-se, lateralmente, avançando na região correspondente a nadir do folheto coronariano direito para a esquerda, em direção ao folheto da valva mitral (região relacionada à comissura posterior da valva) e em direção à ponta do coração, com ressecções mais extensas, mais profundas, liberando completamente os músculos papilares, visando redirecionar o fluxo do sangue ântero-medial, afastando-o da valva mitral. Foram resolvidas, com essa abordagem mais ampla, as obstruções intraventriculares, aumentando o enchimento diastólico do VE e, consequentemente, possibilitando a ejeção de maior volume de sangue com menor risco de obstrução, menor turbilhonamento na VSVE e menor ocorrência de MSA. Abordagens valvares mitrais utilizando-se de distintas técnicas são mais comumente empregadas por mais cirurgiões em maior número de pacientes. A anormalidade valvar mitral e dos músculos papilares é identificada já no ecocardiograma pré-operatório, caracterizado pelo alongamento do folheto anterior, deslocamento anormal dos músculos papilares causando um posicionamento mais anteriorizado da valva mitral, encurtamento de cordas tendíneas e músculos papilares anormais que, por vezes, estendem-se até o folheto valvar e precisam ser ressecados, assim como cordas secundárias, a fim de permitir a excursão completa do folheto e reduzir o refluxo mitral, agora não secundário ao MSA.^190 - 192^

Fatores pré-operatórios determinantes de bom prognóstico no longo prazo são: idade menor que 50 anos, átrio esquerdo menor que 46 mm, ausência de FA e sexo masculino.^189 , 193^ Recentemente, mostrou-se que a espessura da parede posterior (> 13 mm) é um preditor negativo a longo prazo, independente da espessura septal e da severidade do gradiente pressórico na VSVE.^194^

A diferença na sobrevida dos pacientes com CMH obstrutiva que foram submetidos à miectomia está relacionada ao alívio hemodinâmico e à melhora da função diastólica, e esta é secundária à redução da isquemia subendocárdica resultante da menor pressão intracavitária pós-cirurgia.^195^

As principais complicações da miectomia são: BAV total, CIV e regurgitação da valva aórtica, porém incomuns em centros de referência.^196 , 197^ A última diretriz da AHA/ACC enfoca os alvos de resultados da terapia invasiva cirúrgica ( Tabela 15 ). Embora a maioria dos pacientes deva ser encaminhada para terapia invasiva com sintomas avançados (NYHA III e IV), um grupo seleto de pacientes menos sintomáticos, mas com outras evidências de distúrbios hemodinâmicos significativos, podem ser elegíveis para a miectomia em centros com *expertise* para minimizar as alterações estruturais e fisiológicas no longo prazo. Uma vasta literatura aponta para reversão da hipertensão pulmonar, melhora da capacidade de tolerância ao exercício, diminuição do tamanho do átrio esquerdo e diminuição das arritmias ventriculares.^12 , 198^

**Tabela 15:** Resultados desejados com a miectomia septal na CMH

Alvo de resultados no tratamento cirúrgico	Resultados desejados
Mortalidade em 30 dias	≤ 1%
Complicações adversas em 30 dias (tamponamento, infecção e sangramento maior)	≤ 10%
Necessidade de marca-passo por bloqueio AV	≤ 5%
Abordagem mitral no primeiro ano	≤ 5%
Insuficiência mitral residual moderada/severa	≤ 5%
Necessidade de refazer o procedimento	≤ 3%
Melhora da classe funcional – NYHA	> 90%
Gradiente pressórico na via de saída do VE (repouso e provocada) < 50 mmHg	> 90%

AV: atrioventricular; VE: ventrículo esquerdo.

Em análise de 2.268 pacientes adultos submetidos à miectomia cirúrgica em centro de referência, comparou-se a intervenção precoce (CF II) com a indicação classe 1 (1.318 pacientes, 58% enquadravam-se na classe 1 de indicação vs. 950 pacientes, 42% em indicação precoce). Em 6,2 ± 4 anos de seguimento, os pacientes com indicação precoce apresentaram significativa redução de mortalidade e necessidade de uso de cardiodesfibrilador ventricular quando comparados aos pacientes com indicação convencional (p < 0,001) e sobrevida similar à população sem doença pareada para idade e sexo.^199^

A escolha do procedimento invasivo mais apropriado para cada paciente deve ser individualizada e discutida em " *Heart Team* " com experiência no tratamento da CMH, e devem-se levar em conta a fragilidade do paciente, a anatomia coronária para ablação septal e o aparato valvar mitral.^174^

**Figura 16:**
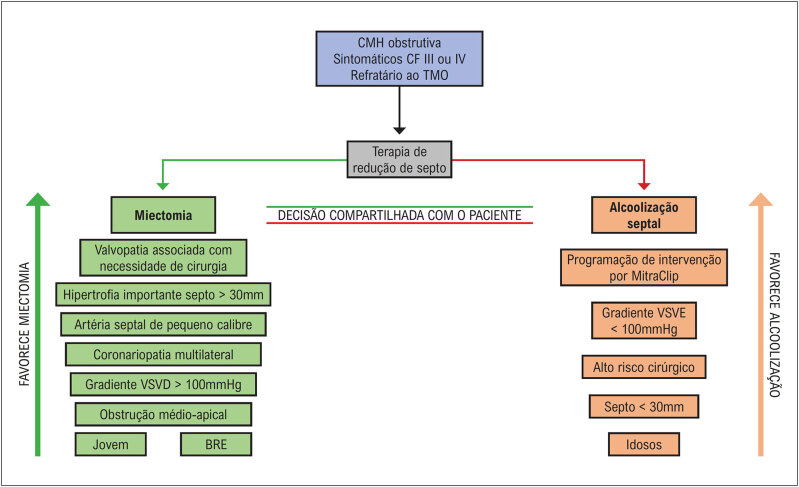
Aspectos a serem considerados na tomada de decisão sobre a estratégia de terapia de redução septal nos pacientes com CMH. CMH: cardiomiopatia hipertrófica; TMO: tratamento médico otimizado; VSVE: via de saída do ventrículo esquerdo; BRE: bloqueio do ramo esquerdo.

A Figura 16 contempla os aspectos a serem considerados na tomada de decisão sobre a estratégia de terapia de redução septal nos pacientes com CMH.

**Figura 17:**
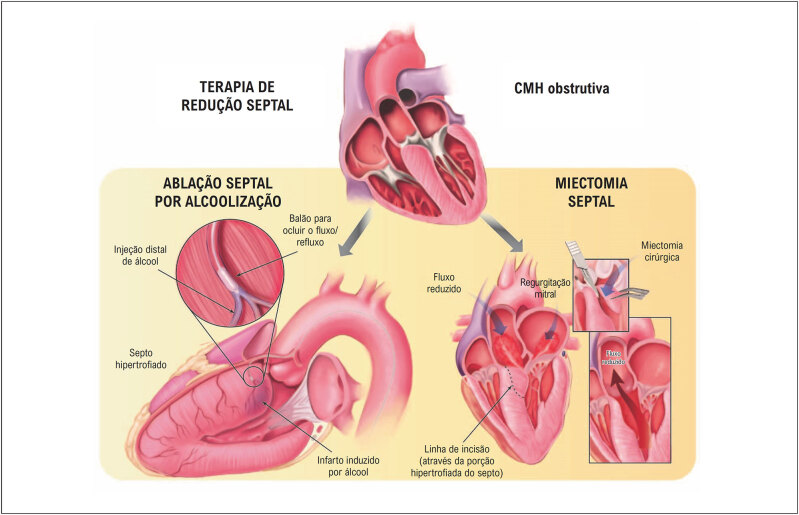
Ilustrando graficamente as principais terapias de redução septal disponíveis, alcoolização e miectomia. Adaptada de Nishimura e Holmes Jr.^200^

A Figura 17 ilustra graficamente as principais terapias de redução septal disponíveis, alcoolização e miectomia.

#### 4.2.3. Ablação Septal por Cateter de Radiofrequência

A ablação septal endocárdica por cateteres com energia de RF foi descrita inicialmente por Lawrenz et al. como nova modalidade, sendo ainda considerada experimental, de tratamento invasivo não cirúrgico dos pacientes com CMH obstrutiva, objetivando redução do gradiente intraventricular como alternativa para a ASA quando a anatomia ou território de irrigação da artéria septal não forem adequados para o procedimento.^201^

A técnica consiste na aplicação de energia de RF através de cateteres utilizados originalmente para ablação de arritmias em estudos eletrofisiológicos. A energia de RF é convertida em energia térmica no miocárdio, causando lesão tecidual, edema perilesional e, posteriormente, cicatriz definitiva. A profundidade da cicatriz causada pela RF varia de 5 a 12 mm e tem seu formato radial bem delimitado a partir do ponto de contato do cateter com o tecido miocárdico. A análise das imagens, em especial da via de acesso ao VE, é relevante para a escolha da técnica (retroaórtica ou transeptal) e expectativa de resultados, bem como programação de procedimentos combinados em caso de ablação como preparo para implante valvar aórtico percutâneo. Outro benefício é o planejamento do alvo da ablação baseado no fenótipo. Quando a hipertrofia é unicamente da região basal, são necessárias aplicações menos extensas que em situações em que a hipertrofia se estende até o terço médio ou apical.

Em metanálise recente, a mortalidade intraoperatória foi inferior a 2%,^202^ com mortalidade ao final de 1 ano de 1,8% até o momento.^203^^,^^204^

Espera-se redução do gradiente entre 60 e 90%. O índice de BAV total e implante de marca-passo definitivo também foi de 0 a 25%, de acordo com a técnica e agressividade selecionada pelos diferentes autores.^203^ Valdigem et al. reportaram baixa taxa de bloqueio de ramo esquerdo (cerca de 10%) e nenhum BAV ou aumento de intervalo atrioventricular (AV) com indicação de marca-passo definitivo.^205^ Ablações mais agressivas obtêm maior redução à custa do aumento de complicações, devido a edema da via de saída e envolvimento do sistema de condução.^202^

A redução do gradiente é progressiva ao longo dos primeiros 3 a 6 meses da ablação, com delimitação da área de fibrose e remodelamento do VE. Alguns pacientes podem ter redução adicional até o final do primeiro ano.^205 , 206^

O uso de ablação septal com RF pode ser considerado nos pacientes com CMH obstrutiva, sintomáticos apesar do tratamento clínico otimizado, quando o risco cirúrgico para miectomia for elevado, a anatomia coronariana não for favorável para alcoolização septal e for realizada por equipe experiente em ablação por cateter com RF e mapeamento eletroanatômico, quando disponível, grau de recomendação IIB, nível de evidência B ( Tabela 16 ).

**Tabela 16:** Recomendações da ablação septal por cateter com radiofrequência e constituição de equipes

Recomendações	Classe de recomendação	Nível de evidência
O uso de ablação septal com RF pode ser considerado nos pacientes com cardiomiopatia hipertrófica obstrutiva, com gradiente intraventricular ≥ 50 mmHg em repouso ou durante provocação, sintomáticos em CF III-IV da NYHA, apesar do tratamento clínico otimizado, quando o risco cirúrgico para miectomia for elevado, a anatomia coronariana não for favorável para alcoolização septal e for realizada por equipe experiente em ablação por cateter com RF e mapeamento eletroanatômico, quando disponível.	IIb	B
A ablação septal com RF pode ser considerada em portadores de gradiente residual após miectomia ou alcoolização septal quando a nova intervenção com a mesma modalidade não é factível ou desejada.	IIb	C
A equipe para realização da ablação septal com RF deve ser composta por pelo menos um eletrofisiologista experiente em casos complexos de ablação por cateter com RF, um ecocardiografista experiente em avaliações de pacientes com cardiopatias complexas e um cardiologista com experiência em terapia intensiva.	IIa	C

RF: radiofrequência.

#### 4.2.4. Estratificação do Risco e Prevenção da Morte Súbita

A morte súbita é a complicação mais visível e impactante da CMH.^1^ Um dos principais alvos terapêuticos no manuseio da doença é a redução da ocorrência desse evento catastrófico. Até a introdução do CDI na prática clínica, confiavelmente terminando taquiarritmias ventriculares, não havia tratamento preventivo da morte súbita.^7 , 207^ Nas últimas 2 décadas, com o emprego do CDI e o desenvolvimento e evolução de algoritmos para a identificação acurada dos pacientes com CMH e alto risco de morte súbita, foi possível uma grande redução da mortalidade da doença de taxas tão altas como 6% ao ano, nas eras pré-CDI, para taxas de 0,5% ao ano, relatadas em coortes recentes em centros terciários.^7 , 207^

Apesar dessa conquista, há ainda lacunas a serem preenchidas na estratificação de risco, principalmente a redução do número de implantes desnecessários.^208^ A identificação acurada do paciente de alto risco que deve apropriadamente receber implante de CDI e também daquele que não deve receber, por ter baixo risco de morte súbita associado ao risco de complicações inerentes ao implante do dispositivo, é um grande desafio.^2 , 209^

#### 4.2.5. Fatores de Risco

Ao longo de décadas, foram publicados vários estudos observacionais retrospectivos que identificaram a associação de variáveis clínicas, demográficas, de exames de imagem, com o risco de futuras taquiarritmias ventriculares e morte súbita que passaram a ser reconhecidos como fatores de risco.^7 , 207 - 210^

Observamos uma constante evolução desses marcadores de risco, ao longo do tempo, alguns deixando de ser valorizados, como o comportamento da pressão arterial durante o teste ergométrico, outros mantendo-se como marcador de risco apenas em determinadas circunstâncias ou associados a outro fator, como a TNVS.^2^ Os detalhes desses marcadores de risco também foram sendo modificados, como, por exemplo, a HVE extrema, que era considerada apenas quando maior de 30 mm, passou a também ser considerada quando ≥ 28 a 29 mm; história familiar de morte súbita, anteriormente considerada somente quando o familiar tinha menos de 40 anos, passou-se a valorizar também com idade maior, até 50 anos.^2^ Novos marcadores de risco foram surgindo, como a presença de aneurisma apical, a queda da FE para inferior ou igual a 50% e o realce tardio na RMC.^2 , 209^

Atualmente, os fatores de risco reconhecidos e valorizados, usados para determinar risco elevado de morte súbita e identificar pacientes para os quais se deve indicar o implante de CDI para prevenção primária, são baseados na história pessoal (antecedente de parada cardíaca prévia ou TVS ou fibrilação ventricular, síncopes inexplicadas), história familiar (morte súbita de familiares), achados ecocardiográficos e de RMC (HVE extrema, FE < 50%, aneurisma apical e realce tardio) e *holter* de 24 horas (presença de TVNS).^209 , 210^ Os detalhes da caracterização desses fatores de risco estão na Tabela 17 .

**Tabela 17:** Caracterização dos fatores de risco para morte súbita cardíaca na CMH

História pessoal: antecedente de taquicardia ventricular sustentada sincopal/FV	Prevenção secundária: alto risco de novos eventos recorrentes
História pessoal: síncopes inexplicadas	Pelo menos um episódio de perda transitória da consciência, descartando-se ser: pela obstrução do TSVE ou vasovagal. Maior importância se ocorrido nos últimos 6 meses.
História familiar: MS na família	MS provável ou certa atribuível a CMH em ≥ 1 parente de 1^o^ ou 2^o^ grau com ≤ 50 anos de idade. Se de 3^o^ grau: quando múltiplos.
ECO/RNM: HVE ≥ 28 mm	Espessura de parede ≥ 28 mm em qualquer segmento de parede ventricular, por ECO/RNM.
ECO/RNM: disfunção sistólica	FE < 50% por ECO ou RNM
ECO/RNM: aneurisma apical de VE	Presente: maior risco
RMC: realce tardio > 15%	Realce tardio difuso e extenso quantificado ou estimado por inspeção visual compreendendo ≥ 15% da massa total do VE.
*Holter:* TVNS	TVNS valorizar se episódios de TVNS são frequentes (≥ 3), mais longos (≥ 10 batimentos) e mais rápidos (≥ 200 batimentos por minuto)

TSVE: trato de saída de ventrículo esquerdo; MS: morte súbita, RNM: ressonância do coração, ECO: ecocardiograma; TVNS: taquicardia ventricular não sustentada; CMH: cardiomiopatia hipertrófica; FE: fração de ejeção; FV: fibrilação ventricular; HVE: hipertrofia ventricular esquerda; VE: ventrículo esquerdo; RMC: ressonância Magnética Cardíaca

A idade do paciente também influencia grandemente o risco de morte súbita nos pacientes com CMH e deve ser levada em conta na tomada de decisão para o implante de CDI. Coortes mostram uma taxa de eventos de MSC muito baixa em pacientes mais idosos (> 60 anos) e clinicamente estáveis, sendo uma população em que o benefício de implante de CDI é duvidoso.^40^ Por outro lado, o risco de MSC é maior em crianças e adolescentes quando comparados com pacientes adultos.^211^ Um estudo de coorte envolvendo 150 pacientes de diversas faixas etárias conduzido na Suécia mostrou um risco de morte súbita maior em pacientes entre os 9 e 13,9 anos de idade (taxa de 7,2% ao ano) quando comparado aos pacientes após 16 anos de idade (taxa de 1,7% ao ano), com relação de risco 3,75 vezes maior (IC 1,18-11,91).

##### 4.2.5.1. Novos Fatores de Risco

A disfunção ventricular esquerda (FE inferior a 50%) e a presença de fibrose e de aneurisma apical foram recentemente reconhecidas como fatores de risco por estarem associadas à morte total e súbita em vários estudos.^2^ No que diz respeito à disfunção ventricular esquerda, sabemos que a hipercontratilidade é um dos mecanismos fisiopatológicos da CMH.^2^ Quando a FE se reduz para valor igual ou inferior a 50%, chamada "fase final" da CMH, a média de sobrevida após o desenvolvimento da disfunção ventricular é de 8,4 anos, e alguns fatores, como presença de fibrose, presença de variantes sarcoméricas patogênicas ou provavelmente patogênicas múltiplas, FA e FE inferior a 35% implicam em pior prognóstico.^212^

A presença de fibrose (visualizado como realce tardio nas imagens de RNM) é fator de risco para a ocorrência de arritmias ventriculares por reentrada, que se baseiam no tripé de substrato, gatilhos e ambiente propiciador.^213 , 214^

A ausência de realce tardio pode identificar pacientes de melhor prognóstico em portadores de CMH com função preservada.^213 , 214^ Existe aumento linear do risco de morte com aumento da porcentagem de realce tardio até um corte de 15%, quando o aumento dessa inclinação da curva de risco é mais íngreme (implicando em maior risco de morte).^213 , 214^ A presença dessa magnitude de realce tardio pode ser muito útil como fator arbitrário para pacientes nos quais a determinação do risco de MS permanece ambígua.^2 , 209^ Já a ausência de realce tardio é associada com baixo risco para eventos adversos.^209^

A presença de aneurisma apical definida com uma parede acinética ou discinética, independentemente do tamanho, foi associada em estudos recentes com morte súbita. Pode não ser detectada pelo ecocardiograma e, sim, na RMC.^2 , 209^

Dessa forma, a presença de pelo menos um fator de risco é suficiente para conferir um risco alto de morte súbita e justificar o implante de CDI.^2 , 7 , 207 - 210^ Apesar de tratados de forma independente, os fatores de risco são potencializadores uns dos outros. Podemos observar que, em portadores de disfunção ventricular, a presença de fibrose ganha relevância maior que em pacientes com função preservada.^2^

Porque o risco de MS se estende ao longo de toda a vida do paciente, a reavaliação periódica do risco se impõe como componente fundamental de toda avaliação longitudinal do paciente com CMH.^2 , 207^ Recomenda-se a estratificação de risco na visita inicial e, em seguida, repetição anualmente.

A decisão de implante de CDI requer a informação ao paciente de todas as vantagens e desvantagens do implante de um dispositivo a longo prazo, processo conhecido como decisão compartilhada e que é muito importante na CMH, particularmente quando a indicação de CDI é ambígua.^2^

#### 4.2.6. Calculadora de Risco de Morte Súbita

Uma ferramenta para apoio à tomada de decisão da melhor estratégia para prevenção da MSC em pacientes com CMH é a estimativa do risco de MSC em 5 anos mediante emprego da calculadora de risco " *HCM Risk-SCD* ", construída através de uma equação de regressão logística que incorpora múltiplos fatores de risco.^3 , 215^ As variáveis consideradas foram: idade, diâmetro atrial esquerdo (DAE), gradiente máximo de VSVE, presença de síncopes inexplicadas, TVNS com frequência ventricular maior que 120 bpm e história familiar de morte súbita em familiares de primeiro grau com idade inferior a 40 anos.^3 , 215^ Esse modelo de predição de risco permite categorizar os pacientes em três categorias de risco: baixo (< 4%), intermediário (4-6%) ou alto (≥ 6%), implicando na recomendação de implante de CDI nos pacientes na categoria de risco mais alta.^215^

Contudo, é importante lembrarmos que o modelo de predição de risco não leva em consideração o impacto dos novos marcadores de morte súbita, incluindo disfunção sistólica (FE < 50%), aneurisma apical e realce tardio, e valoriza variáveis como DAE e gradiente intra-ventricular não considerados no modelo de fatores de risco e não documentados como claramente associados à morte súbita.^2 , 209 , 210^ Além disso, a utilidade do modelo de predição de risco é incerta nos pacientes com CMH após miectomia ou ablação com álcool e não é recomendada para estratificação de risco em crianças e adolescentes com menos de 16 anos.^215^ Em 2019, foi desenvolvido um modelo de predição de risco para crianças entre 0 e 16 anos,^216^ validado retrospectivamente em 2022, com o risco ≥ 6% identificando 70% dos eventos.^217^

Por outro lado, um estudo com 3.703 pacientes (EVIDENCE-HCM) analisando retrospectivamente o *HCM Risk-SCD* demonstrou uma capacidade moderada de discriminar entre os pacientes de alto e não alto risco.^218^

Um grande número de coortes retrospectivas tem investigado a eficácia do modelo de predição de risco ( *HCM Risk-SCD* ) em comparação à abordagem baseada na detecção de fatores de risco, aplicando essas duas abordagens em populações com evolução clínica conhecida (com e sem CDI).^207 , 209 , 219 - 222^ Nessas análises, o modelo de predição de risco ( *HCM Risk-SCD* ) teve uma sensibilidade de 33% para identificar pacientes com eventos de morte súbita subsequentes, enquanto a abordagem baseada em fatores de risco teve sensibilidade de 95%.^207 , 219 - 222^ Já no que diz respeito à especificidade, isso é, identificar os pacientes que têm risco baixo para os quais o implante de CDI é desnecessário, o *HCM Risk-SCD* foi superior, com especificidade de 92% comparada com 78% da abordagem baseada em fatores de risco, com o benefício potencial de diminuir 20% dos implantes. No entanto, o número necessário para tratar e salvar uma vida (NNT) foi comparável entre os dois modelos (6,6 na abordagem baseada em fatores de risco vs. 7,2 usando-se o *HCM Risk-SCD* ). Por outro lado, o número de implantes desnecessários poderia ser menor caso as coortes tivessem maior tempo de seguimento, pois 36% dos pacientes com CMH recebem sua primeira terapia por arritmia de alto risco de taquicardia/fibrilação ventricular além de 10 anos após o implante do CDI.^223^

**Figura 18:**
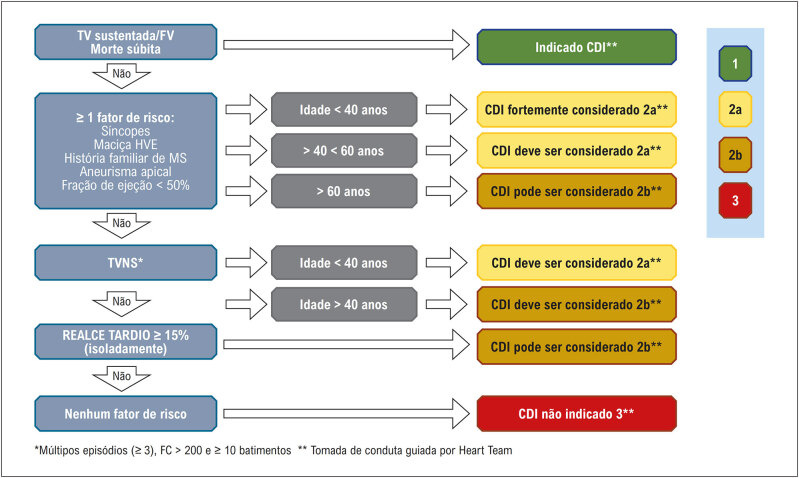
Algoritmo de estratificação de risco de morte súbita e indicação de CDI nos pacientes com CMH. TV: taquicardia ventricular; FV: fibrilação ventricular; HVE: hipertrofia ventricular esquerda; MS: morte súbita; TVNS: taquicardia ventricular não sustentada; CDI: cardiodesfibrilador implantável; FC: frequência cardíaca.

Com base nas evidências apresentadas acima, o algoritmo com as recomendações para o implante de CDI e prevenção de MSC nos pacientes com CMH estão representadas na Figura 18 .

Seguindo-se o algoritmo, recomenda-se o implante de CDI nos pacientes exibindo TVS, MSC revertida ou fibrilação ventricular (Classe de recomendação I, nível de evidência A).

Demais pacientes com CMH que exibam pelo menos um dos fatores de risco para MSC (síncopes, história familiar para morte súbita, HVE maciça, aneurisma apical, FEVE < 50%), devem ser estratificados conforme a idade. Aqueles menores de 40 anos, incluindo as crianças, devem ser considerados para CDI; entre 40 e 60 anos, devem ser considerados para implante de CDI após discussão em centro de referência; os pacientes acima de 60 anos podem ser considerados para implante de CDI ( Tabela 18 ).

**Tabela 18:** Recomendações para estratificação de risco de morte súbita cardíaca e implante de CDI em pacientes com CMH.

Recomendações	Classe de recomendação	Nível de evidência
Os seguintes fatores de risco devem ser pesquisados em toda avaliação clínica: PCR abortada, TV/FV. Síncopes inexplicadas, história familiar de MS < 50 anos de idade, acentuada HVE (≥ 28mm), aneurisma apical, FEVE < 50%.	I	C
Em casos de dúvida na indicação de CDI, a realização de pesquisa de TVNS por monitorização prolongada e a realização de ressonância de coração para pesquisa de realce tardio e aneurisma apical são indicadas.	I	C
O uso da calculadora eletrônica deve ser considerado para avaliar o risco de morte em 5 anos em pacientes com mais de 16 anos no processo de decisão compartilhada quanto ao implante de CDI.	IIa	B
Havendo evidências de ter ocorrido PCR reanimada por TVS ou FV, o implante de CDI está indicado	I	B
O implante de CDI deve ser considerado em pacientes com idade inferior ou igual a 40 anos na presença de pelo menos um dos seguintes fatores de risco: síncopes inexplicadas (excluindo-se ser vasovagal ou atribuída à OTSVE), acentuada HVE (espessura septal ≥ 28 mm), história familiar de MS com idade ≤ 50 anos de idade, aneurisma apical de VE e disfunção sistólica (fração de ejeção ≤ 50%).	IIa	B
O implante de CDI deve ser considerado em pacientes com idade maior que 40 e inferior ou igual a 60 anos na presença de pelo menos um dos seguintes fatores de risco: síncopes inexplicadas (excluindo-se ser vasovagal ou atribuída à OTSVE), acentuada HVE (espessura septal ≥ 28 mm), história familiar de MS com idade ≤ 50 anos de idade, aneurisma apical de VE e disfunção sistólica (fração de ejeção ≤ 50%) em decisão compartilhada.	IIa	B
O implante de CDI pode ser considerado em pacientes com idade superior a 60 anos na presença de pelo menos um dos seguintes fatores de risco: síncopes inexplicadas (excluindo-se ser vasovagal ou atribuída à OTSVE), acentuada HVE (espessura septal ≥ 28 mm), história familiar de MS com idade ≤ 50 anos de idade, aneurisma apical de VE e disfunção sistólica (fração de ejeção ≤ 50%).	IIb	B
O implante de CDI deve ser considerado na presença de TVNS, principalmente se houver múltiplos episódios (> 3), episódios mais rápidos (FC > 200) e mais longos (> 10 batimentos) se a idade for inferior a 40 anos.	IIa	A
O implante de CDI pode ser considerado na presença de TVNS, principalmente se houver múltiplos episódios (> 3), episódios mais rápidos (FC > 200) e mais longos (> 10 batimentos) se a idade for maior ou igual a 40 anos.	IIb	B

TV: taquicardia ventricular; FV: fibrilação ventricular; PCR: parada cardiorrespiratória; MS: morte súbita; HVE: hipertrofia ventricular esquerda; FEVE: fração de ejeção ventricular esquerda; CDI: cardiodesfibrilador implantável; TVNS: taquicardia ventricular não sustentada; TVS: taquicardia ventricular sustentada; OVSVE: obstrução na via de saída de ventrículo esquerdo; VE: ventrículo esquerdo; FC: frequência cardíaca.

Os pacientes que não exibam os fatores de risco listados acima, mas que exibam TVNS (múltiplos episódios, mais longos > 10 batimentos e mais rápidas ≥ 200 bpm), devem ser considerados para implante de CDI se forem mais jovens (< 40 anos, incluindo crianças), enquanto os pacientes ≥ 40 anos podem ser considerados para receber CDI ( Tabela 18 ).

Os pacientes sem outros fatores de risco e sem exibir TVNS, mas com realce tardio em extensão ≥ 15%, podem ser considerados para implante de CDI ( Tabela 18 ).

Os pacientes que não exibirem qualquer dos fatores de risco, TVNS ou realce tardio ≥ 15%, têm contraindicação para implante de CDI ( Tabela 18 ).

Vale ressaltar que pacientes com múltiplos fatores de risco podem ser considerados com maior risco de morte súbita, aspecto que pesa a favor do implante de CDI em cada uma das situações clínicas mostradas no fluxograma.

#### 4.2.7. Seleção e Programação do CDI em Pacientes com Cardiomiopatia Hipertrófica

Depois da decisão de implante de CDI em pacientes com CMH, os próximos passos são a escolha do tipo de sistema a ser implantado e o ajuste de sua programação.^224^ Atualmente, estão disponíveis sistemas de CDI ventricular (V) e atrioventricular (AV) por acesso intravascular e sistema de CDI-V por acesso subcutâneo.

A decisão de escolha desses sistemas depende de vários aspectos clínicos e técnicos inerentes ao paciente e da arritmia que deverá ser identificada e revertida. Os fatores mais relevantes que devem ser considerados são os seguintes: a indicação do CDI (prevenção primária ou secundária); idade do paciente (crianças, jovens e idosos), conformação física (local do implante e adequação), ritmo de base (sinusal estável, bradiarritmia ou FA),^2^ facilidade de acesso intravascular e arritmia documentada (TV sustentada monomórfica ou TV polimórfica/FV).^225^

Nos pacientes em ritmo sinusal estável e condução AV preservada em que a indicação é de prevenção primária de morte súbita, o objetivo do dispositivo é a detecção segura da taquiarritmia ventricular e a liberação do choque para sua reversão. Nesse caso, o CDI transvenoso com *coil* único e o CDI subcutâneo têm sido recomendados, principalmente em indivíduos mais jovens, devido ao menor risco de complicações em longo prazo.^226 - 228^ Na decisão entre os sistemas, deve-se considerar que o CDI transvenoso tem a vantagem da estimulação ventricular em caso de bradicardia acentuada, mas desvantagem em relação ao subcutâneo pela presença do eletrodo intravascular, que pode apresentar disfunção de reconhecimento da atividade elétrica local (" *undersensing* ") ou de estimulação, risco de infeção e endocardite, além do risco envolvido na troca dos eletrodos, principalmente quando mais antigos.^229 - 231^ O CDI subcutâneo, por outro lado, apresenta maior facilidade de remoção, quando necessária. Entretanto, o CDI subcutâneo tem um tamanho maior, não sendo adequado para indivíduos com massa corpórea muito baixa; apresentam menor longevidade da bateria, maior risco de choques inapropriados por percepção inadequada (" *oversensing* ") da onda T e miopotenciais. Por isso, os pacientes devem ser avaliados antes e depois do implante. Além disso, a experiência clínica do seu uso é menor em nosso país.^232 - 235^

É importante destacar que alguns pacientes com CMH apresentam TV monomórfica recorrente; neles, o sistema intravascular é a melhor indicação devido à disponibilidade do mecanismo antitaquicardia do CDI para interromper os episódios de TVS sem a necessidade de liberação dos choques. Na programação desses dispositivos, o sistema antitaquicardia deve ser ativado, e a programação ajustada com objetivo de minimizar os choques (apropriados e inapropriados) do equipamento.^34 , 236 , 237^

Os sistemas de estimulação de câmara única usualmente têm menos complicações, no curto e no longo prazo, em comparação com os sistemas transvenosos de dupla câmara.^36 - 39 , 238 - 241^ Além disso, ensaios clínicos randomizados também mostraram redução aguda da obstrução da via de saída do VE pela estimulação persistente do VD com melhora sintomática em pacientes com CMH obstrutiva.^242 , 243^ Entretanto, os resultados clínicos em longo prazo não foram persistentes, exceto em pacientes acima de 65 anos.^244 - 248^

Os CDIs com estimulação dupla câmara têm um eletrodo atrial adicional no átrio direito (AD) que ajuda no diagnóstico diferencial de TVs das supraventriculares; entretanto, as taxas de complicações são maiores quando comparados aos dispositivos de câmara única.^34 , 236 , 238 - 240^ O risco/benefício de seu uso deve ser balanceado, dependendo das características clínicas do paciente e do potencial benefício oferecido pelo sistema.

O benefício clínico da terapia de ressincronização cardíaca (CRT) foi demonstrado em estudos clínicos randomizados de pacientes com IC, bloqueio de ramo esquerdo, disfunção ventricular sistólica importante e terapia clínica otimizada, portadores de cardiopatia isquêmica e não isquêmica, mas sem representatividade de pacientes com CMH. De fato, séries isoladas envolvendo pacientes com CMH demonstram resposta clínica favorável, mas inferior à resposta obtida em pacientes com cardiopatia isquêmica e dilatada. Estudos mais recentes sugerem que a resposta clínica fica comprometida pela grande extensão da fibrose miocárdica na fase muito avançada da CMH com dilatação e FE abaixo de 35%, sem evidência de benefício na sobrevivência; além disso, os respondedores apresentaram apenas melhora discreta na FEVE.^244 , 248^ O benefício parece ser mais evidente nos pacientes com bloqueio de ramo esquerdo e duração do QRS muito prolongada e FE entre 35% e 50%.^244 - 246^

A Tabela 19 contempla as recomendações para escolha do CDI.

**Tabela 19:** Recomendações para escolha do CDI

Recomendações	Classe de recomendação	Nível de evidência
Nos pacientes com CMH com indicação de CDI para prevenção de morte súbita, CDIs transvenoso de câmara única, câmara dupla ou subcutâneo são recomendados. A escolha do melhor dispositivo deve ser feita após uma discussão compartilhada que deve levar em consideração as preferências do paciente, seu estilo de vida e a necessidade potencial de estimulação para bradicardia ou de utilização do mecanismo antitaquicardia para término de TV monomórfica sustentada.^20 - 32^	I	B
Em pacientes com CMH que estão recebendo um CDI transvenoso, os sistemas com *coil* único são recomendados.^226^	I	B
Os CDIs de dupla câmara devem ser considerados para pacientes com necessidade de estimulação atrial ou sequencial atrioventricular para pacientes com bradicardia mais acentuada ou anormalidades importantes da condução atrioventricular, ou como uma tentativa de aliviar os sintomas da CMH obstrutiva (a maioria comumente em pacientes > 65 anos de idade).^34 , 236^	IIA	B
Em pacientes adultos selecionados com CMH recebendo um CDI com IC classe funcional II a IV da NYHA ambulatorial, bloqueio de ramo esquerdo e FEVE < 50%, a CRT deve ser considerada para redução dos sintomas.^244 - 248^	IIA	B
Em pacientes com CMH nos quais uma decisão foi feita para implante de CDI e que têm taquicardias atriais paroxísticas ou FA, CDIs de dupla câmara podem ser considerados.^239 - 243^	IIb	B

CMH: cardiomiopatia hipertrófica; CDI: cardiodesfibrilador implantável; TV: taquicardia ventricular; IC: insuficiência cardíaca; FEVE: fração de ejeção ventricular esquerda; CRT: terapia de ressincronização cardíaca; FA: fibrilação atrial.

### 4.3. Tratamento das Arritmias Atriais

#### 4.3.1. Introdução

A FA é a arritmia mais comum observada em pacientes com CMH, ocorrendo em aproximadamente 20% dos pacientes em centros de referência, cerca de 6 vezes maior do que na população geral. A arritmia está associada a aumento da morbidade, do risco de AVC e piora da qualidade de vida. O tratamento da FA inclui a prevenção de eventos tromboembólicos e o controle dos sintomas, por controle do ritmo ou de frequência. Os principais fatores predisponentes são: aumento do átrio esquerdo e a idade. Outros fatores possivelmente relacionados à FA em pacientes com CMH são: a obstrução da VSVE, duração da onda P maior que 140 ms, taquiarritmias atriais, alterações do segmento ST-T no ECG basal, extrassístoles ventriculares, realce tardio na RNM e reserva de fluxo coronariano anormal.^2 , 3 , 33^

Estratégias para prever a ocorrência de FA em pacientes com CHM são importantes, como no caso do escore *HCM-AF,* já testado e validado externamente. Nele, avalia-se a possibilidade de desenvolvimento de FA em 5 anos (baixo, intermediário e alto risco), usando variáveis tais como: tamanho do átrio, idade, idade ao diagnóstico de CMH e sintomas de IC.^249^

#### 4.3.2. Tratamento Medicamentoso

A manutenção do ritmo sinusal em pacientes com CMH é a estratégia ideal devido a duas considerações: a) a perda da contração atrial é claramente associada à piora da classe funcional e aumenta a chance de internação por IC; b) o controle precoce do ritmo tem maior chance de evitar o ciclo vicioso entre a perda da contração atrial e o aumento da pressão e do remodelamento atrial esquerdo, aumentando a chance de obter ritmo sinusal a longo prazo.

Apesar de a estratégia de controle de ritmo ser a de preferência, já que a redução de desfechos foi demonstrada quando comparada com o controle de frequência,^27 , 250^ o tratamento medicamentoso para controle da frequência pode ser realizado, preferencialmente com bloqueador de canal de cálcio não dihidropiridínico, betabloqueadores ou suas combinações. O uso da digoxina deve ser evitado pela possibilidade de piorar a obstrução da VSVE devido ao efeito inotrópico positivo.

Na estratégia de controle de ritmo, a utilização dos antiarrítmicos amiodarona e sotalol (principalmente em pacientes mais jovens devido aos paraefeitos a longo prazo) se mostrou segura.^251 - 255^ Devido à presença de significativa HVE, deve-se considerar o risco de proarritmia. A propafenona apresenta dados limitados, e seu uso deve ser limitado a pacientes portadores de CDI.^2^

A eficácia do tratamento medicamentoso é limitada e menor do que em pacientes sem CMH, porém os dados são baseados em estudos pequenos e observacionais com um número reduzido de pacientes.^255^ Um recente estudo retrospectivo com 98 pacientes^252^ relata a manutenção do ritmo sinusal em 62% após 1 ano de tratamento e em apenas 42% após 3 anos. A amiodarona foi descontinuada por significativos efeitos colaterais em 19%.

#### 4.3.3. Tratamento Invasivo

A ablação por cateter é um procedimento minimamente invasivo, seguro e desempenha papel importante para o controle do ritmo em pacientes com CMH, porém a fisiopatologia está relacionada ao maior grau de remodelamento elétrico e estrutural das cavidades cardíacas, com predomínio de dilatação e fibrose intersticial difusa.

Estudos recentes evidenciaram que os resultados são menos favoráveis do que em pacientes com outras patologias ou sem cardiopatia estrutural, com taxas maiores de recidiva e consequente necessidade de repetição do procedimento.^256 , 257^

Em um estudo recente com apenas 111 pacientes^257^ seguidos por 6 anos, 61% dos pacientes submetidos a ablação mantiveram o ritmo sinusal. As recidivas necessitaram repetição do procedimento e uso concomitante de drogas antiarrítmicas.

Metanálises publicadas nos últimos 5 anos demonstraram o dobro de recidiva em relação ao grupo-controle,^258 - 260^ porém, com a associação do tratamento antiarrítmico, a taxa de sucesso após um primeiro procedimento foi de 75%. A precocidade da indicação do procedimento ablativo parece ser importante para a manutenção do ritmo sinusal.

Com isso, as estratégias mais utilizadas para a ablação por cateter nos pacientes com CMH usualmente incluem abordagens mais extensas, envolvendo regiões além do tradicional isolamento das veias pulmonares, como a formações de lesões lineares e ablação de gatilhos extrapulmonares.^2^

Em pacientes submetidos a miectomia cirúrgica, a ablação por RF durante o procedimento pode ser realizada. Essa estratégia foi associada a sobrevida livre de FA em 3 anos de aproximadamente 70%, sendo que o tamanho do átrio é o maior preditor de recidiva.^27^

A ablação do nó atrioventricular com implante de marca-passo como forma de controle de frequência pode ser utilizada em casos refratários. O uso de oclusor do apêndice atrial esquerdo foi avaliado em pequenos grupos, não sendo indicado como padrão para essa população de pacientes.^2^

A Tabela 20 contempla as recomendações para pacientes com CMH e FA.

**Tabela 20:** Recomendações para pacientes com CMH e FA^1^

Recomendações	Classe de recomendação	Nível de Evidência
Em situações em que a estratégia seja o controle de frequência, são recomendados betabloqueadores ou bloqueador do canal de cálcio (verapamil ou diltiazem).	I	C
Em portadores de CMH e FA sintomática, a estratégia de controle de ritmo pela ablação por cateter deve ser considerada.	IIa	B
Em uma estratégia de controle do ritmo, a cardioversão elétrica ou drogas antiarrítmicas devem ser consideradas.	IIa	B
Em pacientes que necessitam de miectomia cirúrgica, a ablação de FA concomitante deve ser considerada.	IIa	B

CMH: cardiomiopatia hipertrófica; FA: fibrilação atrial.

A Tabela 21 contempla as opções de tratamento antiarrítmico em pacientes com CMH e FA.^1^

**Tabela 21:** Opção de tratamento antiarrítmico em pacientes com CMH e FA^1^

Medicamento	Eficácia	Efeito colateral	Toxicidade	Observação
Propafenona	?	?	Pró-arritmia	Recomendado o uso em pacientes com CDI.
Sotalol	Moderada	Fatiga Bradicardia	QTc prolongado Pró-arritmia	O uso deve ser considerado.
Amiodarona	Moderada-alta	Bradicardia	Fígado, pulmão, tireoide, pele neurológico	O uso deve ser considerado.

#### 4.3.4. Anticoagulação

Os eventos embólicos sistêmicos apresentam alta incidência em pacientes com CMH, especialmente em pacientes com FA, e se correlacionam com o aumento de morbidade e mortalidade nessa população.^261 - 263^

Em um estudo de coorte longitudinal retrospectivo com 4.821 pacientes com CMH e sem histórico de FA e eventos tromboembólicos prévios, foi observada a incidência cumulativa de eventos embólicos, tais como AVC, ataque isquêmico transitório (AIT) e embolia periférica de 2,9 % em 5 anos e 6,4% em 10 anos.^264^

A FA é a arritmia sustentada mais comum em CMH e tem uma prevalência estimada em 20 a 25% nesses pacientes, que é 4 a 6 vezes maior do que a prevalência na população em geral.^28 , 265^ Além disso, a presença de FA se correlaciona a um alto risco de tromboembolismo em pacientes com CMH, sendo a prevalência e incidência anual de eventos de tromboembolismo estimadas em 27,1 e 3,8% respectivamente nesses pacientes.^266 - 269^

Os pacientes com CMH desenvolvem FA em uma idade mais jovem em comparação com a população geral, e a FA apresenta-se na forma paroxística em 2/3 dos casos.^250 , 270^ No entanto, a incidência de tromboembolismo não se difere entre pacientes com CMH e FA paroxística e aqueles com FA permanente/persistente.^250 , 269^ Além disso, o risco de tromboembolismo na FA não se relaciona ao número de paroxismos.^271^

A FA tem baixo risco de mortalidade na CMH, embora episódios paroxísticos possam prejudicar a qualidade de vida.^33^ Durante muito tempo, o surgimento de FA em pacientes com CMH foi considerado um marco de aumento de mortalidade e morbidade, particularmente quando associado com obstrução de VSVE, dada a perda de contribuição atrial para enchimento ventricular.^28 , 250 , 272^ No entanto, muitos desses dados que embasavam essa correlação incluíam tratamentos mais antigos, em que a anticoagulação era pouco aplicada, a amiodarona era o único antiarrítmico utilizado e muito antes do uso mais amplo da ablação de FA por cateter e do uso dos novos agentes anticoagulantes orais diretos (DOAC).^250^ As análises mais recentes de pacientes com o tratamento contemporâneo da CMH não consideram mais o surgimento de FA como um fator independente de aumento de morbidade e mortalidade.^27 , 33^ O DAE também foi considerado um fator de risco para tromboembolismo, associado também com o desenvolvimento da FA.^272^ Em pacientes com CMH sem FA, estudos sugerem que cada aumento de 1 mm no DAE aumente o risco de morte relacionada ao AVC, além de o risco de tromboembolismo aumentar exponencialmente, aumentando o DAE a até 45 a 50 mm.^272^

Outros fatores associados ao risco de fenômenos embólicos em pacientes com CMH foram: idade avançada, presença de sintomas e sinais de IC e a extensão da área de realce tardio do gadolínio.^262 , 272 , 273^ Uma extensão de área de realce tardio > 14,4% na RMC foi considerada um preditor independente para eventos tromboembólicos em pacientes com CMH.^274^ Além disso, alguns fatores associados ao surgimento de FA, como OTSVE, movimento anterior sistólico e RM também podem aumentar o risco tromboembólico.^275^

Os eventos embólicos podem ser prevenidos com anticoagulação oral iniciada após o primeiro episódio de FA. Um limiar baixo para a anticoagulação oral para profilaxia de eventos tromboembólicos é justificado em pacientes com CMH, geralmente seguindo o primeiro episódio de FA registrada em ECG de superfície.^26 - 28 , 276^ Os escores e modelos preditivos de tromboembolismo não se correlacionam bem com o resultado clínico de pacientes com CMH,^249 , 272 , 277^ e não há sistema de pontuação completamente de acordo disponível ainda.^278^ Os escores de CHADS2 e CHA2DS2-VASC funcionaram mal em pacientes com CMH, e novos modelos preditivos para estratificação de risco foram criados, porém esses modelos não têm validações externas.^279^ Atualmente, a medida terapêutica mais importante é o uso de anticoagulação ao longo da vida em todos os pacientes que têm CMH e que tenham apresentado pelo menos um único episódio de FA, mesmo que o ritmo sinusal seja restaurado.^3^

A administração de varfarina e, mais recentemente, o uso dos DOACs, como dabigatrana, rivaroxabana, apixabana e edoxabana, têm contribuído muito para a redução dos eventos embólicos periféricos, AVC e a morte (agora < 1%/ano).^33^ Um estudo recente encontrou que os DOACS são pelo menos tão seguros e eficazes quanto à varfarina em pacientes com CHM submetidos à ablação do cateter para FA.^280^

No entanto, o subtratamento da terapia de anticoagulação oral é uma constante preocupação na prática clínica. Dados do mundo real demostraram que o uso de anticoagulantes orais (ACO) em FA com CMH foram abaixo do ideal, com apenas 15,3% dos pacientes usando ACO no momento do diagnóstico de FA, e apenas 61,8%, durante o período do estudo, receberam ACO.^266^ Episódios assintomáticos de FA detectados por acaso através do monitoramento ambulatorial são comuns nos pacientes com CMH (em 25% dos pacientes com dispositivos implantados), e a carga global de FA em uma população de CMH é provavelmente subestimada.^33^ No entanto, não se sabe ao certo quais são as implicações clínicas de episódios de FA de curta duração em pacientes assintomáticos, embora sejam preditivos de FA sintomática no futuro.^250^

Sabe-se que, em pacientes assintomáticos e FA subclínica detectada em dispositivos implantáveis e com duração menor do que 24 horas, o risco de AVC e embolia sistêmica é apenas metade do risco de pacientes com FA clínica detectada em ECGs de superfície.^281^ Entretanto, o risco de sangramento continua sendo alto nessa população. Desse modo, os dados atuais são insuficientes para justificar, de maneira geral, a anticoagulação oral de rotina para episódios de FA subclínica e com menos de 24 horas de duração. Estudos em andamento, como o ARTESIA e o NOAH, trarão informações relevantes para esse cenário clínico.^282 , 283^ Na ausência de dados específicos para pacientes com FA subclínica e CMH, devemos individualizar cada caso para a decisão sobre anticoagulação oral nesse cenário clínico, e os resultados dos estudos em andamento na população geral com FA subclínica poderão ajudar na conduta dos pacientes com CMH.

Pacientes com CMH e aneurisma apical do VE têm alto risco de morte súbita arrítmica e eventos tromboembólicos.^284^ A identificação desse fenótipo amplia a estratificação de risco e pode levar a intervenções de tratamento para complicações potencialmente fatais. Uma proporção substancial desses pacientes apresenta formação de trombo dentro do aneurisma ou sofreu um evento tromboembólico mesmo nos casos que tinham apenas pequenos aneurismas.^78 , 285^ Essa observação sugere que a área do aneurisma apical acinético pode favorecer a formação de trombo intracavitário, independentemente do tamanho, levantando forte consideração para anticoagulação em todos os pacientes com aneurismas apicais.^286^ Em uma coorte de pacientes com CMH e aneurisma apical, não ocorreram eventos embólicos ao longo do período de acompanhamento em pacientes que recebem ACO.^78^

A Tabela 22 contempla as recomendações para anticoagulação nos pacientes com CMH e FA.

**Tabela 22:** Recomendações para anticoagulação nos pacientes com CMH e FA.

Recomendações	Classe de recomendação	Nível de evidência
A identificação de um ou mais episódios de FA sintomáticos em eletrocardiograma de superfície é suficiente para recomendar anticoagulação oral com DOAC (ou varfarina) após a avaliação dos riscos individuais dos pacientes para esses medicamentos.	I	B
A anticoagulação é recomendada com DOAC (1ª linha) ou antagonista da vitamina K (2ª linha), independentemente do escore de CHA2DS2VASC.	I	B
Em portadores de CMH e FA assintomática detectada em dispositivos internos ou externos, com duração maior que 24 horas, a anticoagulação é recomendada com DOAC (1ª linha) e antagonista da vitamina K (2ª linha), independentemente do escore de CHA2DS2VASC.	I	C
O uso de anticoagulante oral para profilaxia de eventos embólicos é recomendado em pacientes com CMH e aneurisma apical de VE, independentemente do tamanho do aneurisma após avaliação dos riscos individuais dos pacientes para uso de anticoagulantes orais.	I	B
Em portadores de CMH e FA assintomática detectada em dispositivos internos ou externos, com duração maior que 5 minutos e menor que 24 horas, a anticoagulação com DOAC (1ª linha) ou antagonista da vitamina K (2ª linha) deve ser considerada, sempre considerando a carga total de FA, a duração dos episódios, os fatores de risco para tromboembolismo e o risco de sangramento do paciente.	IIa	C
Agentes antiplaquetários não fornecem a prevenção adequada para acidente vascular cerebral no paciente com CMH e FA e, portanto, devem ser evitados.	III	B

FA: fibrilação atrial; DOAC: anticoagulantes orais diretos; CMH: cardiomiopatia hipertrófica; VE: ventrículo esquerdo.

### 4.4. CMH em Estágio Avançado de Insuficiência Cardíaca e Transplante Cardíaco

#### 4.4.1. Definição

Pacientes com CMH podem apresentar progressão da doença em dois fenótipos distintos: obstrutivo (provocada ou em repouso) e não obstrutivo. Embora incomum, com prevalência de 2 a 3% dos pacientes em centros de referência para CMH, a IC é responsável pela maioria dos óbitos (2/3 dos óbitos).^33^ A taxa de progressão para IC em classe funcional (NYHA) III/IV foi estimada em 1,6% ao ano para a forma não obstrutiva, 3,2% para obstrução provocada e 7,4% para obstrução em repouso.^33^ A CMH não obstrutiva é comum e usualmente bem tolerada, porém uma minoria de pacientes progride para IC avançada.^33^

O fenótipo obstrutivo, provocado ou de repouso, resulta de obstrução dinâmica grave associada a VE hiperdinâmico com pouca ou quase nenhuma fibrose. Essa forma pode ser revertida com terapias de redução septal.^11^

No outro fenótipo, a IC pode ser causada por disfunção ventricular esquerda progressiva associada à substituição do tecido miocárdico por fibrose extensa e remodelamento da câmara. Esse cenário, embora não tão frequente, resulta em IC avançada, é irreversível, apresenta manejo desafiador e, em geral, tem resposta ruim ao tratamento farmacológico convencional. Em muitos casos, com a progressão da doença, o transplante cardíaco, assim como outras terapias avançadas, deve ser considerado.^33^

Por outro lado, pacientes jovens com CMH não obstrutiva também podem desenvolver IC restritiva grave, uma forma de IC avançada, que requer tratamento agressivo apesar de manter FE maior do que 50%. Apesar do tratamento, esses pacientes podem desenvolver sintomas de IC refratários à terapia padrão e, nesse contexto, tornam-se potenciais candidatos ao transplante cardíaco.^287^

A CMH é raramente a etiologia em pacientes hospitalizados com IC descompensada com mortalidade de 3,4%. De 1.217.039 pacientes hospitalizados com IC descompensada ou choque cardiogênico, em aproximadamente 6.040 pacientes a etiologia foi CMH (0,5%).^288^

Dessa forma, o diagnóstico de IC avançada em pacientes com CMH está relacionado à presença de sintomas refratários ao tratamento clínico padrão tanto em pacientes que evoluíram com disfunção ventricular quanto naqueles que desenvolveram padrão restritivo grave mantendo FE preservada.^2^

A mais recente atualização da diretriz de CMH das sociedades americana e europeia de IC^289^ respeita os critérios que definem IC avançada descritos por Metra et al.,^290^ que incluem sintomas refratários (NYHA III/IV) associado à disfunção de VE grave, ou sintomas refratários associados à elevação persistente de PN em pacientes com disfunção diastólica grave. Tal definição também foi incorporada à atualização da diretriz de IC da Sociedade Brasileira de Cardiologia e inclui tanto pacientes com cardiomiopatia dilatada quanto restritiva.^291^

#### 4.4.2. Manejo Clínico

##### 4.4.2.1. Terapia Farmacológica

Na IC avançada secundária à CMH terminal com evolução para disfunção sistólica, o tratamento sugerido segue as diretrizes de IC, baseando-se em drogas como: inibidores da enzima conversora de angiotensina/bloqueadores dos receptores da angiotensina II, betabloqueadores, espironolactona e agentes diuréticos. Além disso, deve-se considerar a interrupção de agentes inotrópicos negativos, como verapamil e diltiazem, ou a troca por betabloqueadores. Há poucos relatos com uso de sacubitril valsartana e iSGLT2 nesse contexto.^12^ De forma geral, a terapia habitual de IC com FE reduzida reduz sintomas, porém não afeta o remodelamento e não altera o curso da doença.

Em pacientes com retardo na condução intraventricular, a terapia de ressincronização cardíaca pode reduzir os sintomas e até mesmo aumentar a FE.^292^

A piora funcional (NYHA III/IV) na CMH pode ocorrer sem acometimento da FE (> 50%) em até 50% dos pacientes. Nesses casos, há importante acometimento diastólico com fisiologia restritiva e pouca dilatação ventricular.^11 , 14^ Sua abordagem clínica em geral não difere das terapias já previamente implementadas para o controle da doença com betabloqueadores etc. No entanto, o uso de diuréticos de alça tem papel importante no controle da volemia e congestão nesses pacientes.^287^

Em diversos fenótipos, é importante a observação da progressiva disfunção atrial esquerda e sua interface com a FA. Esta última, quando presente, tende a exacerbar de forma contundente os sintomas em ambas as apresentações, elevando a frequência cardíaca e a pressão atrial esquerda. Como estratégia, deve-se priorizar a manutenção e/ou o retorno ao ritmo sinusal sempre que possível.^293^

##### 4.4.2.2. Terapia Não Farmacológica

Na CMH com IC avançada, há limitação funcional importante, dessa forma, recomendam-se exercícios leves que não induzam sintomas e, sempre que possível, supervisionados.

As comorbidades, tais como a obesidade, podem acentuar a obstrução do fluxo de saída, IC e causar resposta clínica insatisfatória.^294^ Além de auxiliar no controle sistêmico (muscular esquelético periférico), o controle da obesidade tem o benefício adicional de melhorar a dinâmica respiratória e reduzir as alterações posturais dinâmicas na pré-carga que, nesses pacientes, podem agravar a obstrução do TSVE e provocar pré-síncope ou síncope.^295^

#### 4.4.3. Indicações de Transplante Cardíaco

Pacientes portadores de CMH, refratários ao tratamento clínico medicamentoso, não candidatos a outra intervenção e que apresentam importante comprometimento da qualidade de vida e da expectativa de vida são candidatos ao transplante cardíaco. Nesse contexto, admite-se que principalmente pacientes com disfunção sistólica, refletindo progressão da doença, representem a maioria dos fenótipos. No entanto, a disfunção diastólica grave também pode ser encontrada. Dados recentes da ISHLT estimam de 2 a 3% das indicações de transplante cardíaco, secundária à CMH.

Uma vez que não é comum esses pacientes estarem internados por IC descompensada, nem mesmo em baixo débito necessitando de suporte inotrópico, abre-se a possibilidade de discussão, em nosso meio, sobre a identificação do paciente de maior gravidade e que eventualmente poderia ser priorizado para transplante.

No estado de São Paulo, por deliberação da Câmara Técnica da Secretaria de Saúde, incluem-se os pacientes que estejam internados e com necessidade de diurético endovenoso nesse perfil de prioridade condição 3, ou seja, semelhante ao paciente de outras etiologias, que estejam internados com inotrópicos, por menos de 6 meses.

#### 4.4.4. Indicações de Dispositivo de Assistência Ventricular

As cavidades ventriculares reduzidas e a fisiologia restritiva comum aos pacientes com CMH e IC avançada limitam a indicação dos dispositivos de assistência ventricular esquerda nessa população. Entretanto, em pacientes que possuem contraindicação ao transplante cardíaco ou indisponibilidade de órgão, o implante do dispositivo de assistência ventricular (DAV) pode ser considerado.

Algumas séries de casos em pacientes com IC avançada e CMH demonstraram um aumento da sobrevida após o implante de DAV esquerda de fluxo contínuo em pacientes com diâmetro diastólico do VE acima de 46 mm (46 a 50 mm).^296 - 299^

**Figura 19:**
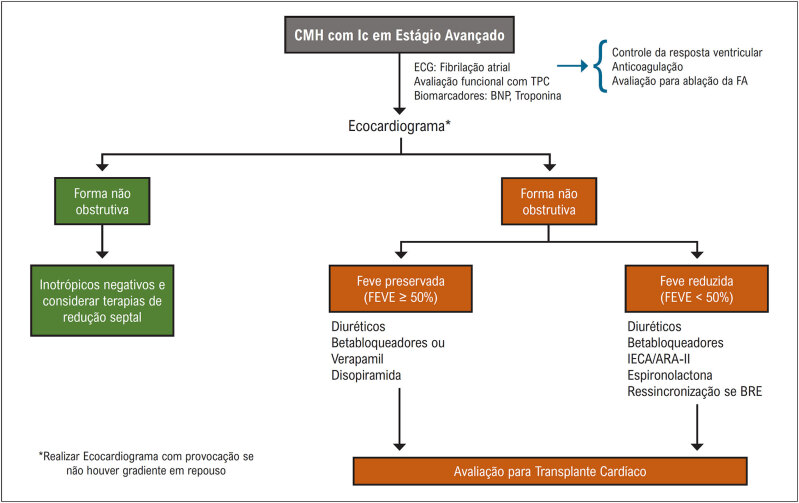
Fluxograma de tratamento da IC avançada na CMH. CMH: cardiomiopatia hipertrófica; IC: insuficiência cardíaca; ECG: eletrocardiograma; TCP: teste cardiopulmonar; BNP: peptídeo natriurético tipo B; FEVE: fração de ejeção ventricular esquerda; FA: fibrilação atrial; iECA: inibidores da enzima de conversão da angiotensina II; ARA-II: antagonistas dos receptores de angiotensina II; BRE: bloqueio de ramo esquerdo; CDI: cardiodesfibrilador implantável.

A Figura 19 ilustra o fluxograma de tratamento da IC avançada na CMH.

A Tabela 23 contempla as recomendações para avaliação diagnóstica, prognóstica e tratamento dos pacientes com CMH e IC avançada.

**Tabela 23:** Recomendações para avaliação diagnóstica, prognóstica e tratamento dos pacientes com CMH e IC avançada

Recomendações	Classe de recomendação	Nível de evidência
O teste cardiopulmonar deve ser realizado em pacientes com CMH com sintomas persistentes para quantificar o grau de limitação funcional e contribuir para a seleção de potenciais candidatos ao transplante cardíaco.	I	B
Pacientes com CMH que desenvolvem disfunção de VE (FEVE < 50%) e IC avançada devem receber tratamento medicamentoso guiado por diretrizes de IC.	I	C
Pacientes com CMH não obstrutiva que evoluem com disfunção de VE (FEVE < 50%) e IC avançada são potenciais candidatos ao transplante cardíaco e devem ser considerados para implante de cardiodesfibrilador implantável.	IIa	B
Pacientes com CMH que desenvolvem disfunção de VE (FEVE < 50%) e IC avançada, a descontinuação de agentes inotrópicos negativos como verapamil ou diltiazem deve ser considerada.	IIa	C^300^
Recomenda-se a consideração de transplante cardíaco para pacientes com CMH não obstrutiva que evoluem com IC avançada com sintomas limitantes ou arritmias malignas refratárias, apesar da terapia otimizada guiada por diretrizes.	I	C
Pacientes com CMH não obstrutiva com disfunção de VE que evoluem com IC avançada com sintomas limitantes refratárias à terapia guiada por diretrizes devem ser considerados para dispositivo de assistência ventricular se houver contraindicação ao transplante cardíaco.	IIa	C

CMH: cardiomiopatia hipertrófica; VE: ventrículo esquerdo; FEVE: fração de ejeção ventricular esquerda; IC: insuficiência cardíaca.

### 4.5. Reabilitação e Exercício Físico

A atividade física desempenha um papel fundamental na prevenção de doenças cardiovasculares. Embora a morte súbita durante a prática de exercício físico seja um evento raro, a CMH ainda é responsável pelo maior número de eventos, traduzindo-se em um desafio à prática clínica para decisão quanto à recomendação de atividade física em um paciente portador de CMH.^301^ Fatores intrínsecos à doença (predisposição genética, estímulo adrenérgico), assim como possíveis fatores extrínsecos (desidratação, uso de esteroides anabolizantes e fatores ambientais) podem predispor à morte súbita.^301^ Para a avaliação pré-participação de atividades físicas desses pacientes, sugere-se uma abordagem relação à estratificação de risco para morte súbita, gradiente VSVE, reposta de pressão ao esforço VE, arritmia ventricular induzida pelo exercício e intensidade de exercício sugerida:^217 , 301^ analisar a Tabela 24 .

**Tabela 24:** Estratificação de risco para prescrição de atividade física de acordo com o perfil de cada paciente

	Assintomático + boa capacidade de exercício	Sintomas relacionados à CMH, mas sem relação com o exercício	História de PCR, síncope ou sintomas durante o exercício
Escore *HCM Risk-SCD* para morte súbita em 5 anos	Baixo risco (< 4%)	Moderado risco (≥ 4% e < 6%)	Alto risco (≥ 6%)
Gradiente de via de saída	Ausente/baixo (< 30 mmHg no repouso/exercício)	Moderado (30 a 49 mmHg no repouso ou exercício)	Alto gradiente (> 50 mmHg no repouso ou exercício)
Resposta da PA ao exercício	Normal	Atenuada (< 20 mmHg de incremento na PA sistólica)	Queda da PA no esforço
Arritmia induzida pelo exercício	Sem arritmias	Extrassístoles ventriculares no exercício	TVNS ou TVS no esforço
Intensidade de exercício sugerida	Alta intensidade	Moderada intensidade	Baixa intensidade

PA: pressão arterial; PCR: parada cardiorrespiratória; TVNS: taquicardia ventricular não sustentada; TVS: taquicardia ventricular sustentada; CMH: cardiomiopatia hipertrófica. Adaptado de Semsarian et al.^301^

A prática de atividade física aeróbica em intensidade moderada está associada com melhora na saúde cardiovascular e sobrevida na população.^302^ Apesar de, historicamente, a prática de atividade física entre paciente com CMH tenha sido proscrita, diversas iniciativas avaliaram a segurança da atividade física nesses pacientes em ambulatórios controlados e monitorizados.^303 , 304^ Experiência inicial com 20 pacientes em centro único demonstrou melhora de pelo menos uma classe funcional em 50% e de capacidade funcional em programa de reabilitação.^303^ Em outro estudo observacional recente, 32 indivíduos com CMH completaram ao menos 3 meses em programa de reabilitação com benefício em capacidade funcional.^304^

Em um ensaio clínico randomizado, foram incluídos indivíduos com a doença entre 18 e 80 anos de idade, sendo excluídos aqueles com síncope no exercício ou história de taquicardia ventricular, obstrução na VSVE refratário ao tratamento clínico (em avaliação para terapia de redução septal), história de hipotensão (queda > 20 mmHg de redução da pressão arterial sistólica), descompensação cardíaca ou implante de CDI ou terapia de redução septal nos últimos 3 meses, FE < 55%, entre outros. Os pacientes foram randomizados para atividade física usual ou reabilitação cardíaca. Os resultados demonstraram incremento de 1,35 (IC95% 0,50-2,21) mL/kg/min no VO_2_ no grupo-treinamento, com diferença entre os grupos de 1,27 (IC95% 0,17-2,37) mL/kg/min em 16 semanas de treinamento. É importante salientar que não foram registrados eventos adversos incluindo morte, morte súbita abortada, choque apropriado pelo CDI ou TVS em nenhum dos grupos, embora o estudo não tivesse poder para avaliar segurança.^300^ Desse modo, a atividade física não supervisionada poderá ser benéfica e segura em pacientes com CMH sem marcadores de risco de morte súbita. Em indivíduos com resposta anormal da pressão arterial ao exercício ou arritmias, como mencionado acima, a atividade física deverá ser realizada, preferencialmente, em ambiente supervisionado.^300 , 303 , 304^

A Tabela 25 contempla as recomendações para a prática de atividade física em pacientes com CMH.

**Tabela 25:** Recomendações para prática de atividade física em pacientes com CMH

Recomendações	Classe de recomendação	Nível de evidência
Recomenda-se a estratificação de risco para a classificação dos pacientes quanto à prescrição de atividade física usando escores específicos e parâmetros observados no esforço.	I	C
A reabilitação cardiopulmonar com treinamento estruturado e não supervisionado é recomendada para pacientes com CMH com intuito de melhora de capacidade funcional em indivíduos de baixo risco.	I	B
A reabilitação cardiopulmonar em paciente com CMH na presença de gradiente na VSVE ≥ 50 mmHg em repouso/Valsalva, em ambiente supervisionado poderá ser considerada	IIb	C
Não se recomenda a reabilitação cardiopulmonar de alta ou moderada intensidade em indivíduos com CMH e história prévia de síncope aos esforços, taquicardia ventricular ou morte súbita abortada.	III	C^301^

CMH: cardiomiopatia hipertrófica; VSVE: via de saída do ventrículo esquerdo.

### 4.6. Avaliação Perioperatória em Cirurgias Não Cardíacas

Pacientes com CMH apresentam maior risco de complicações cardíacas no período perioperatório de cirurgias não cardíacas. As evidências epidemiológicas são escassas e conflitantes no que diz respeito à mortalidade, mas inequivocamente identificam maior risco de complicações cardiovasculares nos pacientes com essa condição. Em série norte-americana retrospectiva, a incidência de infarto do miocárdio e óbito após cirurgia não cardíaca foi comparada entre 227 pacientes com CMH e 554 indivíduos sem CMH, pareados de acordo com ano da operação, sexo e idade.^1 , 305^ Após o ajuste para fatores como histórico de arritmia, IC, doença coronária, diabetes e hipertensão, o risco de infarto ou óbito foi quase o triplo para pacientes com CMH [ *odds ratio* (OR): 2,82, IC95% 2,59–3,07] e o risco de morte foi 61% maior (OR 1,61, IC95% 1,46–1,77).^305^

Em outras casuísticas, não foi observada maior mortalidade, mas consistentemente observou-se maior incidência de complicação cardiovascular, notadamente, descompensação de IC.^306 , 307^

O racional para complicações cardiovasculares perioperatórias em pacientes com CMH é bastante plausível, e o perioperatório representa um desafio hemodinâmico para pacientes com CMH. O *shift* volêmico e a vasodilatação periférica, com oscilações em pré- e pós-carga, podem aumentar gradiente de VSVE ou até mesmo provocar gradiente em pacientes com CMH sem obstrução ao exame de repouso. Além disso, oscilações pressóricas e hematimétricas perioperatórias podem instabilizar o já difícil equilíbrio entre oferta e consumo miocárdico de oxigênio dos pacientes com CMH. Esses pacientes já apresentam, em sua condição basal, maior demanda de oxigênio pelo miocárdio hipertrofiado e oferta de oxigênio prejudicada pela elevada pressão diastólica final em VE, ou mesmo por doença coronária concomitante. Há ainda maior risco de arritmias por estímulo adrenérgico, piora da repercussão hemodinâmica ou desequilíbrio eletrolítico.

Não há estudos de intervenção no perioperatório de cirurgia não cardíaca de pacientes com CMH para embasar recomendações específicas para esse contexto. Entretanto, a estratificação de risco e a terapia clínica devem ser otimizadas antes de cirurgias eletivas. Idealmente, pacientes com CMH devem ser operados em centros de referência, com equipe de anestesiologista, intensivista e cardiologista experiente no manejo da cardiopatia. Pacientes que não têm ecodopplercardiograma realizado no último ano ou que tiveram mudança de sintomas nesse período devem repeti-lo antes de procedimentos com risco intrínseco moderado ou alto. ECG de 12 derivações, troponina-US e BNP também devem ser realizados, mesmo para pacientes em classe funcional I ou II, tornando-se parâmetros de monitorização pós-operatória, no caso de operações com risco intrínseco intermediário ou alto. Os medicamentos usados para controle da CMH, notadamente os betabloqueadores, devem ser mantidos no perioperatório. Pacientes com CMH devem ser avaliados com relação ao estado volêmico e, idealmente, devem ser operados no primeiro horário do dia, evitando longos períodos de jejum e possível hipovolemia por desidratação. Para os procedimentos de longa duração e/ou grande *shift* volêmico, a monitorização hemodinâmica com pressão arterial invasiva e cateter venoso central é mandatória, propiciando terapia hemodinâmica guiada por alvos. Em centros experientes, deve-se considerar o uso de ecodopplercardiograma transesofágico transoperatório para melhor avaliação de volemia e de alterações no gradiente intraventricular. A monitorização deve continuar após o término da cirurgia, necessariamente nas primeiras 48 horas, com reavaliação cardiológica e dosagem diária de BNP e troponina-us para detecção precoce de descompensação.

Técnicas anestésicas específicas, como utilização de agentes com menor impacto hemodinâmico, devem ser observadas desde a indução anestésica, porém a preferência por anestesia geral ou regional é bastante polêmica. Nas casuísticas previamente descritas, não se observou associação entre anestesia geral ou regional e desfechos adversos, mas o potencial de queda abrupta de retorno venoso e vasodilatação periférica associada a bloqueios periféricos faz essa técnica menos atraente para pacientes com CMH, notadamente, aqueles sintomáticos e com gradientes expressivos na VSVE. Na casuística de Dhillon et al., em centro de referência para CMH, a anestesia geral foi utilizada em 89% dos pacientes.^307^

Por fim, a avaliação perioperatória também constitui oportunidade para o diagnóstico de CMH. Para procedimentos eletivos, recomenda-se primeiro a estratificação do risco e tratamento específico (vide tópicos dedicados dessa mesma diretriz) antes da operação não cardíaca.

A Tabela 26 contempla as recomendações para a avaliação perioperatória em cirurgias não cardíacas em pacientes com CMH.

**Tabela 26:** Recomendações para a avaliação perioperatória em cirurgias não cardíacas em pacientes com CMH

Recomendações	Classe de recomendação	Nível de evidência
É recomendada a realização de ECO nos casos de suspeita de CMH, em casos em que o último ECO foi há mais de 1 ano e/ou com mudança de sintomas.	I	C
Recomenda-se manter o betabloqueador em todo perioperatório.	I	C
Recomenda-se a realização de eletrocardiograma, BNP ou NT-proBNP e troponina antes de operações de risco intermediário a alto, e repeti-los no PO imediato e 1 vez ao dia no 1° e 2° dia PO.	I	C
Deve ser considerada a monitorização hemodinâmica com pressão arterial invasiva, catéter venoso central e terapia hemodinâmica guiada por alvo durante cirurgias de alto risco intrínseco.	IIa	C
Pode ser considerada a realização de eco transesofágico durante a operação com alto risco para a avaliação de volemia e gradiente intraventricular em centros com experiência.	IIb	C

ECO: ecocardiograma; CMH: cardiomiopatia hipertrófica; BNP: peptídeo natriurético tipo B; NT-proBNP: fração N-terminal do peptídeo natriurético do tipo B; PO: pós-operatório.

### 4.7. Centros de Referência Multidisciplinares em Cardiomiopatia Hipertrófica

A CMH é uma doença complexa com espectro clínico e genético muito amplo. Apesar de ser a cardiopatia genética mais frequente e ser relativamente comum na população geral, a CMH não é habitual na prática clínica para a maioria dos cardiologistas.^308 , 309^ Muitas vezes, casos sintomáticos são erroneamente manejados, levando de meses até anos para se chegar ao diagnóstico correto.^310^ Além disso, a complexidade e grande variabilidade de apresentação clínica pode acarretar dificuldades no manuseio dos pacientes, especialmente aqueles mais graves. Dessa forma, deve ser considerado o encaminhamento desses pacientes para centros de referência que dispõem de protocolos de excelência para diagnóstico e tratamento da CMH ( Tabela 27 ). Neles, devem ser oferecidos atendimentos especializados e intervenções terapêuticas avançadas ( Tabela 28 ), incluindo testes e aconselhamentos genéticos, implante de cardiodesfibrilador, ecocardiograma com exercício físico, RMC, ablação septal com álcool, miectomia septal cirúrgica, manuseio de arritmias, permitindo, assim, escolhas mais adequadas aos pacientes e aos seus familiares, subsidiadas nas melhores evidências disponíveis.^308 , 309^ Os centros de tratamento especializado possibilitam, também, aos profissionais o ganho de *expertise* , ao cuidar de um grande volume de portadores de CMH, além de possibilitar aos pacientes a inclusão em protocolos de pesquisa multicêntricos testando novas terapias.

**Tabela 27:** Recomendações sobre encaminhamento e constituição dos centros de referência em cardiomiopatia hipertrófica

Recomendações	Classe de recomendação	Nível de evidência
A tomada de conduta sobre terapias avançadas em pacientes com CMH deve ser feita mediante discussão em *Heart Team* multidisciplinar.	I	C
Deve ser considerado o encaminhamento ou acompanhamento conjunto para um centro de referência dos pacientes com diagnóstico de CMH necessitando abordagem diagnóstica e/ou terapêutica especializada.	IIa	C
Recomenda-se o encaminhamento dos pacientes necessitando de terapia de redução septal para centro de referência com excelência de resultados dessas intervenções	I	C

CMH: cardiomiopatia hipertrófica.

**Tabela 28:** Profissionais participantes do Heart Team e procedimentos a serem oferecidos em centros de referência em cardiomiopatia hipertrófica

Médicos	Cardiologista (adulto e pediátrico)
	Especialista em ecocardiografia
	Especialista em ressonância magnética cardíaca e tomografia computadorizada cardíaca
	Especialista em eletrofisiologia
	Especialista em marca-passo e CDI
	Cirurgião cardíaco
	Cardiologista intervencionista
	Geneticista
Outros profissionais	Enfermeiro(a)
	Psicólogo(a)
	Nutricionista
	Assistente social
	Ginecologista – obstetra
Métodos diagnósticos	Análise genética
	Aconselhamento genético
	Ecocardiograma em repouso e exercício
	Ressonância magnética cardíaca
	Tomografia computadorizada cardíaca
	*Holter* de 24 horas
	Monitor de evento arrítmicos de longa permanência
	Estudo eletrofisiológico
	Teste ergométrico
	Teste cardiopulmonar
	Cineangiocoronariografia
	Estudo hemodinâmico invasivo
Procedimentos terapêuticos	Miectomia septal cirúrgica
	Ablação septal com álcool
	Ablação de FA
	Implante de CDI

CDI: cardiodesfibrilador implantável; FA: fibrilação atrial.

Um dos aspectos a ser considerado no encaminhamento dos pacientes é a identificação da necessidade de terapia de redução septal, que apresenta melhores resultados e baixas taxas de mortalidade e complicações em centros de referência com experiência nesses procedimentos ( Tabela 27 ).^310^

A multidisciplinaridade é essencial para a organização adequada de um centro de referência em CMH, dentro do conceito de *Heart Team* , sendo indicada a participação de profissionais treinados, listados na Tabela 28 .^308 , 309^

Em termos mais amplos, o propósito fundamental dos profissionais envolvidos no centro de referência em CMH, assim como em outras atividades dos serviços de saúde no Brasil, é oferecer a todo paciente o "cuidado certo", ou seja: eficaz (pertinente e embasado em evidências científicas), para o doente certo, na hora certa (tempo oportuno), da maneira certa (profissionais treinados e com times multidisciplinares), gerando o resultado certo (redução de desfechos clínicos relevantes), promovendo o engajamento e decisões compartilhadas envolvendo o paciente e sua família de forma eficiente e, mediante tudo isso, promover valor em saúde.^311^

Além desses aspectos, os centros de referência em CMH, na visão do GEMIC/DEIC/SBC, devem estar compromissados em transformar o cuidado dos pacientes com CMH, conectando educação/treinamento dos profissionais com a defesa e educação do público leigo para a doença, com envolvimento de pacientes, familiares e associações de pacientes, além de fomentar a pesquisa clínica e constituir-se em local para incorporação de novas tecnologias, desenvolver núcleos de abordagem translacional, medicina digital (telecardiologia), além de incorporar boas práticas de gestão (protocolos, indicadores, ferramentas de melhoria contínua da qualidade e compromisso com a cultura de segurança do paciente), divulgando seus resultados e buscando certificações, envolvendo-se com registros nacionais e internacionais e desenvolvimento de *networking* .

### 4.8. Situações Clínicas Especiais na Cardiomiopatia Hipertrófica

#### 4.8.1. CMH na Criança e no Adolescente

Embora o entendimento dos mecanismos genéticos, moleculares e fisiológicos da CMH tenha crescido significativamente, as estratégias de avaliação e manejos clínico-cirúrgicos ideais ainda são insuficientes na população pediátrica. De particular interesse é a ampla variabilidade de apresentação clínica na faixa etária pediátrica.

Apresenta uma incidência estimada de 0,24 a 0,47 para 100.000 crianças ao ano, correspondendo de 25 a 50% de todas as miocardiopatias diagnosticadas na infância e adolescência. Constitui o segundo fenótipo mais observado na pediatria segundo registros realizados na América do Norte, Finlândia e Austrália.^312 - 314^ Tem prevalência menor que o da população adulta.^315 , 316^

Existe um pico de incidência no primeiro ano de vida, em que a CMH é três vezes mais diagnosticada que em outras faixas etárias pediátricas, seguido por um segundo pico na adolescência.^312 , 314 , 316 - 318^

A CMH é uma doença de herança autossômica dominante com expressividade variável e de penetrância relacionada à idade de aparecimento do fenótipo/sintomas. Embora a etiologia da CMH seja heterogênea e inclua erros inatos do metabolismo, doenças neuromusculares e síndromes com malformações, na faixa etária pediátrica, a apresentação clínica da CMH decorre, sobretudo, das variantes patogênicas em genes que codificam proteínas do sarcômero.^318 - 321^ Quando a CMH é diagnosticada em lactentes com idade inferior a 1 ano, é menos comum a presença de história familiar positiva ou a detecção de variantes sarcoméricas patogênicas ou provavelmente patogênicas, diferente do observado quando a apresentação ocorre entre crianças e adolescentes.^316^

##### 4.8.1.1. História Natural da CMH na Infância e Adolescência

Devido à raridade da incidência da CMH na pediatria, sua história natural ainda não é bem compreendida.^316^

Na infância, sua evolução pode ser progressiva e apresentar fenótipo completo apenas entre a segunda e terceira décadas de vida.^322^ O diagnóstico no 1º ano de vida está associado com um prognóstico substancialmente pior comparado com diagnóstico após a idade de 1 ano.^321^ A mortalidade anual da CMH é maior do que aquela nos adultos, e mais da metade são de morte súbita em pacientes assintomáticos.

A apresentação clínica depende da causa subjacente, e achados observados na avaliação auxiliam na investigação etiológica.^318 - 320^ Algumas características de fenótipos hipertróficos decorrentes de erros inatos do metabolismo e síndromes associadas a malformações podem ser observadas em uma anamnese bem feita e exame físico detalhado. Estas são diagnosticadas mais frequentemente em lactentes e acompanhadas de atrasos nos marcos do desenvolvimento, com alterações cognitivas, além da presença de dismorfias e comprometimento de outros órgãos e sistemas como alterações neurológicas, hepáticas e renais.^319 , 320^ Identificar essas etiologias é fundamental para direcionar terapias disponíveis e determinar seu prognóstico.^318 , 323 , 324^

Em estudo realizado por Marston et al.,^316^ buscou-se caracterizar a história natural da CMH através da análise de uma coorte multicêntrica de 1.128 crianças e adolescentes com idade entre 1 e 18 anos obtida a partir do estudo ShaRe ( *The Sarcomeric Human Cardiomyopathy Registry* ). Observou-se que os pacientes diagnosticados com CMH sarcomérica nessa faixa etária apresentaram maior risco de desenvolver arritmias ventriculares e necessidade de tratamento avançado para a IC. Além disso, a presença de uma variante sarcomérica no teste genético associa-se a risco elevado de 67% para a ocorrência de desfechos cardíacos, com risco duas vezes maior de desenvolver IC.

A MSC e a IC avançada são as principais complicações nos pacientes com CMH, sendo a morte súbita particularmente comum em adolescentes e adultos jovens. Idade maior que 30 anos e uma história familiar de morte súbita prematura são fatores de risco para MSC.

A IC avançada é o curso natural da doença em muitos pacientes. Após a instalação da IC, a progressão para o óbito é muito rápida. Idade jovem ao diagnóstico, uma história familiar de CMH e a maior espessura da parede estão associadas com uma maior probabilidade de desenvolvimento de IC avançada.

A morte súbita é a principal causa de óbito em crianças com o fenótipo de CMH, sobretudo em adolescentes e adultos jovens e, em menor frequência, em adultos,^217^ podendo acontecer em pacientes previamente hígidos.^320^ Há uma associação positiva da idade com o risco de morte súbita, o que se correlaciona com uma maior penetrância da morte súbita entre adolescentes.^325^

Classicamente, são considerados marcadores de risco de morte súbita: a síncope inexplicada, história familiar de morte súbita relacionada a MCH, hipertrofia maciça do ventrículo e TVNS.^33^ Em metanálise realizada por Xia et al., observou-se que a presença de síncope, TVNS e a obstrução da VSVE estão associadas ao risco de morte súbita em crianças com MCH.^326^ Por outro lado, a história familiar de morte súbita não foi fator de risco, diferente do observado no *Guideline* da Sociedade Europeia de Cardiologia (ESC).^326 , 327^

A identificação de pacientes com benefício de implante de CDI para a prevenção primária da morte súbita ainda é um desafio na pediatria. O escore de risco da ESC não é utilizado para pacientes com idade inferior a 16 anos.^33 , 325^ Recentemente, Norrish et al.^327^ propuseram uma calculadora de risco ( *HCM Risk-Kids* – HCM Risk-Kids model ) utilizando-se variáveis não invasivas como: síncope inexplicada, espessura máxima da parede do VE, diâmetro AE, gradiente na VSVE e TVNS. Recentemente, foi realizada validação externa desse método; que mostrou que o *HCM Risk-Kids* fornece ferramenta validada de avaliação individualizada para crianças com MCH, podendo guiar a decisão terapêutica no implante do CDI.^217^

#### 4.8.2. Gravidez e Parto nos Pacientes com CMH

A gravidez representa um potencial risco às mulheres com CMH, porque, a depender da condição patogênica da doença, o aumento do débito cardíaco, a redução da resistência vascular periférica^328^ e o estado de hipercoagulabilidade materna,^329^ quaisquer deles fisiológicos da gestação, propiciam complicações tais como: IC, arritmias e tromboembolismo.

**1. Aconselhamento reprodutivo** : mulheres portadoras CMH devem planejar a gravidez de acordo com os riscos presumíveis de prognóstico materno-fetal considerando os sintomas, graus de comprometimento do coração (avaliados pelo ETT e/ou RMC), presença de obstrução, terapêutica prévia e o estudo genético.^330 - 332^

**1.A. Avaliação do risco materno** : deve ser apoiada na classificação modificada pela Organização Mundial da Saúde (OMS), que inclui as classes I, II, III e IV-OMS,^333 , 334^ como segue:

- Classe II-OMS (CMH sem obstrução da VSVE e ausência de fatores complicadores): a grande maioria apresenta gravidez com boa evolução e sem complicações;
- Classe III-OMS (CMH com obstrução importante da VSVE, arritmias sintomáticas e/ou disfunção sistólica ventricular moderada): apresenta grande chance de complicações e deve ter a gravidez desaconselhada;
- Classe IV –OMS (CMH com disfunção ventricular ou obstrução de VSVE graves e sintomáticas): elevado risco de complicação e apresentam contraindicação à gestação.

Fatores complicadores, como antecedentes de IC, arritmia ventricular complexa, FA paroxística ou permanente e morte súbita na família, são considerados marcadores de pior prognóstico para a gestação.^335^ Muitas vezes, no planejamento da gravidez surge a oportunidade para uma discussão com especialistas sobre a indicação de CDI ou ablação por cateter de arritmias complexas e/ou sintomáticas, em casos que se incluem na "Classe IA de recomendação". Mulheres submetidas à terapia de redução septal não estão incluídas nas categorias de riscos da OMS devido à falta de dados para tal classificação.

1. **B Avaliação genética** : no planejamento da gravidez, é essencial a consideração sobre a transmissão autossômico dominante mendeliano da CMH, que pode ser causada também por mutações genéticas que codificam os componentes do sarcômero.^336^ A triagem com o uso de testes genéticos ou através da imagem e eletrocardiografia deve ser realizada nesse momento, e a patogenicidade das variantes detectadas deve ser considerada no aconselhamento.
2. **Conduta na gestação** : o seguimento multidisciplinar, a realização de ecocardiograma materno e fetal e a monitorização das arritmias através do sistema *Holter* -24 horas são medidas na rotina durante a gestação de pacientes com CMH.^337^ Caso haja necessidade de tratamento farmacológico, os β-bloqueadores, propranolol ou succinato de metoprolol, associados ou não aos antagonistas do cálcio, preferencialmente o verapamil, são seguros, não teratogênicos e eficazes no controle dos sintomas. As doses diárias e a associação desses fármacos devem ser ajustadas pelos riscos de hipotensão arterial. O CDI deve ser considerado em pacientes que se incluam nas recomendações Classe IA de indicação. A ablação por RF com mapeamento eletroanatômico deve ser indicada em casos de arritmia que propicia instabilidade hemodinâmica com má reposta ao tratamento clínico.^334 , 337^

Pacientes com CMH e FA persistente ou paroxística apresentam risco marcadamente aumentado de AVC, de modo que a anticoagulação oral com a varfarina deve ser considerada, independente do escore CHA2DS2VASc.^338^ Como a FA rápida é, muitas vezes, mal tolerada em pacientes com CMH, a manutenção do ritmo sinusal e o controle da frequência são os principais objetivos do tratamento que podem ser obtidos com a cardioversão elétrica, que não tem contraindicação na gestação.^334 , 337^

**Considerações obstétricas** : a gravidez em portadoras de CMH está associada a recém-nascidos de baixo peso e a prematuridade. O parto vaginal é considerado seguro,^339^ enquanto a cesárea é reservada para as pacientes em classes III/IV -OMS ou para casos em que haja indicação obstétrica, tais como a restrição de crescimento intrauterino e/ou sofrimento fetal. O uso de prostaglandinas para a indução do parto não é aconselhado devido aos efeitos vasodilatadores inerentes desse fármaco. A anestesia com bloqueio pode ser considerada em pacientes na classe II-OMS, enquanto a anestesia geral é particularmente reservada para classe IV-OMS para proporcionar maior segurança materna.

Embora os estudos de genética molecular ao longo da última década tenham contribuído sobremaneira para a compreensão da heterogeneidade clínica e genética da CMH, a complexidade dessa doença ainda não permite a determinação da sua verdadeira incidência em recém-nascidos aparentemente saudáveis que não apresentam anormalidades aos exames de imagens, como o ETT.^2^ De toda forma, o estudo genético de crianças e adolescentes assintomáticos com antecedentes familiares de CMH identificou portadores de mutação "saudáveis". No entanto, existem importantes obstáculos à aplicação clínica das pesquisas genéticas, como a heterogeneidade genética, a baixa frequência com que cada mutação causal ocorre na população geral portadora de CMH e dificuldades metodológicas, como a identificação de uma única mutação patogênica entre 10 genes diferentes e as limitações técnicas laboratoriais.

A Tabela 29 contempla as recomendações para gravidez e parto em portadoras de CMH.

**Tabela 29:** Recomendações para gravidez e parto em portadoras de CMH

Recomendações	Classe de recomendação	Nível de evidência
Recomenda-se o emprego da classificação da OMS-modificada para estratificação de risco da gravidez	I	C
Recomenda-se o uso de betabloqueadores cardiosseletivos e os antagonistas dos canais de cálcio para o controle dos sintomas, e deve-se ter apoio na monitorização do crescimento e vitalidade fetal.	I	C
A cardioversão elétrica deve ser considerada perante a fibrilação atrial persistente ou mal tolerada.	II	C
A anticoagulação com antagonistas da vitamina K ou heparina de baixo peso molecular deve ser considerada na fibrilação atrial, a depender da idade gestacional.	IIA	C
O parto vaginal é a primeira escolha para pacientes em Classe I/II-OMSm, e o parto cesárea deve ser considerado para Classes III/IV-OMSm.	IIA	C

OMS: Organização Mundial da Saúde.
