# Supplementary material for: Diretriz sobre Diagnóstico e Tratamento da Cardiomiopatia Hipertrófica – 2024
Source: Arq Bras Cardiol. 2024 Jun 20;121(7):e202400415. [Article in Portuguese] doi: 10.36660/abc.20240415 (PMC12092053; doi:10.36660/abc.20240415)
Supplement: Translation

# Translation: Guidelines on the Diagnosis and Treatment of Hypertrophic Cardiomyopathy - 2024

**DOI:** 10.36660/abc.20240415i

**Authors:** Fabio Fernandes, Marcus V. Simões, Edileide de Barros Correia, Fabiana Goulart Marcondes-Braga, Otavio Rizzi Coelho, Cláudio Tinoco Mesquita, Wilson Mathias, Murillo de Oliveira Antunes, Edmundo Arteaga-Fernández, Carlos Eduardo Rochitte, Felix José Alvarez Ramires, Silvia Marinho Martins Alves, Marcelo Westerlund Montera, Renato Delascio Lopes, Mucio Tavares de Oliveira, Fernando Luis Scolari, Walkiria Samuel Avila, Manoel Fernandes Canesin, Edimar Alcides Bocchi, Fernando Bacal, Lidia Zytynski Moura, Eduardo Benchimol Saad, Mauricio Ibrahim Scanavacca, Bruno Pereira Valdigem, Manuel Nicolas Cano, Alexandre Antonio Cunha Abizaid, Henrique Barbosa Ribeiro, Pedro Alves Lemos, Gustavo Calado de Aguiar Ribeiro, Fabio Biscegli Jatene, Ricardo Ribeiro Dias, Luis Beck-da-Silva, Luis Eduardo Paim Rohde, Marcelo Imbroinise Bittencourt, Alexandre da Costa Pereira, José Eduardo Krieger, Humberto Villacorta, Wolney de Andrade Martins, José Albuquerque de Figueiredo, Juliano Novaes Cardoso, Carlos Alberto Pastore, Ieda Biscegli Jatene, Ana Cristina Sayuri Tanaka, Viviane Tiemi Hotta, Minna Moreira Dias Romano, Denilson Campos de Albuquerque, Ricardo Mourilhe-Rocha, Ludhmila Abrahão Hajjar, Fabio Sandoli de Brito, Bruno Caramelli, Daniela Calderaro, Pedro Silvio Farsky, Alexandre Siciliano Colafranceschi, Ibraim Masciarelli Francisco Pinto, Marcelo Luiz Campos Vieira, Luiz Claudio Danzmann, Silvio Henrique Barberato, Charles Mady, Martino Martinelli, Ana Flavia Malheiros Torbey, Pedro Vellosa Schwartzmann, Ariane Vieira Scarlatelli Macedo, Silvia Moreira Ayub Ferreira, Andre Schmidt, Marcelo Dantas Tavares de Melo, Moysés Oliveira Lima, Andrei C. Sposito, Flávio de Souza Brito, Andreia Biolo, Vagner Madrini, Stephanie Itala Rizk, Evandro Tinoco Mesquita

**Affiliations:** 1 São Paulo SP Brazil Instituto do Coração (InCor) do Hospital das Clínicas da Faculdade de Medicina da Universidade de São Paulo (HCFMUSP), São Paulo, SP - Brazil; 2 Ribeirão Preto SP Brazil Faculdade de Medicina de Ribeirão Preto da Universidade de São Paulo, Ribeirão Preto, SP - Brazil; 3 São Paulo SP Brazil Governo do Estado de São Paulo, São Paulo, SP - Brazil; 4 Campinas SP Brazil Universidade Estadual de Campinas (UNICAMP), Campinas, SP - Brazil; 5 Rio de Janeiro RJ Brazil Universidade Federal Fluminense (UFF), Rio de Janeiro, RJ - Brazil; 6 São Paulo SP Brazil Universidade São Francisco (USF), São Paulo, SP - Brazil; Pronto Socorro Cardiológico de Pernambuco (PROCAPE), Recife, PE - Brazil; Recife PE Brazil; 7 Recife PE Brazil Universidade de Pernambuco (UPE), Recife, PE - Brazil; 8 Rio de Janeiro RJ Brazil Hospital Pró-Cardíaco, Rio de Janeiro, RJ - Brazil; 9 Durham USA Duke University, Durham - USA; 10 Porto Alegre RS Brazil Hospital de Clínicas de Porto Alegre, Porto Alegre, RS - Brazil; 11 Londrina PR Brazil Universidade Estadual de Londrina, Londrina, PR - Brazil; 12 São Paulo SP Brazil Universidade de São Paulo (USP), São Paulo, SP - Brazil; 13 Curitiba PR Brazil Pontifícia Universidade Católica de Curitiba, Curitiba, PR - Brazil; 14 Rio de Janeiro RJ Brazil Hospital Samaritano, Rio de Janeiro, RJ - Brazil; 15 Boston USA Beth Israel Deaconess Medical Center / Harvard Medical School, Boston - USA; 16 São Paulo SP Brazil Instituto Dante Pazzanese de Cardiologia, São Paulo, SP - Brazil; 17 São Paulo SP Brazil Hospital do Coração (Hcor), São Paulo, SP - Brazil; 18 São Paulo SP Brazil Hospital Israelita Albert Einstein, São Paulo, SP - Brazil; 19 Campinas SP Brazil Pontifícia Universidade Católica de Campinas (PUCC), Campinas, SP - Brazil; 20 São Paulo SP Brazil Fundação Zerbini, São Paulo, SP - Brazil; 21 Porto Alegre RS Brazil Universidade Federal do Rio Grande do Sul (UFRGS), Porto Alegre, RS - Brazil; 22 Rio de Janeiro RJ Brazil Universidade do Estado do Rio de Janeiro (UERJ), Rio de Janeiro, RJ - Brazil; 23 São Luís MA Brazil Hospital Universitário Presidente Dutra, Universidade Federal do Maranhão (UFMA), São Luís, MA - Brazil; 24 São Paulo SP Brazil Faculdade Santa Marcelina, São Paulo, SP - Brazil; 25 São Paulo SP Brazil Fleury Medicina e Saúde, São Paulo, SP - Brazil; 26 Rio de Janeiro RJ Brazil Instituto D’Or de Pesquisa e Ensino (IDOR), Rio de Janeiro, RJ - Brazil; 27 Rio de Janeiro RJ Brazil Instituto Nacional de Cardiologia, Rio de Janeiro, RJ - Brazil; 28 Canoas RS Brazil Universidade Luterana do Brasil, Canoas, RS - Brazil; 29 Curitiba PR Brazil CardioEco Centro de Diagnóstico Cardiovascular e Ecocardiografia, Curitiba, PR - Brazil; 30 Curitiba PR Brazil Quanta Diagnósticos, Curitiba, PR - Brazil; 31 Ribeirão Preto SP Brazil Hospital Unimed Ribeirão Preto, Ribeirão Preto, SP - Brazil; 32 Ribeirão Preto SP Brazil Centro Avançado de Pesquisa, Ensino e Diagnóstico (CAPED), Ribeirão Preto, SP - Brazil; 33 São Paulo SP Brazil Santa Casa de Misericórdia de São Paulo, São Paulo, SP - Brazil; 34 João Pessoa PB Brazil Universidade Federal da Paraíba, João Pessoa, PB - Brazil; 35 Ribeirão Preto SP Brazil Hospital das Clínicas de Ribeirão Preto da Universidade de São Paulo, Ribeirão Preto, SP - Brazil; 36 Campinas SP Brazil Hospital Vera Cruz, Campinas, SP - Brazil; 37 São Paulo SP Brazil Hospital das Clínicas da Faculdade de Medicina de Botucatu, Universidade Estadual Paulista "Júlio de Mesquita Filho" (UNESP), São Paulo, SP - Brazil; 38 São Paulo SP Brazil Centro de Pesquisa Clínica - Indacor, São Paulo, SP - Brazil; 39 Porto Alegre RS Brazil Hospital Moinhos de Vento, Porto Alegre, RS - Brazil; 40 Porto Alegre RS Brazil Hospital de Clínicas de Porto Alegre (HCPA), Porto Alegre, RS - Brazil

**Table:** 

Guidelines on the Diagnosis and Treatment of Hypertrophic Cardiomyopathy - 2024	
The report below lists declarations of interest as reported to the SBC by the experts during the period of the development of these statement, 2022-2024.	
Expert	Type of relationship with industry
Alexandre Antonio Cunha Abizaid	Nothing to be declared
Alexandre da Costa Pereira	Nothing to be declared
Alexandre Siciliano Colafranceschi	Nothing to be declared
Ana Cristina Sayuri Tanaka	Nothing to be declared
Ana Flávia Malheiros Torbey	Nothing to be declared
Andre Schmidt	Nothing to be declared
Andrei C. Sposito	Nothing to be declared
Andreia Biolo	Financial declaration B - Research funding under your direct/personal responsibility (directed to the department or institution) from the Brazilian or international pharmaceutical, orthosis, prosthesis, equipment and implants industry: - Anylam: amyloidosis.
Ariane Vieira Scarlatelli Macedo	Financial declaration A - Economically relevant payments of any kind made to (i) you, (ii) your spouse/partner or any other person living with you, (iii) any legal person in which any of these is either a direct or indirect controlling owner, business partner, shareholder or participant; any payments received for lectures, lessons, training instruction, compensation, fees paid for participation in advisory boards, investigative boards or other committees, etc. From the brazilian or international pharmaceutical, orthosis, prosthesis, equipment and implants industry: - Bayer: Anticoagulação e insuficiência cardíaca; Pfizer: Anticoagulação e amiloidose; Jannsen: Leucemia. Other relationships Funding of continuing medical education activities, including travel, accommodation and registration in conferences and courses, from the brazilian or international pharmaceutical, orthosis, prosthesis, equipment and implants industry: - Bayer: heart failure.
Bruno Caramelli	Nothing to be declared
Bruno Pereira Valdigem	Other relationships Funding of continuing medical education activities, including travel, accommodation and registration in conferences and courses, from the brazilian or international pharmaceutical, orthosis, prosthesis, equipment and implants industry: - BMS: mavacamten; ABBOTT, Boston e Medtronic: materials for catheter ablation.
Carlos Alberto Pastore	Nothing to be declared
Carlos Eduardo Rochitte	Nothing to be declared
Charles Mady	Nothing to be declared
Claudio Tinoco Mesquita	Financial declaration B - Research funding under your direct/personal responsibility (directed to the department or institution) from the Brazilian or international pharmaceutical, orthosis, prosthesis, equipment and implants industry: - Patisiran/Alnylam: amyloidosis. Other relationships Funding of continuing medical education activities, including travel, accommodation and registration in conferences and courses, from the brazilian or international pharmaceutical, orthosis, prosthesis, equipment and implants industry: - Tafamidis/Pfizer: amyloidosis.
Daniela Calderaro	Financial declaration A - Economically relevant payments of any kind made to (i) you, (ii) your spouse/partner or any other person living with you, (iii) any legal person in which any of these is either a direct or indirect controlling owner, business partner, shareholder or participant; any payments received for lectures, lessons, training instruction, compensation, fees paid for participation in advisory boards, investigative boards or other committees, etc. From the brazilian or international pharmaceutical, orthosis, prosthesis, equipment and implants industry: - Bayer: atrial fibrillation, VTE, diabetic nephropathy. Other relationships Funding of continuing medical education activities, including travel, accommodation and registration in conferences and courses, from the brazilian or international pharmaceutical, orthosis, prosthesis, equipment and implants industry: - Bayer: diabetic nephropathy.
Denilson Campos Albuquerque	Other relationships Funding of continuing medical education activities, including travel, accommodation and registration in conferences and courses, from the brazilian or international pharmaceutical, orthosis, prosthesis, equipment and implants industry: - NovoNordisk: Semaglutide.
Edileide de Barros Correia	Financial declaration A - Economically relevant payments of any kind made to (i) you, (ii) your spouse/partner or any other person living with you, (iii) any legal person in which any of these is either a direct or indirect controlling owner, business partner, shareholder or participant; any payments received for lectures, lessons, training instruction, compensation, fees paid for participation in advisory boards, investigative boards or other committees, etc. From the brazilian or international pharmaceutical, orthosis, prosthesis, equipment and implants industry: - Pfizer: amyloidosis; Bristol: mavacantene; Sanofi: fabrazyme; Takeda: replagal. Other relationships Funding of continuing medical education activities, including travel, accommodation and registration in conferences and courses, from the brazilian or international pharmaceutical, orthosis, prosthesis, equipment and implants industry: - Pfizer, AstraZeneka: amyloidosis.
Edimar Alcides Bocchi	Financial declaration A - Economically relevant payments of any kind made to (i) you, (ii) your spouse/partner or any other person living with you, (iii) any legal person in which any of these is either a direct or indirect controlling owner, business partner, shareholder or participant; any payments received for lectures, lessons, training instruction, compensation, fees paid for participation in advisory boards, investigative boards or other committees, etc. From the brazilian or international pharmaceutical, orthosis, prosthesis, equipment and implants industry: - Servier, AstraZeneca, Merck: heart failure. B - Research funding under your direct/personal responsibility (directed to the department or institution) from the Brazilian or international pharmaceutical, orthosis, prosthesis, equipment and implants industry: - Merck: heart failure. Other relationships Funding of continuing medical education activities, including travel, accommodation and registration in conferences and courses, from the brazilian or international pharmaceutical, orthosis, prosthesis, equipment and implants industry: - Servier: heart failure.
Edmundo Arteaga-Fernández	Nothing to be declared
Eduardo Benchimol Saad	Financial declaration A - Economically relevant payments of any kind made to (i) you, (ii) your spouse/partner or any other person living with you, (iii) any legal person in which any of these is either a direct or indirect controlling owner, business partner, shareholder or participant; any payments received for lectures, lessons, training instruction, compensation, fees paid for participation in advisory boards, investigative boards or other committees, etc. From the brazilian or international pharmaceutical, orthosis, prosthesis, equipment and implants industry: - J&J, Abbott: electrophysiology.
Evandro Tinoco Mesquita	Financial declaration A - Economically relevant payments of any kind made to (i) you, (ii) your spouse/partner or any other person living with you, (iii) any legal person in which any of these is either a direct or indirect controlling owner, business partner, shareholder or participant; any payments received for lectures, lessons, training instruction, compensation, fees paid for participation in advisory boards, investigative boards or other committees, etc. From the brazilian or international pharmaceutical, orthosis, prosthesis, equipment and implants industry: - Ache: educational material and Astra classes. Other relationships Funding of continuing medical education activities, including travel, accommodation and registration in conferences and courses, from the brazilian or international pharmaceutical, orthosis, prosthesis, equipment and implants industry: - Pfizer: amyloidosis.
Fabiana Goulart Marcondes-Braga	Financial declaration A - Economically relevant payments of any kind made to (i) you, (ii) your spouse/partner or any other person living with you, (iii) any legal person in which any of these is either a direct or indirect controlling owner, business partner, shareholder or participant; any payments received for lectures, lessons, training instruction, compensation, fees paid for participation in advisory boards, investigative boards or other committees, etc. From the brazilian or international pharmaceutical, orthosis, prosthesis, equipment and implants industry: - Palestras e Comitê Consultivo: AstraZeneca; Boehringher-Lilly; Novartis; Bayer. B - Research funding under your direct/personal responsibility (directed to the department or institution) from the Brazilian or international pharmaceutical, orthosis, prosthesis, equipment and implants industry: - MSD: Victor Trial.
Fabio Biscegli Jatene	Financial declaration A - Economically relevant payments of any kind made to (i) you, (ii) your spouse/partner or any other person living with you, (iii) any legal person in which any of these is either a direct or indirect controlling owner, business partner, shareholder or participant; any payments received for lectures, lessons, training instruction, compensation, fees paid for participation in advisory boards, investigative boards or other committees, etc. From the brazilian or international pharmaceutical, orthosis, prosthesis, equipment and implants industry: - Edwards: prosthetics; remuneration for lectures at scientific events. B - Research funding under your direct/personal responsibility (directed to the department or institution) from the Brazilian or international pharmaceutical, orthosis, prosthesis, equipment and implants industry: - Edwards: valve prostheses; Samsung: wearables.
Fabio Fernandes	Financial declaration A - Economically relevant payments of any kind made to (i) you, (ii) your spouse/partner or any other person living with you, (iii) any legal person in which any of these is either a direct or indirect controlling owner, business partner, shareholder or participant; any payments received for lectures, lessons, training instruction, compensation, fees paid for participation in advisory boards, investigative boards or other committees, etc. From the brazilian or international pharmaceutical, orthosis, prosthesis, equipment and implants industry: - Pfizer: Tafamidis, Alnylam, Patisiran; Bristol: Mavacanteno. B - Research funding under your direct/personal responsibility (directed to the department or institution) from the Brazilian or international pharmaceutical, orthosis, prosthesis, equipment and implants industry: - São Paulo State Registry on amyloidosis, Pfizer, unrelated to medication. Other relationships Funding of continuing medical education activities, including travel, accommodation and registration in conferences and courses, from the brazilian or international pharmaceutical, orthosis, prosthesis, equipment and implants industry: - Pfizer: ISA 2024 congress.
Fabio Sandoli de Brito Junior	Financial declaration A - Economically relevant payments of any kind made to (i) you, (ii) your spouse/partner or any other person living with you, (iii) any legal person in which any of these is either a direct or indirect controlling owner, business partner, shareholder or participant; any payments received for lectures, lessons, training instruction, compensation, fees paid for participation in advisory boards, investigative boards or other committees, etc. From the brazilian or international pharmaceutical, orthosis, prosthesis, equipment and implants industry: - Proctor Edwards, Medtronic, Boston: TAVI.
Felix José Alvarez Ramires	Financial declaration A - Economically relevant payments of any kind made to (i) you, (ii) your spouse/partner or any other person living with you, (iii) any legal person in which any of these is either a direct or indirect controlling owner, business partner, shareholder or participant; any payments received for lectures, lessons, training instruction, compensation, fees paid for participation in advisory boards, investigative boards or other committees, etc. From the brazilian or international pharmaceutical, orthosis, prosthesis, equipment and implants industry: - Novartis: Sacubitril/Valsartana; Pfizer: Patisiran; Merck: Vericiquat; Amgen.
Fernando Bacal	Other relationships Funding of continuing medical education activities, including travel, accommodation and registration in conferences and courses, from the brazilian or international pharmaceutical, orthosis, prosthesis, equipment and implants industry: - Novartis: lecture at congress.
Fernando Luis Scolari	Financial declaration A - Economically relevant payments of any kind made to (i) you, (ii) your spouse/partner or any other person living with you, (iii) any legal person in which any of these is either a direct or indirect controlling owner, business partner, shareholder or participant; any payments received for lectures, lessons, training instruction, compensation, fees paid for participation in advisory boards, investigative boards or other committees, etc. From the brazilian or international pharmaceutical, orthosis, prosthesis, equipment and implants industry: - Bristol Myers Squibb. Other relationships Funding of continuing medical education activities, including travel, accommodation and registration in conferences and courses, from the brazilian or international pharmaceutical, orthosis, prosthesis, equipment and implants industry: - Bristol Myers Squibb.
Flávio de Souza Brito	Financial declaration A - Economically relevant payments of any kind made to (i) you, (ii) your spouse/partner or any other person living with you, (iii) any legal person in which any of these is either a direct or indirect controlling owner, business partner, shareholder or participant; any payments received for lectures, lessons, training instruction, compensation, fees paid for participation in advisory boards, investigative boards or other committees, etc. From the brazilian or international pharmaceutical, orthosis, prosthesis, equipment and implants industry: - AstraZeneca: Forxiga; Novartis: Entresto; Servier: Procoralan; Boehringer Ingelheim: Jardiance; Bayer: Xarelto; Merck: Concor; Daichii Sankyo: Lixiana; Viatris: Inspra; EMS: Bramicar; Biolab: Bivolet; Novonordisk: Ozempic. Other relationships Funding of continuing medical education activities, including travel, accommodation and registration in conferences and courses, from the brazilian or international pharmaceutical, orthosis, prosthesis, equipment and implants industry: - Novonordisk: Ozempic.
Gustavo Calado de Aguiar Ribeiro	Nothing to be declared
Henrique Barbosa Ribeiro	Financial declaration A - Economically relevant payments of any kind made to (i) you, (ii) your spouse/partner or any other person living with you, (iii) any legal person in which any of these is either a direct or indirect controlling owner, business partner, shareholder or participant; any payments received for lectures, lessons, training instruction, compensation, fees paid for participation in advisory boards, investigative boards or other committees, etc. From the brazilian or international pharmaceutical, orthosis, prosthesis, equipment and implants industry: - Medtronic, Boston Scientific, Edwards Lifesciences: TAVI classes and proctorship. B - Research funding under your direct/personal responsibility (directed to the department or institution) from the Brazilian or international pharmaceutical, orthosis, prosthesis, equipment and implants industry: - Medtronic: aortic stenosis.
Humberto Villacorta Junior	Financial declaration A - Economically relevant payments of any kind made to (i) you, (ii) your spouse/partner or any other person living with you, (iii) any legal person in which any of these is either a direct or indirect controlling owner, business partner, shareholder or participant; any payments received for lectures, lessons, training instruction, compensation, fees paid for participation in advisory boards, investigative boards or other committees, etc. From the brazilian or international pharmaceutical, orthosis, prosthesis, equipment and implants industry: - AstraZeneca: Forxiga e Lokelma; Novartis: Entresto; Pfizer: amyloidosis; Roche Diagnostics: NT-proBNP. B - Research funding under your direct/personal responsibility (directed to the department or institution) from the Brazilian or international pharmaceutical, orthosis, prosthesis, equipment and implants industry: - Roche: GDF-15 kits.
Ibraim Masciarelli Francisco Pinto	Nothing to be declared
Ieda Biscegli Jatene	Financial declaration A - Economically relevant payments of any kind made to (i) you, (ii) your spouse/partner or any other person living with you, (iii) any legal person in which any of these is either a direct or indirect controlling owner, business partner, shareholder or participant; any payments received for lectures, lessons, training instruction, compensation, fees paid for participation in advisory boards, investigative boards or other committees, etc. From the brazilian or international pharmaceutical, orthosis, prosthesis, equipment and implants industry: - AstraZeneca: Palivizumabe. Other relationships Participation in government-related regulatory authorities or advocacy authorities in cardiology: - Regional Council of Medicine of the State of São Paulo.
Josá albuquerque de Figueiredo Neto	Financial declaration A - Economically relevant payments of any kind made to (i) you, (ii) your spouse/partner or any other person living with you, (iii) any legal person in which any of these is either a direct or indirect controlling owner, business partner, shareholder or participant; any payments received for lectures, lessons, training instruction, compensation, fees paid for participation in advisory boards, investigative boards or other committees, etc. From the brazilian or international pharmaceutical, orthosis, prosthesis, equipment and implants industry: - Novartis; AstraZeneca; Boehringer; Servier. B - Research funding under your direct/personal responsibility (directed to the department or institution) from the Brazilian or international pharmaceutical, orthosis, prosthesis, equipment and implants industry: - Novartis. Other relationships Funding of continuing medical education activities, including travel, accommodation and registration in conferences and courses, from the brazilian or international pharmaceutical, orthosis, prosthesis, equipment and implants industry: - Novartis; AstraZeneca.
José Eduardo Krieger	Nothing to be declared
Juliano Novaes Cardoso	Nothing to be declared
Lidia Zytynski Moura	Financial declaration A - Economically relevant payments of any kind made to (i) you, (ii) your spouse/partner or any other person living with you, (iii) any legal person in which any of these is either a direct or indirect controlling owner, business partner, shareholder or participant; any payments received for lectures, lessons, training instruction, compensation, fees paid for participation in advisory boards, investigative boards or other committees, etc. From the brazilian or international pharmaceutical, orthosis, prosthesis, equipment and implants industry: - Novartis: Entresto; AstraZeneca: Forxiga; Boehringer: Jardiance; Bayer: Vericiguat; Vifor: Ferrinject. B - Research funding under your direct/personal responsibility (directed to the department or institution) from the Brazilian or international pharmaceutical, orthosis, prosthesis, equipment and implants industry: - Bayer: Vericiguat.
Ludhmila Abrahão Hajjar	Nothing to be declared
Luis Beck-da-Silva	Financial declaration A - Economically relevant payments of any kind made to (i) you, (ii) your spouse/partner or any other person living with you, (iii) any legal person in which any of these is either a direct or indirect controlling owner, business partner, shareholder or participant; any payments received for lectures, lessons, training instruction, compensation, fees paid for participation in advisory boards, investigative boards or other committees, etc. From the brazilian or international pharmaceutical, orthosis, prosthesis, equipment and implants industry: - Viatris; Pfizer; AstraZeneca; NovoNordisk. B - Research funding under your direct/personal responsibility (directed to the department or institution) from the Brazilian or international pharmaceutical, orthosis, prosthesis, equipment and implants industry: - CSL Vifor. Other relationships Funding of continuing medical education activities, including travel, accommodation and registration in conferences and courses, from the brazilian or international pharmaceutical, orthosis, prosthesis, equipment and implants industry: - NovoNordisk; Viatris.
Luis Eduardo Paim Rohde	Financial declaration A - Economically relevant payments of any kind made to (i) you, (ii) your spouse/partner or any other person living with you, (iii) any legal person in which any of these is either a direct or indirect controlling owner, business partner, shareholder or participant; any payments received for lectures, lessons, training instruction, compensation, fees paid for participation in advisory boards, investigative boards or other committees, etc. From the brazilian or international pharmaceutical, orthosis, prosthesis, equipment and implants industry: - AstraZeneca: Dapagliflozina e Ciclossilicato de Zircônio Sódico; Novartis: Sacubitril-Valsartana; Novonordisk: Semaglutida; Aché: Dapagliflozina; Merck Serono: Bisoprolol. Other relationships Funding of continuing medical education activities, including travel, accommodation and registration in conferences and courses, from the brazilian or international pharmaceutical, orthosis, prosthesis, equipment and implants industry: - Novonordisk: Semaglutida; Viatris: Eplerenone.
Luiz Claudio Danzmann	Financial declaration A - Economically relevant payments of any kind made to (i) you, (ii) your spouse/partner or any other person living with you, (iii) any legal person in which any of these is either a direct or indirect controlling owner, business partner, shareholder or participant; any payments received for lectures, lessons, training instruction, compensation, fees paid for participation in advisory boards, investigative boards or other committees, etc. From the brazilian or international pharmaceutical, orthosis, prosthesis, equipment and implants industry: - Novartis: Entresto; AstraZeneca: Forxiga; Servier: Procoralan.
Manoel Fernandes Canesin	Nothing to be declared
Manuel Nicolas Cano	Nothing to be declared
Marcelo Dantas Tavares de Melo	Nothing to be declared
Marcelo Imbroinise Bittencourt	Financial declaration A - Economically relevant payments of any kind made to (i) you, (ii) your spouse/partner or any other person living with you, (iii) any legal person in which any of these is either a direct or indirect controlling owner, business partner, shareholder or participant; any payments received for lectures, lessons, training instruction, compensation, fees paid for participation in advisory boards, investigative boards or other committees, etc. From the brazilian or international pharmaceutical, orthosis, prosthesis, equipment and implants industry: - Pfizer: amyloidosis; Bristol: hypertrophic cardiomyopathy; Sanofi: Fabry disease.
Marcelo Luiz Campos Vieira	Nothing to be declared
Marcelo Westerlund Montera	Nothing to be declared
Marcus V. Simões	Nothing to be declared
Martino Martinelli Filho	Nothing to be declared
Mauricio Ibrahim Scanavacca	Financial declaration A - Economically relevant payments of any kind made to (i) you, (ii) your spouse/partner or any other person living with you, (iii) any legal person in which any of these is either a direct or indirect controlling owner, business partner, shareholder or participant; any payments received for lectures, lessons, training instruction, compensation, fees paid for participation in advisory boards, investigative boards or other committees, etc. From the brazilian or international pharmaceutical, orthosis, prosthesis, equipment and implants industry: - Daichii Sankyo: anticoagulation. B - Research funding under your direct/personal responsibility (directed to the department or institution) from the Brazilian or international pharmaceutical, orthosis, prosthesis, equipment and implants industry: - J&J: ablation of ventricular tachycardia. C - Personal research funding paid by the brazilian or international pharmaceutical, orthosis, prosthesis, equipment and implants industry: - ABBOT: Catheter ablation of the cindam vagal vessel. Other relationships Funding of continuing medical education activities, including travel, accommodation and registration in conferences and courses, from the brazilian or international pharmaceutical, orthosis, prosthesis, equipment and implants industry: - J&J: industry-sponsored symposium.
Minna Moreira Dias Romano	Nothing to be declared
Moysés de Oliveira Lima Filho	Other relationships Employment relationship with the brazilian or international pharmaceutical, orthosis, prosthesis, equipment and implants industry, as well as any employment relationship with health insurance companies or medical audit companies (including part-time jobs) in the year to which your declaration refers: - Member of the Executive Board of Unimed de Ribeirão Preto.
Mucio Tavares de Oliveira Junior	Financial declaration A - Economically relevant payments of any kind made to (i) you, (ii) your spouse/partner or any other person living with you, (iii) any legal person in which any of these is either a direct or indirect controlling owner, business partner, shareholder or participant; any payments received for lectures, lessons, training instruction, compensation, fees paid for participation in advisory boards, investigative boards or other committees, etc. From the brazilian or international pharmaceutical, orthosis, prosthesis, equipment and implants industry: - Sanofi Pasteur: vaccines; Viatris: Inspra; AstraZeneca: Forxiga. B - Research funding under your direct/personal responsibility (directed to the department or institution) from the Brazilian or international pharmaceutical, orthosis, prosthesis, equipment and implants industry: - Sanofi Pasteur: influenza.
Murillo de Oliveira Antunes	Financial declaration A - Economically relevant payments of any kind made to (i) you, (ii) your spouse/partner or any other person living with you, (iii) any legal person in which any of these is either a direct or indirect controlling owner, business partner, shareholder or participant; any payments received for lectures, lessons, training instruction, compensation, fees paid for participation in advisory boards, investigative boards or other committees, etc. From the brazilian or international pharmaceutical, orthosis, prosthesis, equipment and implants industry: - Takeda; Bayer; Pfizer; Sanofi. B - Research funding under your direct/personal responsibility (directed to the department or institution) from the Brazilian or international pharmaceutical, orthosis, prosthesis, equipment and implants industry: - Bristol: Mavacamten. Other relationships Funding of continuing medical education activities, including travel, accommodation and registration in conferences and courses, from the brazilian or international pharmaceutical, orthosis, prosthesis, equipment and implants industry: - Biolab; Takeda; Sanofi; Alnaylam.
Otavio Rizzi Coelho-Filho	Financial declaration A - Economically relevant payments of any kind made to (i) you, (ii) your spouse/partner or any other person living with you, (iii) any legal person in which any of these is either a direct or indirect controlling owner, business partner, shareholder or participant; any payments received for lectures, lessons, training instruction, compensation, fees paid for participation in advisory boards, investigative boards or other committees, etc. From the brazilian or international pharmaceutical, orthosis, prosthesis, equipment and implants industry: - Pfizer: amyloidosis/Tafamides; AstraZeneca: heart failure/Forxiga; Bayer: Firialta; Norvartis: heart failure/dyslipidemia; EMS: heart failure. Other relationships Funding of continuing medical education activities, including travel, accommodation and registration in conferences and courses, from the brazilian or international pharmaceutical, orthosis, prosthesis, equipment and implants industry: - Bayer; Pfizer; AstraZeneca.
Pedro Alves Lemos Neto	Nothing to be declared
Pedro Silvio Farsky	Financial declaration A - Economically relevant payments of any kind made to (i) you, (ii) your spouse/partner or any other person living with you, (iii) any legal person in which any of these is either a direct or indirect controlling owner, business partner, shareholder or participant; any payments received for lectures, lessons, training instruction, compensation, fees paid for participation in advisory boards, investigative boards or other committees, etc. From the brazilian or international pharmaceutical, orthosis, prosthesis, equipment and implants industry: - Lilly: empagliflozin. Other relationships Funding of continuing medical education activities, including travel, accommodation and registration in conferences and courses, from the brazilian or international pharmaceutical, orthosis, prosthesis, equipment and implants industry: - Novo Nordisk: semaglutide.
Pedro Vellosa Schwartzmann	Financial declaration B - Research funding under your direct/personal responsibility (directed to the department or institution) from the Brazilian or international pharmaceutical, orthosis, prosthesis, equipment and implants industry: - MSD; Novartis; AstraZeneca; Alnylam; BridgeBio; Ionis; Lilly. Other relationships Funding of continuing medical education activities, including travel, accommodation and registration in conferences and courses, from the brazilian or international pharmaceutical, orthosis, prosthesis, equipment and implants industry: - Pfizer; AstraZeneca; Novartis; Alnylam; NovoNordisk.
Renato Delascio Lopes	Nothing to be declared
Ricardo Mourilhe-Rocha	Financial declaration A - Economically relevant payments of any kind made to (i) you, (ii) your spouse/partner or any other person living with you, (iii) any legal person in which any of these is either a direct or indirect controlling owner, business partner, shareholder or participant; any payments received for lectures, lessons, training instruction, compensation, fees paid for participation in advisory boards, investigative boards or other committees, etc. From the brazilian or international pharmaceutical, orthosis, prosthesis, equipment and implants industry: - AstraZeneca: insuficiência cardíaca e discalemias;/ Bayer: insuficiência renal; Boehringer-Lilly: insuficiência cardíaca; Novartis: insuficiência cardíaca; NovoNordisk: insuficiência cardíaca; Servier: insuficiência cardíaca; Daiichi-Sankyo: fibrilação atrial. B - Research funding under your direct/personal responsibility (directed to the department or institution) from the Brazilian or international pharmaceutical, orthosis, prosthesis, equipment and implants industry: - BMS: mavacanteno); Boehringer: empagliflozina; Novartis: sacubitril/valsartan.
Ricardo Ribeiro Dias	Nothing to be declared
Silvia Marinho Martins Alves	Other relationships Funding of continuing medical education activities, including travel, accommodation and registration in conferences and courses, from the brazilian or international pharmaceutical, orthosis, prosthesis, equipment and implants industry: - Novartis: Entresto.
Silvia Moreira Ayub Ferreira	Financial declaration A - Economically relevant payments of any kind made to (i) you, (ii) your spouse/partner or any other person living with you, (iii) any legal person in which any of these is either a direct or indirect controlling owner, business partner, shareholder or participant; any payments received for lectures, lessons, training instruction, compensation, fees paid for participation in advisory boards, investigative boards or other committees, etc. From the brazilian or international pharmaceutical, orthosis, prosthesis, equipment and implants industry: - Abbott, CSL Vifor.
Sivio Henrique Barberato	Other relationships Funding of continuing medical education activities, including travel, accommodation and registration in conferences and courses, from the brazilian or international pharmaceutical, orthosis, prosthesis, equipment and implants industry: - Bristol: Camzyos.
Stephanie Itala Rizk	Nothing to be declared
Vagner Madrini Junior	Nothing to be declared
Viviane Tiemi Hotta	Nothing to be declared
Walkiria Samuel Avila	Nothing to be declared
Wilson Mathias Junior	Nothing to be declared
Wolney de Andrade Martins	Nothing to be declared

## 1. Introduction

Scientific knowledge of hypertrophic cardiomyopathy (HCM) has significantly improved in the past decades. A better understanding of its pathogenesis, significant advances in the use of imaging methods, and the more common application of genetic analysis, in addition to a better characterization of the natural history of this myocardial disease, have profoundly reformulated its clinical and prognostic significance. Conversely, these processes were accompanied by the development of new medications addressing molecular mechanisms intrinsically linked to the pathophysiology and pathogenesis of the disease and its manifestations, representing an enormous achievement of science and a milestone in the history of cardiology and myocardial diseases.

Therefore, in light of so much new knowledge, whose incorporation into clinical practice is necessary and urgent, these Guidelines aim to present the most current recommendations for the diagnosis, prognostic staging, and treatment of HCM based on a critical review of the current scientific literature.

In this position statement, classes of recommendation and levels of evidence tables were constructed according to the following definitions ( Table 1 ).

**Table 1:** Definitions of classes of recommendation and levels of evidence used in these Guidelines

Classes (grades) of recommendation:
**Class I** - Conditions for which there is conclusive evidence and, failing that, general agreement that a given procedure is safe and useful/effective.
**Class IIa** - Conditions for which there is conflicting evidence and/or a divergence of opinion about the safety and usefulness/efficacy of a procedure. Most studies/experts approve.
**Class IIb** - Conditions for which there is conflicting evidence and/or a divergence of opinion about the safety and usefulness/efficacy of a procedure. Safety and usefulness are less well established, and there is no predominance of opinions in favor of the method.
**Class III** - Conditions for which there is evidence and/or general agreement that a procedure is not useful/effective and, in some cases, may be harmful.
Levels of evidence:
**Level A** - Data obtained from several large, randomized studies showing concurring results and/or a robust meta-analysis of randomized controlled trials.
**Level B** - Data obtained from a less robust meta-analysis, a single randomized study, or from nonrandomized (observational) studies.
**Level C** - Data obtained from consensual expert opinions.

### 1.1. Definition and Natural History of HCM

HCM is characterized by hypertrophy of the myocardium, which determines an increase in wall thickness, with no ventricular dilatation in the initial stages, occurring in the absence of other cardiac, systematic, metabolic, or syndromic diseases that could explain this phenotypic change.^1^

The diagnosis of HCM can be established by imaging, including two-dimensional (2D) echocardiography and cardiovascular magnetic resonance (CMR). HCM is defined by an end-diastolic wall thickness in any segment of the ventricles of ≥ 15 mm in adults or ≥ 13 mm in family members of a patient with HCM or in combination with a positive genetic test.^2 , 3^

In pediatric patients, values obtained in imaging tests must be indexed to body surface area (BSA). According to the American College of Cardiology (ACC) and the American College of Cardiology (ACC), values greater than a Z-score > 2.5 are defined as pathological hypertrophy in asymptomatic children with no positive family history. Conversely, values greater than 2 standard deviations are sufficient for an early diagnosis to be made.^2 , 4^In adults, a Z-score corresponding to a septal measurement of 15 mm is 6 to 7, while a septum measuring 13 mm equates to a Z-score of 4 to 6.

HCM is the most prevalent cardiomyopathy of genetic origin.^2^It has an autosomal dominant mode of inheritance and is mainly caused by mutations in gene encoding sarcomeric proteins.^5^Phenotypic presentations vary greatly regarding symptoms, clinical progression, prognosis, and penetrance.^6^Most individuals with HCM are symptomatic and have a normal life expectancy, while a minority suffers from debilitating symptoms and are at increased risk of early sudden cardiac death (SCD). HCM accounts for one of the main causes of SCD in young people and athletes.^7 , 8^Because left ventricular outflow tract (LVOT) obstruction is present or develops over time in the majority of patients with HCM, but 1/3 remain nonobstructive, the writing committee recommends the term HCM (with or without LVOT obstruction). Symptoms may be related to a series of pathophysiologic mechanisms, including diastolic dysfunction,^9^heart failure (HF) with preserved or reduced ejection fraction (EF),^10 , 11^LVOT obstruction^12 , 13^with or without significant mitral regurgitation (MR),^14^autonomic dysfunction,^15^ischemia,^16 , 17^and cardiac arrhythmias.^18 - 21^

### 1.2. Epidemiology

To date, HCM has been reported in 122 countries (90% of the world population), with a prevalence of 1 case per 500 persons in the general population, according to the first echocardiography-based epidemiological studies.^22 , 23^However, more recent studies including individuals carrying pathogenic genes (genotype-positive, phenotype-negative) found a prevalence of 1 case per 200 people,^24^meaning that HCM is far more common than initially thought, and an estimated 400,000 people could be affected in Brazil.^25^

### 1.3. Natural History

HCM is a heterogeneous disease with an unpredictable course that presents many particularities and challenges. It may manifest from childhood to the eighth decade of life. More recent cohort studies show that approximately 46% of patients may present a benign course, with a normal life expectancy and no limitations, and the remainder of patients may develop progressive symptoms or present clinical complications and adverse events throughout their lives ( Figure 1 ). The main adverse events reported in patients with HCM include sudden death, myocardial ischemia, progressive functional limitation due to LVOT obstruction or diastolic dysfunction, progression to left ventricular (LV) systolic dysfunction, atrial fibrillation (AF) with increased risk of thrombotic events, and ventricular arrhythmias^26 - 29^( Figure 1 ). Studies suggest that patients diagnosed early in life or those with pathogenic variants in sarcomeric genes are more likely to experience these adverse events throughout their life.^29^

**Figure 1:** Natural history of HCM, with the estimated frequency of benign clinical progression and different complications. * An adequate risk stratification of SCD and ICD implantation can reduce the incidence of SCD to approximately 1%. HCM: hypertrophfic cardiomyopathy; HF: heart failure. Adapted from Maron et al.^33^

Periodic assessment of individual risk for SCD is extremely important and remains a challenge in the management of HCM. The main factors associated with sudden death are younger age (< 30 years); personal history of aborted SCD; sustained ventricular arrhythmias; maximum LV wall thickness; arrhythmic syncope; family history of SCD; nonsustained ventricular tachycardia (NSVT); decreased LVEF (< 50%); and extensive myocardial fibrosis on CMR (> 15-20%).^30^In young high-risk patients, the use of an implantable cardioverter-defibrillator (ICD) should be considered.^31^

HF in HCM is caused by different pathophysiologic mechanisms, including LVOT obstruction and global diastolic or systolic ventricular dysfunction. A study with a mean follow-up of 6 years revealed that 17% of patients progressed to New York Heart Association (NYHA) functional class (FC) III/IV, while 55% remained in NYHA FC I. AF was the most common disease variable associated with progressive HF. Among patients with HCM, 30% to 40% will develop adverse events, including 1) sudden death events; 2) progressive limiting symptoms due to LVOT obstruction or diastolic dysfunction; 3) HF symptoms related to systolic dysfunction; and 4) AF with risk for thromboembolic stroke. However, studies with long-term follow-up have demonstrated that, for patients at risk or who develop one of these HCM-related complications, the application of contemporary cardiovascular therapies and interventions have reduced mortality rates to < 1.0%/year.^24 , 32^

## 2. Clinical Status

### 2.1. Arrhythmias and Syncope

Patients with HCM may develop cardiac arrhythmias, which may be asymptomatic — detected during a routine examination or by 24-hour Holter monitoring — or symptomatic. The most common arrhythmias are extrasystoles (supraventricular or ventricular), AF or atrial flutter, and NSVT or sustained ventricular tachycardia (SVT). When HCM is associated with pre-excitation syndromes, paroxysmal supraventricular tachycardia may also occur, and the risk of AF and syncope also appears to be increased in these cases.^1^In some cases, sudden death may be the first manifestation of HCM, especially during physical exercise, which is the main cause of sudden death in athletes.^34^

The main symptoms indicative of possible cardiac arrhythmias are palpitations, presyncope, and syncope.

### 2.2. Chest Pain

Patients with HCM may present with chest pain triggered by physical exercise or which may also occur at rest. Myocardial ischemia is most commonly caused by HCM-related alterations, leading to a mismatch between myocardial oxygen supply and demand.^35^Microvascular changes, ventricular hypertrophy, increased wall stress, and LVOT obstruction are changes that occur in HCM and may also be responsible for chest pain. Abnormal coronary flow is also caused by the effects of compression and deformation of intramyocardial blood vessels and ventricular relaxation.^17 , 36^However, in cases of typical angina, it is important to exclude epicardial coronary disease, especially in older patients with cardiovascular risk factors. For this purpose, performing coronary computed tomography (CT) angiography or coronary cineangiography is recommended, which can reveal associated obstructive atherosclerosis or changes in the coronary arteries such as myocardial bridge, which can also lead to angina.^17 , 36 , 37^

### 2.3. Heart Failure

Patients with HCM can develop a varied degree of structural and functional cardiac impairment. The vast majority of patients with HF (estimated at 90%) exhibit dynamic LVOT obstruction at rest or during exercise, indicating a central role of this disorder in the development of HF in HCM. Exertional dyspnea and fatigue are the main manifestations, while orthopnea and paroxysmal dyspnea are less common. Lower prevalence, a younger patient population with fewer comorbidities and typically presenting preserved EF, and lower mortality are characteristics of HCM. Because advanced HF manifesting with severe systemic and/or pulmonary congestion is rare, management is mostly performed on an outpatient basis.^38^

Patients with HF with LVOT obstruction, whether at rest or during exercise, present high LV pressure and secondary MR, usually progressing with refractoriness to drug treatment.^7^The condition is generally accompanied by pulmonary hypertension, diastolic dysfunction, and inadequate response to the increase in stroke volume due to exercise and other extrinsic factors.^39^

Dynamic changes in the gradient likely explain the daily or hourly variations and fluctuations in reported symptoms. Obstruction can be triggered by factors such as position, hydration, diet, alcohol consumption, or any variable that increases LV contractility and cardiac output, such as tachycardia or reduced ventricular volume.^15^Although the resting gradient is related to the development of HF, paradoxically, some patients, even with an increased gradient, progress satisfactorily at an older age of more than 65 years.^40^

Patients with nonobstructive HCM tend to progress more favorably, being oligo or asymptomatic, while a minority develops symptoms of advanced disease. Hospitalizations are infrequent, the rate of heart transplantation is approximately 2% to 3%, and death specifically due to HF is rare.^41^

### 2.4. Pathophysiology of HCM

Several preclinical studies indicate that the myocardial hypertrophy observed in HCM is closely linked to increased myocardial contractility, with increased myosin activation. Several mutations in different components of the sarcomere have been associated with this process ( Figure 2 ).

**Figure 2:** Schematic representation of myofilaments, myosin activation, and the main sarcomeric mutations. Adapted from Baron and Maron.^42^

The clinical manifestations of HCM result from the interaction between several pathophysiological elements, namely (1) dynamic LVOT obstruction, (2) myocardial ischemia, (3) cardiac arrhythmias, (4) MR, (5) ventricular diastolic dysfunction, and (6) autonomic dysfunction.^2^

### 2.5. Dynamic LVOT Obstruction

LVOT obstruction, either at rest or with provocation, is present in approximately 75% of patients with HCM.^14^The two main mechanisms involved are septal hypertrophy with LVOT narrowing, leading to abnormal blood flow vectors that displace the mitral valve (MV) leaflets anteriorly; and anatomic alterations in the MV as well as displacement of the papillary muscles and MV apparatus ( Figure 3 ). By increasing LV systolic pressure, LVOT obstruction may also exacerbate LV hypertrophy (LVH), HF, ischemia, and prolong ventricular relaxation.^43 - 45^The presence of peak LVOT gradients ≥ 30 mm Hg is considered to be indicative of obstructive HCM, while the detection of resting or provoked gradients ≥ 50 mm Hg is indicative of significant LVOT obstruction, and septal reduction therapy (SRT) may be considered in these cases depending on the symptoms and refractoriness to clinical treatment.

**Figure 3:** Schematic representation of the main factors that lead to left ventricular outflow tract obstruction and mitral regurgitation in patients with hypertrophic cardiomyopathy, highlighting the anterior systolic movement of the anterior leaflet of the MV (thick black arrows). Adapted from Young et al.^50^

Changes in the LVOT gradient may result from preload, afterload, or myocardial contractility abnormalities secondary to stimuli such as daily activities, breathing, and food and alcohol intake.^46^Thus, provocative maneuvers with simultaneous echocardiography may be necessary in patients with low (< 30 mm Hg) or absent peak resting gradients to induce LVOT obstruction. Such maneuvers include standing, Valsalva maneuver, amyl nitrite inhalation, or exercise.^47 - 49^Depending on the location and its anatomical characteristics, the obstruction can be characterized as valvular, dynamic LVOT obstruction, hypertrophied papillary muscles, anomalous papillary muscle insertion, or muscular obstruction caused by compensatory hyperkinesis after infarction.^46 - 49^

### 2.6. Myocardial Ischemia

Classically, myocardial ischemia in patients with HCM results from a mismatch between oxygen supply and demand in a manner analogous to what occurs in coronary artery disease, but without the presence of atheromatous plaques in the epicardial coronary arteries. The presence of concomitant coronary atherosclerosis portends a worse prognosis, with lower survival rates and a higher risk of sudden death.^36^However, the mechanisms by which ischemia occurs in HCM are much more complex. Despite an increased flow and low basal coronary resistance,^17^patients with HCM have decreased coronary flow reserve when undergoing tachycardia, with a lower ischemic threshold and a significant rate of *angina pectoris* .^17 , 35^The previously mentioned mismatch is the result of several factors. Conditions that reduce oxygen supply include (a) microvascular dysfunction; (b) inadequate capillary density in relation to the increase in myocardial mass; (c) medial hypertrophy; (d) increased intracavitary pressures; and (e) myocardial bridging with significant flow obstruction.^18 , 51^Increases in myocardial oxygen consumption also contribute to this mismatch, and the determining elements are (a) myocardial hypertrophy; and (b) the patient's hyperdynamic state.^2 , 18 , 51^

The clinical importance of ischemia in patients with HCM lies in its deleterious effects, such as electrical instability and consequent malignant arrhythmias, the induction of diffuse myocardial fibrosis or areas of myocardial infarction, and the formation of aneurysms and ventricular systolic dysfunction. Myocardial ischemia contributes to a poor prognosis and severe acute events.^2^

### 2.7. Arrhythmia

Symptogms attributed to arrhythmia are frequently reported in patients with HCM. Syncope, although unexplained in 91% of cases, is reported in 16% of patients with HCM. Arrhythmic events are more frequent in patients with HCM and syncope than in those without reports of syncope (7.7% vs. 3.6%). The mechanisms by which syncope occurs include outflow tract obstruction, usually during or after a bout of exercise; and vasovagal syncope due to dysautonomia, typically preceded by prodromal symptoms.

Understanding the underlying mechanism of syncope is critical for determining the therapeutic approach, and the possibility of malignant ventricular arrhythmia as the cause should always be considered. However, in patients with HCM, syncope may be triggered by low cardiac output secondary to hypovolemia, the use of diuretics or vasodilators, or severe uncompensated anemia.^52 , 53^

Myocardial fibrosis is found as HCM progresses, and many studies have shown that fibrosis is a marker of poor prognosis in this condition. There have been attempts to correlate fibrosis with arrhythmias in HCM. However, the results of different studies are divergent. Recently, it was found that it is not the mere presence, but the actual extent of myocardial fibrosis assessed by late gadolinium enhancement on CMR that determines the occurrence of NSVT in patients with HCM.^54^

### 2.8. MR

MR may occur in direct association with LVOT obstruction and systolic anterior motion (SAM) of the MV leaflet or result from primary structural abnormalities of the MV leaflet. SAM of the MV causes loss of leaflet coaptation, resulting in a predominantly posteriorly or laterally directed jet in mid-to-late systole. However, centrally and anteriorly directed jets may also occur.^15^Factors that affect the severity of LVOT obstruction can also affect the degree of MR. Primary anomalies of the MV and its subvalvular apparatus are also common, including leaflet elongation, anomalous papillary muscle insertion, and anterior displacement of papillary muscles.^44 , 45^The presence of MR can determine the worsening of pulmonary congestion, being an important element in the genesis of dyspnea and reduced functional capacity in patients with HCM.

### 2.9. Diastolic Ventricular Dysfunction

Diastolic dysfunction in HCM occurs through several associated mechanisms, such as nonuniformity in ventricular contraction and relaxation, high intracavitary pressures, and delayed inactivation from abnormal intracellular calcium reuptake. In the absence of LVOT obstruction, exercise intolerance or symptoms of HF may also occur.^9 , 10 , 55^Chamber stiffness can arise from myocardial hypertrophy, ischemia, and replacement or interstitial fibrosis. In some patients, the severity of hypertrophy also significantly compromises ventricular cavity size and stroke volume, causing decreased exercise capacity in HCM, which carries prognostic impact independent of LVOT obstruction.^56^With impairment of ventricular myocardial relaxation, greater dependency on the atrial systole for ventricular filling may occur, leading to poor tolerance of AF. An association between left atrial (LA) fibrosis, HCM, and AF has been reported.^57^

### 2.10. Autonomic Dysfunction

The prevalence of autonomic dysfunction in patients with HCM is uncertain. Abnormal blood pressure response to exercise has been described, as well as impaired heart rate recovery and inappropriate vasodilation.^2 , 16^

## 3. Complementary Tests

The diagnostic approach to HCM involves different imaging modalities for phenotypic identification, with the main goal of identifying obstructive HCM. As shown in the Central Figure , after suspicion based on clinical history, physical examination, and family history, different imaging tests can be used, in addition to genetic testing.

### 3.1. Electrocardiography

Although several changes in the electrocardiogram (ECG) are associated with HCM ( Table 2 ),^58^they have no set pattern. This is possibly due to the variable expressivity of HCM, which makes clinical association and severity characterization based on ECG unfeasible. Anatomically, ECG findings may indicate areas of myocardial fibrosis in the anterior and lateral LV walls.^58^The ECG tracing may be abnormal in 90% of individuals with HCM and in 75% of asymptomatic family members, but ECG changes are not disease-specific.^59^In the pediatric population, the ECG is mostly altered in cases of sarcomeric mutations even before the appearance of LVH on echocardiography.^60^In patients with HCM, ECG changes may include a pseudoinfarction pattern with prominent inferolateral Q waves and prominent precordial R waves, which are related to increased depolarization forces generated by the hypertrophic septum.^59^The association of voltage criteria for LVH may be among the ECG changes, but only 2% of them are isolated.^61^Complete bundle branch blocks are also uncommon in HCM, the presence of which suggests previous invasive interventions to relieve LVOT obstruction.^62^

**Table 2:** Main ECG changes found in patients with HCM

Left axis deviation.
P-wave abnormalities associated with left and right atrial enlargement and P-wave prolongation (a known predictor of AF).^62^
Deep narrow Q waves in inferior (DII, DIII, avF) or precordial leads, but more commonly occurring in lateral leads (D1, aVL, V4-V6) when associated with classic signs of left ventricular hypertrophy.
Repolarization changes with ST-segment depression (> -0,2 mV) and deep T-wave inversion (giant negative T waves); when occurring in the lateral leads, it is a marker of apical HCM.^62 - 65^
A QTc interval > 480 ms is more common in patients with HCM and reflects cardiac hypertrophy, fibrosis, and outflow tract obstruction.

It should be noted that the ECG may change significantly throughout the course of the disease. In the initial stages, increased QRS voltages and ST-T segment changes may be observed. Over the following decades, the most common changes are progressive LA enlargement and various degrees of QRS prolongation, reflecting septal fibrosis and conduction tissue involvement. In the later stages, when the myocardial fibrosis burden becomes significant, left bundle branch block (LBBB) may develop.^62 , 66^ Figure 4 shows common ECG findings in apical HCM.

**Figure 4:** Resting 12-lead ECG of a patient with apical HCM showing left ventricular hypertrophy and deep T-wave inversion in the anteroseptal and lateral walls. Authors’ personal archive.

### 3.2. Biomarkers

Natriuretic peptides (NP) are established biomarkers in the diagnosis of HF. Although they may be altered in patients with phenotypic manifestations of HCM, they are not specific for its diagnosis. Type B NP (BNP) and the N-terminal prohormone of BNP (NT-proBNP) are correlated with FC and severity of ECG changes, such as LVOT gradient, septal thickness, and degree of MR.^67 , 68^Likewise, high-sensitivity cardiac troponin (TcAS) may be altered. A study found abnormal TcAS values (> 0.014 ng/mL) in 54% of patients with HCM, which were correlated with the severity of echocardiographic parameters such as septal thickness and left atrium diameter.^69^Although these biomarkers are not specific for the diagnosis of HCM, they are important prognostic markers.^3 , 67 - 69^NP and TcAS are predictors of cardiovascular events, such as death from all causes, cardiovascular death, heart transplantation, and hospitalizations due to HF. However, they are not predictors of SCD and ICD implantation.^3 , 67 - 69^A recent meta-analysis identified NT-proBNP and ultrasensitive C-reactive protein as predictors of cardiovascular death and TcAS as a predictor of the combined event of HF, malignant ventricular arrhythmias, and stroke.^70^

### 3.3. Echocardiography

Transthoracic echocardiography (TTE) is crucial in the initial assessment and diagnosis of HCM, which is established by the presence of an end-diastolic wall thickness of ≥ 15 mm in a nondilated ventricle, in the absence of another cause of LVH ( Figure 5 ).^2^In patients with a family history of HCM or a known disease-causing genetic mutation, a diastolic myocardial thickness ≥ 13 mm may be diagnostic. In the pediatric population, a single cutoff value cannot be used for patients of different ages and body surface areas, as the diagnostic criteria needs to be adjusted for growth. Thus, cardiac measurements are expressed as Z-scores, corresponding to standard deviations from size-specific means. For children, Z-scores > 2 are diagnostic of HCM.^71 , 72^Measurements should be taken during diastole, guided by the ECG tracing, and at the moment of the largest LV diameter, avoiding shortenings and the inclusion of chordae and structures such as trabeculations in this measurement. If LVH is identified on echocardiography, probable causes should be investigated during the examination, such as aortic stenosis, subaortic membrane, and aortic coarctation.^73^The use of contrast is of great value, especially in screened patients with a positive genetic test result, with ECG changes suggestive of HCM, and with apical forms. Measurements of myocardial mass taken with three-dimensional (3D) echocardiography in patients with HCM have a better correlation with measurements obtained by CMR when compared with measurements obtained by 2D echocardiography and should be the method of choice, if available.^74 , 75^RV free wall thickness should be measured at end-diastole in the subcostal view and should not include epicardial fat or myocardial trabeculations.^76^ Table 3 describes important findings in the echocardiogram of patients with HCM. This imaging method also allows the identification of apical aneurysm, which is associated with a higher risk of sudden death ( Figure 7 ).

**Figure 5:** Transthoracic echocardiogram of a patient with HCM showing significant increase in septal wall thickness (25 mm) in the parasternal longitudinal (A) and transverse view at the level of the papillary muscles (B). LA: left atrium; LV: left ventricle; RV: right ventricule. Source: Authors’ personal archive.

**Table 3:** Important information in the echocardiogram report of patients with HCM

Indexed LA volume
IVS thickness, PW
Location of segments with increased myocardial thickness
Left ventricular ejection fraction
Describe apical aneurysm when present
Global longitudinal strain assessment, if available
Analysis of diastolic function
Describe the presence of intraventricular gradients (midventricular, apical, or in the LVOT)
Describe the presence of SAM of the MV
Describe the anatomy and presence of abnormalities of the MV, papillary muscles, and subvalvular apparatus
Describe mitral regurgitation and mechanisms

LA: left atrium; IVS: interventricular septum; PW: posterior wall; LVOT: left ventricular outflow tract; SAM: systolic anterior motion; MV: mitral valve.

**Figure 7:** Transthoracic echocardiogram of a patient with HCM showing an apical aneurysm in the apical four-chamber view (A and B) and apical two-chamber view (C). RA: right atrium; LA: left atrium; RV: right ventricle; LV: left ventricle. Source: Authors’ personal archive.

#### 3.3.1. Assessment of Systolic Function

HCM commonly presents with preserved or even hyperdynamic LVEF. Less than 10% of patients with HCM have reduced LVEF, and LVEF values < 0.50 portend a worse prognosis. To overcome the limitations of measuring LVEF using the biplane Simpson's method, analysis of cardiac mechanics using global longitudinal strain (GLS) appears to be a promising tool in the differential diagnosis of HCM phenocopies ( Figure 6 )^2 , 75^and to improve prognostic stratification in cases with preserved LVEF. Despite being well-known and researched tool for over a decade, its visual parametric analysis is more relevant, especially for differentiating HCM from Fabry disease and cardiac amyloidosis.^74 , 76^An inverse relationship between myocardial thickness, and also the amount of myocardial fibrosis, and the GLS is well established

**Figure 6:** Representation of longitudinal strain assessment in a patient with HCM. A reduction in longitudinal strain values is observed in the hypertrophic (basal, middle, and apical) segments of the septal wall (arrows) in the apical four-chamber view. Source: Authors’ personal archive.

#### 3.3.2. Assessment of Diastolic Function

LV diastolic dysfunction in HCM is secondary to myocardial stiffening, which appears in the early phase of ventricular filling, and to reduced compliance, which manifests itself in the later stages and is associated with changes in left atrial geometry and function.^2^The variables with higher diagnostic accuracy for detecting diastolic dysfunction are LA volume index > 34 mL/m^2^,^71 , 72^lateral and septal mitral annulus velocity < 8 cm/s on tissue Doppler, elevated pulmonary artery systolic pressure (tricuspid regurgitation flow velocity > 2.8 m/s), and MV early filling (E) velocity values;; the latter has better performance when tissue doppler is also used, by calculation of the mean E/e′ ratio (if > 14).^73^To support more difficult decisions, measuring the durations of A (via the MV) and Ar (via retrograde flow in pulmonary veins) waves may be useful; however, it should be remembered that these parameters are absent in AF, which is a common manifestation in the progression of HCM. In the presence of AF, parameters such as mitral deceleration time (< 150 msec) and increased E/e’ ratio have the best accuracy. Importantly, when there is moderate-to-severe MR, tissue Doppler velocities may be overestimated and left atrium enlargement may be even greater, and these variables should be considered individually. There is evidence that the presence of a restrictive filling pattern in LV diastolic dysfunction (grade III or IV) is associated with a worse prognosis in this group of patients, regardless of the presence of LVOT obstruction. Analysis of LA function by speckle tracking can identify changes early, with potential prognostic value for secondary outcomes, such as the development of AF. Furthermore, stress tests can provide important information on diastolic function in these patients and are indicated not only for the investigation of dynamic obstruction, but also for the early identification of diastolic dysfunction^74^. Therefore, the aforementioned parameters should be assessed after physical exertion.

### 3.4. Assessment of LVOT Obstruction and Mitral Regurgitation

Dynamic LVOT obstruction is of extreme clinical importance because of its impact on mortality and morbidity. When considering both obstruction at rest and induced by provocative maneuvers, it is found in approximately 2/3 of patients.^72 , 73 , 76^In addition to the degree and location of septal hypertrophy, changes in the valvular and subvalvular apparatus play an important role in the genesis of dynamic LVOT obstruction. The result of the dynamic interaction between the septum and the MV in obstructive phenotypes is progressive LVOT flow obstruction by the redundant anterior leaflet, a phenomenon known as systolic anterior motion (SAM). By definition, the patient is considered a carrier of the obstructive form in the presence of a peak gradient ≥ 30 mm Hg at rest or provoked by maneuvers such as the Valsava maneuver or exercise.^74^A series of morphological changes in the valvular and subvalvular apparatus help to understand the dynamic mechanism of flow obstruction and guide the etiological diagnosis of HCM in relation to other phenocopies that progress with asymmetric hypertrophy and dynamic LVOT obstruction, such as Fabry disease and cardiac amyloidosis.^76 , 77^Papillary muscle abnormalities reduce the distance between the papillary muscles and the septum. Therefore, in addition to facilitating LVOT obstruction, these changes may lead to dynamic obstruction in the middle layer of the ventricle.^26^Anterior displacement of hypertrophied papillary muscles plays a key role in the genesis of SAM, contributing to a close proximity to the septum and reducing pressure on the anterior leaflet, making it more redundant.^78^Anomalous papillary muscle insertion has different manifestations, including apical displacement and insertion abnormalities, such as direct insertion into the anterior mitral leaflet (similarly to mitral arcade) or into the LVOT. Such changes in the arrangement of the papillary muscles add to pathophysiological changes, directly and indirectly favoring dynamic obstruction LVOT obstruction.^79^Other findings favoring papillary dysfunction have been reported, such as false tendons, secondary chordae, muscle bands, anterior cusp elongation, and apical hypertrabeculation. Patients with obstructive phenotypes are more likely to experience symptoms and have a risk of SCD, which is why dynamic obstruction (affecting almost 1/3 of these patients) should be investigated with the use of provocative maneuvers such as the Valsalva maneuver, amyl nitrite inhalation, or exercise. Assessing the severity of MR in patients with LVOT obstruction is challenging, as the mechanism by which both conditions occur are related to SAM. A comprehensive assessment of LA volume, the degree of LVOT obstruction, and the extent of the regurgitant jet can provide data for at least a qualitative quantification of the degree of MR. Another limitation includes the difficulty in differentiating between MR and LVOT obstruction jets. As a general rule, outflow jet velocities > 5.5 m/s are also consisted with MR. Furthermore, the Doppler signal in MR has a rounded shape and appears during isovolumetric contraction, while the dynamic LVOT obstruction jet has a typical dagger shape with a late peaking curve, indicating a delayed protosystolic ejection murmur.

#### 3.4.1. Family Screening

TTE, complemented by clinical and ECG assessment, is the method of choice for the screening of patients with suspected HCM. The frequency of examinations in asymptomatic patients and family members of patients diagnosed with HCM varies with the patient's age, the pathogenicity of the gene implicated in the disease, and the age of symptom onset in affected family members. For asymptomatic patients and children and adolescents with pathogenic variants and/or early signs and symptoms, TTE should be performed every 1 or 2 years. In the absence of these conditions, TTE should be performed every 2 or 3 years. In adults, screening is recommended every 3 to 5 years. However, regardless of age, assessment should be conducted at any time in patients with symptoms of dyspnea, palpitations, dizziness, or syncope ( Table 4 ).

**Table 4:** Suggested frequency for screening asymptomatic family members of patients with HCM

Clinical status	TTE frequency
Children and adolescents with pathogenic variants **AND/OR** who have family member with early onset of HCM	1 to 2 years
Children and adolescents without the conditions described above	2 to 3 years
Adults	3 to 5 years
Clinical assessment, TTE, and ECG at any time in the presence of dyspnea, palpitation, dizziness, or syncope.	

TTE: transthoracic echocardiogram; HCM: hypertrophic cardiomyopathy; ECG: electrocardiogram.

CMR should be reserved for patients with poor acoustic windows for TTE or for those displaying ECG abnormalities despite apparently normal TTE results.

Table 5 shows the recommendations for the use of echocardiography in patients with HCM.

**Tabela 5:** Recomendações para emprego da ecocardiografia em pacientes com CMH

Recommendation	Grade of recommendation	Level of evidence
TTE is recommended in the initial evaluation of patients with suspected HCM.	I	B
For patients with HCM who experience a change in clinical status or a new clinical event/complication, repeat TTE is recommended.	I	B
In patients with HCM with no changes in clinical status or events, repeat TTE is recommended every 1 to 2 years to assess the degree of LVH, dynamic LVOT obstruction, MR, and LV systolic function.	I	C
For patients with HCM and a LVOT gradient < 50 mm Hg, a TTE with provocative maneuvers (Valsalva maneuver and/or orthostasis) is recommended.	I	B
For symptomatic patients with HCM who do not have a resting or provoked LVOT gradient ≥ 50 mm Hg, exercise TTE is recommended.	I	B
Exercise TTE may also be performed in asymptomatic patients with HCM who do not have a resting or provoked LVOT gradient ≥ 50 mm Hg.	IIa	C
Contrast-enhanced TTE may be performed when the characterization of LVH is inconclusive on non-contrast TTE.	IIa	C
TEE may be performed in patients with HCM in whom TTE is inconclusive or for planning SRT or further evaluation of the mitral valve apparatus.	IIa	C
For patients undergoing surgical myectomy, TEE is recommended.	I	B
TTE or TEE imaging with contrast injection is recommended to guide alcohol septal ablation, particularly in localizing the territory supplied by the targeted septal artery.	I	B
TTE within 3 to 6 months of SRT is recommended to evaluate procedural results.	I	B
In first-degree relatives of patients with HCM, a TTE is recommended as part of initial family screening.	I	B

TTE: transthoracic echocardiography; TEE: transesophageal echocardiography; HCM: hypertrophic cardiomyopathy; LVH: left ventricular hypertrophy; LVOT: left ventricular outflow tract; LV: left ventricle; SRT: septal reduction therapy.

### 3.5. Cardiac Magnetic Resonance

CMR has become an important complementary test to confirm the diagnosis, establish the prognosis, and assist in the therapeutic planning — including the prevention of sudden death — of patients with clinical suspicion or an established diagnosis of HCM. Its main advantages include satisfactory temporal resolution, good spatial resolution, and high contrast resolution, which allow the identification of areas of perfusion defects, edema, and myocardial fibrosis. CMR also assists in the identification of unconventional forms of HCM (such as apical forms), more localized hypertrophies, and the presence of other defects such as LV tip aneurysms. The great reproducibility of CMR makes it the best method to determine the volumes, masses, thickness, and systolic function of both ventricles, and the use of more modern techniques for dynamic image acquisition allows estimation of the LVOT gradient. Thus, the role of CMR in the assessment of HCM has improved significantly^26^^,^^76 - 80^( Figure 8 ).

**Figura 8:** RMC de pacientes de 49 anos com CMH com obstrução significativa da VSVE em repouso com movimento anterior sistólico do folheto anterior da valva mitral com consequente regurgitação mitral moderada. A: Imagem em cine SSFP diastólica em corte de 3 câmaras. B: Imagem em cine SSFP sistólica em corte de 3 câmaras. A seta azul mostra o movimento anterior sistólico do folheto anterior da valva mitral. A seta vermelha mostra a regurgitação mitral. C: Imagem diastólica em SSFP do eixo curto medial, mostrando maior espessura de 28 mm, no segmento anterosseptal medial. D: Realce tardio em eixo curto mostrando realce tardio com padrão mesocárdico (seta vermelha) acometendo 8% da massa do VE. VE: ventrículo esquerdo, Ao: aorta, AE: átrio esquerdo. Fonte: Arquivo pessoal dos autores.

CMR can be used to identify or confirm areas of pathological increase in myocardial thickness in patients with clinical signs or whose tests have raised the suspicion of HCM, especially if the echocardiogram is inconclusive or conflicting. In addition, CMR allows the accurate evaluation of other anatomical characteristics, such as anomalous papillary muscle insertion directly into the MV, mitral leaflet extension, accessory muscle bundles, and aberrant muscle bundles, to confirm or complete the echocardiographic evaluation. Such findings are particularly relevant when it comes to candidates for surgical or interventional treatment of myocardial hypertrophy.^26 , 76 , 78 , 81 , 82^Reduced LV contractile function, especially if accompanied by cavity dilation, is a marker of poor prognosis, and CMR can play a fundamental role in these cases, especially if the echocardiogram is inconclusive or conflicting. Similarly, localized ventricular contractility defects, such as dyskinesia and LV apex aneurysms, are more accurately assessed by CMR, as is the case of regional LV thinning, which is often associated with the presence of fibrosis and may be associated with a poor prognosis.^26 , 78 - 81 , 83^

However, the main contribution of CMR is tissue characterization, particularly with the use of late gadolinium enhancement (LGE) techniques. This imaging method adequately determines the presence and quantity of fibrosis affecting the cardiac muscle of patients with different cardiomyopathies. LGE involving more than one segment portends a worse prognosis, with increased risk of SCD due to arrythmia, while LGE involving > 15% of myocardial mass has a 2-fold increased risk. Additionally, extensive LGE involvement is associated with increased risk of progression to dilated cardiomyopathy, with development of ventricular dysfunction. Despite some controversy among researchers regarding the best way to quantify total LGE volume, it is agreed that very limited or absent LGE involvement is associated with low risk of SCD due to arrhythmia.^26 , 78 , 79 , 83 - 85^Recent studies have demonstrated that the amount of LGE is related to different mutations causing hypertrophy, and that these elements have additive prognostic value.^86^

LGE techniques are particularly useful in the differential diagnosis of other causes of myocardial hypertrophy. Most often, LGE patterns are different in cases of amyloidosis, sarcoidosis, Fabry disease, and other mutations. Thus, using this method of myocardial assessment may be crucial to establish the correct diagnosis and guide treatment. The differentiation between HCM and athlete's heart is particularly important, given the deleterious effects that strenuous exercise has on patients with HCM.^78 , 84 , 86^

Despite its relevant contributions, LGE has some limitations. The morphology of some cardiomyopathies overlaps, and the changes may not be present in earlier stages of the disease. To address these limitations, T1 mapping has emerged as another method of tissue characterization by CMR. T1 mapping allows the assessment of changes in the myocardium that may not be visually identifiable, but which are already underway and whose detection may lead to a diagnosis and adequate management. Changes in T1 values and extracellular space measurements (T1-mapping before and after infusion of the paramagnetic contrast agent) are useful to distinguish HCM from Fabry disease, as well as to differentiate increased wall thickness secondary to hypertension from HCM early.^86 - 88^Preliminary data suggest that changes in T1 values may be associated with a greater occurrence of arrhythmias, and may be implemented in SCD risk calculators in this group of patients.^89^

Table 6 shows the recommendations for the use of CRM in patients with HCM.

**Table 6:** Recommendations for the use of CMR in patients with HCM

Recommendations	Grade of recommendation	Level of evidence
CRM is recommended to confirm the diagnosis of HCM when the echocardiogram is inconclusive.	I	B
CRM is useful in the differential diagnosis of phenocopies of myocardial hypertrophy, such as athlete's heart, amyloidosis, Fabry disease, and hypertrophy secondary to hypertension.	I	B
CRM is recommended to improve risk stratification, including better analysis of anatomical changes and the presence of LGE.	I	B
CMR is recommended as a supplemental test to measure myocardial thickness, detect variations in papillary muscle anatomy, diagnose myocardial ischemia, and verify the presence of LVOT obstruction.	I	B
CMR should be considered for the identification and diagnosis of LGE to estimate the risk of sudden death and, as an additional criterion, to decide whether to implant an ICD, especially in cases where the other criteria were inconclusive.	IIa	B
CMR should be considered to assist in the evaluation of the anatomy and mechanisms of LVOT obstruction in patients who are candidates for surgery or interventional therapy.	IIa	B

CMR: cardiovascular magnetic resonance; ICD: implantable cardioverter-defibrillator; LV: left ventricle.

### 3.6. Computed Tomography

Experience with the use of CT in the study of cardiomyopathies is still limited to a few centers, but it has been shown to be useful in the assessment of LV systolic function and even for the detection of myocardial fibrosis, and may serve as an option when echocardiography is inconclusive or CMR is unavailable. Due to its high spatial resolution, CT can reveal anatomical changes involving the MV, papillary muscles, and, occasionally, atypical myocardial bundles.^90 - 92^

CT can also contribute to a more accurate assessment of the coronary arteries, identifying obstructions (which may be present in 7% to 19% of cases), diagnosing the presence of intramyocardial pathways, detecting possible significant compression by the cardiac muscle, and allowing the evaluation of the first septal branches, which is crucial in cases where interventional procedures are planned, especially in candidates for percutaneous treatment.^92 - 95^

Table 7 shows the recommendations for the use of CT in patients with HCM

**Table 7:** Recommendations for the use of CT in patients with HCM

Recommendations	Grade of recommendation	Level of evidence
CT is recommended for the noninvasive evaluation of coronary disease and the course of the coronary arteries in patients with HCM.	I	B
CT is recommended for the evaluation of coronary arteries and septal branches for planning invasive treatment of HCM.	I	B
CT should be considered for the evaluation of chest pain in patients with HCM at intermediate risk for coronary artery disease.	IIa	C
CT may be considered for the evaluation of ventricular anatomy and function in patients with suspected HCM in whom the echocardiogram is conflicting or inconclusive, for whom MRI is contraindicated, or when MRI is unavailable.	IIb	C
CT may be considered for the identification and quantification of LGE in patients with HCM in whom the echocardiogram is conflicting or inconclusive, for whom MRI is contraindicated, or when MRI is unavailable.	IIb	C

CT: computed tomography.

### 3.7. Genetic Testing

#### 3.7.1. The Importance of Genetic Diagnosis

When a clinical diagnosis has already been established, diagnostic confirmation can be obtained by DNA analysis to establish the molecular defect. The establishment of a molecular diagnosis can increase diagnostic certainty in uncertain cases in which the patient has mild-to-moderate hypertrophy, such as athletes or hypertensive patients with myocardial hypertrophy who are also suspected of having HCM ( **see Phenocopies of HCM** ).^96 , 97^

The molecular diagnosis of mutations also allows the identification of children and adults with subclinical manifestations of the disease. These individuals, especially if related to someone with HCM, would be candidates for stricter control of risk factors for HCM, as well as close surveillance.

Finally, it should be noted that a molecular diagnosis, especially in asymptomatic individuals, does not mean that the individual has the disease, but rather that he/she has an increased risk of developing it ( **see Genotype-positive, phenotype-negative** ).^98^

According to previously published studies, genetic screening of patients with HCM and their families is the most cost-effective strategy when compared with clinical screening alone.^98^Once the mutation is identified, screening of family members becomes even more important, because it allows early diagnosis and provides adequate monitoring of carriers, in addition to assuring family members who do not have the mutation that there is no risk of developing the disease. A flowchart of the genetic testing process in case of suspected HCM is shown in Figure 9 .

**Figure 9:** Genetic testing for clinical suspicion of HCM. HCM: hypertrophic cardiomyopathy.

Table 8 summarizes the advantages of applying genetic testing to patients with HCM.

**Table 8:** The advantages of genetic testing for HCM

Greater diagnostic accuracy
Better understanding of disease progression and risk assessment
Well-informed treatment and management decisions
Possibility of identifying family members with HCM and provide genetic counseling
Potential for personalized medicine based on specific genetic mutations

HCM: hypertrophic cardiomyopathy.

Next-generation sequencing (NGS) is considered the method of choice for genetic testing in HCM. It allows for a faster and cheaper diagnosis, in addition to providing the opportunity to include more genes in the analysis without a substantial increase in cost.

The number of genes and their association with different diagnoses varies according to each service provider and must be assessed individually in each case (see the most frequent mutations **in Sarcomere mutations** ).

#### 3.7.2. Sarcomere Mutations

Sarcomere mutations refer to genetic alterations in the genes responsible for the formation of the sarcomere, which is the functional unit of muscle fibers. These mutations can lead to several muscle disorders, such as HCM, dilated cardiomyopathy, and limb-girdle muscular dystrophy. The severity and type of muscle disorder caused by sarcomere mutations can vary widely, ranging from mild weakness to severe cardiac or skeletal muscle impairment.

Genetic tests made it possible to confirm that HCM is predominantly a disease of the sarcomere. A molecular diagnosis can be established in 45% to 60% of cases. The pathogenic variants described in HCM occur in several sarcomeric proteins, and the most prevalent (approximately 70%) are mutations in the genes encoding the beta-myosin heavy chain (MYH7) and myosin-binding protein C (MYBPC3). All other genes involved, including cardiac troponin I and T (TNNI3, TNNT2), tropomyosin alpha-1 chain (TPM1), and myosin light chain 3 (MYL3), present a lower frequency of pathogenic variants, and some mutations are found only in one family. Despite major advances in sequencing techniques, it is still not possible to identify the causal mutation in approximately 30% to 40% of patients with HCM.^96^Based on this data, a growing body of evidence has emerged based on registry data, which shows two distinct groups of patients with HCM: those who are sarcomeric-positive (SARC+) and those who are sarcomeric-negative (SARC-). Patients with SARC+ HCM tend to be younger at diagnosis and are more likely to have a family history of HCM or SCD, a greater degree of hypertrophy (predominantly asymmetric), myocardial fibrosis, SCD, and less LVOT obstruction at rest than patients with SARC-HCM.^86 , 99^They are also more likely to present microvascular dysfunction, which may be related to the greater development of fibrosis. Conversely, patients with SARC- HCM often present other comorbidities (hypertension and obesity), a higher prevalence of sigmoid septum and LVOT obstruction, and less fibrosis.

The identification of relevant associations between specific genes or mutations and predictable outcomes, especially those with higher risk, remains an important challenge. Recently, the international Sarcomeric Human Cardiomyopathy Registry (ShaRe) expanded this concept.^100^Involving 4,591 patients with HCM followed up for a mean of 5.4 years, it identified the presence of a sarcomere mutation as one of the predictors of adverse outcomes, including ventricular arrhythmias. It was also observed that multiple sarcomere mutations tend to present earlier with a more severe phenotype (HF requiring transplantation and ventricular assist device [VAD]).

These observations are evidence that HCM may not be exclusively a monogenic disease and may be associated with other modulating genetic factors or nongenetic factors related to exposure and other comorbidities. This is further supported by the difficulty in establishing genotype-phenotype correlations and/or the variable expressivity of the disease among patients from the same family and carriers of the same candidate genetic variant. It is possible that even genetic variants characterized as pathogenic or likely pathogenic may be influenced by epigenetic or environmental factors and other genetic variants not previously considered.^101 , 102^

Still, some genotype-phenotype associations have attracted more attention and are presented in Table 9 .^103^

**Table 9:** Genotype-phenotype associations in HCM

Gene	Phenotype
Thin filament mutations (TNNT2, TNNI3, TPM1, ACTC)	Mild and atypical hypertrophy (concentric and apical)
	Increased LV fibrosis
	Higher rates of progression to HF
	Higher probability of remodeling, leading to severe systolic or diastolic dysfunction
TNNI3 mutations	Restrictive phenotype
ACTC mutations	Apical hypertrophy
MYH7 mutations	Higher rates of AF (independent of clinical and ECG factors)
	Earlier age at diagnosis
	More frequent progression to heart transplantation
	More ventricular arrhythmias
	Higher degree of conduction abnormalities
	Restrictive phenotype
MYBPC3 mutations	Onset in older patients and lower penetrance
PLN mutations	Nonsustained ventricular tachycardia

LV: left ventricle; HF: heart failure; AF: atrial fibrillation. Adapted from Maron et al.^104^

Table 10 presents the recommendations for genetic testing in HCM.

**Table 10:** Recommendations for genetic testing in HCM

Recommendations	Grade of recommendation	Level of evidence
In patients with a phenotypic presentation of HCM, when another genetic condition is suspected, genetic testing for HCM and other genetic causes of unexplained myocardial thickening ("phenocopies") is recommended.	I	B
In patients with HCM, genetic testing should be considered to facilitate the identification of family members at risk for developing HCM.	IIa	B
In first-degree relatives of patients with HCM, genetic screening should be considered.	IIa	B
In patients with HCM, genetic testing for the assessment of SCD risk can be considered.	IIb	B
For patients with HCM who have undergone genetic testing and were found to have no pathogenic variants (i.e., harbor only benign/likely benign variants), cascade genetic testing of the family is not recommended.	III	B

HCM: hypertrophic cardiomyopathy; SCD: sudden cardiac death.

#### 3.7.3. Genotype-Positive, Phenotype-Negative Patients

Increased availability of genetic mapping for family members of patients with HCM has led to a higher prevalence of sarcomere mutation carriers (genotype-positive, G+) without phenotypic manifestations of ventricular hypertrophy on the echocardiogram or CMR (phenotype-negative, P-).^105 , 106^

The prognostic value and natural history of these patients are not well established, as there are no published studies with an adequate sample size or long-term follow-up to allow the establishment of strategies for clinical follow-up or preventive therapies. The time and degree of expression of the HCM phenotype depend on several factors, such as type of mutation, race, and sex.^107 , 108^

A study by Jensen et al.^109^included a group of 36 patients aged < 18 years who were G+/P-. After 12 years of follow-up, 6% had developed the HCM phenotype at 26 and 28 years of age, with no reports of cardiovascular events and death during this period. Vermeer et al.^110^found similar results in a group of 119 children with a mean age of 12 years, of whom only 5% developed the HCM phenotype after a mean follow-up of 6.9 ± 3.8 years, with zero cardiovascular events in the period. In younger individuals aged < 12 years who were G+/P-, 15.3% developed HCM after a follow-up of 6 years, and 52% were younger than 10 years old.^111^

In an international multicenter study conducted by Maurizi et al.,^112^203 G+/P- individuals with a mean age of 32±11 years (20% aged > 50 years) were followed up for a mean period of 6±2 years. Clinical examination, ECG, and echocardiography were performed at 1- to 5-year intervals. Approximately 10% of patients converted to the HCM phenotype (P+), with a conversion rate of 0.3%/year and a similar frequency irrespective of age group, a benign clinical course, and no HCM-related cardiovascular events.

These studies support the recommendation for clinical and imaging follow-up every 1 to 2 years from childhood to young adulthood due to the higher penetration in these age groups. In adult patients, follow-up is recommended every 3 to 5 years, as the phenotype conversion rate is lower due to lower penetration in individuals who reach this age group without expressing the HCM phenotype. The prognosis of G+/F- individuals has been benign in all age groups, with low morbidity and mortality, and rare SCD events. Therefore, there are no restrictions on participation in competitive sports or recommendations for 24-hour Holter monitoring or stress test for the assessment of SCD risk. The use of preventive pharmacological therapy and ICD for primary prevention are not recommended. Patients with a positive family history of SCD may undergo individualized assessment, although there is no evidence to support an increased risk in these patients who are G+/F.^2^

Table 11 shows the recommendations for the follow-up of individuals who are G+/P- for HCM.

**Table 11:** Recommendations for the follow-up of individuals who are genotype-positive, phenotype-negative for HCM

Recommendation	Grade of recommendation	Level of evidence
For children and adolescents up to 30 years of age, clinical follow-up, ECG, and TTE are recommended every 1 to 2 years; CMR is recommended in case of doubt.	I	C
For adults, clinical follow-up, ECG, and TTE are recommended every 3 to 5 years; CMR is recommended in case of doubt.	I	C
For patients with no family history of SCD, participation in competitive sports of any intensity can be considered.	IIa	C
For patients with no family history of SCD, participation in competitive sports of any intensity can be considered after evaluation with exercise test and 24-hour ECG every 1 to 2 years.	IIb	C
ICD implantation and pharmacological treatment are not recommended for the primary prevention of sudden death.	III	C

TTE: transthoracic echocardiogram; CMR: cardiovascular magnetic resonance; ECG: electrocardiogram; ICD: implantable cardioverter-defibrillator.

#### 3.7.4. Phenocopies of Sarcomeric HCM

Phenocopies are conditions that mimic the sarcomeric HCM phenotype, with similar imaging findings, making the diagnosis more challenging. Although phenocopies have a phenotype identical to that of HCM and a similar distribution and magnitude of wall thickening, they involve different pathophysiological mechanisms, natural history, and therapeutic strategies, some of which may even alter the clinical course of the disease when established early.^113 , 114^Clinical presentation is of utmost importance in the diagnosis of HCM phenocopies, including demographic data, laboratory tests, clinical course, and ECG findings. Imaging alone can raise or support diagnostic suspicion and guide treatment.^73 , 115 , 116^Factors that may be useful in the diagnosis include age at onset of LVH, mode of inheritance, extracardiac manifestations, and changes in complementary tests.

#### 3.7.5. Age

Age at diagnosis or of symptom onset is an important indicator of etiology and the first factor to be considered in the differential diagnosis of HCM and specific diseases. The most prevalent etiologies in addition to HCM differ according to age group: neonatal period - Pompe disease, infant of diabetic mother, and mitochondrial diseases; children aged 0 to 20 years - Danon disease and RASopathies such as Noonan syndrome and Leopard syndrome; adolescence - Friedreich's ataxia and muscular dystrophy; between 20 and 30 years - PRKAG2 and athlete's heart; 40 years - Fabry disease; after 60 years: amyloidosis and high blood pressure^73^( Figure 10 ).

**Figure 10:** Etiologies of left ventricular hypertrophy according to age at onset

#### 3.7.6. Family History

For cases with a genetic etiology, the three-generation pedigree may be useful in the differential diagnosis, as some conditions that evolve with LVH have an X-linked dominant inheritance pattern, such as Fabry disease and Danon disease.^73 , 115 , 116^These conditions can be excluded if the father of an affected male relative is also affected, allowing other diagnostic hypotheses to be made. For example, although PRKAG2 syndrome has clinical manifestations similar to that of Fabry disease, it has an autosomal dominant inheritance pattern. The mode of transmission is also relevant with regard to the magnitude of clinical expression, which is more severe in men when it is linked to the X chromosome, with therapeutic implications.^73 , 115 , 116^

#### 3.7.7. Extracardiac Manifestations

Changes in laboratory tests can reveal certain conditions, such as elevated serum creatine phosphokinase (CPK) levels in muscular dystrophies, Pompe disease, and Danon disease; and elevated TGO/TGP in Danon disease. Other clinical manifestations may also indicate specific etiologies, such as gait and balance problems in Friedreich's ataxia; cognitive impairment in Danon disease; angiokeratomas and cornea verticillata in Fabry disease; lentigines, deafness, and short stature in Leopard syndrome; macroglossia, periorbital purpura, polyneuropathy, and orthopedic changes such as bilateral carpal tunnel syndrome, spontaneous rupture of the biceps tendon, and lumbar spinal stenosis in amyloidosis; muscle weakness in muscular dystrophy; and hypertensive retinopathy in arterial hypertension^73 , 116^( Figure 11 ).

**Figure 11:** Comparison of the main HCM phenocopies. ECG: electrocardiogram; ECHO: echocardiogram; DE-MRI: delayed enhancement magnetic resonance imaging. HFpEF: heart failure with preserved ejection fraction; HF: heart failure; AVB: atrioventricular block; LV: left ventricle; LVH: left ventricular hypertrophy; LVOT: left ventricular outflow tract; SAM: systolic anterior movement. Adapted from Bruscky et al.^116^

### 3.8. ECG

The ECG may be decisive in the differential diagnosis of heart diseases that present with ventricular hypertrophy. ECG changes may precede structural changes in the heart, including LVH, and may be the only manifestation of myocardial disease. A short PR interval (< 120 ms), inferior ST depression, prolonged QRS duration, complete or incomplete right bundle branch block (RBBB), and R wave in aVL > 11 mm are indicative of Fabry disease.^117^Pre-excitation is indicative of Danon disease and PRKAG2, but may also be present in Pompe disease.^73^Changes suggestive of amyloidosis include low QRS voltage on the ECG with increased LV wall thickness on the echocardiogram, a pseudoinfarction pattern, decreased R wave progression from V1 to V3, low QRS voltage in frontal plane leads, AF, bundle branch blocks, AV blocks, and a Sokolov index < 15 mm.^118^

### 3.9. Echocardiogram

Low QRS voltages with increased LV wall thickness on echocardiography, mild pericardial effusion, interatrial and interventricular septum thickening, refractoriness of the septum, and reduced strain with a "cherry on top" pattern are findings indicative of amyloidosis.

### 3.10. CMR

CMR can help clarify structural findings on echocardiography and differentiate the causes of LVH by characterizing the location and extent of LGE. T1 and T2 mapping may also assist in this characterization. Although some diseases with LVH have typical LGE patterns, they are not always present and there is no defined specificity. In hypertensive heart disease, LGE is either absent or very discrete and does not present a typical pattern. LGE in HCM is patchy and mesocardial, predominantly located in areas with a marked degree of hypertrophy, particularly at the insertion points of the RV free wall to the interventricular septum. In amyloidosis, LGE has a circumferential subendocardial pattern, but may be absent in the early stages or difficult to detect in the later stages. Fabry disease is characterized by mesocardial LGE affecting the basal inferolateral mid-wall and, unlike other diseases that progress with LVH, low T1 mapping is characteristic ( Figure 12 ).^73^

**Figura 12:** LGE pattern and location in different types of HCM.^116^LGE: late gadolinium enhancement; MRI: magnetic resonance imaging.

### 3.11. Main HCM Phenocopies

#### 3.11.1. Cardiac Amyloidosis

Cardiac amyloidosis is caused by extracellular amyloid deposits in the myocardium and may be due to immunoglobulin deposition (AL amyloidosis) or transthyretin deposition. Cardiac involvement significantly worsens the prognosis in amyloidosis, and symptoms include angina, HF, arrhythmias, and sudden death. The condition is also characterized by multisystem involvement such as carpal tunnel syndrome, easy bruising, macroglossia, neuropathy, and hepatomegaly. The ECG typically shows low QRS voltages; while the echocardiogram shows biventricular hypertrophy with valve thickening, biatrial dilatation, and diastolic dysfunction. Echocardiography techniques such as strain and strain rate imaging, derived from speckle tracking, can help distinguish cardiac amyloidosis from HCM. Although cardiac amyloidosis typically presents with concentric and nonobstructive LVH, asymmetrical obstructive LVH patterns mimicking HCM have also been described.^120^CMR showing subendocardial LGE is pathognomonic of cardiac amyloidosis and also predicts the prognosis of the condition. As a result of its poor prognosis, the treatment of cardiac amyloidosis typically involves supportive care.^121^

#### 3.11.2. Mitochondrial Cytopathies

Mitochondrial cytopathies are a heterogeneous group of disorders caused by mutations in the maternal mitochondrial genome or in nuclear genes responsible for encoding mitochondrial proteins and which lead to dysfunctional energy production and multisystem involvement (particularly the central nervous system, heart, and skeletal system). Affected individuals may experience symptoms at any time from childhood to adulthood.

There are several types of mitochondrial cytopathies. Common symptoms that may increase diagnostic suspicion include myopathy, myalgia, fatigue not explained by myocardial dysfunction, ophthalmoplegia, hearing loss, and psychomotor difficulties.

Myocardial hypertrophy is the most common form of cardiac involvement; however, mitochondrial cardiomyopathies can also present as dilated, restrictive, or LV noncompaction cardiomyopathy (LVNC).^122^Typically, nonobstructive cardiomyopathy with mild concentric hypertrophy is found in approximately 1/4 of patients with mitochondrial disease, which represents 50% of cardiac manifestations in people with mitochondrial cardiomyopathies. It can be detected even in the antenatal period and may be the only manifestation of a mitochondrial disease or part of a multisystem disease. Obstructive hypertrophy occurs rarely, but when cardiac hypertrophy occurs, it often progresses to systolic dysfunction, followed by LV dilation. Dilated cardiomyopathy, which may be primary or secondary to ventricular hypertrophy, occurs less frequently, while restrictive cardiomyopathy is a rare manifestation of mitochondrial diseases.^123^Although LVNC is also a rare finding in mitochondrial diseases, mitochondrial diseases are highly prevalent in patients with LVNC. LVNC is more common in men and tends to develop during pregnancy in women. It may occasionally disappear during the course of the disease in some individuals with mitochondrial diseases.

In case of clinical suspicion, initial evaluation for mitochondrial disease should include blood analysis with complete red blood cell count, creatine phosphokinase, transaminases, albumin, lactate and pyruvate, amino acids, and acylcarnitines, along with quantitative or qualitative urinary organic acids.

Mitochondrial diseases are caused by mutations in the maternally inherited mtDNA or in one of the many genes that encode mitochondrial proteins but are located in the nuclear genome. mtDNA genome sequencing and heteroplasmy analysis can now be performed in blood. Overall, the advent of newer technologies that rely on NGS methodologies have emerged as the gold standard methodology for mtDNA genome sequencing. Therefore, the initial genetic test to evaluate mitochondrial disease in blood, urine, or tissue, depending on symptom presentation and sample availability, should be complete sequencing of the mitochondrial genome. In the case of high clinical suspicion but negative genetic testing, exome sequencing of the nuclear genome is recommended. Special attention should be paid to mutations in the nuclear genome causing mitochondrial disease in cases diagnosed in adulthood.^124^

Identification of a mitochondrial disease-causing mutation allows families to end their diagnostic journey and receive appropriate genetic counseling, carrier testing, and selective prenatal diagnosis.

#### 3.11.3. Barth Syndrome

Barth syndrome is an X-linked disorder characterized by cardiomyopathy, skeletal myopathy, growth retardation, neutropenia, and increased urinary levels of 3-methylglutaconic acid. It is caused by mutations in the TAZ gene that codes for tafazzin, a phospholipid transacylase located in the inner mitochondrial membrane and which plays an important role in cardiolipin remodeling. LVNC and dilated cardiomyopathy are the most common phenotypes, while HCM appears to be less common. Other cardiac manifestations of Barth syndrome are arrhythmias (including supraventricular and ventricular tachycardia) and sudden death.^125^

Other diseases in this group that may be associated with cardiomyopathy are caused by mutations in DNAJC19, TMEM70, and AGK. 3-Methylglutaconic aciduria associated with DNAJC19 mutations (dilated cardiomyopathy and ataxia syndrome) results from deficient mitochondrial protein import and is characterized by LVNC or dilated cardiomyopathy, nonprogressive cerebellar ataxia, testicular dysgenesis syndrome, and growth failure. Mutations in TMEM70 (mitochondrial complex V deficiency), encoding a protein involved in the insertion of ATP synthase (complex V) into the mitochondrial membrane, result in multisystem mitochondrial disease with HCM. Sengers syndrome, caused by mutations in AGK, might also be accompanied by 3-methylglutaconic aciduria and is characterized by HCM, cataracts, myopathy, exercise intolerance, and lactic acidosis. The AGK gene product is an acylglycerol kinase and is involved in the assembly of ANT1, a mitochondrial adenine nucleotide transporter.

#### 3.11.4. Friedreich's Ataxia

Friedreich's ataxia is an autosomal recessive neurodegenerative disorder caused by mutations in FXN, which encodes frataxin, a mitochondrial iron-binding protein involved in the synthesis of the iron-sulfur clusters required by the electron transport chain. The clinical presentation includes progressive ataxia after adolescence, dysarthria, loss of lower limb reflexes, peripheral sensory neuropathy, and diabetes. HCM is the most common cardiac manifestation.^126^

The identification of Friedreich's ataxia as the cause of ventricular hypertrophy depends on the identification of extracardiac manifestations, which include progressive ataxia and lower limb weakness, dysarthria, nystagmus, and loss of proprioception, and may also include scoliosis, diabetes, and impaired vision and hearing. Most patients will progressively lose the ability to walk. Cardiac involvement is rarely found in other hereditary ataxias. However, the phenotype is highly variable, and, in some patients, the first manifestation is cardiomyopathy.

From a cardiovascular point of view, there are a series of phenotypic manifestations, including concentric/asymmetric hypertrophy or dilated cardiomyopathy. Concentric/asymmetric hypertrophy is less common, but dilated cardiomyopathy with arrhythmia is more often associated with mortality. The systolic function of patients with Friedreich's ataxia may show a decline at the end of life. However, there are few data from long-term prospective studies of cardiac progression in these patients, and the cause of death is often attributed to HF and arrhythmia.^127^

Echocardiographic findings include a granular appearance similar to that seen in amyloidosis, although without pericardial effusion or biatrial enlargement. CMR studies detect LV fibrosis and concentric remodeling even before hypertrophy, together with a decreased myocardial perfusion reserve.

#### 3.11.5. Glycogen Storage Disease

Several important features can help to distinguish glycogen storage disease (GSD) from LVH with sarcomeric gene defects. These include evidence of multisystem clinical features at an early age, massive LVH (stimulated by glycogen-filled vacuoles rather than sarcomeric disarray or cellular fibrosis that is characteristic of sarcomeric LVH), early progression to dilated cardiomyopathy, and ECG abnormalities such as ventricular pre-excitation and conduction system defects.^128^Ventricular pre-excitation in these conditions has been considered to result from the unique mechanism of disruption of the annulus fibrosus by glycogen-filled myocytes rather than the presence of histologically distinct bypass tracts. The typical characteristics of each condition are detailed below.

It is currently known that four metabolic storage cardiomyopathies (PRKAG2, LAMP2 [Danon disease], Pompe disease, and Forbes disease) present a clinical LVH phenotype that mimics the expression of "typical" sarcomeric LVH and function as HCM phenocopies. Although these metabolic cardiomyopathies comprise only a small fraction of adult patients with unexplained LVH (< 1%), diagnostic distinction from HCM is crucial because their natural history, prognosis, and, in some cases, treatment strategies are markedly different.

Cardiomyopathy caused by mutations in PRKAG2 has an autosomal dominant pattern of inheritance. The morphologic expression of PRKAG2 is typically a symmetric pattern of LVH in which wall thickness is largely similar in all segments of the chamber, but often massive (> 30 mm), in the absence of outflow tract obstruction. This phenocopy is associated with a spectrum of ECG conduction abnormalities, most commonly ventricular pre-excitation (often diagnosed as Wolff-Parkinson-White [WPW]) and bundle branch block. Adverse events related to the disease typically occur in the third and fourth decade of life, most commonly atrial fibrillation/flutter or conduction disease that may require a pacemaker. The risk of SCD and progression to significant systolic dysfunction appears to be similar to that of HCM, which makes the diagnosis more difficult.^129^

Danon disease is caused by a deficiency in lysosome-associated membrane protein-2 (LAMP2) and mainly affects cardiac and skeletal muscle cells, with neurologic manifestations. The disease is characterized by severe cardiomyopathy, mild skeletal myopathy, ophthalmological abnormalities, and different degrees of intellectual disability and psychiatric symptoms. Age of onset ranges from childhood to adulthood. Female carriers generally show signs at an older age and have a lower incidence of intellectual disability. Cardiomyopathy may be the initial manifestation and is typically hypertrophic, although dilated cardiomyopathy may also occur, and cardiac arrhythmias, due in part to progressive fibrosis, are a common cause of sudden death.^130^

Even in the absence of muscle weakness, subtle myopathy is almost always present in men, manifesting with persistently elevated CPK levels. Male patients often undergo repeated gastroenterology tests due to elevated transaminases, with no other indication of liver disease. Neuropsychiatric manifestations range from intellectual disability or psychosis to even minor behavioral abnormalities, attention deficit disorder, anxiety disorder, or may be absent altogether. Pigmentary retinopathy is common but rarely leads to clinically overt visual disturbances.

Clinical suspicion of the condition should prompt testing of serum CPK and liver enzyme levels, both of which are typically elevated in this condition. The ECG may show WPW-like changes (short PR interval and pre-excitation) in up to 2/3 of men and less than 1/3 of women. This finding in combination with increased QRS voltage, especially among male teenagers, should also raise suspicion of this condition. Skeletal muscle biopsy shows intra-sarcoplasmic periodic acid-Schiff-positive vacuoles. Although echocardiography usually shows severe concentric LVH, asymmetric septal hypertrophy may also be present in some patients, resembling the pattern seen in sarcomeric HCM. In later stages, changes of dilated cardiomyopathy predominate. Molecular genetic screening revealing a mutation in the LAMP2 gene confirms the diagnosis. There is no specific medical therapy available for the condition. ICD implantation should be considered, although severe hypertrophy is associated with high defibrillation thresholds and failure to terminate ventricular fibrillation (VF).

#### 3.11.6. Cardiac Oxalosis

Cardiac oxalosis is an extremely rare cause of LVH. It consists of extracellular deposition of oxalate due to an overproduction caused by a deficiency of the peroxisomal liver enzyme alanine-glyoxylate aminotransferase. It is part of the so-called primary hyperoxalurias, rare autosomal recessive disorders of oxalate metabolism. The cardiac features found in this condition are HF resulting from systolic dysfunction and restrictive cardiomyopathy, LVH in 1/3 of patients, and rhythm disorders. Other clinical manifestations include urolithiasis, nephrocalcinosis, and progressive renal failure.^131^

#### 3.11.7. Fabry Disease

Fabry disease (also known as Anderson-Fabry disease or angiokeratoma corporis diffusum) is a rare disorder of α-galactosidase A metabolism, a lysosomal enzyme that degrades neutral glycosphingolipids, mainly globotriaosylceramide (Gb3). Changes in the enzyme result in progressive intracellular lipid accumulation.

Cardiac involvement is present in all forms of Fabry disease, with concentric LVH being the most common presentation.^132^The prevalence of patients with Fabry disease among those previously diagnosed with HCM ranges from 0.5% to 12%, depending on the reported series.^133^

The ECG is a very useful tool to suggest the diagnosis (LVH criteria and short PR interval) and is also useful in risk stratification, such as with the presence of AF and conduction system disease, particularly complete atrioventricular block (AVB).^117^A short PR interval, which may be present in children under 10 years of age, is seen in approximately 40% of patients; interestingly, it is not a result of an accessory pathway (or as suggested in PRKAG2 cardiomyopathy), but rather of accelerated atrioventricular (AV) conduction.^134^Some studies report an incidence of AF on ambulatory ECG monitoring of 17%.^135^Ventricular arrhythmias may be present in 8% of cases,^136^and angina has also been reported in some patients with Fabry disease. This is most commonly seen in combination with LVH and may be due to an increase in oxygen demand for reasons similar to those occurring in HCM, but also in response to diffuse arteriopathy associated with cellular damage to the arterial walls. For example, the prevalence of aortic dilation increases with age, is more prevalent in men, and generally affects the sinus of Valsalva and the ascending aorta.^137^

The importance of identifying Fabry disease lies in the existence of specific therapy for the disease.^138^

#### 3.11.8. Noonan Syndrome and Other RASopathies

The RASopathies are a group of multisystem syndromes caused by germline mutations in genes that encode signal transducers and regulatory proteins functionally linked to the RAS/mitogen-activated protein kinase (MAPK) pathway. Collectively, these disorders have an estimated prevalence of 1 in 1,000 to 1 in 2,500 live births. According to a large clinical registry, RASopathies may account for the underlying diagnosis in approximately 18% of childhood HCM, especially among infants under 1 year of age, in whom they represent approximately 42% of cases.^126^

The RASopathies include Noonan syndrome (NS), NS with multiple lentigines (NSML, formerly known as LEOPARD syndrome), cardiofaciocutaneous syndrome (CFC), Mazzanti syndrome (also known as NS-like disorder with loose anagen hair), Costello syndrome (CS), neurofibromatosis type 1, and Legius syndrome, which are well recognized and clinically characterized. However, other clinically related conditions are emerging.

The extent of clinical variability characterizing each RASopathy appears to be related to the extent of molecular variability and heterogeneity of these diseases. For example, NS, which is the most common RASopathy, is caused by mutations in more than 10 genes (such as PTPN11, SOS1, SOS2, NRAS, KRAS, MRAS, RRAS2, RIT1, LZTR1, RAF1, MAP2K1), which are mostly associated with certain characteristics, including adequate growth and cognition (SOS1, SOS2), high prevalence of pulmonary stenosis (PTPN11), and HCM (e.g., RAF1, MRAS, and RIT1). Conversely, other RASopathies are relatively homogeneous, being caused by a narrow spectrum of mutations in a single gene, as in the case of CS and Mazzanti syndrome, which are caused by a set of mutations in HRAS and SHOC2, respectively.^139^

Myocardial hypertrophy in RASopathies is more prevalent during childhood when compared with HCM. In 5% to 10% of cases, hypertrophy in RASopathies is associated with a severe clinical presentation, particularly in infants with signs of HF, with a 1-year mortality rate of 70%.^140^With the exception of these cases, the clinical status tends to improve over time, and the progression of LVH described in RASopathies is unusual. A process of inverse LV remodeling with regression of z-values of myocardial wall thickness measurements over time in serial echocardiographic examinations has been reported in many clinical studies.^141^

## 4. Treatment

Within the natural history of HCM, treatment options and clinical follow-up vary according to each individual presentation. An overview of treatment options is shown in Figure 13 .

Family members of individuals with G+/P- HCM who carry a mutation should be monitored periodically. This is further addressed in a specific section of these Guidelines.

Roughly 50% of individuals with the HCM phenotype may be asymptomatic and have a benign clinical evolution with a low risk of SCD, for whom drug treatment or interventions are not necessary. They should undergo periodic assessment of risk stratification and disease progress.

The other 40% to 50% of patients with clinically manifest CHM may experience complications such as an increased risk of SCD and symptoms of HF and AF. Therapy options for these conditions are summarized in Figures 13 , 14 , and 15 and will be further discussed in the following sections of these Guidelines.

**Figure 13:** Flowchart of treatment options for HCM-related complications. HCM: hypertrophic cardiomyopathy; SCD: sudden cardiac death; ICD: implantable cardioverter-defibrillator; ODT: optimal drug therapy.

**Figure 14:** Flowchart of treatment options for HF in obstructive HCM. HCM: hypertrophic cardiomyopathy; LVEF: left ventricular ejection fraction.

**Figure 15:** Flowchart of treatment options for HF in nonobstructive HCM. HCM: hypertrophic cardiomyopathy; SCD: sudden cardiac death; EF: ejection fraction; CCB: calcium channel blocker; HFrEF: heart failure with reduced ejection fraction; ACEi: angiotensin-converting enzyme inhibitors; ARB: angiotensin receptor blocker; ARNI: angiotensin receptor-neprilysin inhibitors; MRA: mineralocorticoid receptor antagonist; SGLT2i: sodium-glucose cotransporter 2-inhibitors; ICD: implantable cardioverter-defibrillator.

### 4.1. Drug Therapy

Pharmacological treatment is the first option for patients with symptomatic HCM, whether obstructive or nonobstructive. However, there is no clear evidence of its role in reducing mortality in this population. Still, optimal drug therapy (ODT) can significantly improve symptoms and reduce the need for SRT procedures in obstructive cases. In the next sections, we will discuss the main classes of drugs indicated for the treatment of HCM.

#### 4.1.1. Beta-Blockers

Beta-blockers are indicated for symptomatic relief (e.g., angina, dyspnea) in individuals with both obstructive and nonobstructive HCM. This indication is based on the expected beneficial effects associated with beta-adrenergic blockade, such as increased diastolic time, improved coronary perfusion, reduced myocardial oxygen consumption, reduced risk of tachyarrhythmias, and, consequently, reduced risk of SCD. The inotropic effects of catecholaminergic stimulation are known to increase LVOT obstruction gradients, and this knowledge has promoted an attempt to treat LVOT obstruction with beta-adrenergic receptor blockers.^142 , 143^

A very recent randomized, crossover clinical trial with 29 patients comparing metoprolol vs placebo showed that metoprolol reduced LVOT obstruction at rest and during exercise, provided symptom relief, and improved quality of life in patients with obstructive HCM.^144^Despite the infancy of the evidence, there is a consensus among experts that beta-blockers should be used as first-line therapy in HCM.

##### 4.1.1.1. Non-Dihydropyridine Calcium Channel Blockers

The nondihydropyridine calcium channel blockers (CCBs) verapamil and diltiazem may provide symptom relief for patients with obstructive HCM. Both agents have vasodilatory properties, in addition to negative inotropic and chronotropic effects, and have been recommended for the symptomatic treatment of HCM in several guidelines.^2 , 3^Verapamil (initial dose of 40 mg, three times a day, up to a maximum of 480 mg per day) is the most studied and may be indicated when beta-blockers are contraindicated or ineffective. However, strict monitoring is necessary in patients with severe obstruction (≥ 100 mm Hg) or elevated pulmonary artery systolic pressure, as it can cause pulmonary edema.^3^

Rosing et al.^145^assessed the hemodynamic effects of intravenous verapamil in 27 patients with HCM. The authors observed that increasing doses of verapamil produced small increases in heart rate (from 72 ± 3 to 81 ± 6 bpm, p < 0.01) and a considerable reduction in systolic blood pressure (from 118 ± 8 to 99 ± 5 mm Hg, p < 0.005). A high dose (0.021 mg/kg/min) decreased the baseline LVOT gradient from 94±14 to 49±14 mm Hg (p < 0.05) and the mean LVOT gradient during the Valsalva maneuver from 76±5 to 63±13 mm Hg (p < 0.01).

The long-term clinical effects of verapamil were demonstrated in an observational study with 78 participants. Of the total number of patients, 54% experienced an improvement in symptoms. Functional capacity assessed by stress test demonstrated an increase in exercise time after 5 days of starting verapamil by 3.1±0.6 minutes. A further increase of 2.3 ± 0.6 minutes (25±7%, p < 0.0025) over the initial value with verapamil was recorded at the patients’ last visit (median 12 months after initiation of therapy).^146^

Regarding the adverse effects of nondihydropyridine CCBs in HCM, episodes of hemodynamic collapse have been reported in patients with elevated LVOT gradients at rest (above 80 to 100 mm Hg) and symptoms of HF taking verapamil, with reports of significant bradycardia and low cardiac output.^147^

Therefore, despite the limited evidence, nondihydropyridine CCBs (verapamil or diltiazem) are recommended to control symptoms in patients with obstructive or nonobstructive HCM, replacing beta-blockers when not tolerated or ineffective (class I, level of evidence C).

#### 4.1.2. Cardiac Myosin Inhibitors

##### 4.1.2.1. Mavacamten

Mavacamten is a small-molecule selective allosteric inhibitor of the cardiac myosin ATPase developed to mitigate actin-myosin cross-bridge interactions, thereby reducing cardiac contractility. Its primary clinical attribute is reducing LVOT pressure gradient, based on negative inotropic properties, similar in this regard to disopyramide.^148^The EXPLORER-HCM clinical trial evaluated the clinical benefit of mavacamten (titrated from 2.5 to 15 mg per day) more consistently. The randomized, placebo-controlled, 30-week trial included 251 patients with symptomatic obstructive HCM (LVOT gradient > 50 mm Hg and NYHA FC II-III) currently using beta-blockers or CCBs (> 90% of participants) as first-line therapy. The primary endpoint was an improvement in peak oxygen consumption (pVO_2_) during exercise of 3.0 mL/kg/min without worsening of NYHA FC or an improvement in pVO_2_of 1.5 mL/kg/min with a reduction in NYHA class. Only 37% of patients on mavacamten achieved the primary endpoint vs 17% of patients in the placebo group (p < 0.0001). Mavacamten also significantly reduced post-exercise LVOT gradients and improved patients’ FC. Mavacamten was shown to be safe and well tolerated, with an overall adverse event profile comparable to placebo.^149^A subsequent study evaluated the benefit of mavacamten on quality-of-life scores (Kansas City Cardiomyopathy Questionnaire [KCCQ]). The proportion of patients with a marked change (KCCQ-Overall Summary ≥ 20 points) was 36% (33 of 92) in the mavacamten group vs 15% (13 of 88) in the placebo group, with an estimated absolute difference of 21% (95%CI 8.8-33.4) and a number needed to treat of 5 (95%CI 3-11). These gains returned to baseline after stopping treatment.^150^An echocardiographic substudy demonstrated that more patients using mavacamten (80.9%) compared with placebo (34.0%) had resolution of SAM of the MV after 30 weeks (p < 0.0001). Mavacamten also improved measures of LV diastolic function, including atrial volume index, lateral E/e’, and NT-proBNP levels, findings that suggest improvement in markers of the pathophysiology of obstructive HCM.^151^In another substudy involving only 35 patients (mavacamten, n = 17; placebo, n = 18), CMR data demonstrated that patients who received mavacamten had a greater reduction in LV mass index (mean between-group difference, -15.8 g/m^2^[95%CI -22.6 to -9.0]; p < 0.0001). There was also a more marked reduction in LVEF with mavacamten when compared with placebo (-6.6% [6.39%] vs -3.9% [7.7%]; p = 0.002), but no significant between-group differences in LGE and extracellular volume were observed.^152^

The VALOR-HCM study investigated the use of mavacamten in 112 patients with obstructive HCM with an indication for SRT based on current guideline recommendations. The primary endpoint was the proportion of patients who underwent SRT or remained eligible for this therapy after 16 weeks of treatment. Most of the enrolled patients were in NYHA FC III-IV with a mean post-exercise LVOT gradient of 84 mm Hg. After 16 weeks, 43 of 56 patients in the placebo group (77%) and 10 of 56 patients receiving mavacamten (18%) met guideline criteria or underwent SRT (p < 0.0001).^153^On January 2, 2023, the Brazilian Health Regulatory Agency (Anvisa) approved the use of mavacamten in Brazil for patients with symptomatic obstructive HCM in NYHA FC II-III.

This evidence indicates that mavacamten should be considered for symptom relief in adult patients with obstructive HCM, LVEF > 55%, resting or provoked intraventricular gradient > 50 mm Hg, and NYHA FC II-III despite the use of beta-blockers or nondihydropyridine CCBs in maximum tolerated doses (grade of recommendation IIa, level of evidence B) ( Table 12 ).

**Tabela 12:** Recomendações para o tratamento medicamentoso da CMH

Recommendation	Grade of recommendation	Level of evidence
The use of beta-blockers is recommended in symptomatic patients to attenuate symptoms in both obstructive and nonobstructive HCM.	I	B
The use of nondihydropyridine CCBs is recommended in patients who are intolerant to beta-blockers or when beta-blockers are ineffective, both in obstructive and nonobstructive HCM.	I	B
The use of mavacamten should be considered in adult patients with obstructive HCM, intraventricular gradient > 50 mm Hg at rest or provoked, LVEF > 55%, and NYHA FC II or III who remain symptomatic despite the use of beta-blockers or nondihydropyridine CCBs at maximum tolerated doses to reduce symptoms and the intraventricular gradient.	IIa	B
The use of non-dihydropyridine CCBs should be considered in combination with beta-blockers to control heart rate and improve symptoms.	IIa	C
The use of angiotensin-II AT1 receptor blockers may be considered to improve physical capacity and control systemic arterial hypertension.	IIb	B
The use of vasodilators should be avoided, especially in patients with obstructive HCM.	IIa	C

11 HCM: hypertrophic cardiomyopathy; CCBs: calcium channel blockers; LVEF: left ventricular ejection fraction; NYHA FC: New York Heart Association functional class.

The ODYSSEY-HCM, a randomized, double-blind, placebo-controlled clinical trial, is underway to evaluate the safety, tolerability, and efficacy of mavacamten in adults with symptomatic nonobstructive HCM (ClinicalTrials - Protocol CV027031).

##### 4.1.2.2. Aficamten

Aficamten is the second cardiac myosin inhibitor being evaluated in clinical studies. It has a shorter half-life than mavacamten, reaches a steady-state concentration within 2 weeks, and appears to have a wider therapeutic window. In the randomized, placebo-controlled phase II study REDWOOD-HCM (n = 41), high doses of aficamten (10-30 mg daily) had a favorable safety profile and reduced gradients at rest (mean difference: -40±27 mm Hg and 43±37 mm Hg in Cohorts 1 and 2, p = 0.0003 and p = 0.0004 vs. placebo, respectively) and with Valsalva (-36±27 mm Hg and -53±44 mm Hg, p = 0.001 and < 0.0001 vs. placebo, respectively).^154^Phase III clinical trials are needed to document the benefits and safety of aficamten and to allow the development of recommendations for its use.

##### 4.1.2.3. Disopyramide

Disopyramide is a type I antiarrhythmic drug with a negative inotropic effect and can be considered a potential alternative in the treatment regimen for HCM with LVOT obstruction.^155^

In a few case series including populations with HCM, disopyramide was effective in reducing LVOT gradients.^155 - 157^The drug was tested in a multicenter retrospective study comparing 118 participants on disopyramide vs 373 participants who did not receive the drug for a mean follow-up period of 3.1±2.6 years. The study demonstrated that 66% of patients did not require additional invasive intervention, and annual all-cause cardiac mortality rates were not statistically different between the disopyramide and control groups (1.4% vs. 2.6%/year, p = 0.07). SCD rates also did not differ between the treatment and control groups (1.0%/year vs 1.8%/year).

The safety of outpatient initiation of disopyramide was investigated in a large case series by Adler et al.^158^Of 2,015 patients with HCM seen at the clinic, 168 were started on disopyramide. During long-term follow-up (mean 447 days), 38 patients (23%) developed side effects of disopyramide and 18 (11%) discontinued the drug because of these side effects. Disopyramide at a dose of 300 mg prolonged the mean QTc interval by 19±23 ms. Anticholinergic effects, such as xerostomia, xerophthalmia, urinary retention, and constipation may occur, and these symptoms can be reduced with the concomitant use of pyridostigmine. Accelerated AV node conduction can also cause rapid conduction through this structure in the event of AF.^159^

Contemporary guidelines recommend the use of disopyramide for patients who do not respond to beta-blockers or CCBs, preferably in combination with some of these drugs, to reduce the symptoms of HCM with dynamic LVOT obstruction at rest or provoked.^2^However, disopyramide is not available in Brazil.

##### 4.1.2.4. Renin-Angiotensin-Aldosterone System Inhibitors

Renin-angiotensin-aldosterone system (RAAS) inhibitors are reserved for patients with nonobstructive HCM who developed heart failure with reduced ejection fraction (HFrEF). In patients with nonobstructive HCM without LV systolic dysfunction, the use of this therapeutic strategy should be evaluated on an individual basis due to the lack of clear benefits on this population.

Among RAAS modulators, angiotensin II AT1 receptor blockers (ARBs) have been the most studied. Based on the rationale behind the use of ARBs for the reduction of hypertrophy in hypertensive patients, a randomized study compared the effect of losartan vs placebo on the reduction of ventricular mass in patients with HCM. Despite not demonstrating significant between-group differences, the trial showed good patient tolerance to the drug, without an increase in syncope episodes. Losartan was also able to reduce blood pressure, being an option in the treatment of arterial hypertension concomitant to HCM.^147^

A pool of studies with the same class of drugs was included in a recent meta-analysis, and it was observed that the use of ARBs showed no benefit in reducing ventricular hypertrophy or improving EF.^160^

The Valsartan for Attenuating Disease Evolution in Early Sarcomeric Hypertrophic Cardiomyopathy (VANISH) trial was a randomized phase 2 study including 178 patients (mean age: 20-30 years) with sarcomeric HCM. Participants were randomly assigned to receive valsartan (320 mg daily in adults; 80-160 mg daily in children) or placebo. The study included multiple composite endpoints consisting of structural, functional, and biomarker changes. The results suggested an improvement in the composite primary endpoint, indicating an opportunity to attenuate disease progression. However, this response should be further evaluated in studies with a longer follow-up.^161^

### 4.2. SRTs

One of the noteworthy characteristics of HCM is the strong association between symptoms and LVOT obstruction, with resolution of obstruction being a recognized therapeutic target. Although negative inotropic agents can attenuate LVOT obstruction and symptoms in many patients, approximately 5% to 10% remain refractory to pharmacological therapy.^143^

Thus, SRTs are invasive alternatives for patients with obstructive HCM who present resting or provoked LVOT gradients > 50 mm Hg and remain symptomatic (NYHA FC III-IV) despite ODT. Currently, SRTs include alcohol septal ablation (ASA), surgical myectomy, and radiofrequency ablation.

#### 4.2.1. ASA

Selective injection of alcohol into the septal perforator branch to create a localized septal scar was first proposed in 1994,^162^with the aim of reducing the intraventricular gradient in patients with obstructive HCM. ASA also reduces the degree of MR and LV end-diastolic pressure, consequently improving symptoms of dyspnea, increasing functional capacity and quality of life, and contributing to secondary effects such as reduction in AF burden and severity of pulmonary hypertension.^163 - 169^

Since its advent, the procedure has significantly improved. ASA is now an important alternative to surgical myectomy in symptomatic patients, as it presents lower rates of periprocedural complications, including chest pain (due to avoiding sternotomy), shorter hospital stays, and mortality rates < 1% in experienced centers.^165 , 170^However, there is no randomized study comparing the effect of ASA vs surgical myectomy on the reduction of the interventricular gradient. Therefore, the literature mostly consists of registries, sometimes with propensity scores and long-term follow-up. Compared with surgical myectomy, ASA is associated with less intraventricular gradient reduction, especially in patients with very high baseline gradients (> 100 mm Hg) and massive septal hypertrophy (> 30 mm), despite similar improvements in FC.^171 , 172^Conversely, the presence of intrinsic disease of the mitral apparatus or papillary muscle favors myectomy. In these cases, the obstruction is caused by elongation of the MV leaflets, especially the anterior one, and by the malposition of the papillary muscles (especially the lateral one), inserted anteriorly. They change the position of the MV coaptation zone, which is displaced anteriorly.^169^

Transcatheter MV edge-to-edge repair (MitraClip) may be useful in reducing the LVOT gradient and relieving HF symptoms in patients with HCM and refractory symptomatology linked to LVOT obstruction for whom SRT is contraindicated. In a review of 4 studies conducted between 2010 and 2016 (15 patients, mean age 79.5 ± 8.1 years), there was an immediate gradient reduction from 75.8 ± 39.7 to 11.0 ± 5.6 mm Hg, but patients were only followed for a maximum of 6 months.

Furthermore, in long-term follow-up, overall mortality and the occurrence of SCD after ASA were similar to those observed with surgical myectomy, as well as in relation to controls with nonobstructive HCM.^164 , 174 - 176^

The main periprocedural complication of ASA is RBBB and, eventually, advanced AVB, with 7% to 20% of cases requiring a permanent pacemaker.^166 - 168 , 175 , 177^In a recent meta-analysis including 4,213 patients undergoing ASA, the rate of permanent pacemaker implantation was 10% vs 5% after surgical myectomy.^164^Previous studies determined that advanced age, presence of LBBB, periprocedural AVB, and QRS interval duration > 120 ms were predictors of the need for a permanent pacemaker.^178 - 180^Typically, 48-hour telemetry is recommended, with placement of a temporary pacemaker, especially in circumstances of higher risk for advanced AVB.^179 - 181^24-Hour Holter monitoring prior to hospital discharge is also recommended.

Due to the variability of the septal blood supply, monitoring with contrast-enhanced TEE or TTE is essential during the procedure.^182 - 185^If the contrast agent cannot be localized exclusively to the basal septum at and adjacent to the point of mitral-septal contact, the procedure should be abandoned due to the risk of complications.^182 - 184^Injection of large volumes of alcohol (> 2-3 mL) in multiple septal branches with the aim of gradient reduction is not recommended due to the high risk of complications and arrhythmic events.^186^Recurrence of ventricular septal defect (VSD) as a complication of ASA or surgical myectomy has been rarely reported in current series.^164^However, it may be more frequent in patients with mild septal hypertrophy (< 16 mm).^3^In this case, alternative therapies may be recommended for the treatment of selected patients refractory to clinical treatment.

Table 13 shows the recommendations for angiography and invasive hemodynamic monitoring.

**Table 13:** Recommendations for angiography and invasive hemodynamic monitoring

Recommendation	Grade of recommendation	Level of evidence
Invasive hemodynamic monitoring is recommended in patients with HCM who are candidates for SRT and in whom there is doubt regarding the presence and severity of LV outflow tract obstruction on noninvasive examinations; invasive left heart catheterization with a manometer may be recommended.	I	B
Invasive hemodynamic monitoring is recommended in patients with symptomatic HCM with evidence of ischemia; coronary angiography (MDCT or invasive) is recommended.	I	B
Invasive hemodynamic monitoring is recommended in patients with HCM who have risk factors for coronary atherosclerosis; coronary angiography (MDCT or invasive) is recommended before surgical myectomy.	I	B

HCM: obstructive hypertrophic cardiomyopathy; LV: left ventricle; MDCT: multidetector computed tomography.

Table 14 shows the recommendations for SRT in obstructive HCM.

**Table 14:** Recommendations for SRT in obstructive HCM.

Recommendations	Grade of recommendation	Level of evidence
SRT is recommended for patients with obstructive HCM with a resting or provoked LVOT gradient > 50 mm Hg in NYHA functional class III-IV despite optimal medical therapy.	I	B
SRT is recommended in patients with exertional syncope caused by a resting or provoked LVOT gradient > 50 mm Hg despite optimal medical therapy.	I	B
SRT should be performed by experienced operators, working in a Heart Team with experience in managing patients with HCM.	I	C
SRT is recommended for patients with obstructive HCM with significant symptoms (NYHA FC III-IV) despite optimal medical therapy, in whom surgery is contraindicated or has high risks due to the presence of important comorbidities or advanced age; ASA is recommended in the presence of favorable anatomy and should be performed in experienced centers.	I	B
Surgical myectomy should be preferred over ASA in patients with an indication for SRT and requiring other surgical intervention (i.e., mitral repair or replacement, papillary muscle intervention, multivessel coronary artery disease, aortic stenosis).	I	B
TTE or TEE imaging with contrast injection is recommended to guide ASA, particularly in localizing the branch directed toward the septal portion.	I	B
For symptomatic patients with obstructive HCM, septal reduction therapy performed at experienced centers in eligible patients can be considered as an alternative to increasing medication after a shared decision including the risks and benefits.	IIb	B
SRT may be considered for symptomatic patients in NYHA FC II in case of progressive pulmonary hypertension caused by LVOT obstruction or MR, LA enlargement with more than 1 episode of AF, low functional capacity attributed to obstruction in stress test (gradients > 100 mm Hg) in young adults and children.	IIb	B
In asymptomatic patients with obstructive HCM and normal exercise capacity, SRT is not indicated.	III	C
In patients with symptomatic obstructive HCM in whom SRT is an option, mitral valve replacement should not be recommended as an option to clear the LVOT.	III	C

SRT: septal reduction therapy; HCM: hypertrophic cardiomyopathy; LVOT: left ventricular outflow tract; NYHA FC: New York Heart Association functional class; ASA: alcohol septal ablation; TTE: transthoracic echocardiography; TEE: transesophageal echocardiography; MR: mitral regurgitation; LA: left atrium; AF: atrial fibrillation.

#### 4.2.2. Septal Myectomy

Septal myectomy for LVOT clearance was described by Morrow more than 60 years ago. A rectangular knife is used for the resection of the most prominent part of the interventricular septum, immediately below the aortic annulus, the site at which it is opposed by the anterior mitral leaflet. Septal myectomy significantly reduces or even eliminates the LVOT gradient in approximately 90% of cases, promoting a reduction in SAM, improved exercise capacity, and symptom relief.^187 , 188^

In addition to improving quality of life, surgical myectomy is associated with a life expectancy similar to that of the general population without disease and significantly higher than that of patients with HCM and LVOT obstruction who have not undergone surgery.^189^

In the past 2 decades, in parallel with the use of TTE in most cardiovascular procedures, a need to expand the surgical resection of excess muscle in the LVOT and the rest of the intraventricular region for the treatment of obstructive HCM has been recognized. The original Morrow techniques evolved to a myectomy that is more extended laterally, beginning at the region corresponding to the nadir of the right coronary cusp and then proceeding leftward toward the mitral leaflet (at the posterior commissure) and the apex. A more extensive, deeper resection is performed, completely releasing the papillary muscles with the aim of redirecting anteromedial blood flow away from the MV. This broader approach resolves intraventricular obstructions, increasing LV diastolic filling and, consequently, enabling the ejection of a greater volume of blood with less risk of obstruction, less turbulent flow in the LVOT, and less SAM. MV approaches using different techniques are more commonly used by more surgeons in a greater number of patients. MV and papillary muscle anomalies are identified in the preoperative echocardiogram, and include anterior leaflet elongation, anterior motion of the MV caused by papillary muscle displacement, shortened chordae tendineae, and anomalous papillary muscle insertion directly into the valve leaflet. These anomalies are treated with surgical resection, which also includes resection of anomalous chordae, to allow complete mitral leaflet excursion and MR reduction, which will no longer be caused by SAM.^190 - 192^

Preoperative factors that determine good long-term prognosis are age < 50 years, LA diameter (LAD) < 46 mm, absence of AF, and male sex.^189 , 193^Recently, it was shown that posterior wall thickness (> 13 mm) is a long-term negative predictor, independent of septal thickness and severity of the LVOT pressure gradient.^194^

The difference in survival of patients with obstructive HCM who underwent myectomy is related to hemodynamic relief and improvement in diastolic function, and this is secondary to the reduction in subendocardial ischemia resulting from lower post-surgery intracavitary pressure.

The main complications of myectomy are complete AVB, VSD, and aortic valve regurgitation, but they are uncommon in experienced centers.^196 , 197^The latest AHA/ACC Guideline focuses on outcome targets for invasive surgical therapy ( Table 15 ). Although most patients with advanced symptoms (NYHA FC III-IV) should be referred for invasive therapy, a select group of less symptomatic patients but with other evidence of significant hemodynamic disturbances may be eligible for myectomy at experienced centers to minimize long-term structural and physiological damage. There is extensive literature reporting reversal of pulmonary hypertension, improvement in exercise tolerance capacity, LAD reduction, and reduction in ventricular arrhythmias.^12 , 198^

**Table 15:** Desired results with septal myectomy in HCM

Target results in surgical treatment	Desired results
30-day mortality	≤ 1%
Complications at 30 days (tamponade, infection, and major bleeding)	≤ 10%
Need for a pacemaker due to AV block	≤ 5%
Mitral approach in the first year	≤ 5%
Moderate-to-severe residual mitral regurgitation	≤ 5%
Need for reoperation	≤ 3%
Improvement in NYHA functional class	> 90%
LVOT pressure gradient (at rest or provoked) < 50 mm Hg	> 90%

HCM: hypertrophic cardiomyopathy; AV: atrioventricular; NYHA FC: New York Heart Association; LVOT: left ventricular outflow tract.

A study with 2,268 adult patients undergoing surgical myectomy at an experienced center compared early intervention (FC II) vs Class I indication (1,318 patients [58%] met class 1 indication and 950 patients [42%] underwent early surgery). At 6.2 ± 4 years of follow-up, patients who underwent early surgery showed a significant reduction in mortality and the need for the use of an ICD when compared with patients who met Class I indication (p < 0.001) and had a survival rate similar to a normal age-sex-matched population.^199^

The most appropriate invasive procedure for each patient should be decided on an individual basis and discussed with a Heart Team experienced in the treatment of HCM, considering the patient's state, coronary anatomy for septal ablation, and MV apparatus.^174^

Figure 16 shows which aspects should be considered when deciding on the most appropriate SRT strategy in patients with HCM.

**Figure 16:** Aspects to be considered when deciding which septal reduction therapy strategy should be used in patients with HCM. HCM: hypertrophic cardiomyopathy; ODT: optimal drug therapy; LVOT: left ventricular outflow tract; LBBB: left bundle branch block.

Figure 17 shows a schematic diagram of the main septal reduction therapies available: alcohol septal ablation and septal myectomy.

**Figure 17:** Schematic diagram of the main septal reduction therapies available: alcohol septal ablation and septal myectomy. Adapted from Nishimura and Holmes Jr.^200^

#### 4.2.3. Radiofrequency Ablation

Endocardial septal ablation with RF catheters was first described by Lawrenz et al. as a new modality, still considered experimental, of nonsurgical invasive treatment of patients with obstructive HCM. It aimed to reduce the intraventricular gradient as an alternative to ASA when the anatomy or territory supplied by the septal artery are not favorable.^201^

The technique consists of applying RF energy with catheters originally used to ablate arrhythmias in electrophysiological studies. RF energy is converted into thermal energy in the myocardium, causing tissue damage, perilesional edema and, subsequently, permanent scarring. The depth of the scar caused by RF varies from 5 to 12 mm, and its radial shape is well defined from the point of contact of the catheter with the myocardial tissue. Imaging, especially of the LV access route, is useful for choosing the technique (retroaortic or transseptal) and determining expected results, as well as for planning combined procedures in the case of ablation in preparation for TAVI. Another benefit is phenotype-based ablation target planning. When the hypertrophy only affects the basal region, less extensive applications are necessary than in situations where the hypertrophy extends into the middle or apical part.

In a recent meta-analysis, intraoperative mortality was less than 2%,^202^and 1-year mortality was 1.8%.^203 , 204^

A gradient reduction of 60% to 90% is expected. The rate of complete AVB and permanent pacemaker implantation was 0% to 25%, depending on the technique and aggressiveness selected by the different authors.^203^Valdigem et al.^205^reported a low rate of LBBB (approximately 10%) and no AVB or increase in AV interval with the indication of a permanent pacemaker. More aggressive ablations achieve greater reduction at the expense of increased complications due to outflow tract edema and conduction system involvement.^202^

Gradient reduction is progressive throughout the first 3 to 6 months of ablation, with delimitation of the area of fibrosis and LV remodeling. Some patients may experience additional reduction by the end of the first year.^205 , 206^

RF septal ablation can be considered in patients with obstructive HCM who remain symptomatic despite ODT when the surgical risk for septal myectomy is high, the coronary anatomy is not favorable for ASA, and the procedure is performed by a team experienced in RF ablation and electroanatomic mapping, when available, grade of recommendation IIB, level of evidence B ( Table 16 ).

**Table 16:** Recommendations for the use of radiofrequency ablation and team building

Recommendations	Grade of recommendation	Level of evidence
RF septal ablation may be considered in patients with obstructive HCM, a resting or provoked LVOT gradient > 50 mm Hg, who remain symptomatic (NYHA FC III-IV) despite optimal medical therapy when the surgical risk for septal myectomy is high, the coronary anatomy is not favorable for alcohol septal ablation, and the procedure is performed by a team experienced in RF ablation and electroanatomic mapping, when available.	IIb	B
RF septal ablation may be considered in patients with residual gradient after septal myectomy or septal alcohol ablation when redo surgery is not feasible or desired.	IIb	C
The team to perform RF septal ablation must include at least one electrophysiologist experienced in complex cases of RF catheter ablation, an echocardiographer experienced in evaluating patients with complex heart diseases, and a cardiologist with experience in intensive care.	IIa	C

RF: radiofrequency; HCM: hypertrophic cardiomyopathy; LVOT: left ventricular outflow tract; NYHA FC: New York Heart Association functional class.

#### 4.2.4. Risk Stratification and Prevention of SCD

SCD is the most visible and impactful complication of HCM.^1^One of the main therapeutic targets in managing the disease is to reduce the occurrence of this catastrophic event. Until the introduction of the ICD into clinical practice, which reliably terminates ventricular tachyarrhythmias, there was no preventive treatment for SCD.^7 , 207^In the past 2 decades, with the use of the ICD and the development and evolution of algorithms for the accurate identification of patients with HCM at high risk of SCD, a large reduction in HCM-related mortality was achieved. Recent cohorts report reductions from rates as high as 6% per year pre-ICD to 0.5% in tertiary centers.^7 , 207^

Still, there are some gaps to be filled in risk stratification, particularly the reduction of unnecessary implants.^208^The accurate identification of high-risk patients who need an ICD vs those who do not need one, based on the risk of SCD and the risks of complications inherent to ICD implantation, is a major challenge.^2 , 209^

#### 4.2.5. Risk Factors

Over the decades, several retrospective observational studies have been published that found clinical, demographic, and imaging variables to be associated with the risk of future ventricular tachyarrhythmias and SCD, which have come to be recognized as risk factors.^7 , 207 - 210^

These risk markers have evolved considerably over time. Some are no longer valued, such as blood pressure response to stress test, while others are considered risk markers only in certain circumstances or in association with other factors, such as NSVT.^2^Some risk markers have also been modified. For example, LVH was only considered massive if > 30 mm, but now values ≥ 28 to 29 mm are also accepted; and family history of SCD was only considered relevant if the family member was < 40 years of age, but this value has been adjusted up to 50 years.^2^New risk markers have emerged, such as the presence of apical aneurysm, EF reduction to ≤ 50%, and LGE on CMR.^2 , 209^

Risk factors currently recognized and valued for determining high risk of SCD risk and identifying patients for whom ICD implantation should be indicated as primary prevention include personal history (previous cardiac arrest, SVT, ventricular fibrillation, unexplained syncope), family history (SCD in the family), echocardiographic and CMR findings (massive LVH, EF < 50%, apical aneurysm, and LGE), and 24-hour Holter monitoring (presence of NSVT).^209 , 210^ Table 17 shows the characterization of risk factors for SCD in HCM.

**Table 17:** Characterization of risk factors for SCD in HCM

Personal history: previous sustained ventricular tachycardia/VF	Secondary prevention: high risk of recurrent events
Personal history: unexplained syncope	At least one episode of transient loss of consciousness not attributable to LVOT obstruction or vasovagal syndrome, especially when occurring in the past 6 months.
Family history: SCD	SCD definitively or likely attributable to HCM in ≥ 1 first- or second-degree relatives aged ≤ 50 years. Multiple SCDs in third-degree relatives should also be considered relevant.
Echo/MRI: LVH > 28 mm	Wall thickness ≥ 28 mm in any segment of the ventricular wall by echocardiography/MRI.
Echo/MRI: systolic dysfunction	EF ≤ 50% by echocardiography/MRI.
Echo/MRI: LV apical aneurysm	Presence: higher risk.
MRI: LGE > 15%	Diffuse and extensive LGE either quantified or estimated by visual inspection, comprising ≥ 15% of total LV mass.
Holter: NSVT	Significant when episodes are frequent (≥ 3), longer (≥ 10 beats), and faster (≥ 200 bpm).

17 SCD: sudden cardiac death; HCM: hypertrophic cardiomyopathy; VF: ventricular fibrillation; LVOT: left ventricular outflow tract; Echo: echocardiogram; MRI: magnetic resonance imaging; LVH: left ventricular hypertrophy; EF: ejection fraction; LV: left ventricle; LGE: late gadolinium enhancement; NSVT: nonsustained ventricular tachycardia.

Patient age also greatly influences the risk of SCD in patients with HCM and should be considered when making decisions about ICD. Cohorts show a very low SCD event rate in older (> 60 years) and clinically stable patients, a population for which the benefit of an ICD is uncertain.^40^Conversely, the risk of SCD is higher in children and adolescents when compared with adult patients.^211^A cohort study involving 150 patients of different age groups conducted in Sweden showed a higher risk of SCD in patients between 9 and 13.9 years of age (rate of 7.2% per year) when compared with patients aged more than 16 years of age (rate of 1.7% per year), with a risk ratio 3.75 times higher (CI 1.18-11.91).

##### 4.2.5.1. New Risk Factors

Left ventricular dysfunction (EF < 50%) and the presence of fibrosis and apical aneurysm have recently been recognized as risk factors because they were associated with overall mortality and SCD in several studies.^2^With regard to LV dysfunction, hypercontractility is a known pathophysiological mechanism of HCM.^2^When EF reduces to ≤ 50%, called "end-stage" HCM, the average survival after the development of ventricular dysfunction is 8.4 years, and factors such as the presence of fibrosis, multiple pathogenic/likely pathogenic sarcomeric variants, AF, and EF < 35% portend a worse prognosis.^212^

The presence of fibrosis (LGE on MRI) is a risk factor for reentry ventricular arrhythmias, whose onset has been associated with the triangle of substrate, trigger, and enabling environment.^213 , 214^

The absence of LGE can determine a better prognosis in patients with HCM and preserved function.^213 , 214^There is a linear increase in the risk of death with an increasing percentage of LGE up to a cutoff of 15%, when the risk curve shows a steeper slope (implying greater risk of death).^213 , 214^This is very useful as an arbitrary factor for patients in whom the determination of SCD death remains uncertain.^2 , 209^The absence of LGE is associated with a low risk of adverse events.^209^

Recent studies have associated the presence of apical aneurysm with either an akinetic or dyskinetic wall, independent of size, with SCD. Detection rates are higher with CRM than echocardiography.^2 , 209^

Therefore, the presence of at least one risk factor is sufficient to determine a high risk of SCD and justify ICD implantation.^2 , 7 , 207 - 210^Despite being treated independently, risk factors reinforce each other. For example, the presence of fibrosis is more relevant in patients with ventricular dysfunction than in those with preserved function.^2^

Because patients with HCM have a lifetime risk of SCD, periodic risk reassessment is a critical component of any longitudinal assessment.^2 , 207^Risk stratification is recommended at the initial visit and then annually.

The decision to implant an ICD requires informing the patient of all the long-term advantages and disadvantages of implanting a device, a process known as shared decision and which is very important in HCM, particularly when the indication for an ICD is uncertain.^2^

#### 4.2.6. SCD Risk Calculator

A tool to support decision-making regarding the best strategy for preventing SCD in patients with HCM is the HCM Risk-SCD calculator, which estimates the risk of SCD at 5 years and was constructed using a logistic regression equation which incorporates multiple risk factors.^3 , 215^The variables considered were age, LAD, peak LVOT gradient, presence of unexplained syncope, NSVT with ventricular rate > 120 bpm, and family history of SCD in first-degree relatives aged < 40 years.^3 , 215^This risk prediction model allows categorizing patients into three risk categories: low (< 4%), intermediate (4-6%), and high (> 6%); ICD implantation is recommended for patients at high risk.^215^

However, it should be noted that the risk prediction model does not take into account the impact of new markers of SCD, such as systolic dysfunction (EF < 50%), apical aneurysm, and LGE, and considers variables such as LAD and peak LVOT gradient which are not considered in the risk factor model and have not been clearly associated with SCD.^2 , 209 , 210^Additionally, the utility of the risk prediction model is uncertain in patients with HCM after septal myectomy or ASA, and is not recommended for risk stratification in children and adolescents under 16 years of age.^215^In 2019, a risk prediction model was developed for children aged 0 to 16 years.^216^It was retrospectively validated in 2022, with a risk ≥ 6% identifying 70% of events.^217^

A study with 3,703 patients (EVIDENCE-HCM) retrospectively analyzed the HCM Risk-SCD calculator and demonstrated that it has a moderate capacity to discriminate between high- and non-high-risk patients.^218^

A large number of retrospective cohorts have investigated the effectiveness of the risk prediction model (HCM Risk-SCD) in comparison with identification of risk factors in populations with known clinical outcome (with and without IDC).^207 , 209 , 219 - 222^In these studies, the risk prediction model (HCM Risk-SCD) had a sensitivity of 33% for identifying patients with subsequent SCD, while the risk factor-based approach had a sensitivity of 95%.^207 , 219 - 222^Regarding specificity, that is, identifying low-risk patients who do not need an ICD, the HCM Risk-SCD was superior, with a specificity of 92% vs 78% for the risk factor-based approach, with the potential benefit of reducing 20% of implants. However, the number needed to treat (NNT) to save 1 life was comparable between the two approaches (6.6 with the risk factor-based approach vs 7.2 with the HCM Risk-SCD). Conversely, the number of unnecessary implants could be lower if the cohorts had a longer follow-up period, as 36% of patients with HCM received their first therapy for high-risk arrhythmia (VT/VF) more than 10 years after ICD implantation.^223^

Based on the previously described evidence, an algorithm for ICD indication and SCD prevention in patients with HCM is shown in Figure 18 .

**Figure 18:** Algorithm for risk stratification of SCD and ICD indication in patients with HCM. VT: ventricular tachycardia; VF: ventricular fibrillation; LVH: left ventricular hypertrophy; SCD: sudden cardiac death; NSVT: nonsustained ventricular tachycardia; ICD: implantable cardioverter-defibrillator; HR: heart rate.

According to the algorithm, ICD implantation is recommended in patients exhibiting SVT, reversed SCD, or ventricular fibrillation (grade of recommendation I, level of evidence A).

Other patients with HCM who exhibit at least one of the risk factors for SCD (syncope, family history of SCD, massive LVH, apical aneurysm, LVEF < 50%) should be stratified according to age. Those under 40, including children, should be considered for an ICD; those aged between 40 and 60 years old should be considered for an ICD implantation after discussion at an experienced center; and patients over 60 years of age may be considered for an ICD ( Table 18 ).

**Table 18:** Recommendations for risk stratification of SCD and ICD implantation in patients with HCM.

Recommendations	Grade of recommendation	Level of evidence
The following risk factors should be investigated in every clinical evaluation: aborted cardiac arrest, VT/VF. Unexplained syncope, family history of SCD in individuals aged < 50 years, massive LVH (> 28 mm), apical aneurysm, LVEF < 50%.	I	C
In cases in which there are doubts regarding ICD indication, investigation of NSVT with prolonged monitoring and CRM to detect LGE and apical aneurysm is indicated.	I	C
The use of the HCM Risk-SCD calculator should be considered to assess the risk of SCD at 5 years in patients over 16 years of age in the shared decision process about ICD.	IIa	B
If there is evidence of reversed cardiac arrest due to SVT or VF, an ICD is recommended.	I	B
An ICD should be considered in patients aged ≤ 40 years in the presence of at least one of the following risk factors: unexplained syncope (not attributable to vasovagal syndrome or LVOT obstruction), massive LVH (septal thickness ≥ 28 mm), family history of SCD in individuals aged < 50 years, LV apical aneurysm, and systolic dysfunction (EF ≤ 50%).	IIa	B
An ICD should be considered in patients aged > 40 to ≤ 60 years in the presence of at least one of the following risk factors: unexplained syncope (not attributable to vasovagal syndrome or LVOT obstruction), massive LVH (septal thickness ≥ 28 mm), family history of SCD in individuals aged < 50 years, LV apical aneurysm, and systolic dysfunction (EF ≤ 50%) in shared decision.	IIa	B
An ICD may be considered in patients aged > 60 years in the presence of at least one of the following risk factors: unexplained syncope (not attributable to vasovagal syndrome or LVOT obstruction), massive LVH (septal thickness ≥ 28 mm), family history of SCD in individuals aged < 50 years, LV apical aneurysm, and systolic dysfunction (EF ≤ 50%).	IIb	B
An ICD should be considered in the presence of NSVT, especially in case of multiple (> 3), faster (HR > 200), and longer (> 10 beats) episodes, in individuals aged < 40 years.	IIa	A
An ICD may be considered in the presence of NSVT, especially in case of multiple (> 3), faster (HR > 200), and longer (> 10 beats) episodes, in individuals aged ≥ 40 years.	IIb	B

SCD: sudden cardiac death; ICD: implantable cardioverter-defibrillator; HCM: hypertrophic cardiomyopathy; VT: ventricular tachycardia; VF: ventricular fibrillation; LVH: left ventricular hypertrophy; LVEF: left ventricular ejection fraction; NSVT: nonsustained ventricular tachycardia; SVT: sustained ventricular tachycardia; LVOT: left ventricular outflow tract; LV: left ventricle; HR: heart rate.

Patients with none of the risk factors listed above but who have NSVT (multiple, longer [≥ 10 beats], and faster [≥ 200 bpm] episodes) should be considered for an ICD if they are younger (< 40 years, including children), while patients aged > 40 years may be considered for ICD ( Table 18 ). Patients without other risk factors who do not have NSVT may be considered for an ICD if LGE > 15% ( Table 18 ).

For patients who do not exhibit any of the risk factors, NSVT, or LGE > 15%, an ICD is not recommended ( Table 18 ).

It is worth noting that patients with multiple risk factors can be considered at higher risk of SCD, which favors the use of an ICD in all clinical situations shown in the flowchart.

#### 4.2.7. ICD Selection and Programming in Patients with HCM

After the decision to implant an ICD in patients with HCM, the next steps involve choosing the type of system to be implanted and adjusting its programming.^224^Currently, ventricular and AV ICD systems are available for intravascular access and ventricular ICD systems are available for subcutaneous access.

The choice of system depends on several clinical and technical aspects inherent to the patient and the arrhythmia, which should be identified and treated. The most relevant factors to be considered are ICD indication (primary or secondary prevention); age (children, young adult, older patients), anatomy (implant location and suitability), basic rhythm (stable sinus rhythm, bradyarrhythmia, or AF),^2^ease of intravascular access, and documented arrhythmia (sustained monomorphic VT or polymorphic VT/VF).

In patients with stable sinus rhythm and preserved AV conduction who have an ICD indication for primary prevention of SCD, the purpose of the device is to safely detect and terminate ventricular tachyarrhythmia. In this case, a single-coil transvenous ICD or a subcutaneous ICD is typically recommended, particularly in younger individuals, as these devices have lower risks of long-term complications.^226 - 228^When choosing a system, it should be considered that transvenous devices have the advantage of providing ventricular pacing in case of marked bradycardia; however, compared with subcutaneous devices, they have the disadvantage of using an intravenous lead, which may cause undersensing or pacing failure and pose a risk of infection and endocarditis, in addition to the risk involved in lead replacement (especially older ones).^229 - 231^The subcutaneous ICD, on the other hand, is easier to remove when necessary. However, the subcutaneous ICD is larger and is not suitable for individuals with very low body mass, has a shorter battery life, and a greater risk of inappropriate shocks due to T-wave or myopotential oversensing. Therefore, patients should be assessed before and after implantation. In addition, clinical experience with the subcutaneous ICD in Brazil is limited.^232 - 235^

For patients with HCM who present with recurrent monomorphic VT, the intravascular system is the best choice because it provides antitachycardia pacing (ATP), a therapy that terminates SVT episodes without the need for shocks. The ICD should be programmed to reduce appropriate and inappropriate shocks, and the ATP therapy should be activated.^34 , 236 , 237^

Single-chamber ICD systems usually have fewer short- and long-term complications compared with dual-chamber transvenous systems.^36 - 39 , 238 - 241^Furthermore, randomized clinical trials have also shown a marked reduction in LVOT obstruction by permanent RV pacing, with improvement of symptoms in patients with obstructive HCM.^242 , 243^However, long-term clinical results were not persistent, except in patients over 65 years of age.^244 - 248^

Dual-chamber ICDs have an additional right atrial (RA) lead, which helps differentiate ventricular tachycardia from supraventricular tachycardia; however, the rate of complications is higher when compared with single-chamber devices.^34 , 236 , 238 - 240^The risks and benefits of its use should be assessed according to the clinical characteristics of the patient and the potential benefits offered by the device.

The clinical benefit of cardiac resynchronization therapy (CRT) has been demonstrated in randomized clinical trials of patients with HF, LBBB, significant systolic ventricular dysfunction, ODT, and ischemic and nonischemic heart disease, but not in patients with HCM. However, isolated series involving patients with HCM demonstrated a favorable clinical response, but inferior to the response obtained in patients with ischemic and dilated cardiomyopathy. More recent studies suggest that the clinical response is compromised by the great extent of myocardial fibrosis in end-stage HCM, which is characterized by dilation and EF < 35%, without evidence of survival benefit. Furthermore, CRT responders showed only a slight improvement in LVEF.^244 , 248^The benefit appears to be more evident in patients with LBBB, a very prolonged QRS duration, and EF between 35% and 50%.^244 - 246^

Table 19 shows the recommendations for ICD selection.

**Table 19:** Recommendations for ICD selection

Recommendations	Grade of recommendation	Level of evidence
In patients with HCM with an ICD indication for SCD prevention, either a single- or dual-chamber transvenous ICD or a subcutaneous ICD is recommended. The device should be selected after a shared decision-making process that takes into consideration patient preferences, lifestyle, and expected potential need for pacing for bradycardia or sustained monomorphic VT termination.^20 - 32^	I	B
In patients with HCM who are receiving an intravenous ICD, single-coil systems are recommended.^226^	I	B
Dual-chamber ICDs should be considered for patients with a need for atrial or atrioventricular sequential pacing for marked bradycardia or significant conduction abnormalities, or as an attempt to relieve symptoms of obstructive HCM (most commonly in patients >65 years of age).^34 , 236^	IIa	B
In selected adult patients with HCM receiving an ICD who have NYHA class II to ambulatory class IV HF, left bundle branch block, and LVEF <50%, CRT should be considered for symptoms reduction.^244 - 248^	IIa	B
In patients with HCM in whom a decision has been made for ICD implantation and who have paroxysmal atrial tachycardias or AF, dual-chamber ICDs may be considered.^239 - 243^	IIb	B

HCM: hypertrophic cardiomyopathy; ICD: implantable cardioverter-defibrillator; VT: ventricular tachycardia; HF: heart failure; LVEF: left ventricular ejection fraction; CRT: cardiac resynchronization therapy; AF: atrial fibrillation.

### 4.3. Treatment of Atrial Arrhythmias

#### 4.3.1. Introduction

AF is the most common arrhythmia observed in patients with HCM, affecting approximately 20% of patients in experienced centers. Patients with HCM have a 6-fold greater likelihood of developing AF compared with the general population. AF is associated with increase in morbidity, risk of stroke, and worsening quality of life. AF treatment involves preventing thromboembolic events and controlling symptoms using rhythm or rate control strategies. The main predisposing factors are LA enlargement and age. Other factors possibly related to AF in patients with HCM are LVOT obstruction, P-wave duration > 140 ms, atrial tachyarrhythmias, ST-T segment changes on baseline ECG, ventricular extrasystoles, LGE on MRI, and abnormal coronary flow reserve.^2 , 3 , 33^

Strategies to predict the occurrence of AF in patients with CHM are important, such as the HCM-AF score, which has already been tested and externally validated. It predicts the probability of AF at 5 years (low, intermediate, and high risk), using variables such as atrium size, age, age at HCM diagnosis, and HF symptoms.^249^

#### 4.3.2. Drug Therapy

Maintaining sinus rhythm is a key strategy for the successful treatment of patients with HCM because a) loss of atrial contraction is clearly associated with worsening FC and increases the chance of hospitalization for HF; and b) early rhythm control has a greater chance of avoiding the vicious cycle between loss of atrial contraction and increased pressure and left atrial remodeling, increasing the long-term probability of achieving sinus rhythm.

Although the rhythm control strategy is preferred, as it is more effective in reducing outcomes than rate control,^27 , 250^drug treatment for rate control can be administered, preferably with a nondihydropyridine CCB, beta-blockers, or a combination of both. Digoxin should be avoided because there is a possibility it could exacerbate LVOT obstruction due to a positive inotropic effect.

In the rhythm control strategy, the use of the antiarrhythmic drugs amiodarone and sotalol (mainly in younger patients due to long-term side effects) was shown to be safe.^251 - 255^Due to the presence of significant LVH, the risk of proarrhythmia should be considered. Data on propafenone is limited, and its use should be reserved for patients with an ICD.^2^

The effectiveness of drug treatment is limited and lower than in patients without HCM, but the data are based on small, observational studies with a small number of patients.^255^A recent retrospective study with 98 patients^252^reported sinus rhythm maintenance in 62% of patients after 1 year of treatment and in only 42% after 3 years. Amiodarone was discontinued due to significant side effects in 19% of patients.

#### 4.3.3. Invasive Treatment

Catheter ablation is a minimally invasive, safe procedure that plays an important role in the management of patients with HCM. However, its pathophysiology is related to a greater degree of electrical and structural cardiac remodeling, with a predominance of dilation and diffuse interstitial fibrosis.

Recent studies have shown that the outcomes of catheter ablation in patients with HCM are less favorable than in patients with other conditions or without structural heart disease, with higher recurrence rates and a need for reoperation.^256 , 257^

In a recent study with only 111 patients^257^followed for 6 years, 61% of those undergoing ablation remained in sinus rhythm. Recurrences required reoperation and concomitant use of antiarrhythmic drugs.

Meta-analyses published in the last 5 years showed that the rate of recurrence was twice that of the control group.^258 - 260^However, with the concomitant use of antiarrhythmic drugs, the success rate after the first procedure was 75%. Early indication of catheter ablation appears to be important for maintaining sinus rhythm.

Therefore, the most used strategies for catheter ablation in patients with HCM usually involve more extensive approaches in addition to pulmonary vein isolation, such as linear lesions and ablation of triggers not associated with the pulmonary vein.^2^

In patients undergoing surgical myectomy, RF ablation may also be performed during the procedure. This strategy was associated with a 3-year AF-free survival rate of approximately 70%, with atrium size being the greatest predictor of recurrence.^27^

AV node ablation with pacemaker implantation as a form of rate control can be used in refractory cases. The use of a LA appendage occlusion devices was evaluated in small samples and is not recommended as standard care for this patient population.^2^

Table 20 shows the recommendations for patients with HCM and AF.

**Table 20:** Recommendations for patients with HCM and AF^1^

Recommendations	Grade of recommendation	Level of evidence
In patients with AF in whim rate control strategy is planned, either beta-blockers or CCBs are recommended (verapamil or diltiazem).	I	C
In patients with HCM and symptomatic AF, catheter ablation as part of the rhythm control strategy.	IIa	B
A rhythm control strategy with cardioversion or antiarrhythmic drugs can be considered.	IIa	B
In patients who require surgical myectomy, concomitant surgical AF ablation should be considered.	IIa	B

20 HCM: hypertrophic cardiomyopathy; AF: atrial fibrillation; CCBs: calcium channel blockers.

Table 21 shows the antiarrhythmic treatment options for patients with HCM and AF.^1^

**Table 21:** Antiarrhythmic treatment options for patients with HCM and AF^1^

Drug	Efficacy	Side effect	Toxicity	Notes
Propafenone	?	?	Proarrhythmia	Recommended for patients with an ICD
Sotalol	Moderate	Fatigue Bradycardia	Prolonged QTc Proarrhythmia	Use should be considered.
Amiodarone	Moderate-high	Bradycardia	Liver, lung, thyroid, skin, neurologic	Use should be considered.

#### 4.3.4. Anticoagulation

Systemic embolic events have a high incidence in patients with HCM, especially in patients with AF, and are associated with increased morbidity and mortality in this population.^261 - 263^

In a retrospective longitudinal cohort study including 4,821 patients with HCM and no history of AF and previous thromboembolic events, the cumulative incidence of embolic events such as stroke, transient ischemic attack (TIA), and peripheral embolism was 2.9% at 5 years and 6.4% at 10 years.^264^

AF is the most common sustained arrhythmia in HCM, with an estimated prevalence of 20% to 25% in this population, which is 4 to 6 times higher than the prevalence in the general population.^28 , 265^Furthermore, the presence of AF is associated with a high risk of thromboembolism in patients with HCM, with an estimated prevalence and annual incidence of thromboembolism of 27.1 and 3.8%, respectively.^266 - 269^

Patients with HCM develop AF at a younger age compared with the general population, and paroxysmal AF is present in 2/3 of cases.^250 , 270^However, the incidence of thromboembolism does not differ between patients with HCM and paroxysmal AF and those with permanent/persistent AF.^250 , 269^Furthermore, the risk of thromboembolism in AF is not related to the number of paroxysms.^271^

AF has a low risk of mortality in HCM, although paroxysmal episodes can impair quality of life.^33^For a long time, the emergence of AF in patients with HCM was considered a hallmark of increased mortality and morbidity, particularly when associated with LVOT obstruction, given the loss of atrial contribution to ventricular filling.^28 , 250 , 272^However, the data supporting this correlation was based on older treatments, in which the use of anticoagulation was minimal and amiodarone was the only antiarrhythmic available, and were obtained long before the widespread use of catheter ablation for AF and new direct oral anticoagulants (DOACs).^250^Recent analyses of patients undergoing contemporary HCM treatment no longer consider the emergence of AF an independent factor of increased morbidity and mortality.^27 , 33^LAD has also been considered a risk factor for thromboembolism and is associated with AF development.^272^In patients with HCM without AF, studies suggest that every 1 mm increase in LAD increases the risk of stroke-related death, in addition to the risk of thromboembolism increasing exponentially, increasing the LAD to up to 45 to 50 mm.^272^

Other factors associated with the risk of embolic phenomena in patients with HCM are advanced age, presence of symptoms and signs of HF, and the extent of LGE.^262 , 272 , 273^A LGE extent > 14.4% on CMR was considered an independent predictor of thromboembolic events in patients with HCM.^274^Furthermore, some factors associated with the onset of AF, such as LVOT obstruction, SAM, and MR may also increase thromboembolic risk.^275^

Embolic events can be prevented by initiating oral anticoagulation after the first AF episode. A low threshold for oral anticoagulation for prophylaxis of thromboembolic events is warranted in patients with HCM, usually following the first episode of AF recorded on surface ECG.^26 - 28 , 276^Thromboembolism predictive scores and models do not correlate well with clinical outcomes of patients with HCM,^249 , 272 , 277^and there is no consensus on which scoring system should be used.^278^The CHADS2 and CHA2DS2-VASc scores performed poorly in patients with HCM, and new predictive models for risk stratification have been created, but these models were not externally validated.^279^Currently, the most important therapeutic measure is the use of lifelong anticoagulation in all patients with HCM who have had at least one episode of AF, even if sinus rhythm is restored.^3^

The use of warfarin and, more recently, DOACs, such as dabigatran, rivaroxaban, apixaban, and edoxaban, has contributed greatly to the reduction of peripheral embolic events, stroke, and death (<1%/year).^33^A recent study found DOACS to be at least as safe and effective as warfarin in patients with HCM undergoing catheter ablation for AF.^280^

However, undertreatment of anticoagulant therapy remains a concern in clinical practice. Real-world data demonstrated that the use of oral anticoagulants in AF with HCM was suboptimal, with only 15.3% of patients using oral anticoagulants at the time of AF diagnosis, and only 61.8% received oral anticoagulants during the study period.^266^Asymptomatic episodes of AF detected on ambulatory monitoring are common in patients with HCM (in 25% of patients with implanted devices), and the overall burden of AF in an HCM population is likely underestimated.^33^However, clinical implications of short AF episodes in asymptomatic patients are unknown, although they are predictive of symptomatic AF in the future.^250^

In asymptomatic patients with subclinical AF detected by internal devices lasting less than 24 hours, the risk of stroke and systemic embolism is only half of that of patients with clinical AF detected on surface ECG.^281^However, the risk of bleeding remains high in this population. Therefore, current data are insufficient to support, in general, routine oral anticoagulation for episodes of subclinical AF lasting less than 24 hours. Ongoing studies, such as ARTESIA and NOAH, will provide relevant information for this clinical scenario.^282 , 283^In the absence of specific data for patients with subclinical AF and HCM, the decision to initiate oral anticoagulation in this setting should be made on an individual basis, and the results of ongoing studies in the general population with subclinical AF may help in the management of patients with HCM.

Patients with HCM and LV apical aneurysm are at high risk of sudden arrhythmic death and thromboembolic events.^284^Identification of this phenotype expands risk stratification and may lead to treatment interventions for potentially fatal complications. A significant proportion of these patients have a thrombus within the aneurysm or have suffered a thromboembolic event even in cases that had only small aneurysms.^78 , 285^This suggests that the akinetic apical aneurysm may promote intracavitary thrombus formation independent of size, raising strong consideration for anticoagulation in all patients with apical aneurysms.^286^In a cohort of patients with HCM and apical aneurysm, no embolic events occurred during the follow-up period in those receiving oral anticoagulation.^78^

Table 22 shows the recommendations for anticoagulation in patients with HCM and AF.

**Table 22:** Recommendations for anticoagulation in patients with HCM and AF.

Recommendations	Grade of recommendation	Level of evidence
The detection of one or more symptomatic AF episodes on surface ECG is sufficient to recommend oral anticoagulation with DOAC (or warfarin) after assessment of patients' individual risks for these medications.	I	B
Anticoagulation with DOAC as first-line option and vitamin K antagonists as second-line option is recommended, independent of CHA_2_DS_2_-VASc score.	I	B
In patients with HCM and asymptomatic AF detected by internal or external devices and lasting less than 24 hours, anticoagulation with DOAC as first-line option and vitamin K antagonists as second-line option is recommended, independent of CHA_2_DS_2_-VASc score.	I	C
The use of oral anticoagulants for prophylaxis of embolic events is recommended in patients with HCM and LV apical aneurysm, regardless of the size of the aneurysm, after assessing the patients' individual risks for the use of oral anticoagulants.	I	B
In patients with HCM and asymptomatic AF detected by internal or external devices and lasting more than 5 minutes but less than 24 hours, anticoagulation with DOAC as first-line option and vitamin K antagonists as second-line option should be considered, taking into consideration total AF burden, duration of AF episodes, risk factors for thromboembolism, and bleeding risk.	IIa	C
Antiplatelet agents do not provide adequate prevention of stroke in patients with HCM and AF and therefore should be avoided.	III	B

HCM: hypertrophic cardiomyopathy; AF: atrial fibrillation; ECG: electrocardiogram; DOAC: direct oral anticoagulants; LV: left ventricle.

### 4.4. Advanced HF and Heart Transplantation in HCM

#### 4.4.1. Definition

Patients with HCM can progress to two distinct phenotypes: obstructive (provoked or at rest) and nonobstructive. Although uncommon, affecting only 2% to 3% of patients in HCM centers, HF is responsible for the majority of deaths (2/3 of deaths).^33^The rate of progression to NYHA FC III-IV HF was estimated at 1.6% per year for the nonobstructive phenotype, 3.2% for provoked obstruction, and 7.4% for resting obstruction.^33^Nonobstructive HCM is common and usually well tolerated, but a minority of patients progresses to advanced HF.^33^

The obstructive phenotype, whether provoked or at rest, results from severe dynamic obstruction associated with a hyperdynamic LV with little or no fibrosis. It can be treated by SRT.^11^

In nonobstructive HCM, HF may be caused by progressive LV dysfunction associated with the replacement of myocardial tissue by extensive fibrosis and chamber remodeling. Although uncommon, this scenario results in advanced HF, is irreversible, is challenging to manage, and generally has a poor response to conventional pharmacological treatment. In many cases, as the disease progresses, heart transplantation, as well as other advanced therapies, should be considered.^33^

Conversely, young patients with nonobstructive HCM may also develop severe restrictive HF, a form of advanced HF that requires aggressive treatment despite maintaining EF values > 50%. Despite treatment, these patients may develop HF symptoms that are refractory to standard therapy and become potential candidates for heart transplantation.^287^

HCM is rarely the etiology in hospitalized patients with decompensated HF, with a mortality rate of 3.4%. Of 1,217,039 patients hospitalized with decompensated HF or cardiogenic shock, the etiology was HCM in only 6,040 patients (0.5%).^288^

Therefore, the diagnosis of advanced HF in patients with HCM is related to the presence of symptoms refractory to standard clinical treatment both in patients who developed ventricular dysfunction and in those who developed a severe restrictive pattern while maintaining preserved EF.^2^

The most recent update to the HF Association of the European and American Societies of Cardiology^289^guidelines on HCM uses the criteria described by Metra et al.^290^to define HF: refractory symptoms (NYHA FC III-IV) associated with severe LV dysfunction, or refractory symptoms associated with persistent NP elevation in patients with severe diastolic dysfunction. This definition was also incorporated into the update of the Brazilian Society of Cardiology HF guidelines and includes both patients with dilated and restrictive cardiomyopathy.^291^

#### 4.4.2. Clinical Management

##### 4.4.2.1. Pharmacological Therapy

In advanced HF secondary to end-stage HCM with progression to systolic dysfunction, the suggested treatment follows HF guidelines and is based on the use of drugs such as angiotensin-converting enzyme inhibitors/angiotensin II receptor blockers, beta-blockers, spironolactone, and diuretics. Additionally, consideration should be given to interrupting the use of negative inotropic agents, such as verapamil and diltiazem, or switching to beta-blockers. There are few reports on the use of sacubitril valsartan and sodium-glucose transport protein 2 inhibitors in this setting.^12^Usual therapy for HFrEF typically reduces symptoms but does not affect remodeling and does not alter the course of the disease.

In patients with delayed intraventricular conduction, CRT can reduce symptoms and even increase EF.^292^

Worsening of FC (NYHA III-IV) in HCM can occur without EF involvement (> 50%) in up to 50% of patients. In these cases, there is significant diastolic involvement with restrictive physiology and little ventricular dilation.^11 , 14^The clinical approach in general does not differ from therapies previously implemented to control the disease with beta-blockers, among others. However, the use of loop diuretics plays an important role in controlling blood volume and congestion in these patients.^287^

In several phenotypes, it is important to observe progressive LA dysfunction and its its potential progression to AF. The latter, when present, tends to sharply exacerbate symptoms in both presentations, increasing heart rate and left atrial pressure. As a strategy, priority should be given to maintaining and/or returning to sinus rhythm whenever possible.^293^

##### 4.4.2.2. Nonpharmacological Therapy

In HCM with advanced HF, there is important functional limitation. Therefore, engaging in light and, whenever possible, supervised exercises that do not induce symptoms is recommended.

Comorbidities, such as obesity, can exacerbate outflow obstruction, HF, and cause unsatisfactory clinical response.^294^In addition to assisting in systemic (peripheral skeletal muscle) control, obesity control has the additional benefit of improving respiratory dynamics and reducing dynamic postural changes in preload that, in these patients, can worsen LVOT obstruction and cause presyncope or syncope.^295^

#### 4.4.3. Indications for Heart Transplantation

Patients with drug-refractory HCM who are not candidates for other interventions and present with significantly impaired quality of life and life expectancy are candidates for heart transplantation. In this context, it is assumed that mainly patients with systolic dysfunction, reflecting disease progression, represent the majority of phenotypes. However, severe diastolic dysfunction can also be present. Recent data from the International Society for Heart and Lung Transplantation (ISHLT) estimate that 2% to 3% of heart transplant indications are secondary to HCM.

Since it is not common for these patients to be hospitalized for decompensated HF, not even with low cardiac output requiring inotropic support, this opens up the possibility of discussing, in our setting, the identification of the most severely ill patients who could eventually be prioritized for transplantation.

In the state of São Paulo, after deliberation by the Technical Division of the Department of Health, patients who are hospitalized requiring intravenous diuretics are included in this condition 3 priority profile, that is, similar to patients with other etiologies who are hospitalized requiring inotropic agents for less than 6 months.

#### 4.4.4. Indications for Ventricular Assist Device

The reduced ventricular cavities and restrictive physiology common to patients with HCM and advanced HF limit the indication of LVADs in this population. However, VAD implantation can be considered in patients who have a contraindication to heart transplantation or organ unavailability.

Some case series of patients with advanced HF and HCM demonstrated an increase in survival after implantation of a continuous flow LVAD in patients with an LV diastolic end diameter > 46 mm (46 to 50 mm).^296 - 299^

Figure 19 illustrates the treatment flowchart for advanced HF in HCM.

**Figure 19:** Treatment flowchart for advanced HF in HCM. HCM: hypertrophic cardiomyopathy; HF: heart failure; ECG: electrocardiogram; CPET: cardiopulmonary exercise testing; BNP: B-type natriuretic peptide; SRT: septal reduction therapy; LVEF: left ventricular ejection fraction; AF: atrial fibrillation; ACEi: angiotensin-converting enzyme inhibitors; ARA-II: angiotensin II receptor antagonists; LBBB: left bundle branch block.

Table 23 shows the recommendations for diagnostic and prognostic evaluation and treatment of patients with HCM and advanced HF.

**Table 23:** Recommendations for diagnostic and prognostic evaluation and treatment of patients with HCM and advanced HF

Recommendations	Grade of recommendation	Level of evidence
Cardiopulmonary exercise testing should be performed in patients with HCM and persistent symptoms to quantify the degree of functional limitation and contribute to the selection of potential candidates for heart transplantation.	I	B
Patients with HCM who develop LV dysfunction (LVEF < 50%) and advanced HF should receive HF guideline-directed drug treatment.	I	C
Patients with nonobstructive HCM who develop LV dysfunction (LVEF < 50%) and advanced HF are potential candidates for heart transplantation and should be considered for ICD implantation.	IIa	B
In patients with HCM who develop LV dysfunction (LVEF < 50%) and advanced HF, discontinuation of negative inotropic agents such as verapamil or diltiazem should be considered.	IIa	C^300^
Heart transplantation should be considered for patients with nonobstructive HCM who develop advanced HF with limiting symptoms or refractory malignant arrhythmias despite optimal guideline-directed medical therapy.	I	C
Patients with nonobstructive HCM with LV dysfunction who develop advanced HF with limiting symptoms refractory to guideline-directed therapy should be considered for ventricular assist device if heart transplantation is contraindicated.	IIa	C

23 HCM: hypertrophic cardiomyopathy; LV: left ventricle; LVEF: left ventricular ejection fraction; HF: heart failure; ICD: implantable cardioverter-defibrillator.

### 4.5. Rehabilitation and Physical Activity

Physical activity plays a key role in preventing cardiovascular diseases. Although SCD during physical activity is a rare event, HCM still accounts for the largest number of events, translating into a challenge for clinical practice when deciding whether to recommend physical activity to a patient with HCM.^301^Factors intrinsic to the disease (genetic predisposition, adrenergic surges) as well as possible extrinsic factors (dehydration, use of anabolic steroids, and environmental factors) can predispose individuals to SCD.^301^An approach for assessment prior to physical activity participation in patients with HCM is suggested including SCD risk stratification, LVOT gradient, LV blood pressure response to exercise, exercise-induced ventricular arrhythmia, and suggested exercise intensity^217 , 301^( Table 24 ).

**Table 24:** Risk stratification for prescribing physical activity according to each patient's profile

	Asymptomatic + good exercise capacity	Symptoms related to HCM but unrelated to exercise	History of cardiac arrest, syncope, or exercise-induced symptoms
HCM Risk-SCD score at 5 years	Low risk (< 4%)	Moderate risk (≥ 4% and < 6%)	High risk (≥ 6%)
LVOT gradient	No/low (< 30 mm Hg at rest or exercise)	Moderate (30 to 49 mm Hg at rest or exercise)	High (> 50 mm Hg at rest or exercise)
BP response to exercise	Normal	Attenuated (< 20 mm Hg increase in systolic BP)	Systolic BP drop
Exercise-induced arrhythmia	No arrhythmia	Exercise-induced ventricular extrasystoles	Exercise-induced NSVT or SVT
Suggested exercise intensity	High intensity	Moderate intensity	Low intensity

LVOT: left ventricular outflow tract; BP: blood pressure; NSVT: nonsustained ventricular tachycardia; SVT: sustained ventricular tachycardia; HCM: hypertrophic cardiomyopathy. Adapted from Semsarian et al.^301^

Engaging in moderate-intensity aerobic physical activity is associated with improvements in cardiovascular health and survival in the population.^302^Although, historically, physical activity has not been recommended in patients with HCM, several initiatives have evaluated the safety of physical activity in these patients in controlled and monitored outpatient clinics.^303 , 304^Initial experience with 20 patients enrolled in a rehabilitation program in a single center demonstrated an improvement of at least one functional class by 50% and in functional capacity.^303^In a recent observational study, 32 individuals with HCM completed at least 3 months of rehabilitation with benefits in functional capacity.^304^

A randomized clinical trial included individuals with HCM aged 18 to 80 years, excluding those with a history of exercise-induced syncope or ventricular tachycardia, medically refractory LVOT obstruction (under evaluation for SRT), history of hypotension (> 20 mm Hg decrease in systolic blood pressure), cardiac decompensation or ICD implantation or SRT in the previous 3 months, and LVEF < 55%, among others. Included patients were randomized to usual physical activity or cardiac rehabilitation. The results showed an increase of 1.35 (95%CI 0.50-2.21) mL/kg/min in VO_2_in the exercise training group, with a between-group difference of 1.27 (95%CI 0.17-2.37) mL/kg/min at 16 weeks of training. Although the study was not powered to assess safety, it is important to highlight that no major adverse events occurred in either group, such as death, aborted SCD, appropriate ICD shock, or SVT.^300^Therefore, unsupervised physical activity may be beneficial and safe in patients with HCM without SCD risk markers. In individuals with an abnormal blood pressure response to exercise or arrhythmias, as mentioned above, physical activity should preferably be performed in a supervised environment.^300 , 303 , 304^

Table 25 shows the recommendations for participation in physical activity in patients with HCM.

**Table 25:** Recommendations for participation in physical activity in patients with HCM

Recommendations	Grade of recommendation	Level of evidence
Risk stratification is recommended for classifying patients regarding the prescription of physical activity using specific scores and exercise-induced parameters.	I	C
Cardiopulmonary rehabilitation with a structured, unsupervised training program is recommended for patients with HCM with the aim of improving functional capacity in low-risk individuals.	I	B
Cardiopulmonary rehabilitation may be considered in patients with HCM in the presence of LVOT gradient ≥ 50 mm Hg at rest/Valsalva, in a supervised environment.	IIb	C
High- or moderate-intensity cardiopulmonary rehabilitation is not recommended for individuals with HCM and a previous history of exercise-induced syncope, ventricular tachycardia, or aborted SCD.	III	C^301^

HCM: hypertrophic cardiomyopathy; LVOT: left ventricular outflow tract; SCD: sudden cardiac death.

### 4.6. Perioperative Assessment in Noncardiac Surgery

Patients with HCM are at increased risk of perioperative cardiac complications in noncardiac surgery. Epidemiological evidence is scarce and conflicting regarding mortality, but it unequivocally identifies an increased risk of cardiovascular complications in patients with HCM. In a retrospective U.S. series, the incidence of myocardial infarction and death after noncardiac surgery was compared between 227 patients with HCM and 554 controls without HCM, matched for year of operation, sex, and age.^1 , 305^After adjusting for factors such as history of arrhythmia, HF, coronary artery disease, diabetes, and hypertension, the presence of HCM almost tripled the odds of myocardial infarction or death (odds ratio [OR]: 2.82, 95%CI 2.59-3.07) and increased the odds of death by 61% (OR: 1.61, 95%CI 1.46-1.77).^305^

Increased mortality was not observed in other series, but an increased incidence of cardiovascular complications was consistently observed, notably HF decompensation.^306 , 307^

The rationale for perioperative cardiovascular complications in patients with HCM is quite plausible, and the perioperative period presents a hemodynamic challenge for patients with HCM. Volume shift and peripheral vasodilation, with alterations in preload and afterload, can increase resting LVOT gradient or even provoke LVOT gradient in patients with HCM without obstruction. Furthermore, perioperative blood pressure and red blood cell fluctuations can destabilize the already difficult balance between myocardial oxygen supply and demand in patients with HCM. These patients already present, in their baseline condition, increased oxygen demand due to hypertrophied myocardium and impaired oxygen supply resulting from increased LV end-diastolic pressure, or even concomitant coronary artery disease. These patients are also at increased risk of arrhythmias due to adrenergic stimulation, worsening of hemodynamic repercussions, or electrolyte imbalance.

There are no intervention studies in the perioperative period of noncardiac surgery in patients with HCM to support specific recommendations in this setting. However, risk stratification and medical therapy should be optimized before elective surgery. Ideally, patients with HCM should undergo surgery in centers of excellence, including a team of anesthesiologists, intensivists, and cardiologists experienced in the management of heart disease. Patients without a Doppler echocardiogram performed in the previous year or who have had a change in symptoms during this period should repeat it before procedures with moderate to high intrinsic risk. A 12-lead ECG, ultrasensitive troponin assay, and BNP measurement should also be performed, even in patients with functional class I or II, becoming postoperative monitoring parameters in the case of procedures with moderate to high intrinsic risk. Medications used to control HCM, notably beta-blockers, should be continued in the perioperative period. Patients with HCM should be evaluated regarding their volume status and, ideally, be operated on early in the morning, avoiding a long fasting period and possible hypovolemia due to dehydration. For long-lasting procedures and/or large volume shifts, hemodynamic monitoring with invasive blood pressure measurement and central venous catheter is mandatory, providing goal-directed hemodynamic therapy. In experienced centers, the use of intraoperative transesophageal Doppler echocardiography should be considered to better assess blood volume and changes in the intraventricular gradient. Monitoring should continue after the end of surgery, necessarily within the first 48 hours, with cardiac reassessment and daily measurements of BNP and ultrasensitive troponin for early detection of decompensation.

Specific anesthetic techniques, such as the use of agents with less hemodynamic impact, should be observed since the induction of anesthesia, but the preference for general or regional anesthesia is quite controversial. In the previously described series, no association was observed between general or regional anesthesia and adverse outcomes, but the potential for an abrupt drop in venous return and peripheral vasodilation associated with peripheral nerve blocks makes this technique less attractive for patients with HCM, notably those who are symptomatic and have substantial LVOT gradients. In the case series by Dhillon et al.,^307^in an experienced HCM center, general anesthesia was used in 89% of patients.

Finally, perioperative assessment also provides an opportunity for diagnosing HCM. In elective procedures, it is recommended that risk stratification and specific treatment be conducted before noncardiac surgery (see dedicated topics in this guideline).

Table 26 shows the recommendations for perioperative assessment in noncardiac surgery in patients with HCM.

**Table 26:** Recommendations for perioperative assessment in noncardiac surgery in patients with HCM

Recommendations	Grade of recommendation	Level of evidence
ECHO is recommended for patients in whom HCM is suspected, in cases where the last ECHO was performed more than 1 year ago, and/or in those with a change in symptoms.	I	C
Beta-blockers should be continued throughout the perioperative period.	I	C
ECG, BNP or NT-proBNP, and troponin measurements should be performed before moderate- to high-risk operations and repeated immediately after the procedure and once daily on postoperative days 1 and 2.	I	C
Hemodynamic monitoring with invasive blood pressure measurement, central venous catheter, and goal-directed hemodynamic therapy should be considered during procedures with high intrinsic risk.	IIa	C
Transesophageal ECHO may be considered during high-risk operations to assess blood volume and intraventricular gradient in experienced centers.	IIb	C

ECHO: echocardiography; HCM: hypertrophic cardiomyopathy; ECG: electrocardiogram; BNP: B-type natriuretic peptide; NT-proBNP: N-terminal prohormone of B-type natriuretic peptide.

### 4.7. Multidisciplinary HCM Centers of Excellence

HCM is a complex disease with a broad clinical and genetic spectrum. Despite being the most common genetic heart disease and relatively common in the general population, HCM is not frequent in clinical practice for most cardiologists.^308 , 309^Symptomatic cases are often mismanaged, taking months to years to reach the correct diagnosis.^310^Furthermore, the complexity and great variability in clinical presentation can lead to difficulties in managing patients, especially those with more severe disease. Therefore, referral of these patients to specialized HCM centers that have established protocols of excellence for the diagnosis and treatment of HCM should be considered ( Table 27 ). HCM centers should provide specialized care and advanced therapeutic interventions ( Table 28 ), including genetic testing and counseling, ICD implantation, exercise echocardiography, CMR, ASA, surgical septal myectomy, and management of arrhythmias, thus allowing patients and their families to make more appropriate choices, supported by the best available evidence.^308 , 309^Specialized HCM centers also enable physicians to gain expertise when caring for a large number of patients with HCM, in addition to enabling patients to be included in multicenter research protocols investigating new therapies.

**Table 27:** Recommendations for referrals and establishment of HCM excellence centers

Recommendations	Grade of recommendation	Level of evidence
Decision-making regarding advanced treatment options in patients with HCM should be made through shared discussions with a multidisciplinary Heart Team.	I	C
Patients diagnosed with HCM requiring a specialized diagnostic and/or therapeutic approach should be considered for referral to or joint follow-up at a center of excellence.	IIa	C
It is recommended that patients requiring septal reduction therapy be referred to a specialized HCM center that has obtained excellence of results in these interventions.	I	C

HCM: hypertrophic cardiomyopathy.

**Table 28:** Health professionals participating in the Heart Team and procedures to be offered in HCM excellence centers

Physicians	Cardiologist (adult and pediatric)
	Echocardiography specialist
	Cardiovascular magnetic resonance and cardiac computed tomography specialist
	Electrophysiology specialist
	Pacemaker and ICD specialist
	Cardiac surgeon
	Interventional cardiologist
	Geneticist
Other health professionals	Nurse
	Psychologist
	Dietitian
	Social worker
	Gynecologist - obstetrician
Diagnostic methods	Genetic testing
	Genetic counseling
	Echocardiogram at rest and exercise
	Cardiac magnetic resonance
	Cardiac computed tomography
	*24-hour Holter monitoring*
	Long-term arrhythmia monitoring
	Electrophysiologic testing
	Exercise stress testing
	Cardiopulmonary exercise testing
	Coronary cineangiography
	Invasive hemodynamic monitoring
Therapeutic procedures	Surgical septal myectomy
	Alcohol septal ablation
	AF ablation
	ICD implantation

HCM: hypertrophic cardiomyopathy; ICD: implantable cardioverter-defibrillator; AF: atrial fibrillation.

A factor to be considered when referring patients is the identification of the need for SRT, which has shown better results and low rates of mortality and complications in specialized HCM centers with experience in these procedures ( Table 27 ).^310^

Multidisciplinary care is essential for proper organization of a specialized HCM center, within the Heart Team concept, with the recommended participation of trained health professionals, as listed in Table 28 .^308 , 309^

In broader terms, the main purpose of health professionals involved in centers of excellence for HCM, as well as in other health service activities in Brazil, is to offer every patient the "right care," that is: effective (relevant and evidence-based), for the right patient, at the right time (opportune time), in the right way (trained health professionals within multidisciplinary teams), producing the right outcome (reduction of relevant clinical outcomes), promoting engagement and shared decision-making involving patients and their families efficiently and, ultimately, promoting value in health.^311^

Furthermore, in the view of the Department of Heart Failure (DEIC) /Cardiomyopathy Study Group (GEMIC) /Brazilian Society of Cardiology (SBC), specialized HCM centers should be committed to transforming the care of patients with HCM. To this end, they should connect education/training of health professionals with the defense and education of the lay public about the disease, with the involvement of patients, families, and patient associations, in addition to promoting clinical research and providing the setting for incorporation of new technologies, by developing translational research centers and digital medicine (telecardiology). Moreover, they should incorporate good management practices (protocols, indicators, continuous quality improvement tools, and commitment to a patient safety culture), disseminate their results and seek certifications, become involved in national and international registries, and develop networking mechanisms.

### 4.8. Special Clinical Situations in HCM

#### 4.8.1. HCM in Children and Adolescents

Although understanding of the genetic, molecular, and physiologic mechanisms of HCM has grown substantially, optimal assessment strategies and medical-surgical management are still insufficient in the pediatric population. Of particular interest is the wide variability in clinical presentation across the pediatric age groups.

HCM has an estimated incidence of 0.24 to 0.47 per 100,000 children per year, accounting for 25% to 50% of all cardiomyopathies diagnosed during childhood and adolescence. It is the second most observed phenotype in pediatrics according to registries in North America, Finland, and Australia.^312 - 314^Pediatric HCM has a lower prevalence than that of the adult population.^315 , 316^

The incidence of HCM peaks in the first year of life, when its diagnosis is 3 times as high as that in other pediatric age groups, followed by a second peak during adolescence.^312 , 314 , 316 - 318^

HCM is an autosomal dominant disease with variable expression and penetrance related to the age at which the phenotype/symptoms appear. Although the etiology of pediatric HCM is heterogeneous and includes inborn errors of metabolism, neuromuscular disorders, and malformation syndromes, its clinical presentation is mainly due to pathogenic variants in genes that encode sarcomere proteins.^318 - 321^When HCM is diagnosed in infants under 1 year of age, the presence of a positive family history or the detection of pathogenic or likely pathogenic sarcomere gene variants is less common, unlike what is observed when the presentation occurs among children and adolescents.^316^

##### 4.8.1.1. Natural History of HCM in Childhood and Adolescence

Due to the rarity of the incidence of HCM in pediatrics, its natural history remains poorly understood.^316^

In children, HCM may be progressive and reach maximal phenotype only between the second and third decades of life.^322^Diagnosis during the first year of life is associated with a substantially worse prognosis than diagnosis after the age of 1 year.^321^The annual mortality rate of pediatric HCM is higher than that in adults, and more than half are associated with SCD in asymptomatic patients.

The clinical presentation depends on the underlying cause, and findings from the evaluation assist in the etiologic investigation.^318 - 320^Some characteristics of hypertrophic phenotypes resulting from inborn errors of metabolism and malformation syndromes can be observed in thorough history-taking and detailed physical examination. These are most frequently diagnosed in infants and are accompanied by delays in developmental milestones with cognitive impairment, presence of dysmorphic features, and involvement of other organs and systems, such as neurologic, hepatic, and renal abnormalities.^319 , 320^Identifying these etiologies is essential to direct the available therapies and determine the prognosis.^318 , 323 , 324^

Marston et al.^316^sought to characterize the natural history of HCM through the analysis of a multicenter cohort of 1128 children and adolescents aged 1 to 18 years obtained from the SHaRe registry. Patients diagnosed with sarcomeric HCM in this age group were at increased risk of developing ventricular arrhythmias and requiring advanced HF treatment. In addition, the presence of a sarcomeric variant on genetic testing was associated with a 67% increased risk of cardiac outcomes, with a greater than two-fold increased risk of developing HF.

SCD and advanced HF are the main complications in patients with HCM, with SCD being particularly common in adolescents and young adults. Age greater than 30 years and a family history of premature SCD are risk factors for SCD.

Advanced HF is the natural course of the disease in many patients. After the onset of HF, the progression to death is very rapid. Young age at diagnosis, a family history of HCM, and greater wall thickness are associated with a greater likelihood of developing advanced HF.

SCD is the main cause of death in children with the HCM phenotype, especially in adolescents and young adults, occurring less frequently in adults,^217^and it can occur in previously healthy patients.^320^There is a positive association between age and risk of SCD, which correlates with a higher penetrance of SCD among adolescents.^325^

Classically, the following are considered SCD risk markers: unexplained syncope, family history of HCM-related SCD, massive ventricular hypertrophy, and NSVT.^33^In a meta-analysis, Xia et al.^326^observed that the presence of syncope, NSVT, and LVOT obstruction is associated with the risk of SCD in children with HCM. Conversely, a family history of SCD was not a risk factor, unlike what was observed in the European Society of Cardiology (ESC) Guidelines.^326 , 327^

Identifying patients who would benefit from ICD implantation for primary prevention of SCD remains a challenge in pediatrics. ESC risk scoring cannot be used below age 16 years.^33 , 325^Recently, Norrish et al.^327^proposed a risk calculator (the HCM Risk-Kids model) using noninvasive variables, such as unexplained syncope, maximal LV wall thickness, LA diameter, LVOT gradient, and NSVT. An external validation of this method has been recently performed showing that the HCM Risk-Kids provides a validated individualized assessment tool for children with HCM, which can guide the decision-making on ICD implantation.^217^

#### 4.8.2. Pregnancy and Childbirth in Women with HCM

Pregnancy represents a potential risk to women with HCM because, depending on the pathogenicity of the disease, increase in cardiac output, reduction in peripheral vascular resistance,^328^and maternal hypercoagulable state,^329^any of these physiologic changes that occur during pregnancy may lead to complications, such as HF, arrhythmias, and thromboembolism.

1. Reproductive counseling: women with HCM should plan their pregnancy according to presumed risk factors for maternal-fetal prognosis, considering symptoms, degree of heart involvement (assessed by TTE and/or CMR), presence of obstruction, previous therapy, and genetic testing.^330 - 332^

1. A. Maternal risk assessment: it should be supported by the modified World Health Organization (WHO) classification, which includes WHO risk classes I, II, III, and IV,^333 , 334^as follows:

- WHO class II (HCM without LVOT obstruction or complicating factors): most patients have an uneventful pregnancy with no complications;
- WHO class III (HCM with important LVOT obstruction, symptomatic arrhythmias, and/or moderate ventricular systolic dysfunction): great likelihood of complications, and patients should be counseled against pregnancy;
- WHO class IV (HCM with severe and symptomatic ventricular dysfunction or LVOT obstruction): high risk of complications, and pregnancy is contraindicated.

Complicating factors are considered markers of a worse prognosis for pregnancy, such as a history of HF, complex ventricular arrhythmia, paroxysmal or permanent AF, and family history of SCD.^335^Often, when planning a pregnancy, the opportunity arises for a discussion with specialists about an indication for ICD or catheter ablation of complex and/or symptomatic arrhythmias, in cases that fall into a Class IA recommendation. Women undergoing SRT are not included in the WHO risk categories due to a lack of data for such classification.

1. B. Genetic evaluation: when planning a pregnancy, it is essential to consider the Mendelian autosomal dominant inheritance of HCM, which can also be caused by mutations in genes that encode sarcomere components.^336^Screening using genetic testing or imaging and ECG should be performed at this time, and the pathogenicity of detected variants should be considered in counseling.
2. Approach during pregnancy: multidisciplinary follow-up, maternal and fetal echocardiography, and 24-hour Holter monitoring of arrhythmias are routine practices during pregnancy in patients with HCM.^337^If drug treatment is required, beta-blockers, propranolol, or metoprolol succinate, whether or not combined with CCBs, preferably verapamil, are safe, non-teratogenic, and effective in controlling symptoms. Daily doses and drug combinations should be adjusted for the risk of arterial hypotension. ICD implantation should be considered in patients who fall into a Class IA recommendation for its indication. Radiofrequency ablation with electroanatomic mapping should be indicated in cases of arrhythmia leading to hemodynamic instability with poor response to medical treatment.^334 , 337^

Patients with HCM and persistent or paroxysmal AF are at markedly increased risk of stroke, so oral anticoagulation with warfarin should be considered, independent of CHA_2_DS_2_-VASc score.^338^Because rapid AF is often poorly tolerated in patients with HCM, maintenance of sinus rhythm and rate control are the main goals of treatment and can be achieved with electrical cardioversion, which is not contraindicated during pregnancy.^334 , 337^

**Obstetric considerations:** pregnancy in women with HCM is associated with low birth weight and prematurity. Vaginal delivery is considered safe,^339^while cesarean delivery is reserved for patients within WHO classes III/IV or for cases where there are obstetric indications, such as intrauterine growth restriction and/or fetal distress. The use of prostaglandins is not recommended for labor induction due to the inherent vasodilatory effects of this drug. Block anesthesia may be considered in patients within WHO class II, while general anesthesia is particularly reserved for patients within WHO class IV to provide greater maternal safety.

Although molecular genetic studies over the last decade have greatly contributed to the understanding of the clinical and genetic heterogeneity of HCM, the complexity of this disease does not yet allow the determination of its true incidence in apparently healthy newborns who do not show imaging abnormalities, such as on TTE.^2^In any case, genetic testing of asymptomatic children and adolescents with a family history of HCM has identified "healthy" mutation carriers. However, there are important barriers to the clinical application of genetic testing, including genetic heterogeneity, low frequency with which each causal mutation occurs in the general population with HCM, and methodological difficulties such as the identification of a single pathogenic mutation among 10 different genes and technical limitations of laboratory testing.

Table 29 shows the recommendations for pregnancy and delivery in women with HCM.

**Table 29:** Recommendations for pregnancy and delivery in women with HCM

Recommendations	Grade of recommendation	Level of evidence
The use of the modified WHO classification for pregnancy risk stratification is recommended.	I	C
The use of cardioselective beta-blockers and calcium channel blockers is recommended to control symptoms, supported by monitoring of fetal growth and vitality.	I	C
Electrical cardioversion should be considered in persistent or poorly tolerated atrial fibrillation.	II	C
Anticoagulation with vitamin K antagonists or low-molecular-weight heparin should be considered in atrial fibrillation, depending on gestational age.	IIa	C
Vaginal delivery is the first choice for patients within modified WHO class I/II, and cesarean delivery should be considered for patients within modified WHO class III/IV.	IIa	C

WHO: World Health Organization.
